# Can school children influence adults’ behavior toward jaguars? Evidence of intergenerational learning in education for conservation

**DOI:** 10.1007/s13280-019-01230-w

**Published:** 2019-08-21

**Authors:** Silvio Marchini, David W. Macdonald

**Affiliations:** 1 Wildlife Ecology, Management and Conservation Lab, Forest Science Department, Luiz de Queiroz College of Agriculture, University of São Paulo, Avenida Pádua Dias 11, Piracicaba, SP 13418-900 Brazil; 2 Wildlife Conservation Research Unit, Department of Zoology, The Recanati-Kaplan Centre, University of Oxford, Tubney House, Abingdon Road, Tubney, OX13 5QL UK

**Keywords:** Behavioral change, Carnivores, Environmental education, Human–wildlife conflict

## Abstract

A school-based experiment was conducted in the Brazilian Amazon to examine the effects of passively received information versus active elaboration on the ‘perceptions’ of jaguars (*Panthera onca*) among students, and the effects of information communicated via illustrated book on those perceptions among student’s parents. Books distributed via school decreased fathers’ perceptions of social acceptance of jaguar killing, but the same books distributed via a conservation organization did not. This suggests that fathers were influenced not only by the information explicitly conveyed in the content of books, but also by the implicit message that jaguar conservation was socially supported. Elaboration alone produced more persistent effects than information alone, but some negative attitudes were reinforced. Information and elaboration combined created stronger and more enduring effects than either intervention alone. These findings are important in designing interventions for our coexistence with jaguars and other charismatic species worldwide.

**Electronic supplementary material:**

The online version of this article (10.1007/s13280-019-01230-w) contains supplementary material, which is available to authorized users.

## Introduction

Conservation education recognizes the central role of people in conservation efforts and is designed to increase knowledge, improve attitudes and change behaviors toward natural resources in general, and wildlife in particular (Patrick et al. 2007). Conservation education has targeted both children and adults, with varying levels of success (Gore et al. 2006; Sponarski et al. 2016) because knowledge and attitudes often do not directly influence behavior (Heimlich et al. 2013). Less explored has been the potential of child-oriented communication interventions to reach families of students and the community through intergenerational influence (Ballantyne et al. 2001; Vaughan et al. 2003; Duvall and Zint 2007; Damerell et al. 2013). Child-to-adult influence may be particularly promising in the management of conflicts over the conservation of charismatic species that threaten human livelihoods (often referred to as human–wildlife conflicts; Redpath et al. 2015) for two reasons: (i) such species have strong prominence in the minds and hearts of both children and adults so that participants are more likely to engage in learning, and (ii) in addition to transferring knowledge and affecting attitudes, children can conceivably influence social norms among adults, and there is a growing evidence that social norms play a central role in human–wildlife conflict (Marchini and Macdonald 2012; Kansky et al. 2016). Despite the growing body of literature providing evidence for bi-directional influence between parents and children (Damerell et al. 2013), little is known about how education can be transferred from children to adults, and indirectly induce targeted behavioral changes in the context of human–wildlife conflict.

As the largest wild felid in the Western Hemisphere, and an opportunistic predator, the jaguar (*Panthera onca*) poses a direct and recurrent threat to large livestock and to human safety (Marchini et al. 2017) and is the most emblematic species involved in human–wildlife conflicts in Brazil (Marchini and Crawshaw 2015) and Latin America. The persecution of jaguars is assumed to be a direct response to conflict. However, the killing of jaguars by farmers has been shown to be determined by their attitudes towards jaguar killing and their perceptions of how common such behavior is among their neighbors (Marchini and Macdonald 2012, 2018) and, therefore, changes in these factors should produce behavior changes (i.e. tolerate jaguars instead of persecute them).

Reaching farmers in rural Latin America—in Amazonia, in particular—and effectively improving their perceptions of jaguars, however, remains a challenge. As well as economic incentives (e.g. monetary compensation for livestock loss; Dickman et al. 2011) and legal prohibitions and sanctions (e.g. establishment of protected areas) to dissuade people from killing jaguars, there have been a few attempts to foster tolerance to the species by increasing knowledge. These efforts have focused on providing landowners with factual information—mostly through printed manuals (e.g., Hoogesteijn 2000; Marchini and Luciano 2010)—on the importance of jaguar conservation and on how to prevent predation problems. Nonetheless, the cost-effectiveness of a communication campaign based on print media is lower in areas with low human density, difficulty of access due to poor road conditions, and high rates of adult illiteracy. A communication strategy whereby school children act as catalysts of perception change among their parents and other community members could be, therefore, an effective means of mitigating conflict and preventing jaguar killing in rural Amazonia.

In this study, we examined the effect of a school-based approach on “perceptions of jaguars” among students and their fathers on the Brazilian Amazon deforestation frontier, more precisely, on (i) knowledge about jaguars, (ii) attitude to jaguars, (iii) perceived impact of jaguars on livestock, (iv) perceived impact of jaguars on human safety, (v) attitude to killing jaguars, and (vi) perceived social norm regarding jaguar killing. We conducted an in-classroom experiment to compare the effect of passively received information versus active elaboration information on the perceptions of jaguars among 5th and 6th graders (11–15 year olds), and investigated whether, and how most effectively, school children can influence their parents’ perceptions of jaguars.

### Rationale

In the context of human–wildlife conflicts, factual information can improve perceptions of the species in question by shifting to more realistic levels the perceptions of the benefits and threats posed by the species (Marker et al. 2003; Slagle et al. 2013), and empowering people to cope with any damage caused and to find guidance on conflict mitigation. However, there have been cases in which information-based interventions aimed at improving attitudes toward predators proved ineffective, sometimes even reinforcing negative attitudes among those already holding strong views (Bruskotter and Wilson 2014). Knowledge of jaguars is poor among children (and adults alike) in rural Amazonia (Cavalcanti et al. 2010), where the jaguar is predominantly perceived as threatening. Nonetheless, jaguar attacks on people are extremely rare and have occurred almost invariably when hunted jaguars are cornered or injured, or when jaguars are defending cubs or carcasses (Hoogesteijn et al. 2016). In this study, we provided students with information (through lectures and activity books) about the jaguar (its ecological, economic and cultural importance, its impact on human livelihoods in relation to how it has been affected by human actions such as deforestation and persecution, and how to prevent livestock depredation problems). We hypothesized that by providing such information we would significantly increase students’ knowledge of the species, improve their perceptions of the impact of jaguars on their livelihoods, and improve their attitudes towards them.

Passive involvement in education, however, generally leads to limited retention of information, and to learn well, students need to be actively engaged during a lesson, e.g. writing, discussing, or solving problems (Bonwell and Eison 1991; Fadel et al. 2015). The more elaborate mental processing associated with active learning makes novel information both easier to remember and more personally meaningful (Kane 2004). In a similar vein, Petty and Cacioppo (1996) propose that attitude change depends upon the amount of thoughtful consideration (cognitive elaboration) that occurs in response to a persuasive communication. Because jaguars have arguably a stronger presence in the hearts and minds of Brazil’s children and adolescents than does any other native mammal, generally eliciting strong, but mixed, feelings, we expected that children would be motivated to engage in a discussion about jaguar issues and to process cognitively the related information. Lack of knowledge about jaguars, however, could hinder their ability to elaborate. We hypothesized, then, that elaboration would cause a greater impact on students who had been provided with information about the species.

Jaguars are generally killed by adult male landowners and ranch employees. Therefore, any school-based approach to jaguar conservation will be effective in the short term only if students can influence behaviors among their fathers or other men in the community. Men in rural Brazilian Amazon and likely elsewhere in the jaguar’s range tend to have strong feelings towards the species (Marchini and Macdonald 2018), which might increase interest and consequently enhance intergenerational learning about jaguar-related issues. We predicted that students participating in a jaguar education program at school would transfer to their fathers knowledge gained from lectures and educational materials and, in so doing, cause a change in their fathers’ attitudes to jaguars. Another way schools might contribute to conservation is by acting as a conduit for the distribution of communication materials. Communication effectiveness depends on the credibility and trustworthiness of the information source (Petty and Cacioppo 1996). Community institutions such as schools, cooperatives and church are arguably more credible and trustworthy to rural Amazonians than are outside institutions. As a result they can act as role models by demonstrating attitudes and behaviors that the community can readily identify with and imitate (Bandura 1997; Wright et al. 2015). We predicted that communication material—more specifically an illustrated book on jaguar ecology and predation problems—would be more effective in changing landowners’ attitudes and mainly social norms about jaguars if it reached landowners through the local school, with clear endorsement by the school, rather than through a non-governmental conservation organization.

## Materials and methods

### Experimental design

**Fig. 1:**
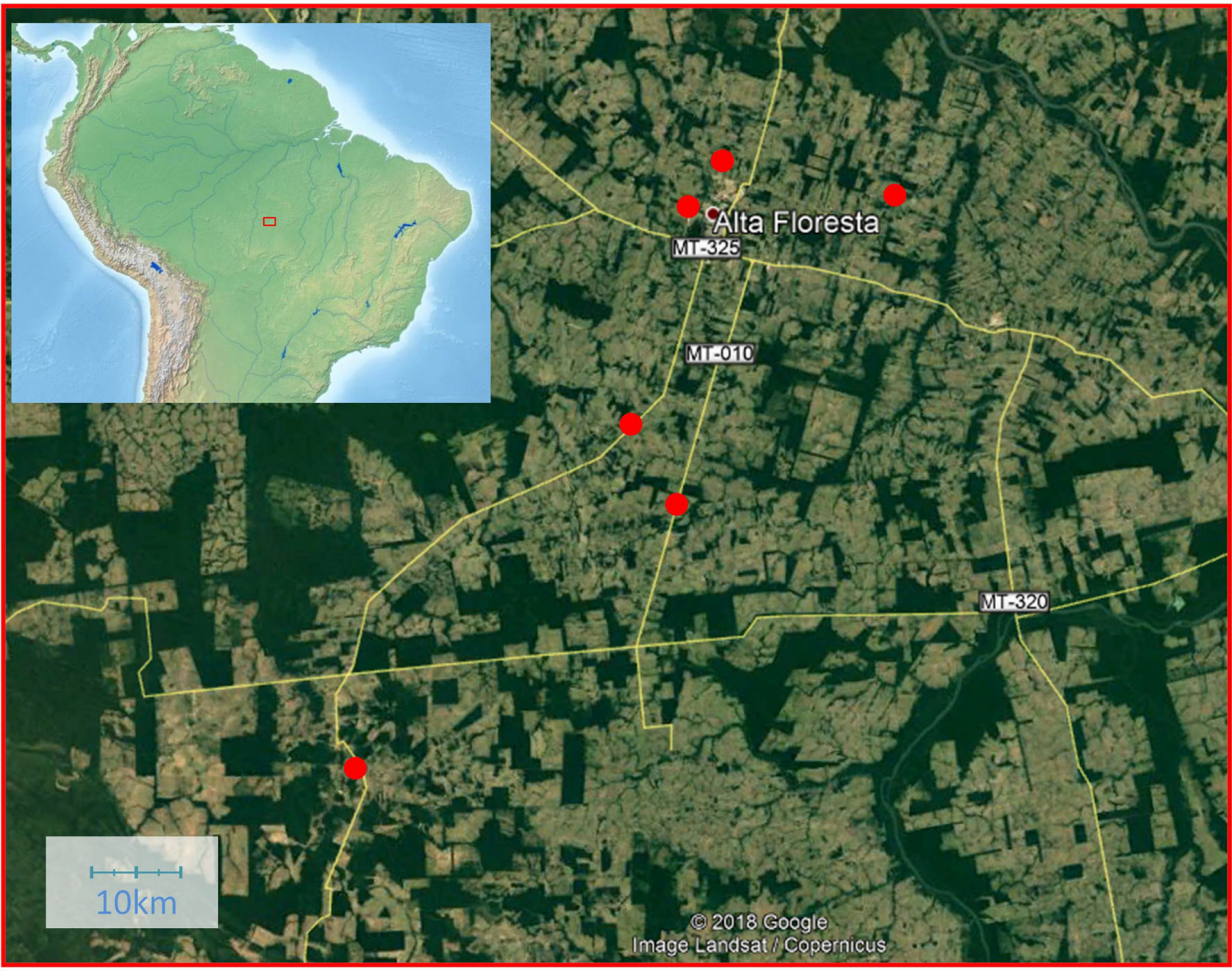
Location of the study region in Alta Floresta, southern Amazonia, Brazil. The six schools (red dots) are shown

This study was conducted between July and November 2010 in Alta Floresta, on the deforestation frontier in southern Amazonia, Brazil (Fig. 1), where cattle depredation by jaguars is considered a problem and persecution is a major threat to jaguars (Marchini and Macdonald 2012, 2018). Alta Floresta was founded in 1976 and colonized by migrant farmers, mostly from southern Brazil. Today, its economy is based primarily on cattle ranching, besides timber extraction and agriculture (Brazilian Institute of Geography and Statistics 2016), and all the adult men in this study raised cattle. The work took place at six rural public schools and was divided into two parts: (A) influencing students directly via information and elaboration interventions; and (B) influencing fathers indirectly via school-based communication interventions with their children. Informed consent was obtained from all schools’ directors and individual adult participants in the study. All procedures performed in this study were in accordance with the ethical standards of the institutional research committee.

**Fig. 2:**
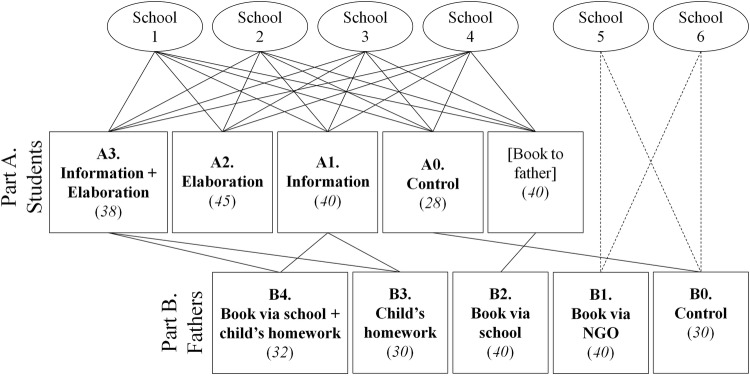
Diagram showing assignment of students and fathers to experimental treatments (and sample sizes). Dashed lines indicate that fathers were not aware of the involvement of their children’s school in the study

**Fig. 3:**
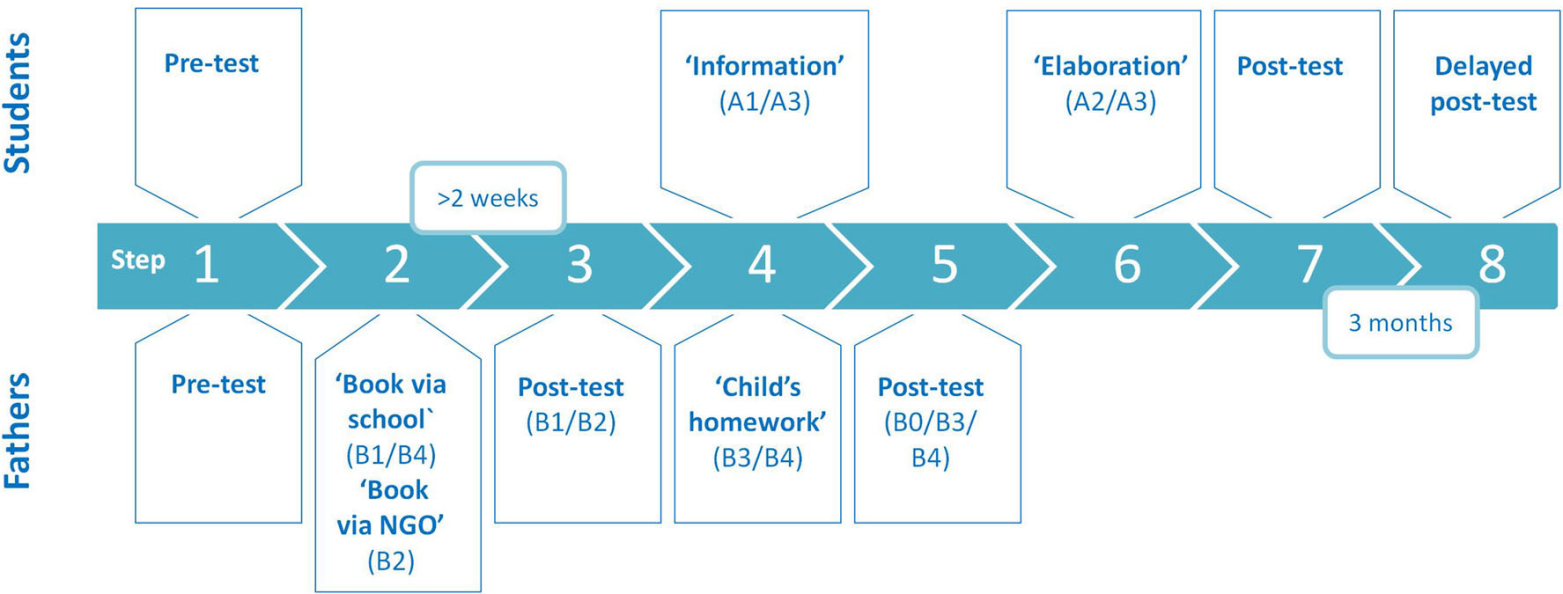
Timeline of tests and treatments

At four of the six schools, one-fifth of all students in the 5th and 6th grades (ages 11 to 14) were randomly assigned to one of the following treatments of Part A: (A0) control; (A1) information; (A2) elaboration; and (A3) information plus elaboration (Fig. 2). The remaining fifth of students were assigned to Part B (treatment B2, ‘book via school’). Students in the group A0 (‘control’) were not exposed to any lecture or group discussion, and continued their education as usual. Treatment A1 (‘information’) consisted of three 90-min lectures (one per week over 3 weeks), about jaguar issues. Lectures were based on two activity books (Appendices S1 and S2) and covered topics such as jaguar ecology, impact of jaguars on human livelihoods, jaguar conservation status, reasons to conserve jaguars, and measures to minimize the impact of jaguars on livestock. Lectures and activity books focused on factual knowledge and did not attempt to convey ideas on whether certain attitudes or behaviors towards jaguars would be morally right or wrong. For each lecture, some sections of the activity books were assigned as homework. The lectures were given by a local teacher (who had taught previously at one of the participating schools) under the supervision of the researcher. Treatment A2 (‘elaboration’) consisted of a structured group discussion in which students raised, shared and debated all their beliefs and perceptions relating to jaguars (Appendix S3). The discussion was approximately 2 h long and was moderated by the researcher with the assistance of the same local teacher that taught the lectures for the information treatment. The researcher and his assistant were as objective and neutral in appearance and behavior as possible and did not provide students with any factual or judgmental information about jaguars during the discussion. Students in the group A2 did not use activity books, and were not assigned homework. Students in the group A3 (‘information plus elaboration’) were exposed to the same lectures as group A1, and were given their third lecture 1 week before taking part in the elaboration exercise (Fig. 3).

Except for the fathers in the group B0 (‘control’), who were not exposed to any information, the adults received information about jaguars from one or both of two sources: the book *People and Jaguars: a Guide for Coexistence* (Marchini and Luciano 2010; Appendix S4), and their children’s homework. *A Guide for Coexistence* is an easy-to-read, highly illustrated color book. It is divided into the following sections: ‘Jaguars; what they are and how they live’, ‘Jaguars: are they a problem for us?’, ‘People and jaguars: when we are the problem’, ‘Reasons to coexist with jaguars’, ‘How to coexist with jaguars’ and ‘Beyond coexistence: learning more about and enjoying jaguars’. As with the activity books, the guide focuses on factual information and does not convey opinions about what may be morally right or wrong in the relationships between people and jaguars. In treatment B1 (‘book via NGO’), fathers randomly selected from a list of fathers of 5th and 6th graders at two other schools received the book from the researcher or his assistant. The researcher and his assistant visited each of these fathers at home, introduced themselves as representatives of a local non-governmental conservation organization (Cristalino Ecological Foundation, CEF), interviewed the father, and handed him one copy of the book on behalf of CEF. These fathers were not informed about the involvement of their children’s school in providing parents’ contact details for the study. In treatment B2 (‘book via school’), another group of fathers received the book directly from their children, together with an endorsement letter from the school explaining that the book was part of broader jaguar education program. Treatment B3 (‘child’s homework’) consisted of being asked to sign their children’s homework, and treatment B4 (‘book via school + child’s homework’) consisted of both receiving the book from their children and signing their homework. Fathers in B3 and B4 received a letter from the school’s director explaining that the school was implementing a jaguar education program directed at students, parents and community members.

### Measurements

We conducted a pilot study during which three focus groups involving 36 students altogether and qualitative interviews with 90 adults were conducted. By listening to people talk freely, we were able to identify salient beliefs, perceptions, and peculiarities of the local parlance, which were then used in the design of the questionnaire, and in adjusting our language to the target groups. During the piloting process, open-ended questions were replaced incrementally by clear, quantitative questions that would produce data suitable for statistical analysis (Newing 2011). Students were then evaluated three times: before and immediately after exposure to the treatment (pre-test and post-test, respectively) and again 3 months later (delayed post-test) to test for retention of treatment effects (Fig. 3). Fathers were evaluated using a pre-test and a post-test only. Students were evaluated in class by the means of self-completion questionnaires. The researcher introduced himself and his assistant as representatives of an educational project from outside the school and gave the students a brief introduction. The introduction was devoid of value statements and merely invited them to participate in a project involving questions about their opinions about wildlife. The researcher stressed that there were no right or wrong answers, that no grade would be assigned, and therefore, students could feel at ease about offering their views transparently. The researcher also emphasized the importance of each student completing their questionnaires individually. Teachers were given the choice of staying or leaving the room; a few stayed but did not interfere with the survey. Questionnaires and pencils were distributed and questions were read out one at a time. We checked that students understood each question and numbered their responses correctly. This approach allowed us to repeat questions and provide further explanations where necessary. Questionnaires were completed in approximately 30 min and returned directly to the researcher.

Fathers were evaluated at home using face-to-face interviews (Newing 2010) conducted by the researcher. The researcher was always accompanied by a local field assistant during the interviews. The researcher and his assistant were as objective and neutral in appearance and behavior as possible. The researcher introduced himself to the fathers of students in schools 1 to 4 as a representative of an educational project, rather than a conservation organization. The researcher introduced himself to the fathers contacted through schools 5 and 6 as a representative of CEF. He explained to respondents that the purpose of the survey was to collect data on people’s perceptions of wildlife, and that these would ultimately contribute to a conservation project. Separate questionnaires were designed for evaluating students and fathers but both types examined the following: (1) knowledge about jaguars and depredation problems; (2) attitude towards jaguars; (3) perception of the impact of jaguars on human safety; (4) perception of the impact of jaguars on livestock; (5) attitudes towards killing jaguars; (6) descriptive norm regarding the killing of jaguars. Respondents knowledge was recorded on a dichotomous scale (1 for correct, 0 for otherwise), perceptions of jaguar impact on a 6-point scale coded 0–5 (no impact to high impact), attitudes on a 5-point Likert scale ranging from − 2 (very unfavorable) to 2 (very favorable), and descriptive norm on a 5-point scale from 0 (minimum) to 4 (maximum) (Table 1). We created average scales to summarize each of these components and used Cronbach’s alpha to improve the internal coherence of the scales by discarding items to maximize the alpha value (Vaske 2008). The effect of these variables on the intention to kill jaguars among landowners in southern Amazonia has been examined elsewhere (Marchini and Macdonald 2012).

**Table 1:** Variables addressed, survey questions and response categories used to assess them, and their reliability (Cronbach’s alpha; *α*) (Vaske 2008)

Variable/survey question/range	Response categories	α
*Knowledge about jaguars and depredation problems (0 to 10)*	0 = incorrect and do not know 1 = correct	0.77
The jaguar generally begins to consume its prey from the front while the puma consumes the areas from the ribs backwards (correct)		
A jaguar’s prey is usually hidden and covered with leaves, while a puma’s prey isn’t (incorrect)		
The jaguar’s prey generally presents a bite mark at the base of the neck, while the puma’s prey generally has a bite on the throat (correct)		
The jaguar’s footprint is longer than wide with thinner and pointed toes, while the puma’s footprint is slightly wider than long, with round toes (incorrect)		
A female jaguar produces on average 1 or 2 cubs every other year (correct)		
Black jaguars are far more dangerous to cattle than yellow jaguars (incorrect)		
The heaviest jaguar ever captured weighed approximately 150 kilos (correct);		
Jaguars kill more people every year in Brazil than do domestic dogs (incorrect);		
Where cattle are more abundant than native prey, jaguars take more cattle than native prey (correct)		
Calves kept closer to the forest edge have in general a chance of being killed by jaguars (incorrect)		
*Perception of jaguar impact on livestock (0 to 20)*	0 = None 1 = Very small 2 = Small 3 = Medium 4 = High 5 = Very high	0.83
How would you rate the damage associated with predation ever caused by jaguars to your father		
How would you rate the risk of any damage associated with predation to your father in the next 12 months (students only)		
How would you rate the damage associated with predation ever caused by jaguars to you		
How would you rate the risk of any damage associated with predation to you in the next 12 months (fathers only)		
*Perception of the impact of jaguars on human safety (0 to 10)*		0.91
Number of people ever hurt by a jaguar in the neighborhood		
How would you rate the risk of you being hurt by a jaguar in the next 12 months		
*Attitude to jaguars (− 10 to 10)*		0.89
You would like the jaguar population in the region to:	− 2 = decrease a lot, − 1 = decrease, 0 = stay the same, 1 = increase, 2 = increase a lot	
If all the jaguars disappeared forever from the region, you would feel:	− 2 = very happy, to 2 = very sad	
What you feel towards jaguars is better described as:	− 2 = dislike a lot, to 2 = like a lot	
The jaguar has its value, even if it does not generate any income to you:	− 2 = strongly disagree, to 2 = strongly agree	
If you had to walk on your own in a forest where there are jaguars, you would feel scared:		
*Attitude to killing jaguars (− 2 to 2)*		0.87
Killing any jaguar that appears in my property is…	− 2 = very useful, to 2 = very useless	
	− 2 = very exciting, to 2 = very boring	
*Descriptive norm (0 to 8)*		0.80
How many of your neighbours do you think kill jaguars?	0 = none of them 1 = less than half of them 2 = about half them 3 = more than half of them 4 = all of them	
Think of the landowners in Alta Floresta—what percentage of them do you think kill jaguars?		

### Data analysis

All analyses were conducted in SPSS, version 14. We used paired sample t tests to compare the six composite measures—knowledge about jaguars and predation problems, attitude towards jaguars, perception of the impact of jaguars on human safety, perception of the impact of jaguars on livestock, attitudes towards killing jaguars and descriptive norm regarding the killing of jaguars—between pre-test, post-test and delayed post-test. General linear model (GLM) was used to examine possible interactions between the explanatory variables (treatment by time) in determining attitudes to jaguars and to jaguar killing.

## Results

A total of 151 students (68 females and 83 males) completed both pre- and post-tests, 145 of which also completed the delayed post-test, and 172 fathers were surveyed. Mean pupil age was 12.63 (SD = 1.18, range = 11–18) and mean father age was 46.38 (SD = 5.89, range = 36–63).

### Information versus elaboration

**Fig. 4:**
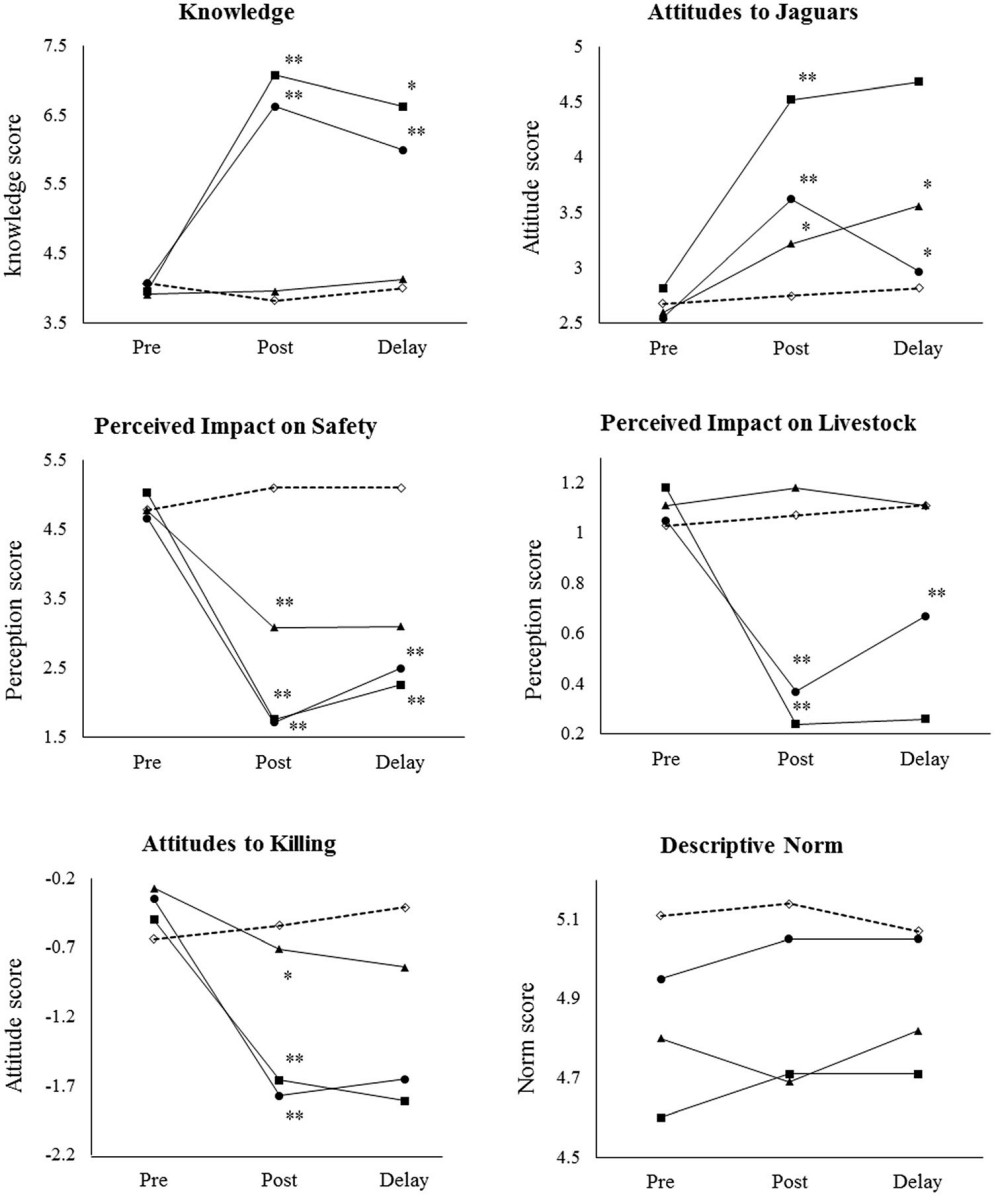
Variation in students’ perceptions of jaguars between pre-test, post-test and delayed post-test in response to the following treatments: A0 control (open diamond), A1 information (filled circle), A2 elaboration (filled triangle), and A3 information plus elaboration (filled square). *p < 0.05; **p < 0.01

Both passively received information (B1/B3) and active elaboration (B2/B3) had significant effects on students (Table 2 and Fig. 4). The comparison between pre- and post-tests showed that information alone significantly improved knowledge about jaguars, improved attitudes towards them, reduced the perceptions of their impacts on both human safety and livestock, and made people less favorable towards jaguar killing. Only descriptive norm was not affected by information. Elaboration alone changed attitudes to jaguars, perceived impact on human safety and attitudes to jaguar killing. Combined, information and elaboration significantly changed knowledge, attitudes to jaguars, perceived impact on human safety and livestock and attitudes to killing. However, the delayed post-test revealed that some of these effects were not completely retained. Comparisons between post and delayed post-tests showed that knowledge, attitudes to jaguars and perceived impacts on human safety and livestock among students who had been exposed to information alone, and knowledge and perceived impact on human safety among students who had been exposed to information and elaboration combined, changed in the opposite direction than in the pre–post-test comparison (nonetheless, differences between pre-test and delayed post-test were still significant). The effect of elaboration alone on perceived impact on safety and attitudes to killing was retained and for attitudes to jaguars it significantly increased between post and delayed tests.

**Table 2:** Mean scores ± standard deviations for, and paired *t* tests between, pre-test, post-test and delayed post-test on students’ perceptions of jaguars

Treatment	Measure	Pre	Post	Pre-post			Delayed	Post-delayed			Pre-delayed		
				*t*	df	*p*		*t*	df	*p*	*t*	df	*p*
*A0* Control	Knowledge	4.07 ± 1.12	3.82 ± 1.06	1.42	27	0.165	4.00 ± 1.19	− 0.895	27	0.379	0.348	24	0.731
	Attitudes to jaguars	2.68 ± 2.31	2.75 ± 2.06	− 0.348	27	0.731	2.82 ± 1.96	− 0.372	27	0.713	− 0.583	24	0.565
	Perception of impact on safety	4.79 ± 1.68	5.11 ± 1.42	− 1.880	27	0.071	5.11 ± 1.22	0.000	27	1.000	− 1.560	24	0.130
	Perception of impact on livestock	1.03 ± 0.88	1.07 ± 0.85	− 0.297	27	0.769	1.11 ± 0.83	− 0.273	27	0.787	− 0.626	24	0.537
	Attitudes to killing	− 0.64 ± 1.97	− 0.54 ± 1.86	− 1.000	27	0.326	− 0.41 ± 1.84	1.544	27	0.134	0.528	24	0.602
	Descriptive norm	5.11 ± 1.31	5.14 ± 1.43	− 0.328	27	0.745	5.07 ± 1.33	− 0.626	27	0.537	0.570	24	0.573
*A1* Information	Knowledge	4.08 ± 1.38	**6.63 **± **1.19**	− **11.129**	**39**	< **0.001**	**6.00 **±** 1.24**	**4.038**	**39**	**0.000**	− **7.980**	**38**	< **0.001**
	Attitudes	2.55 ± 2.49	**3.62 **± **2.15**	− **4.611**	**39**	< **0.001**	**2.97 **± **2.57**	**2.579**	**39**	**0.014**	− **3.597**	**38**	**0.001**
	Perception of impact on safety	4.67 ± 1.84	**1.72 **± **1.34**	**15.312**	**39**	< **0.001**	**2.50 **± **1.80**	− **3.740**	**39**	**0.001**	**9.618**	**38**	< **0.001**
	Perception of impact on livestock	1.05 ± 1.21	**0.37 **± **0.92**	**4.970**	**39**	< **0.001**	**0.67 **± **0.92**	− **2.762**	**39**	**0.009**	**2.564**	**38**	**0.014**
	Attitudes to killing	− 0.35 ± 1.96	− **1.77 **± **1.74**	**8.924**	**39**	< **0.001**	− 1.65 ± 1.76	− 1.403	39	0.168	**9.313**	**38**	< **0.001**
	Descriptive norm	4.95 ± 1.93	5.05 ± 1.62	− 1.071	39	0.291	5.05 ± 1.83	0.255	39	0.800	− 1.356	38	0.183
*A2* Elaboration	Knowledge	3.91 ± 1.04	3.96 ± 1.11	− 0.298	44	0.767	4.13 ± 1.16	− 1.034	44	0.307	− 1.279	42	0.208
	Attitudes	2.60 ± 2.05	**3.22 **± **3.67**	− **2.213**	**44**	**0.032**	**3.56 **± **3.53**	− **2.236**	**44**	**0.030**	− **3.473**	**42**	**0.001**
	Perception of impact on safety	4.78 ± 1.63	**3.09 **± **3.07**	**5.703**	**44**	< **0.001**	3.11 ± 3.24	− 0.172	44	0.864	**5.093**	**42**	< **0.001**
	Perception of impact on livestock	1.11 ± 1.19	1.18 ± 1.28	− 0.684	44	0.497	1.11 ± 1.17	0.596	44	0.554	0.000	42	1.000
	Attitudes to killing	− 0.27 ± 1.65	− **0.71 **± **2.31**	**2.379**	**44**	**0.022**	− 0.84 ± 2.35	1.633	44	0.110	**2.857**	**42**	**0.007**
	Descriptive norm	4.80 ± 1.87	4.69 ± 1.86	1.530	44	0.133	4.82 ± 2.02	− 1.354	44	0.183	− .330	42	0.743
*A3* Information + elaboration	Knowledge	3.95 ± 1.27	**7.08 **± **1.38**	− **12.493**	**37**	< **0.001**	**6.63 **± **1.42**	**2.340**	**37**	**0.025**	− **8.889**	**37**	< **0.001**
	Attitudes	2.82 ± 2.29	**4.52 **± **4.00**	− **4.865**	**37**	< **0.001**	4.68 ± 3.76	− 1.356	37	0.183	− **5.676**	**37**	< **0.001**
	Perception of impact on safety	5.03 ± 1.55	**1.76 **± **1.44**	**15.619**	**37**	< **0.001**	2.26 ± 1.97	− **3.452**	**37**	**0.001**	**10.991**	**37**	< **0.001**
	Perception of impact on livestock	1.18 ± 1.13	**0.24 **± **0.63**	**7.267**	**37**	< **0.001**	0.26 ± 0.53	− 0.329	37	0.744	**6.034**	**37**	< **0.001**
	Attitudes to killing	− 0.50 ± 1.75	− **1.66 **± **1.88**	**7.333**	**37**	< **0.001**	− 1.81 ± 1.95	1.639	37	0.110	**6.963**	**37**	< **0.001**
	Descriptive norm	4.60 ± 2.04	4.71 ± 2.15	− 0.813	37	0.422	4.71 ± 2.05	0.000	37	1.000	− 1.434	37	0.160

Bold numbers indicate differences between scores are statistically significant at the 0.05 level

The direction of the effect of elaboration on attitudes to jaguars and attitudes to jaguar killing depended on students’ initial attitudes. The interaction between attitudes to jaguars and test (pre versus post) was significant for elaboration alone (GLM: F = 17.236, df = 1, *p* < 0.001) and elaboration and information combined (GLM: *F* = 19.738, df = 1, *p* < 0.001). The interaction between attitudes to jaguar killing and test was significant for elaboration alone (GLM: *F* = 4.190, df = 1, *p* = 0.047), but not for elaboration and information combined (GLM: *F* = 0.780, df = 1, *p* = 0.383). As described in Table 3, among students who had positive attitudes towards jaguars in the pre-test, attitudes became significantly more positive after elaboration alone, or information and elaboration combined. Among students who had neutral or negative attitudes towards jaguars in the pre-test, attitudes became more negative after exposure to elaboration alone, or elaboration and information, although this latter change was not significant. Likewise, in students who had negative attitudes to jaguar killing in the pre-test, attitudes became significantly more negative after elaboration alone, or information and elaboration combined. However, among students who had positive attitudes to jaguar killing in the pre-test, attitudes did not change after exposure to elaboration alone, and became less positive after exposure to information and elaboration combined.

**Table 3:** Mean scores ± standard deviation for, and paired *t* tests between, pre-test and post-test on students’ attitudes to jaguars and jaguar killing. Initial attitude: ‘positive’ for scores above 0, ‘neutral/negative’ for scores 0 or below

Treatment	Target of attitude	Initial attitude	Pre	Post	*t*	df	*p*
*A2* Elaboration	Jaguar	Positive	3.15 ± 1.27	4.12 ± 2.62	− 3.723	39	0.001
		Neutral/negative	− 1.80 ± 1.79	− 4.00 ± 2.74	4.491	4	0.011
	Jaguar killing	Positive	1.31 ± 0.45	1.31 ± 1.96	0.000	15	1.000
		Neutral/negative	− 1.14 ± 1.41	− 1.83 ± 1.65	3.839	28	0.001
*A3* Information + elaboration	Jaguar	Positive	3.35 ± 1.63	5.50 ± 2.83	− 6.977	33	< 0.001
		Neutral/negative	− 1.75 ± 2.06	− 3.75 ± 2.75	2.828	3	0.066
	Jaguar killing	Positive	1.27 ± 0.47	0.18 ± 1.33	3.833	10	0.003
		Neutral/negative	− 1.22 ± 1.55	− 2.40 ± 1.52	6.150	26	< 0.001

### Intergenerational learning

**Fig. 5:**
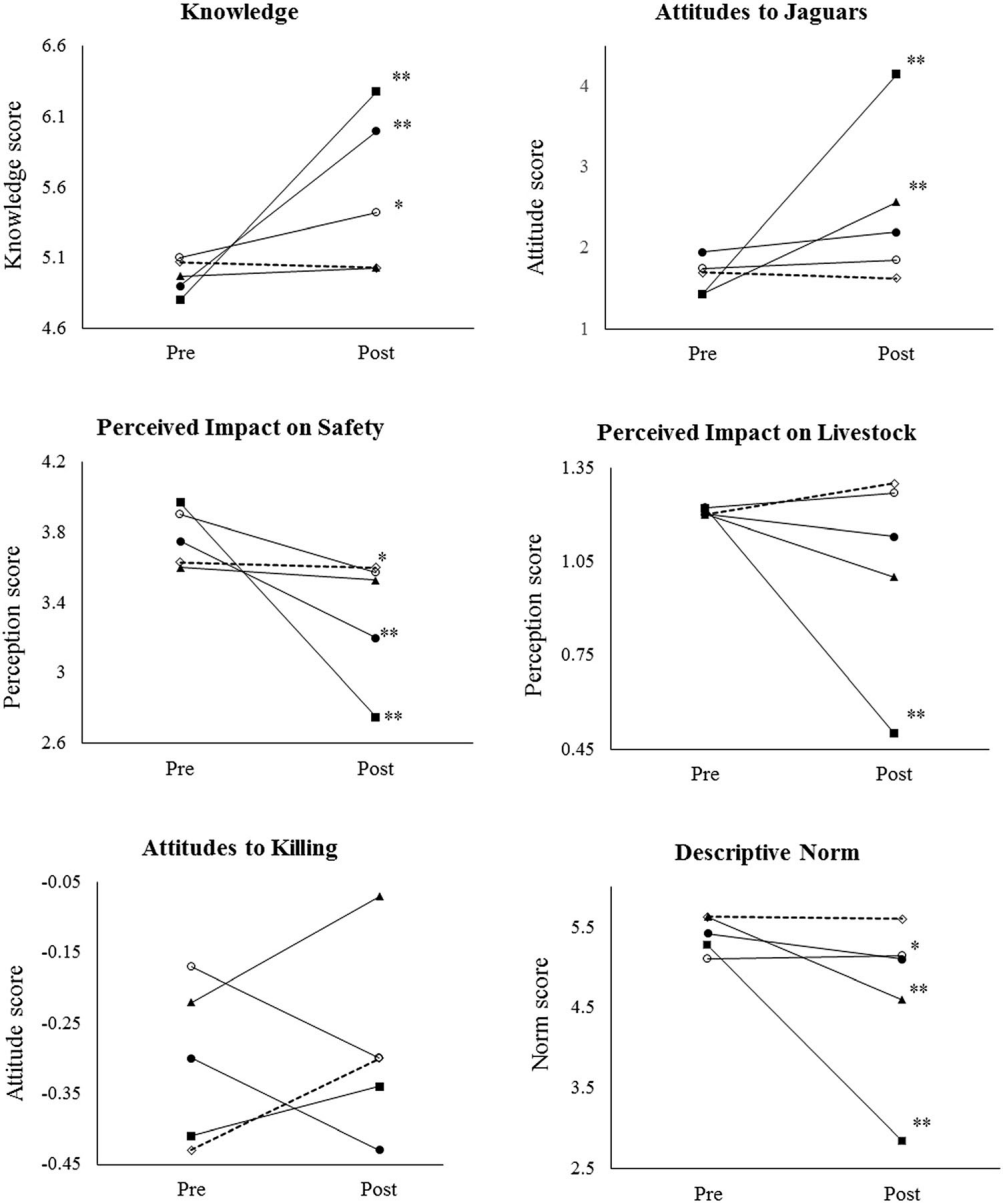
Variation in fathers’ perceptions of jaguars between pre-test and post-test in response to the following treatments: B0 control (open diamond), B1 book via conservation organization (open circle), B2 book via child’s school (filled circle), B3 child’s homework (filled triangle), and B4 book via child’s school plus child’s homework (filled square). *p < 0.05; **p < 0.01

Fathers were affected significantly both by books and by their children (Table 4 and Fig. 5). Books alone increased fathers’ knowledge and decreased their perception of the impact of jaguars on human safety, regardless of the means through which they received the book i.e. from an NGO or the local school. However, the significance of the effect was greater among those who received the book from the school (*p* < 0.001 against 0.036 for knowledge, and *p* = 0.006 against 0.026 for perception of impact on human safety). Besides, books received from the local school caused a decrease in fathers “perception of descriptive norm related to jaguar killing. Children’s homework improved their fathers” attitudes towards jaguars and decreased their perception of descriptive norm, but did not significantly affect other variables. In contrast, receiving the book from the school combined with seeing their child’s homework increased fathers’ knowledge, improved their attitudes towards jaguars, decreased their perceptions of the impact of jaguars on both human safety and livestock, and decreased their perception of descriptive norm. The only variable that remained unaffected by any intervention was fathers’ attitude towards killing jaguars.

**Table 4:** Mean scores ± standard deviation for, and paired *t* tests between, pre-test and post-test on fathers’ perceptions of jaguars

Treatment	Dependent variable	Pre	Post	*t*	df	*p*
*B0* Control	Knowledge	5.07 ± 1.65	5.03 ± 1.63	0.297	29	0.769
	Attitudes to jaguars	1.70 ± 1.93	1.63 ± 1.85	1.000	29	0.326
	Perception of impact on safety	3.63 ± 1.79	3.60 ± 1.85	0.441	29	0.662
	Perception of impact on livestock	1.20 ± 1.06	1.30 ± 1.09	− 1.140	29	0.264
	Attitudes to killing	− 0.43 ± 2.02	− 0.30 ± 1.90	− 1.439	29	0.161
	Descriptive norm	5.63 ± 1.40	5.60 ± 1.30	0.297	29	0.769
*B1* Book via conservation organization	Knowledge	5.10 ± 1.60	**5.42 **± **1.52**	− **2.177**	**39**	**0.036**
	Attitudes to jaguars	1.75 ± 1.86	1.85 ± 1.76	− 1.433	39	0.160
	Perception of impact on safety	3.90 ± 1.76	**3.57 **± **1.79**	**2.314**	**39**	**0.026**
	Perception of impact on livestock	1.22 ± 1.05	1.27 ± 1.11	− 0.628	39	0.534
	Attitudes to killing	− 0.17 ± 1.93	− 0.30 ± 1.71	1.152	39	0.256
	Descriptive norm	5.10 ± 1.90	5.15 ± 1.80	− 0.495	39	0.623
*B2* Book via school	Knowledge	4.90 ± 1.51	**6.00 **± **1.69**	− **5.284**	**39**	< **0.001**
	Attitudes to jaguars	1.95 ± 2.26	2.20 ± 2.14	− 1.818	39	0.077
	Perception of impact on safety	3.75 ± 1.64	**3.20 **± **1.56**	**2.905**	**39**	**0.006**
	Perception of impact on livestock	1.20 ± 0,92	1.00 ± 0.93	1.433	39	0.160
	Attitudes to killing	− 0.22 ± 1.69	− 0.07 ± 1.59	− 1.964	39	0.057
	Descriptive norm	5.42 ± 1.48	**5.10 **± **1.82**	**2.061**	**39**	**0.046**
*B3* Child’s homework	Knowledge	4.97 ± 1.24	5.03 ± 1.24	− 0.701	29	0.489
	Attitudes to jaguars	1.43 ± 4.85	**2.57 **± **4.14**	− **4.753**	**29**	< **0.001**
	Perception of impact on safety	3.60 ± 2.08	3.53 ± 1.74	0.360	29	0.722
	Perception of impact on livestock	1.20 ± 0.66	1.13 ± 0.94	0.626	29	0.536
	Attitudes to killing	− 0.30 ± 2.19	− 0.43 ± 2.06	1.000	29	0.326
	Descriptive norm	5.63 ± 1.35	**4.60 **± **1.40**	**3.903**	**29**	**0.001**
*B4* Book via school + child’s homework	Knowledge	4.8 ± 11.73	**6.28 **± **2.05**	− **6.679**	**31**	< **0.001**
	Attitudes to jaguars	1.43 ± 3.20	**4.15 **± **2.54**	− **8.718**	**31**	< **0.001**
	Perception of impact on safety	3.97 ± 1.38	**2.75 **± **1.52**	**4.978**	**31**	< **0.001**
	Perception of impact on livestock	1.22 ± 0.83	**0.50 **± **0.76**	**5.578**	**31**	< **0.001**
	Attitudes to killing	− 0.41 ± 1.56	− 0.34 ± 1.37	− 0.571	31	0.572
	Descriptive norm	5.28 ± 1.90	**2.84 **± **1.87**	**7.012**	**31**	< **0.001**

Bold numbers indicate differences between scores are statistically significant at the 0.05 level

## Discussion

This study shows that school-based education and communication interventions can have a powerful impact on student’s perceptions of jaguars, and those of their fathers; this process could be used to positive conservation effect. The finding that students can influence their fathers’ perceptions of jaguars suggests that conservationists can use rural schools to reach at once tens of students in a classroom, or hundreds on the school’s soccer pitch, who will in turn transfer the conservation message to their fathers. Given the logistical challenge of visiting landowners one-by-one at home in rural Amazonia, this strategy might be relatively cost-effective. The exceptionally strong prominence of the jaguar in people’s hearts and minds (Marchini and Luciano 2010), combined with the relatively high rate of primary school enrolment in Brazil (97.7%) (Brazilian Institute of Geography and Statistics 2016) and the willingness of public school directors and teachers to cooperate with conservationists, renders school-based intergenerational learning a particularly promising approach for jaguar conservation.

Attitudes and descriptive norms related to jaguar killing are the most immediate determinants of jaguar killing behavior among farmers and ranchers in Amazonia (Marchini and Macdonald 2012). Therefore, a relevant effect detected among fathers in this study was the decrease in their perceptions of how common the killing of jaguars is among their neighbors. Conservationists have used education and communication to increase knowledge and improve attitudes among target stakeholders, indirectly influencing the change in stakeholders’ behaviors for the benefit of conservation (Jacobson et al. 2006). However, attempts to influence social norms regarding conservation-orientated behaviors have been far less considered (but see Chaves et al. 2018). We found that books distributed via local schools changed descriptive norms among fathers, while [the same] books distributed via a conservation organization did not. This result suggests that parents’ perceptions can be influenced not only by the information explicitly conveyed in the content of books and their children’s homework, but also by the implicit message that a community institution (and therefore other community members) supports jaguar conservation more than they had realized. The use of role models, case studies, and examples of coexistence with jaguars, could conceivably enhance the power of school-based communication campaigns to create or redefine social norms concerning conservation-orientated behaviors (Monroe 2003). The ineffectiveness of the book distributed via a conservation organization may result from a lack of trust on environmental agencies, which has been shown to determine intolerance to wildlife in general (Bruskotter and Wilson 2014) and to big cats in the Brazilian Atlantic Forest, in particular (Engel et al. 2017).

This study also demonstrated the potentially detrimental effect of prejudiced communication compounding negative attitudes towards species involved in human–wildlife conflict. Students’ attitudes towards jaguars—both positive and negative—were reinforced by the elaboration exercise; even the negative attitudes held by a few students, towards jaguars, were strengthened. Possible explanations for this polarization of attitudes are cognitive bias, and reactance. Students may have learned selectively (i.e. confirmation bias) the new information raised and shared by the group, about the benefits and drawbacks of coexisting with jaguars, to support their schemas concerning the species, i.e. pre-existing biases that provide a framework or structure for their beliefs regarding jaguars (Brewer and Treyens 1981). Attitudes can also become more extreme or polarized as a result of reactance. During the elaboration intervention, students were encouraged to share their opinions with the rest of the group without knowing that they would subsequently have to justify their views. Only a few students in each group expressed negative opinions about jaguars and their views were challenged by the majority of their class mates. By defending their opinions under peer pressure, those students became more convinced that they were right or developed even more extreme views, a phenomenon known as the boomerang effect or reactance (Brehm 1966). Reactance can occur in anyone who believes that his or her freedom to choose freely how to think, feel, or act may be or has been limited. Conservationists should become acquainted with stakeholders’ views and attitudes, and consider carefully the implications of exposing or challenging them, for fear of causing an unintended, perverse strengthening of a misplaced view. Failure to consider reactance can cause communication interventions to backfire, producing the opposite effect to that intended.. When reactance is likely to be an issue, communication campaigns would arguably be more effective if they rely on descriptive norms (involving perceptions of which behaviors are typically performed) rather than injunctive norms (involving perceptions of which behaviors are typically disapproved of) (Cialdini 2003), and strive to promote positive conduct (with prescriptive messages) rather than demote negative conduct (with proscriptive messages) (Winter et al. 2000).

Information alone significantly improved knowledge, attitudes towards jaguars, and perceptions of jaguar impacts on human safety and livestock among students and fathers. However, some of these changes did not endure. Because knowledge of jaguars among children and adults in rural Amazonia is typically poor, and most people in the region over-estimate the real impact of jaguars on human livelihoods (Cavalcanti et al. 2010), information-based interventions (e.g. lectures and books) were indeed expected to improve knowledge and perceptions regarding the species. Although such interventions did improve students’ knowledge, attitudes and perceptions in post-tests conducted immediately afterwards, these effects (while still significantly different) were not sustained at the same level 3 months later. Information and elaboration together had a stronger effect than information alone on knowledge, attitudes towards jaguars and perceived impact on livestock. As expected, these changes were more enduring than those produced by knowledge alone. Information may have moderated the effect of elaboration on students’ attitudes towards killing jaguars: while positive attitudes towards killing remained unchanged after elaboration alone, they decreased significantly when elaboration was preceded by information. In contrast, however, the same moderating effect of information was not observed on students’ negative attitudes towards jaguars per se. This finding suggests that attitudes towards jaguars as a species are less rational, and may be more entrenched, than attitudes towards killing them. While attitudes towards killing jaguars may be manipulated using information-based interventions, education and communication approaches aimed at ameliorating strongly negative attitudes towards jaguars themselves may be more effective if they capitalize particularly on the strong, mixed emotions elicited by the species.

## Conclusion

This work reveals a path in the direction of reducing jaguar killing in Amazonia via children’s influence on their parents and has practical implications for reducing persecution of other charismatic species elsewhere. While the efforts to increase people’s tolerance of wildlife and discourage persecution continue to focus largely on economic incentives and legal prohibitions and sanctions, communication strategies providing information and social support can also be effective at changing behavior, and deserve more attention from conservationists and policy-makers. With proper collaboration across organizations and disciplines, and targeted capacity building, conservation education and communication will, in turn, benefit from systematic, rigorous approaches to monitor and evaluate success, and from evidence-based lines of action to the design of effective interventions for our coexistence with wildlife, like the one developed in this study.

## Electronic supplementary material

Below is the link to the electronic supplementary material.

Supplementary material 1 (PDF 15467 kb)
