# Supplementary material for: Can school children influence adults’ behavior toward jaguars? Evidence of intergenerational learning in education for conservation
Source: Ambio. 2019 Aug 21;49(4):912–25. doi: 10.1007/s13280-019-01230-w (PMC7028835; doi:10.1007/s13280-019-01230-w)
Supplement: Supplementary file 1 — Supplementary material 1 (PDF 15467 kb) [file 13280_2019_1230_MOESM1_ESM.pdf]

***Ambio***

Electronic Supplementary Material
*This supplementary material has not been peer reviewed*

Title: **Can school children influence adults' behavior toward jaguars? Evidence of intergenerational learning in education for conservation**

Silvio Marchini, David W. Macdonald

**Appendices S1 and S2. Activity books: ‘Jaguars: what they are and how they live’ and ‘People and jaguars: conflict and coexistence’.**

Silvio Marchini
Ricardo Luciano

LIVRO DE ATIVIDADES

# ONÇAS: O QUE SÃO E COMO VIVEM

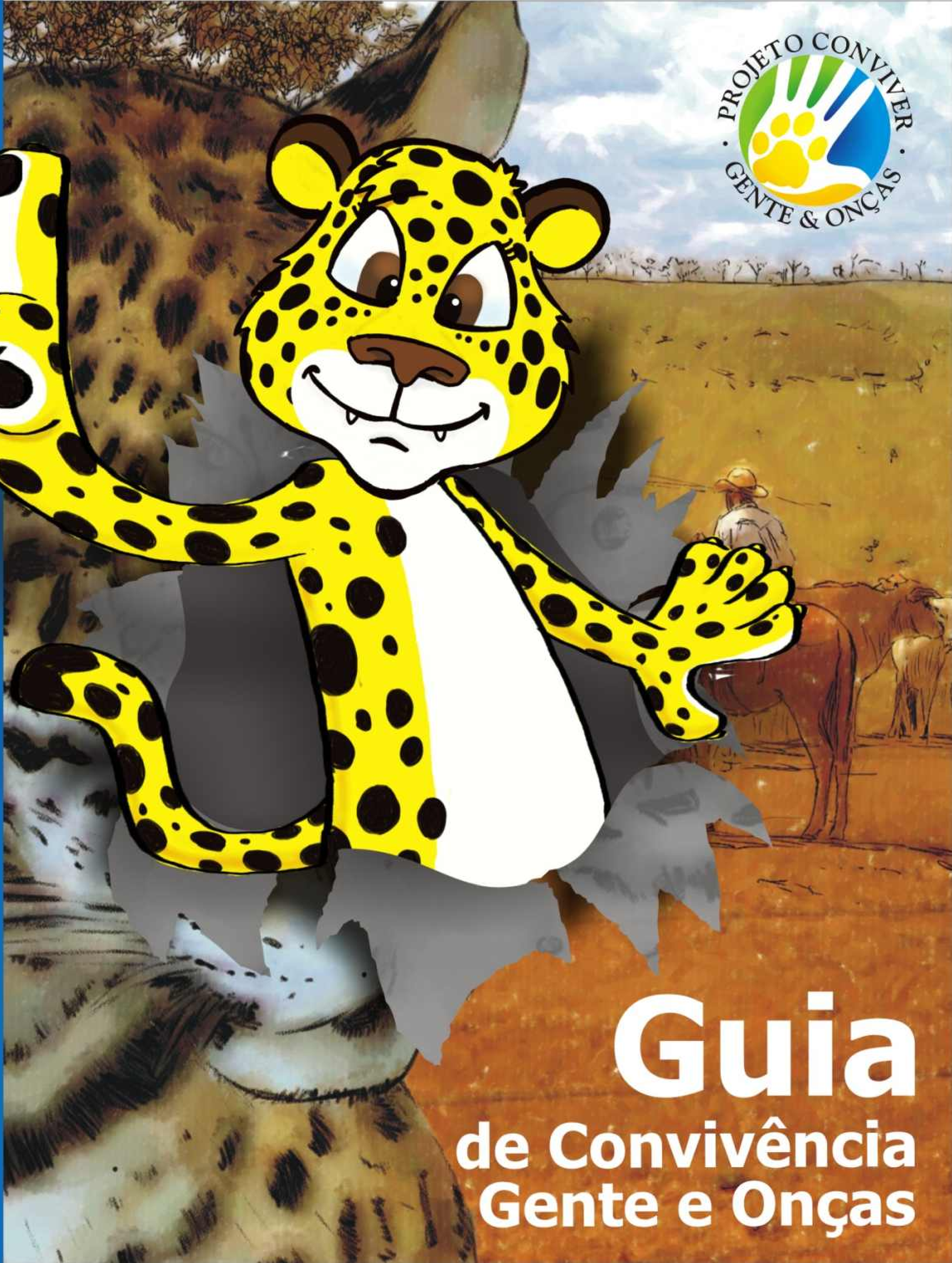

# Guia
## de Convivência Gente e Onças

PROJETO CONVIVER · GENTE & ONÇAS

Fundação
Ecológica
Cristalino

**Presidente**
Vitoria da Riva Carvalho

**Diretor Executivo**
Renato Aparecido de Farias

**ISBN:** 000-00-000000-0-0

Copyright © 2008 by Editora Amazonarium Ltda

**Autor:** © Silvio Marchini
**Ilustrador:** © Ricardo Luciano
**Páginas:** 24

Impresso no Brasil

Todos os direitos reservados. Nenhuma parte deste livro pode ser reproduzida ou utilizada por qualquer meio, sem prévia autorização por escrito da editora.

**Projeto gráfico e diagramação:**
Ricardo Luciano

COLEÇÃO GUIA DE CONVIVÊNCIA

## Livro de Atividades 1

# ONÇAS: O QUE SÃO E COMO VIVEM

Texto
**Silvio Marchini**

Ilustração
**Ricardo Luciano**

Uma realização
**Fundação Ecológica Cristalino**
**Wildlife Conservation Research Unit**

**1ª Edição**

Alta Floresta, Mato Grosso, Brasil
Maio de 2009

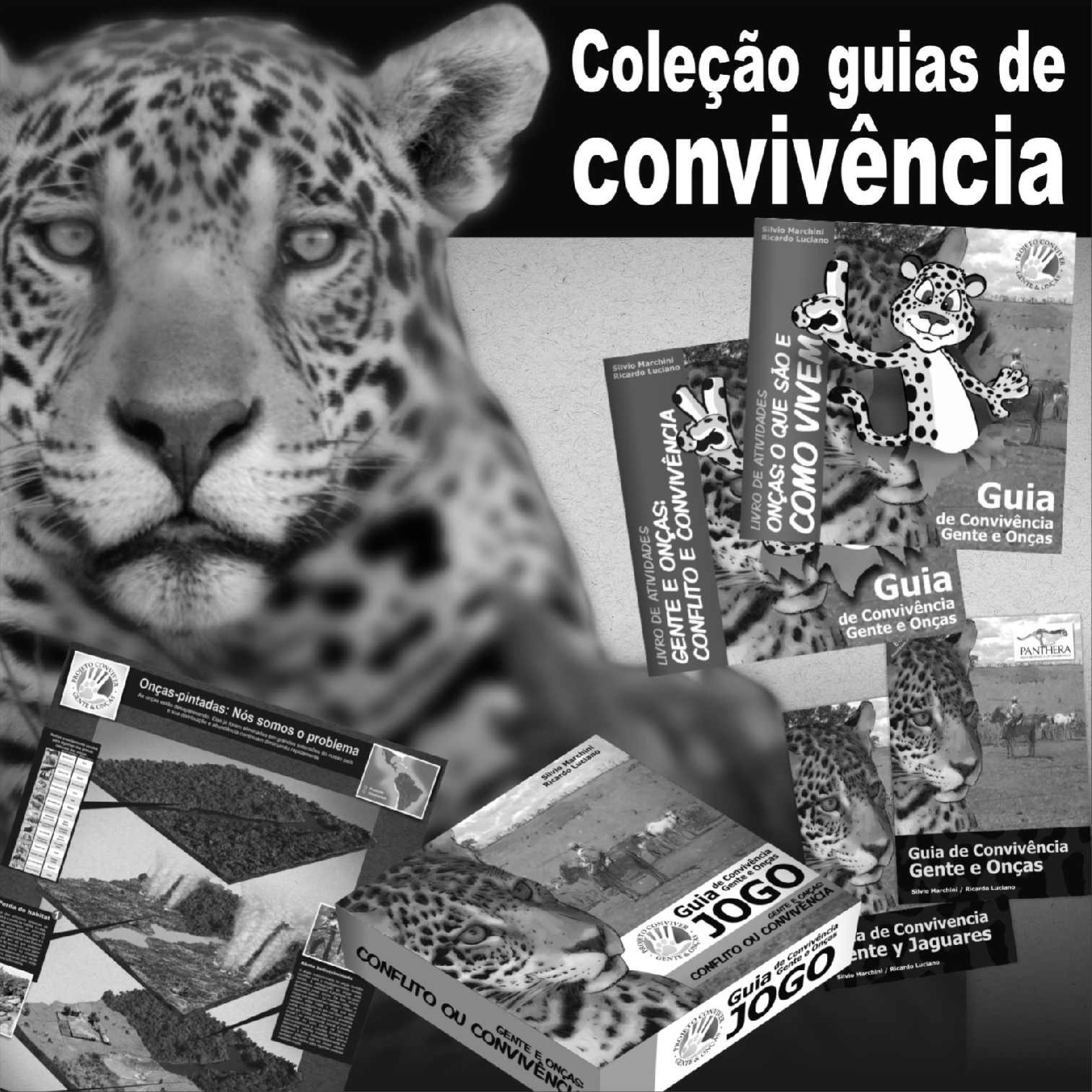

Coleção guias de convivência
Silvio Marchini
Ricardo Luciano
LIVRO DE ATIVIDADES
ONÇAS: O QUE SÃO E COMO VIVEM
Guia de Convivência Gente e Onças
LIVRO DE ATIVIDADES
GENTE E ONÇAS: CONFLITO E CONVIVÊNCIA
Guia de Convivência Gente e Onças
PROJETO CONVIVER GENTE & ONÇAS
PANTHERA
Guia de Convivência Gente e Onças
Silvio Marchini / Ricardo Luciano
Silvio Marchini / Ricardo Luciano
Onças-pintadas: Nós somos o problema
Silvio Marchini
Ricardo Luciano
Guia de Convivência Gente e Onças
JOGO
GENTE E ONÇAS: CONFLITO OU CONVIVÊNCIA

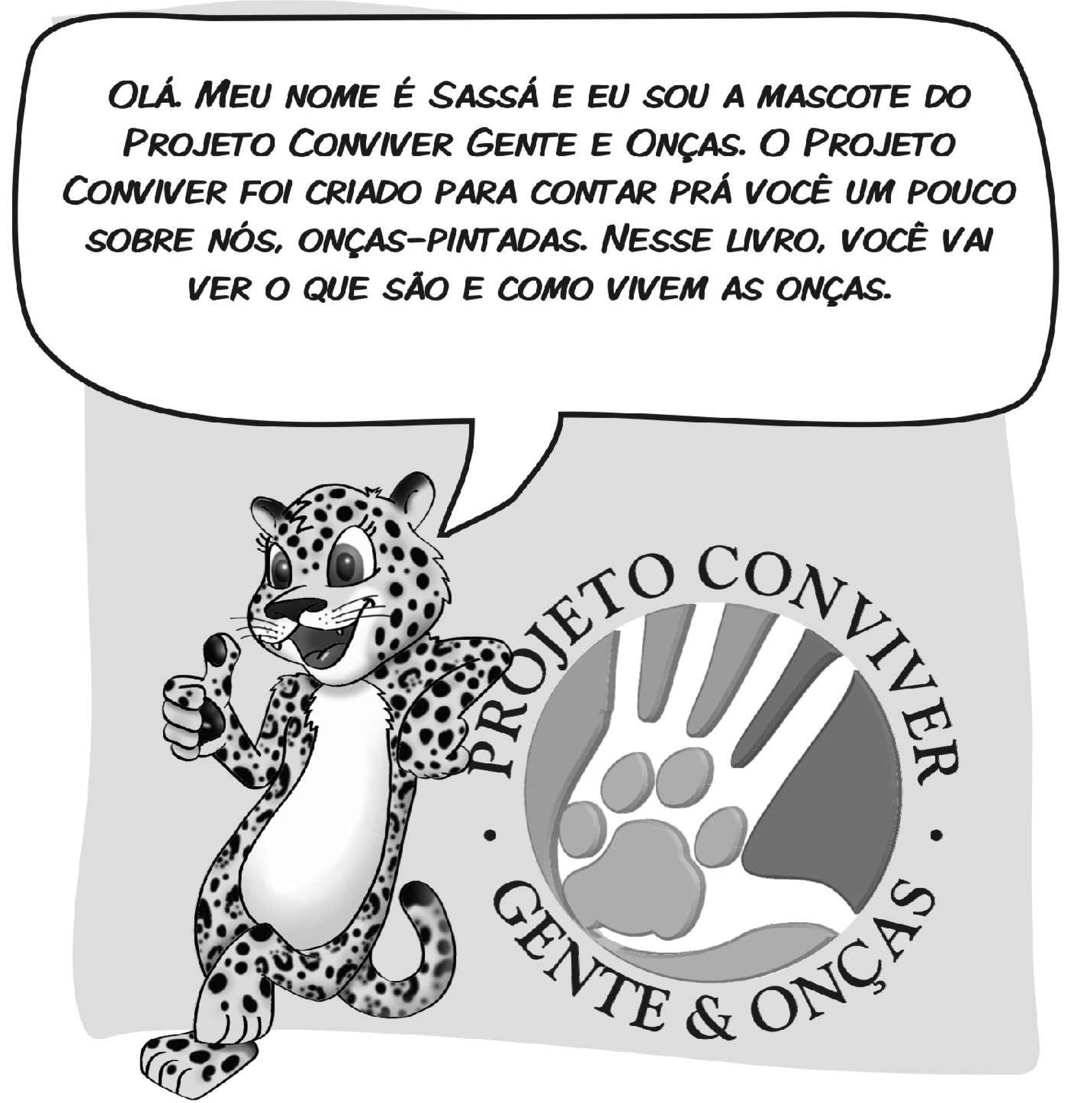
Olá. Meu nome é Sassá e eu sou a mascote do Projeto Conviver Gente e Onças. O Projeto Conviver foi criado para contar prá você um pouco sobre nós, onças-pintadas. Nesse livro, você vai ver o que são e como vivem as onças.
Projeto Conviver
Gente & Onças

# Onças-pintadas: o que são e como vivem

A onça-pintada é o maior carnívoro terrestre do Brasil. A maior onça já encontrada foi um macho que pesava em torno de 150 quilos. As maiores onças são encontradas no Pantanal, onde os machos pesam em torno de 100 quilos e as fêmeas 76 quilos. Uma onça macho pode medir 80 centímetros de altura e dois metros e meio do focinho à ponta da cauda. Em regiões de floresta, como a Amazônia, as onças são um pouco menores.

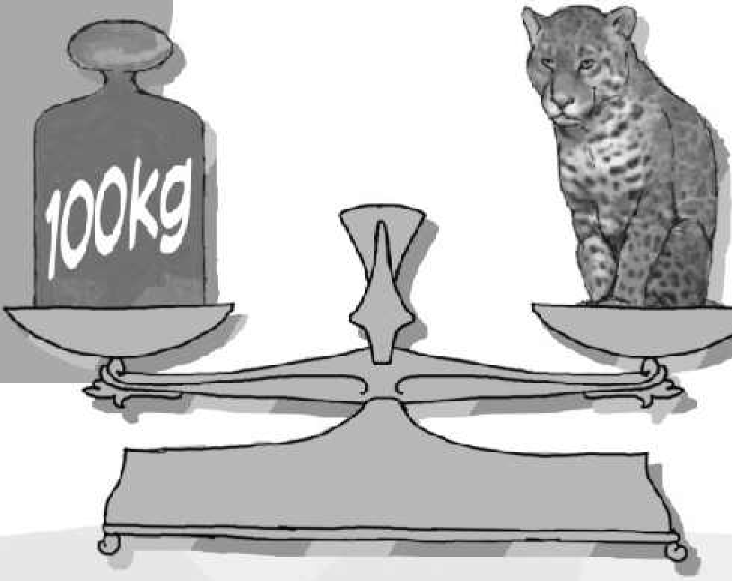


Escreva o seu peso na linha pontilhada e compare com o peso da onça. Quem é o mais pesado?

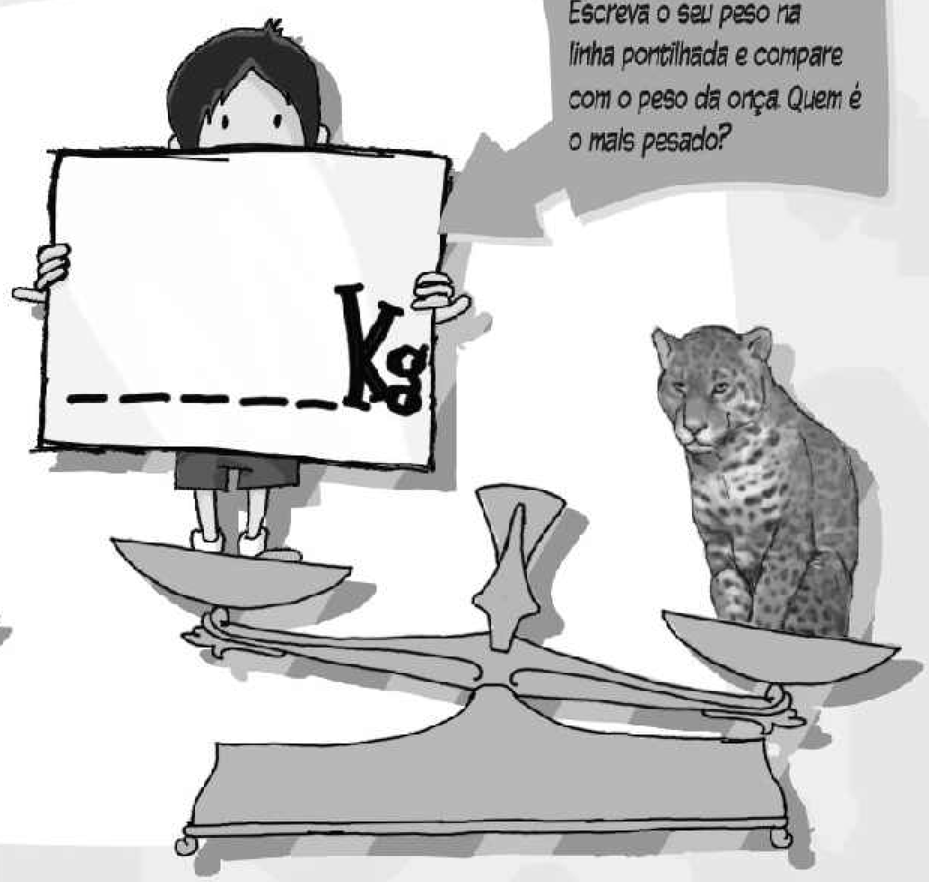


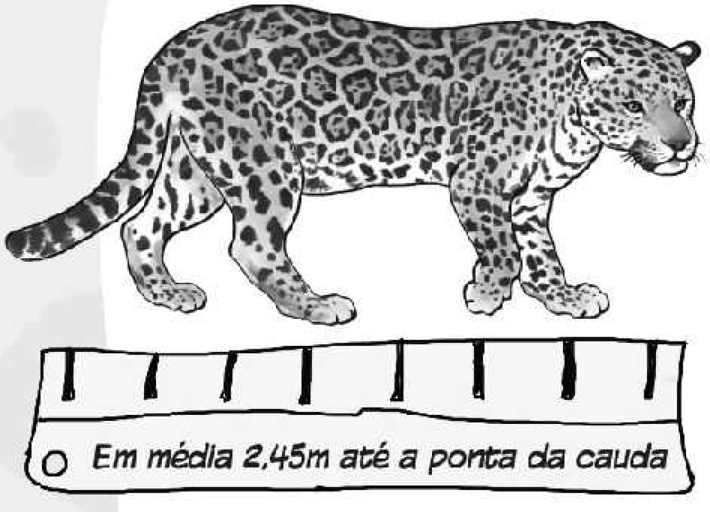


Agora, escreva a sua altura na linha pontilhada e compare com o comprimento da onça. Quem é o mais comprido?

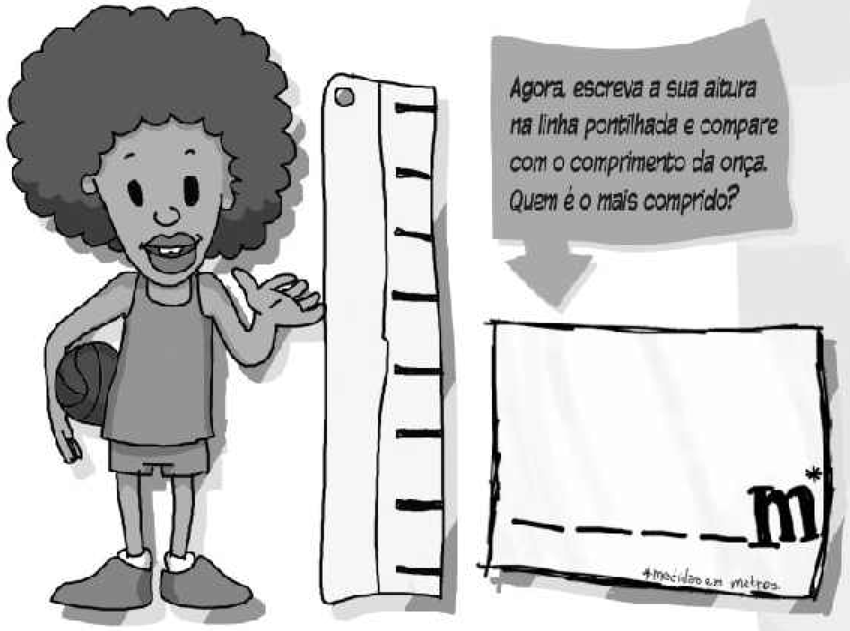


**Experimente:**
Usando um giz de quadro negro e uma fita métrica, anote a medida do comprimento da onça na parede e compare com a sua altura.

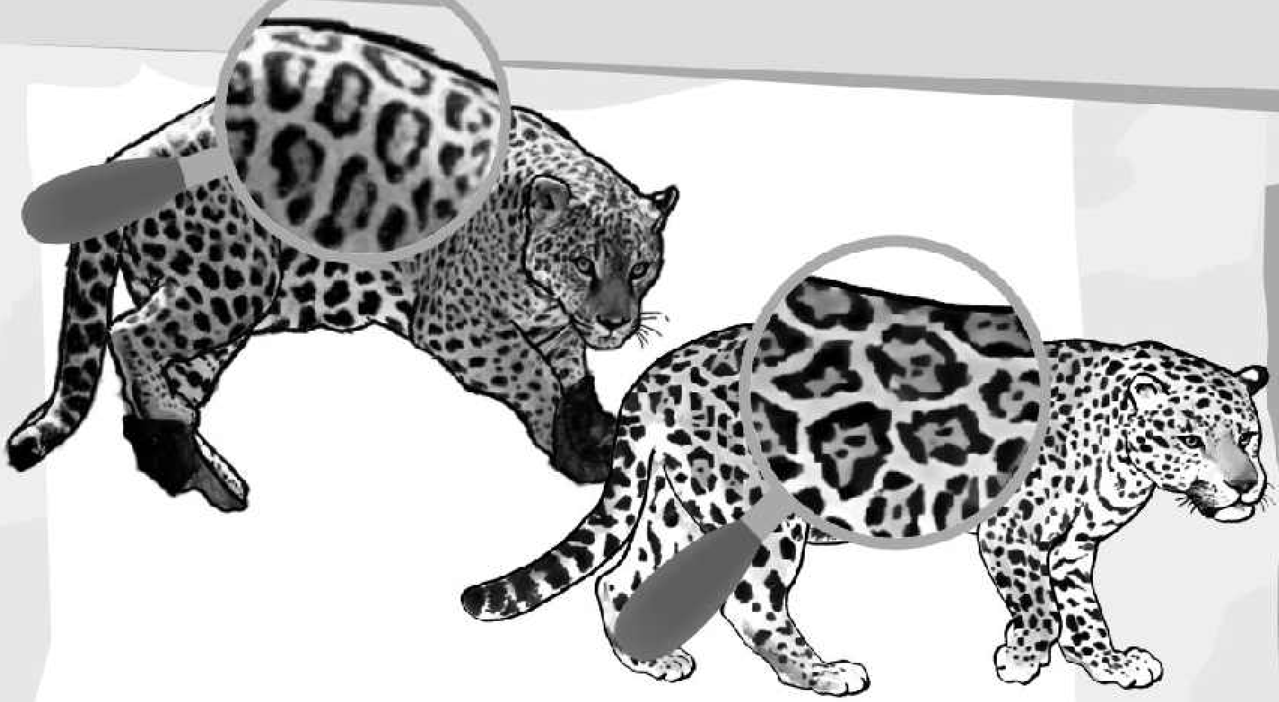

Nada é mais típico de uma onça-pintada do que justamente suas pintas. Cada onça tem seu próprio padrão de pintas, ou seja, não existem duas onças com o mesmo padrão de pintas. Por isso, olhando com atenção para as pintas, é possível identificar cada onça individualmente.

Agora que você viu que a identidade de cada onça são as suas pintas, crie um padrão de pintas identificando a sua onça.

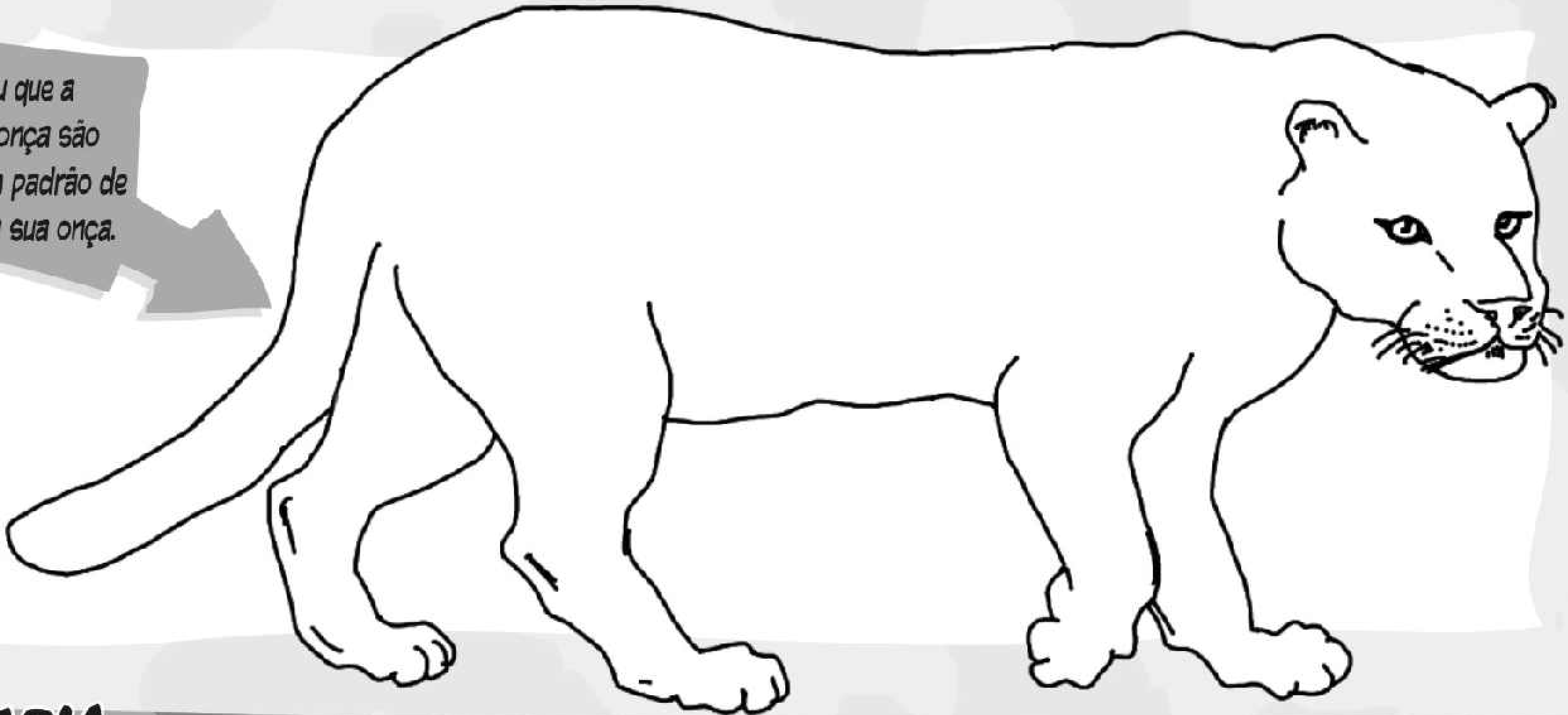

## Você sabia...

Que usando "armadilhas Fotográficas" os cientistas conseguem Fotografar as onças de uma região inteira, identificar cada uma delas pelo seu padrão de pintas e, desse modo, saber quantas onças existem?
Vamos aprender como esse equipamento Funciona.

1- IDENTIFICANDO AS PRINCIPAIS PARTES DE UMA ARMADILHA FOTOGRAFICA

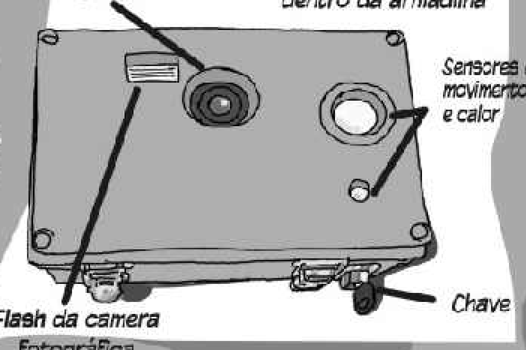


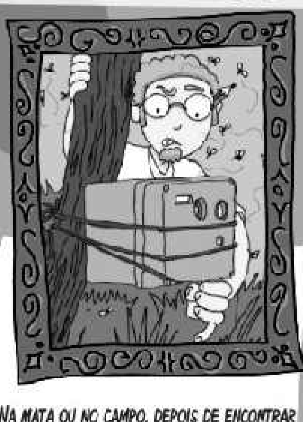

2 – NA MATA OU NO CAMPO, DEPOIS DE ENCONTRAR UM BOM LUGAR PARA COLOCAR A ARMADILHA, É SÓ POSICIONÁ-LA, USÁ-LA E AGUARDAR...

3 – ...QUE UMA ONÇA, AO PASSAR EM FRENTE AOS SENSORES DE CALOR E MOVIMENTO DA ARMADILHA, ACIONE O MECAMISMO QUE DISPARA A CÂMERA FOTOGRÁFICA...

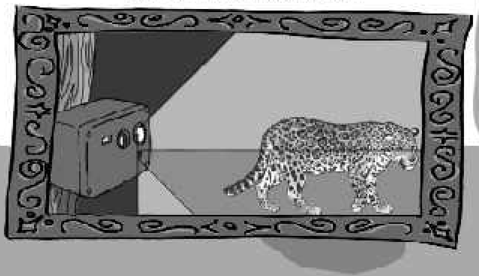

4 – ...PRODUZINDO UMA BELA FOTO DE ONÇA, COMO ESSA TIRADA POR UMA DE NOSSAS ARMADILHAS FOTOGRÁFICAS NA AMAZÔNIA.

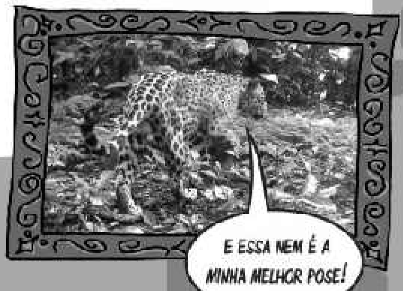

# ONÇAS-PINTADAS: O QUE SÃO E COMO VIVEM

A onça-pintada amarela e a onça preta pertencem, na verdade, à mesma espécie. A única diferença entre elas está na quantidade de pigmento escuro (melanina) nos pêlos. Assim, como existe gente loira e gente morena, existem os dois tipos de onças.

Não existe evidência de que onças amarelas e pretas se comportem de maneira diferente, embora muitos acreditem que essa ou aquela é mais perigosa, mais arredia, ataca mais o gado e assim por diante.

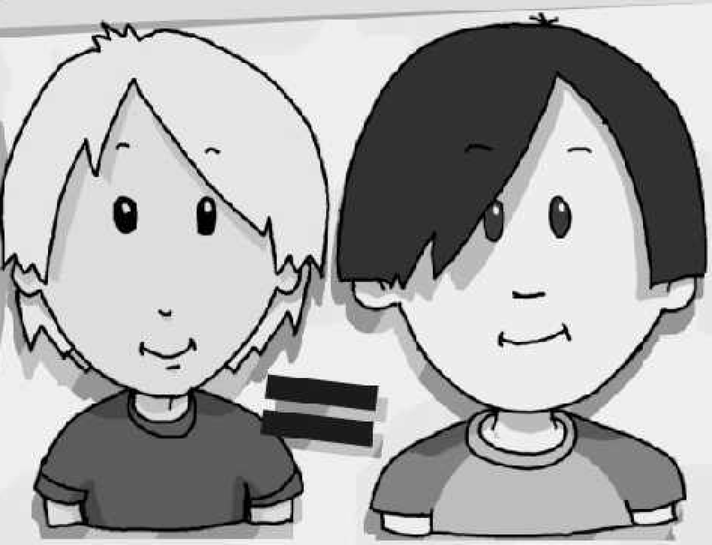

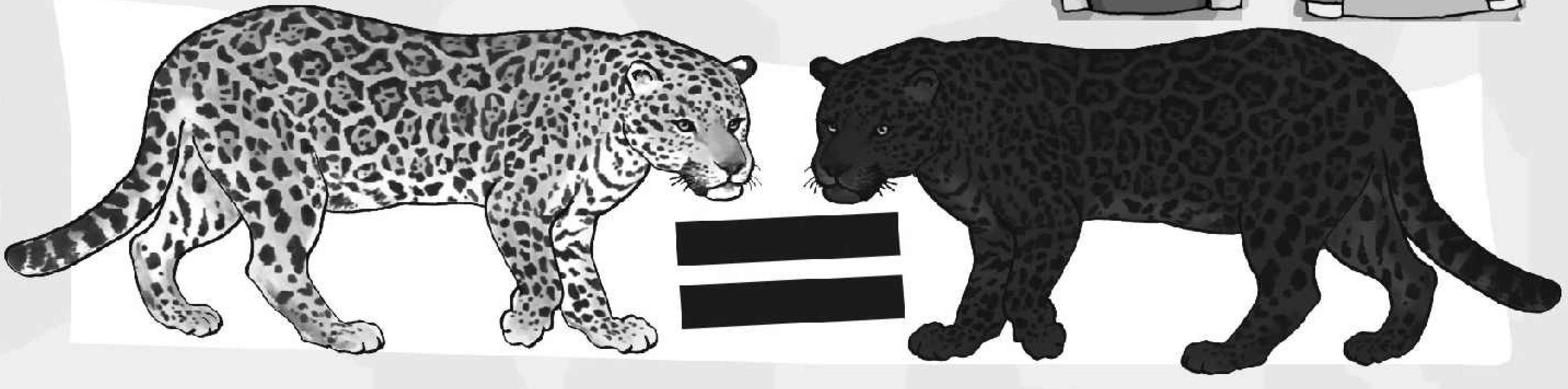

Onças-pintadas são, às vezes, confundidas com onças-pardas. Onça-pintada (amarela ou preta) e onça-parda, essas sim, são espécies diferentes (embora sejam aparentadas, como humanos e macacos)

A onça-parda, também conhecida como suçuarana, puma, leão-baio ou onça-vermelha, é menor e tem aparência menos robusta. Além disso, a onça-parda não ruge (esturra) como a onça-pintada. Ela produz um som mais parecido com um miado.

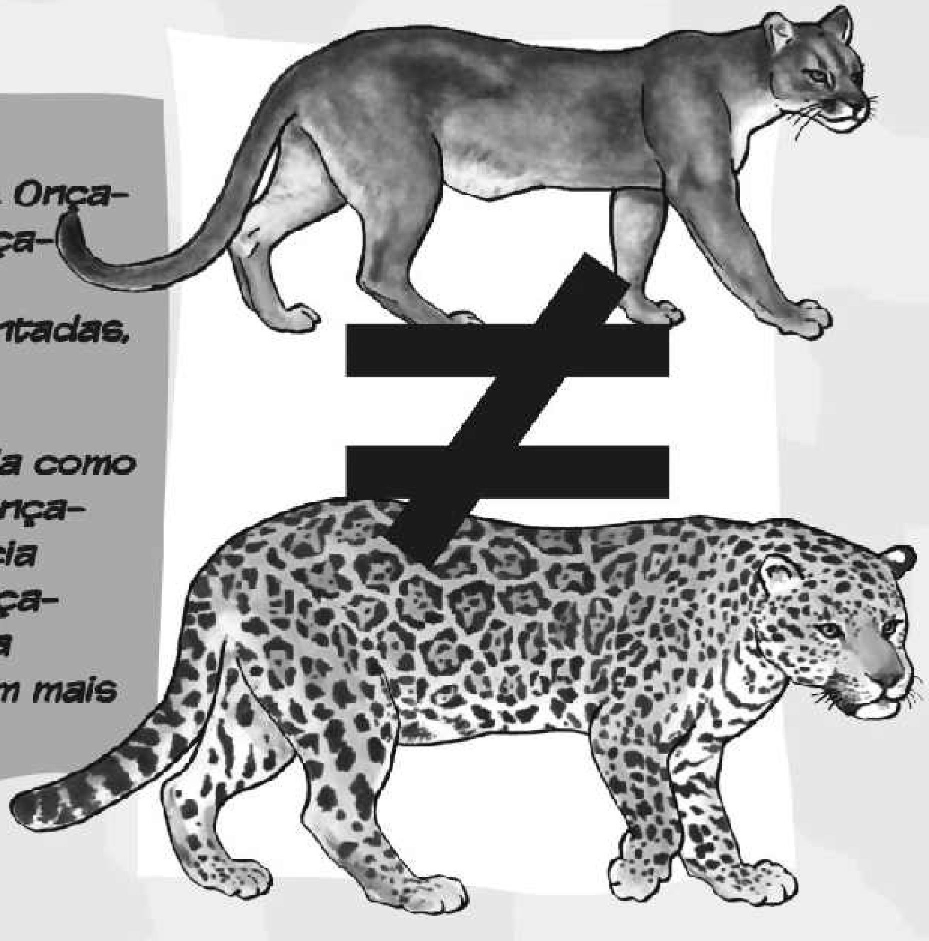

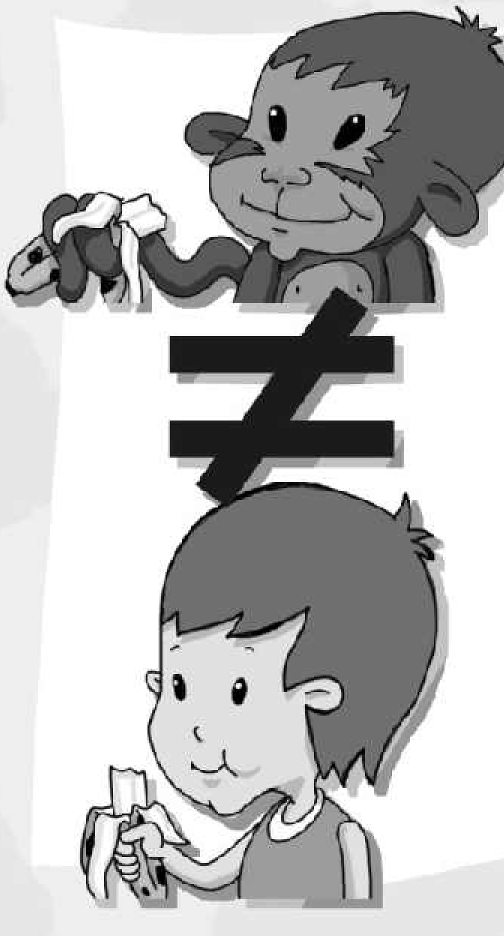

X maximo = 13cm
Y maximo = 12cm

*Ainda mais fácil de confundir são os rastros (pegadas ou batidas) das duas espécies de onças: pintada e parda. É possível, porém, distinguir entre os dois rastros. O rastro da onça-pintada,geralmente maior que o da parda, tem os dedos arredondados, com a largura total um pouco maior que o comprimento.*

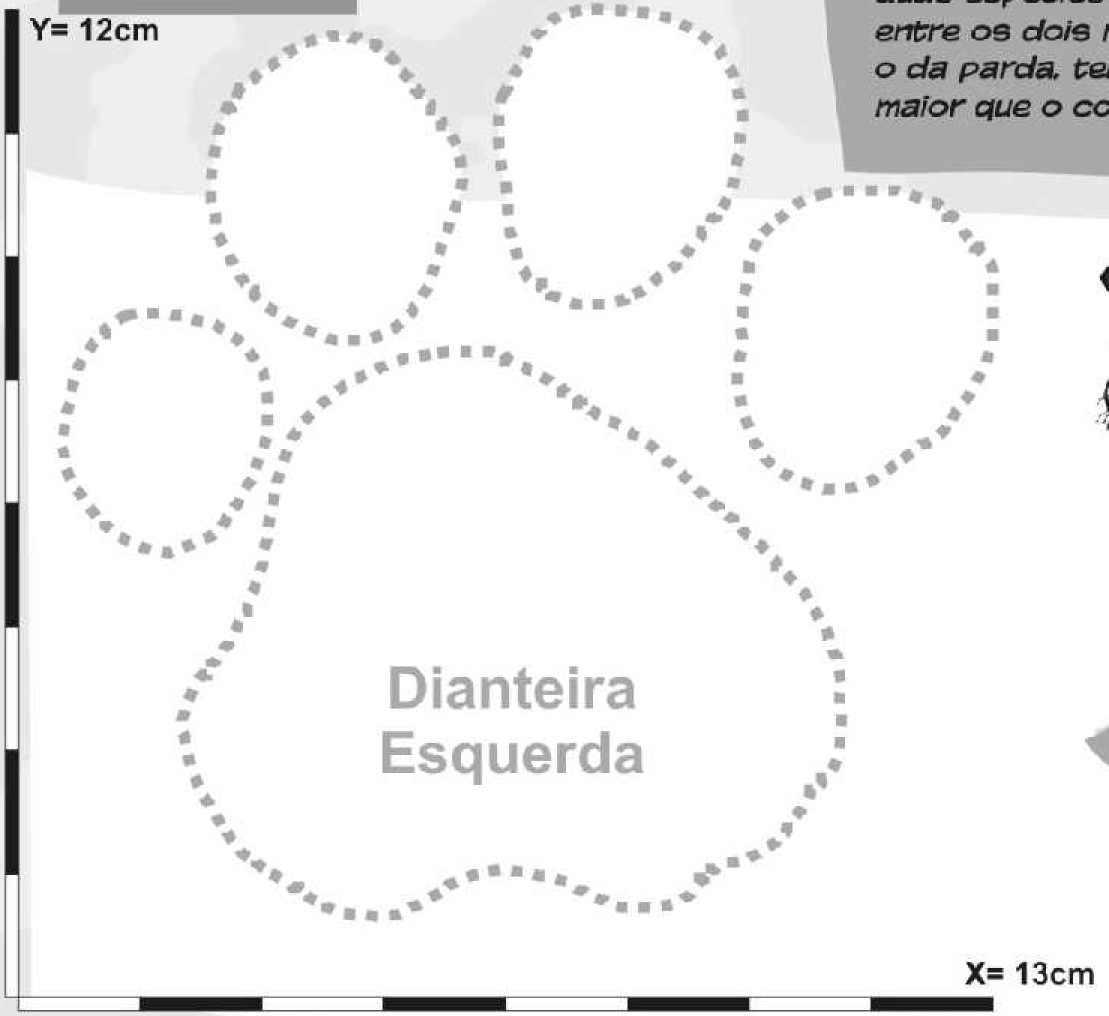


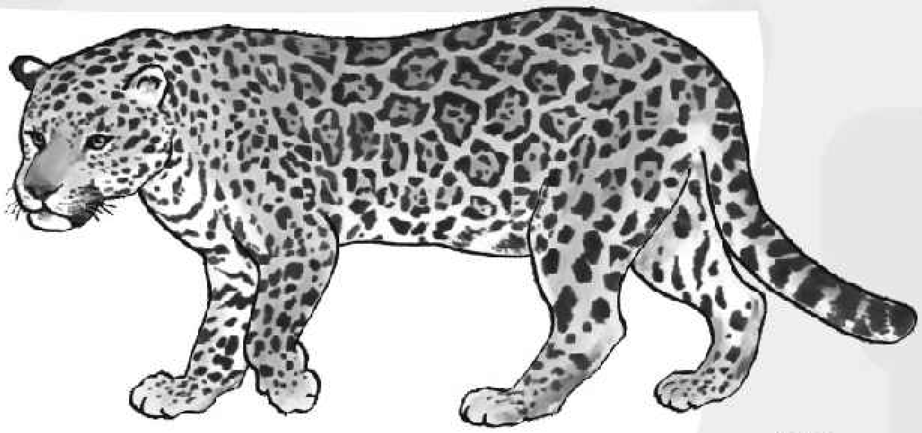

Contorne sua mão com um lápis em cima da pegada da onça-pintada e imagine o tamanho dessa pata.

X maximo = 10cm
Y maximo = 9cm

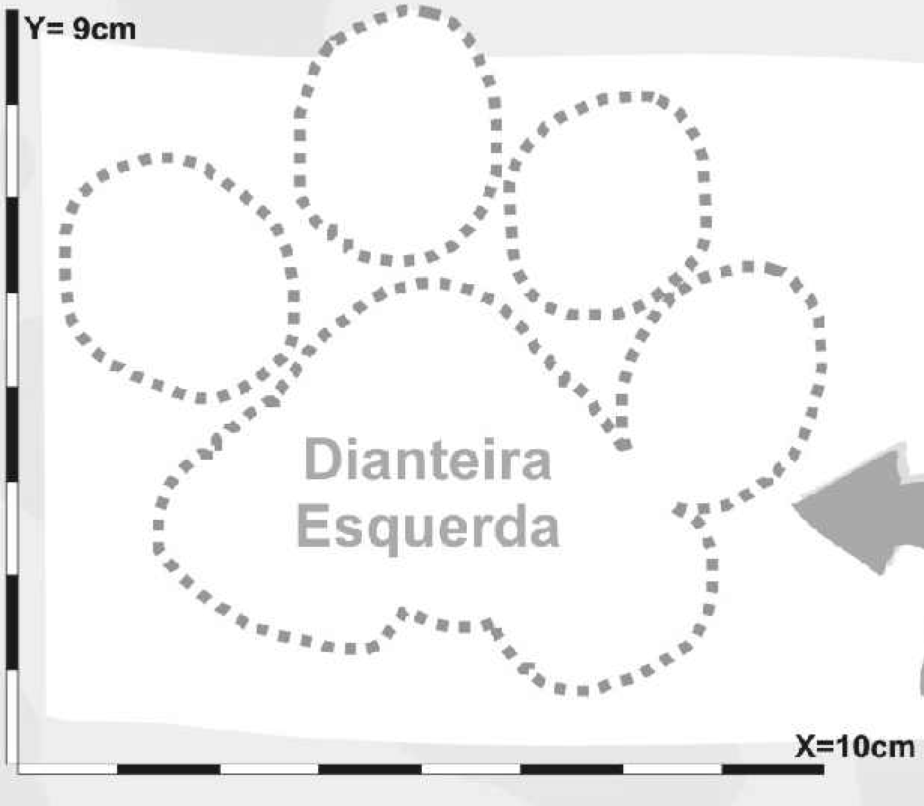


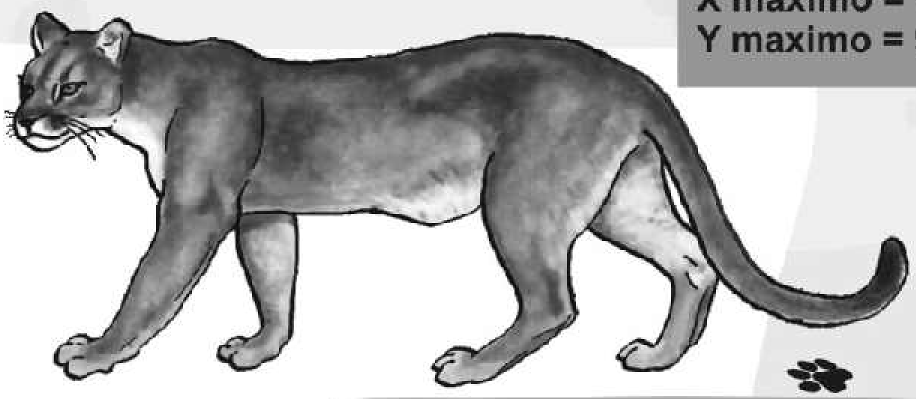

Contorne sua mão com um lápis em cima da pegada da onça-parda e imagine o tamanho dessa pata.

*Já a pegada da onça-parda é mais comprida do que larga e os dedos tendem a ser pontudos (parecida com a pegada do cachorro doméstico, porém sem marcar as unhas, como fazem os cachorros)*

# ONÇAS-PINTADAS: O QUE SÃO E COMO VIVEM

(1) Identifique as pegadas e depois correlacione com os bichos corretos

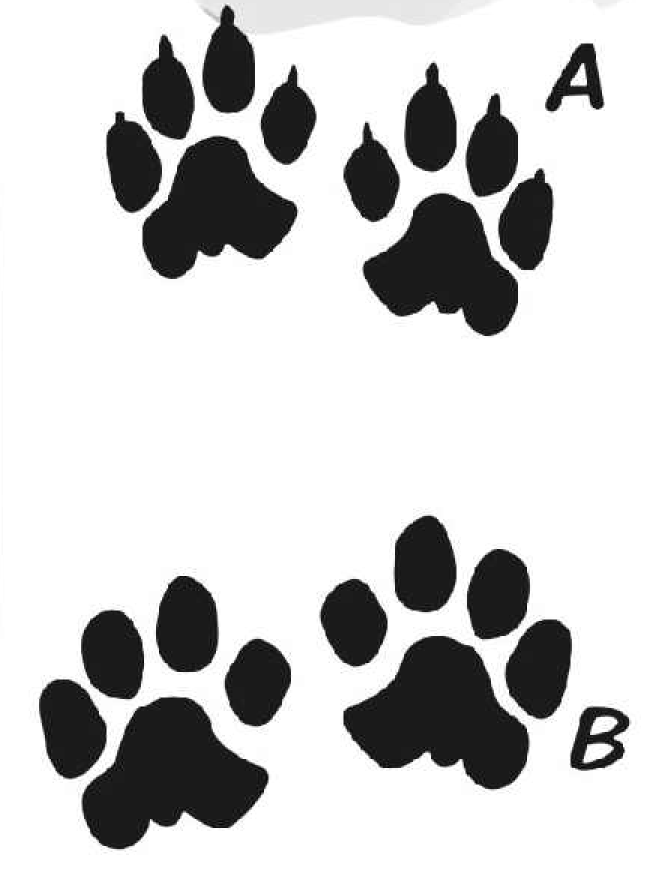


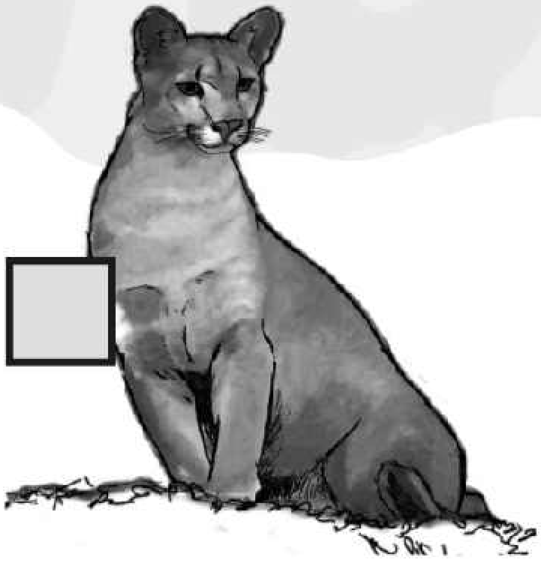

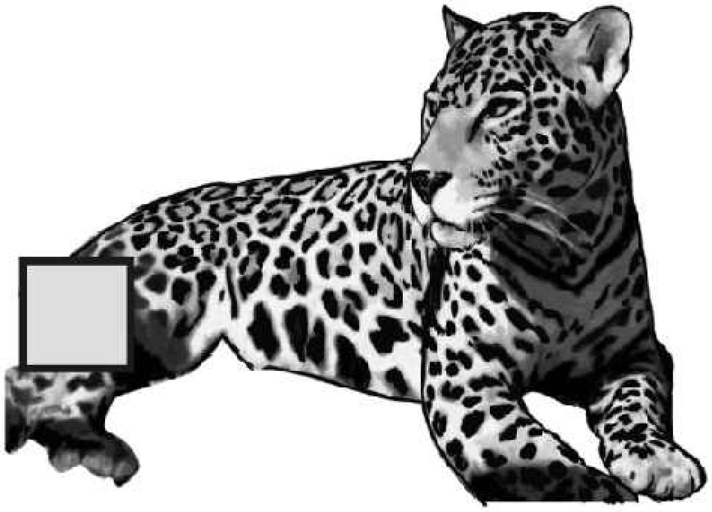

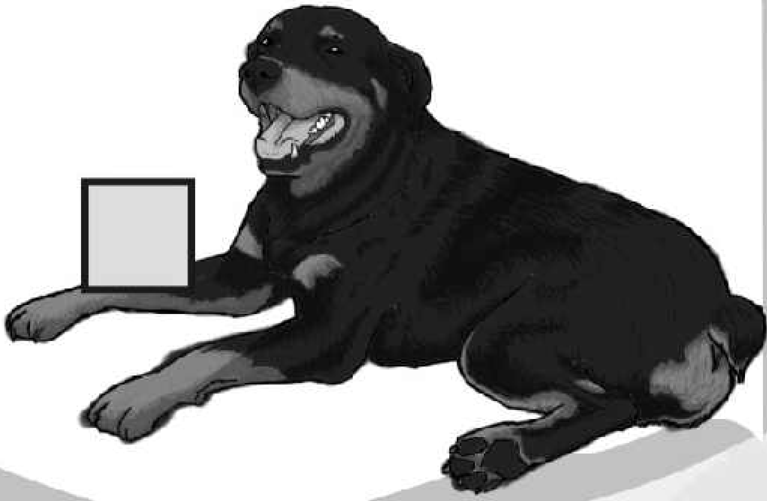

As onças fêmeas começam a ter filhotes entre 2 e 3 anos de idade e os machos entre 3 ou 4 anos. Elas podem ter até 4 filhotinhos por ninhada mas na maioria das vezes têm apenas dois filhotes. As onças dão cria a cada um ano e meio ou dois.

Como na mata as onças vivem entre 11 e 15 anos (no zoológico elas vivem até 23 anos), uma fêmea não tem mais do que 10 ou 12 filhotes ao longo de sua vida.

Que tal um probleminha de matemática? Se a Sassá começar a ter filhotes aos 3 anos de idade, tiver ninhadas de 2 filhotinhos a cada 2 anos, e for caçada depois de completar 13 anos de idade, quantos filhotes ela vai deixar?

(2)

Agora que você encontrou a resposta, selecione o quadro abaixo quecorresponde à sua resposta e invente um nome para cada filhotinho da Sassá.

A

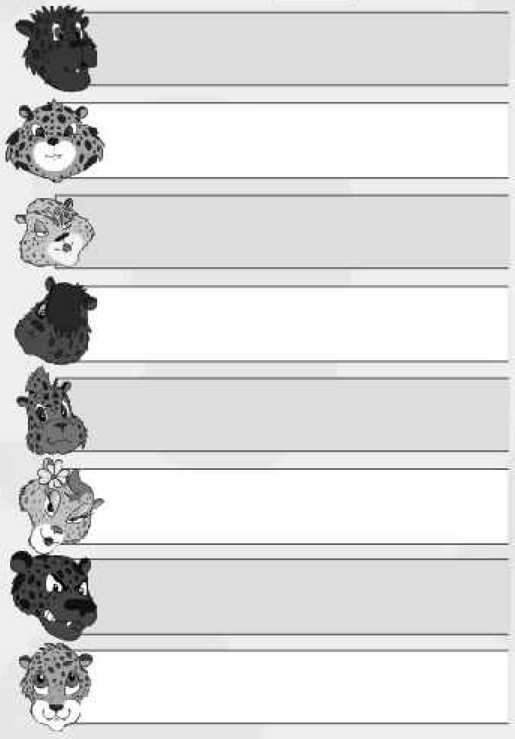

B

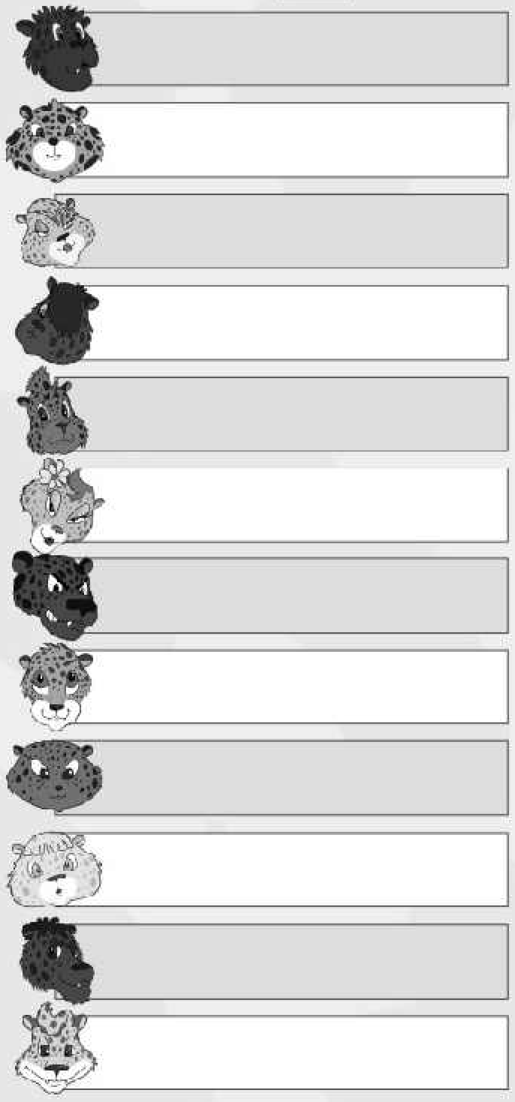

C

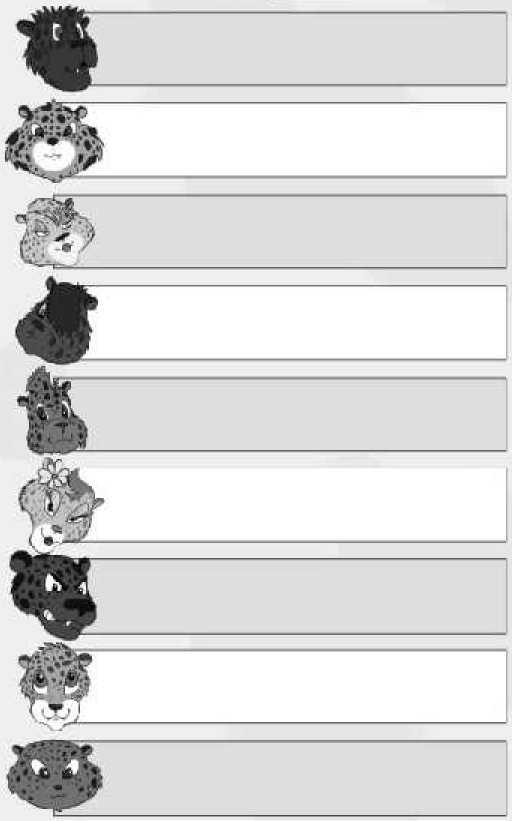

D

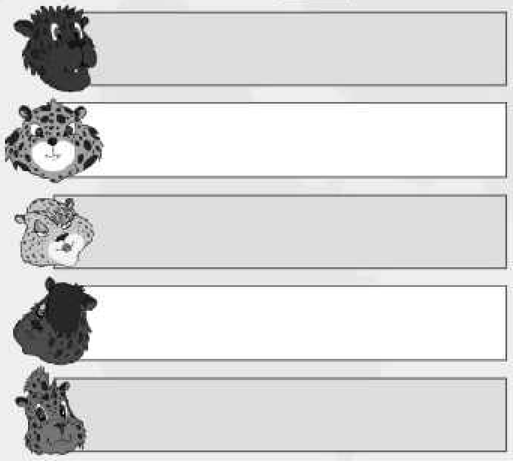

Os filhotinhos da onça são cegos até duas semanas depois de nascer. Eles começam a comer carne por volta dos 2 meses e meio de idade, mamam até por volta do terceiro mês e começam a sair do ninho para caminhar com a mãe aos 6 meses. Eles ficam com a mãe até um ano e meio ou dois de idade.

# ONÇAS-PINTADAS: O QUE SÃO E COMO VIVEM

*Como são grandes, as **onças** preferem presas grandes. Suas presas naturais incluem anta, veado, porco-do mato, capivara, tamanduás, tatús, bichos-preguiças e jacarés. Além disso, a **onça** come qualquer animal pequeno que possa capturar, incluindo macacos, pássaros, jabutis e tartarugas, sapos e até peixes.*

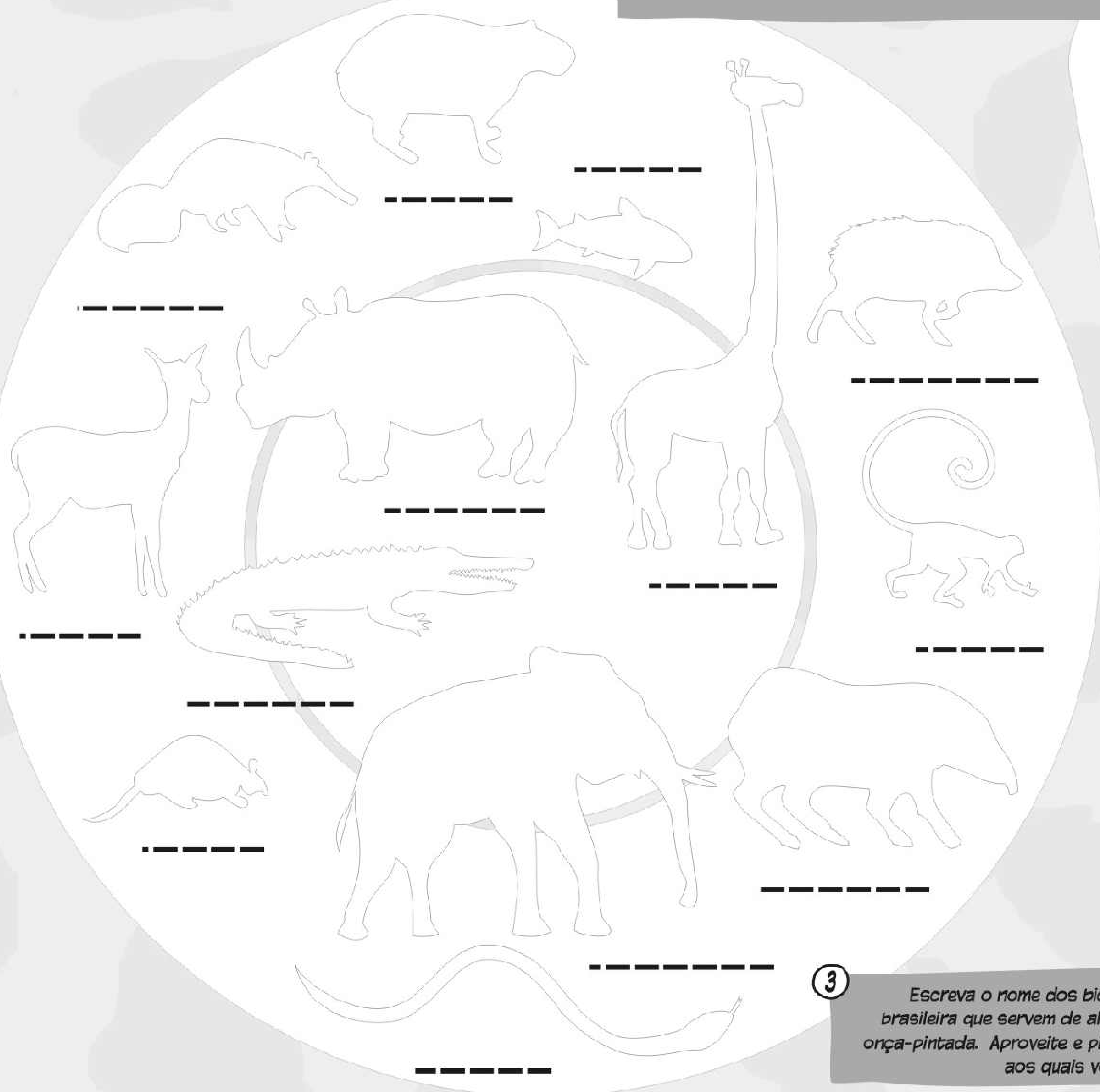

**3** *Escreva o nome dos bichos da Fauna brasileira que servem de alimento para a onça-pintada. Aproveite e pinte os bichos aos quais você deu nome.*

Uma onça-pintada pode comer de 35 a 40 quilos de carne por semana. O quanto cada onça come, porém, depende da necessidade (uma fêmea amamentando seus filhotes vai precisar mais) e da facilidade de encontrar as presas. Uma onça pode comer até 25 quilos de uma vez ou passar vários dias faminta sem nada para comer, dependendo da ocasião. **Por isso, ela prefere caçar animais grandes. Quanto maior a presa, mais comida e, portanto, melhor prá onça.**

4

**Na coluna ao lado, indique a ordem de preferência de presas da onça (escreva os números de 1 a 7: a presa mais interessante para a onça é a número 1, a presa menos interessante é a número 7)**

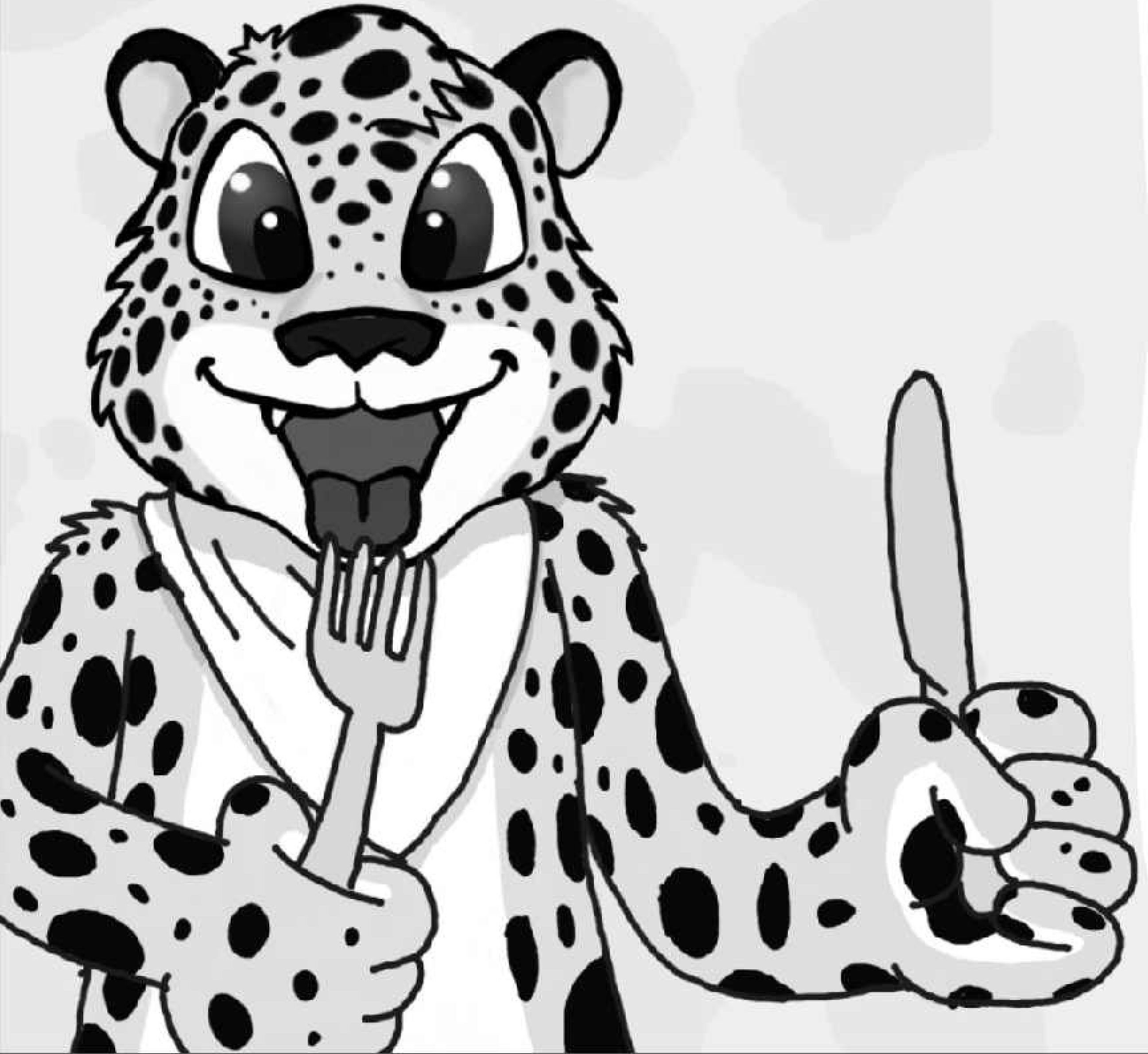

## Ordem de preferência De presas da onça

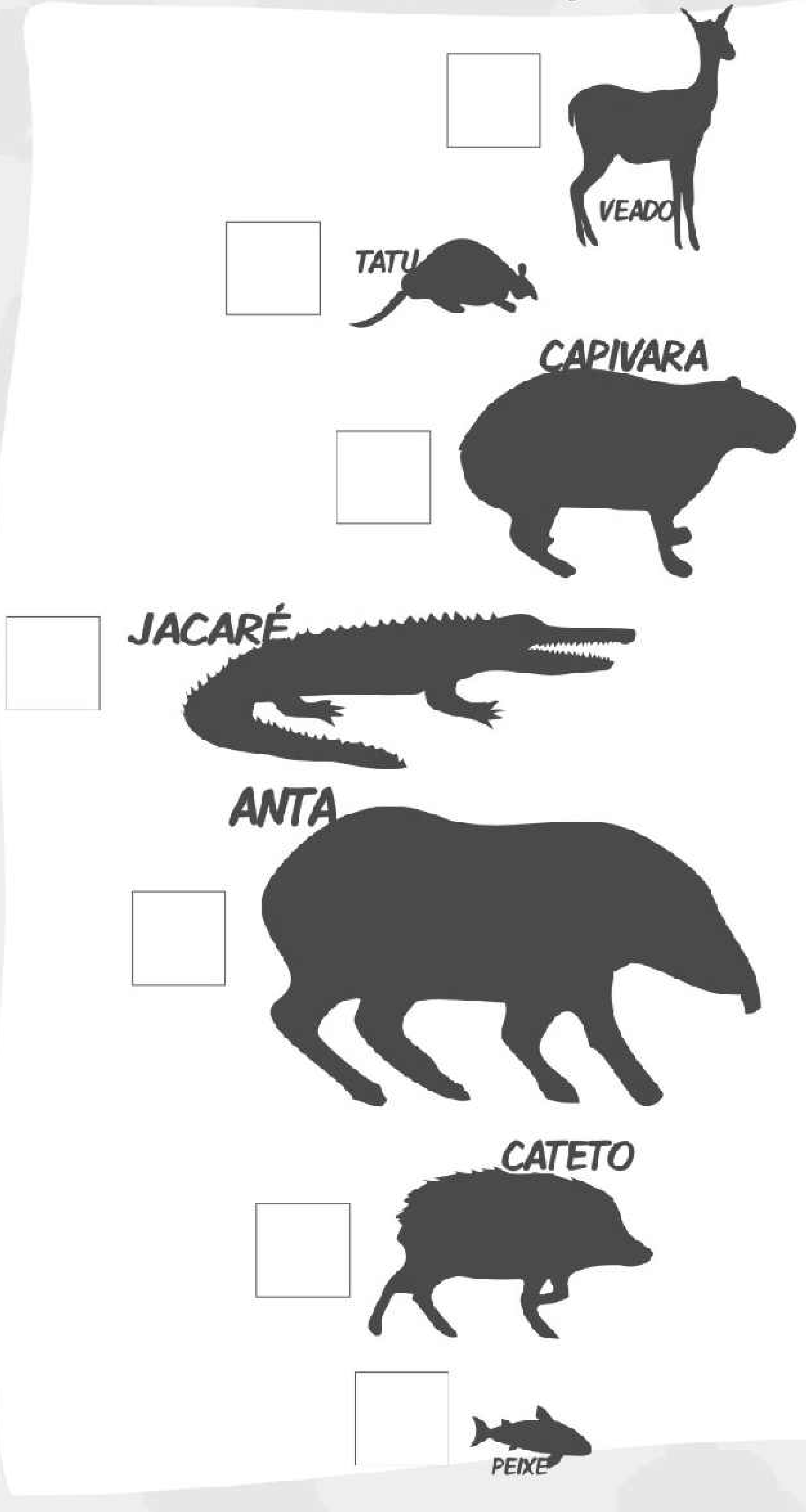

# ONÇAS-PINTADAS: O QUE SÃO E COMO VIVEM

Mas nem sempre as presas maiores são fáceis de encontrar. Na verdade, animais grandes, como a anta, tendem a existir em baixas densidades (ou seja, uma aqui e a próxima a quilômetros de distância). Quando as presas grandes são raras, a onça tem que se contentar com presas menores que são encontradas e capturadas com mais facilidade. O que a onça acaba comendo, portanto, depende não só do tamanho da presa, **mas da facilidade de encontrar e capturar aquela presa.**

TERRITÓRIO DA ONÇA

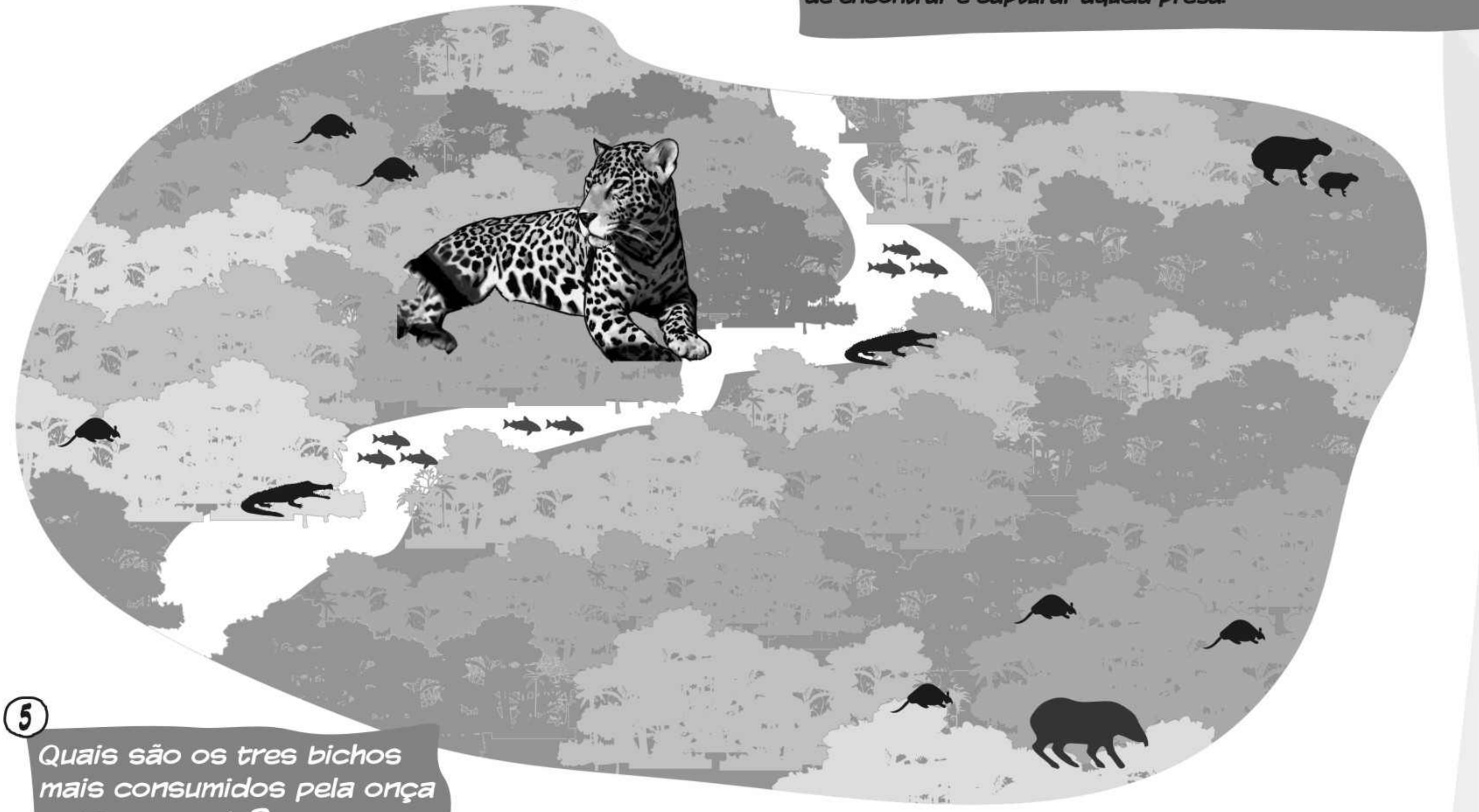

5

Quais são os tres bichos mais consumidos pela onça nesse exemplo?

| | |
|---|---|
| 1° | |
| 2° | |
| 3° | |

Na Figura abaixo você vai ver que a presa mais consumida pela onça-pintada varia de lugar para lugar. No Pantanal, por exemplo, os bichos que ela mais come são o jacaré e o queixada.

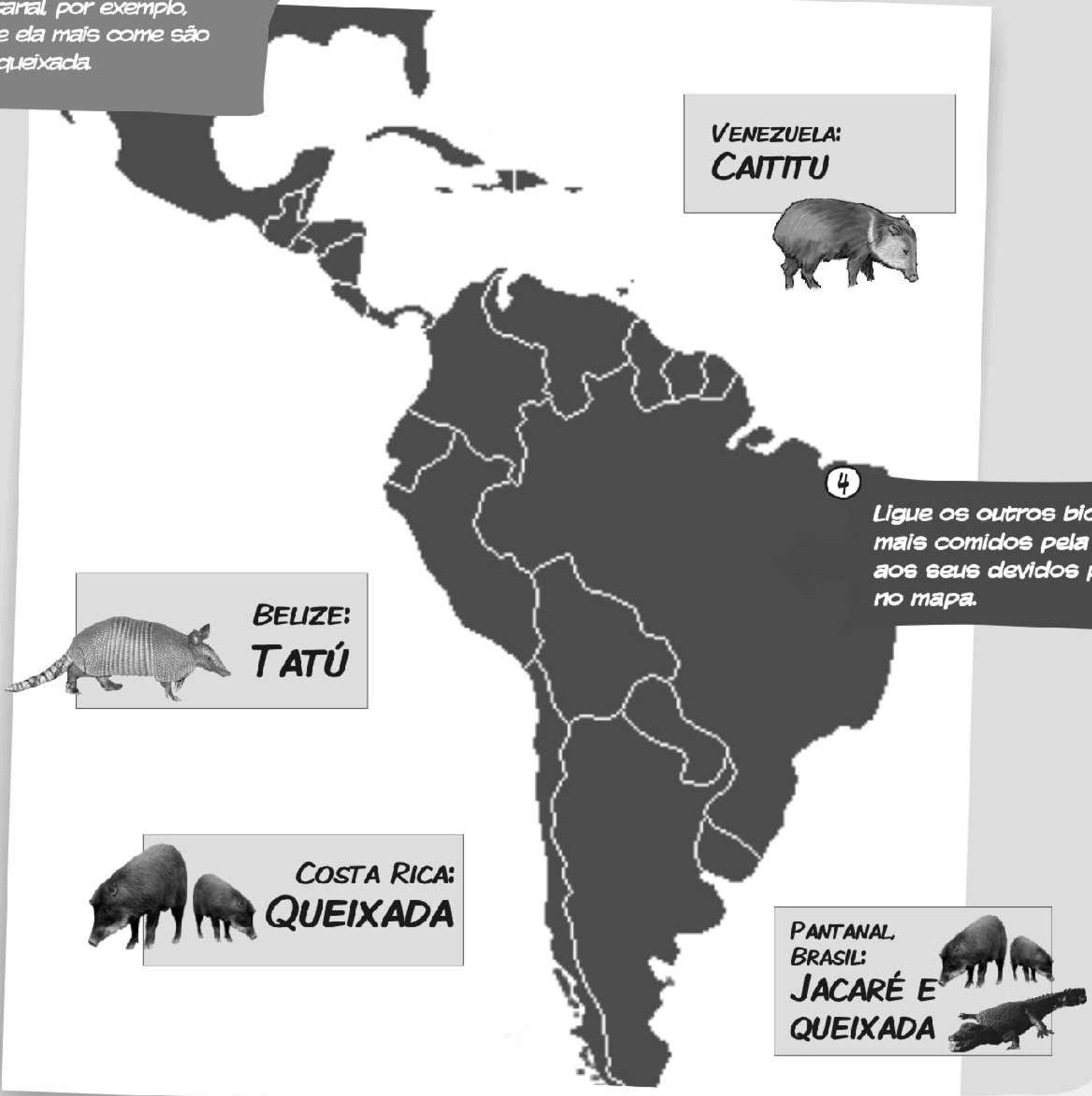


4

Ligue os outros bichos mais comidos pela onça aos seus devidos países no mapa.

# ONÇAS-PINTADAS: O QUE SÃO E COMO VIVEM

O habitat das onças-pintadas inclui as Florestas, como as da Amazônia e Mata Atlântica, os cerrados e as áreas alagáveis, como o Pantanal.

As onças preferem viver em locais com grande disponibilidade de água - como as margens dos rios e as áreas alagadas - e vegetação densa, que lhes sirva de abrigo e onde possam espreitar suas presas.

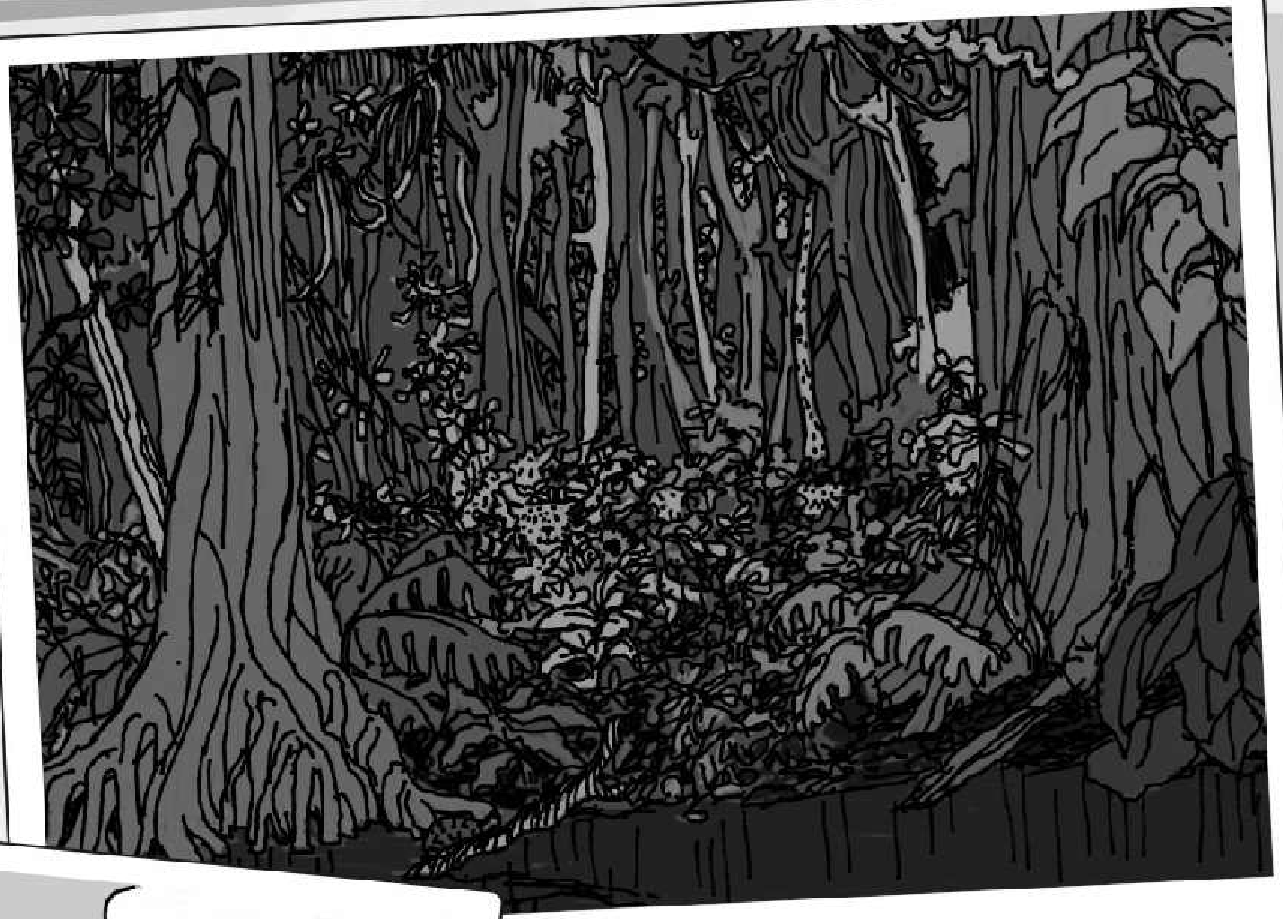

AMAZÔNIA E MATA ATLÂNTICA

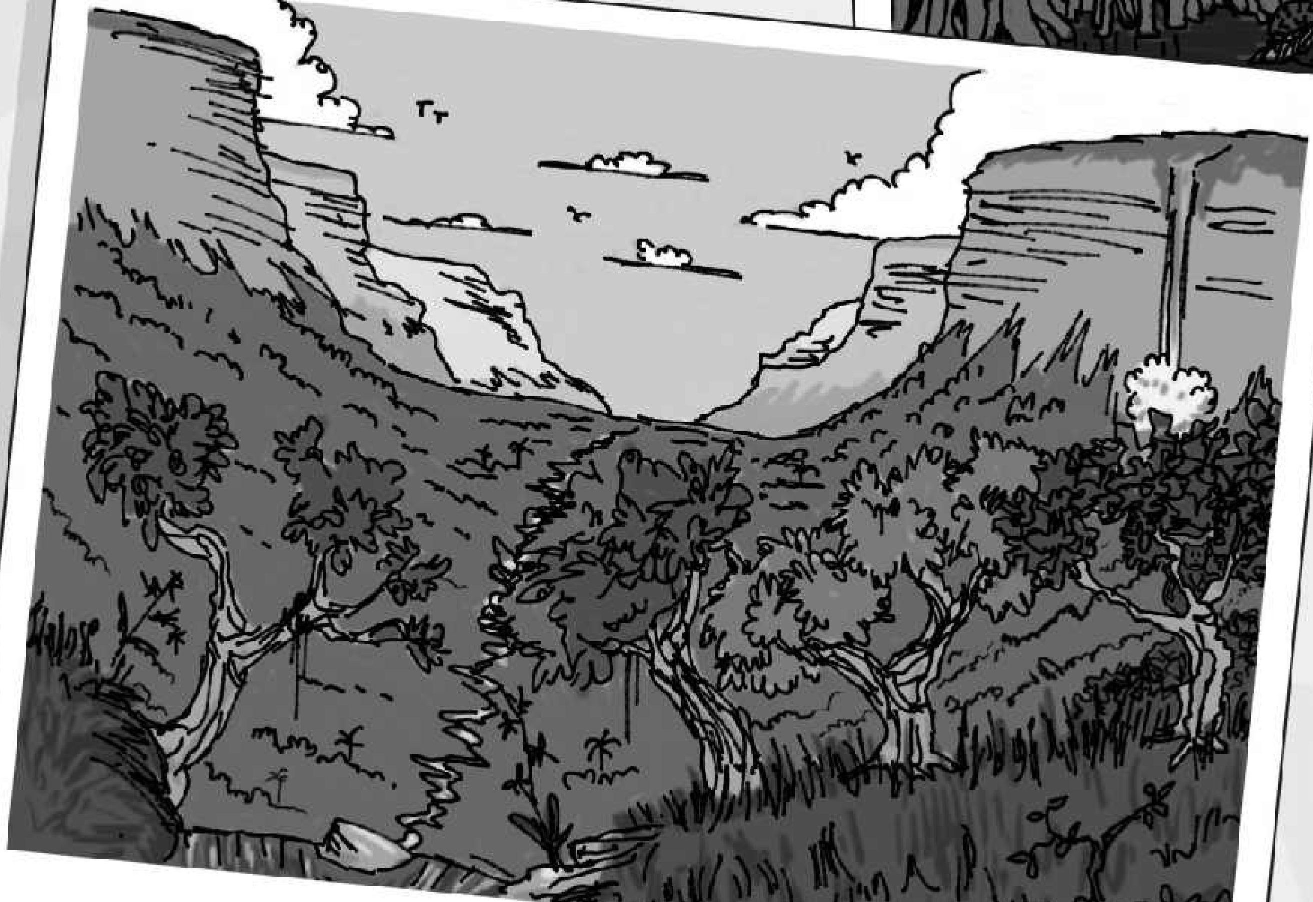

CERRADO

Você já consegue identificar uma onça com Facilidade?

Então vamos ver: encontre a onça em seu habitat

6

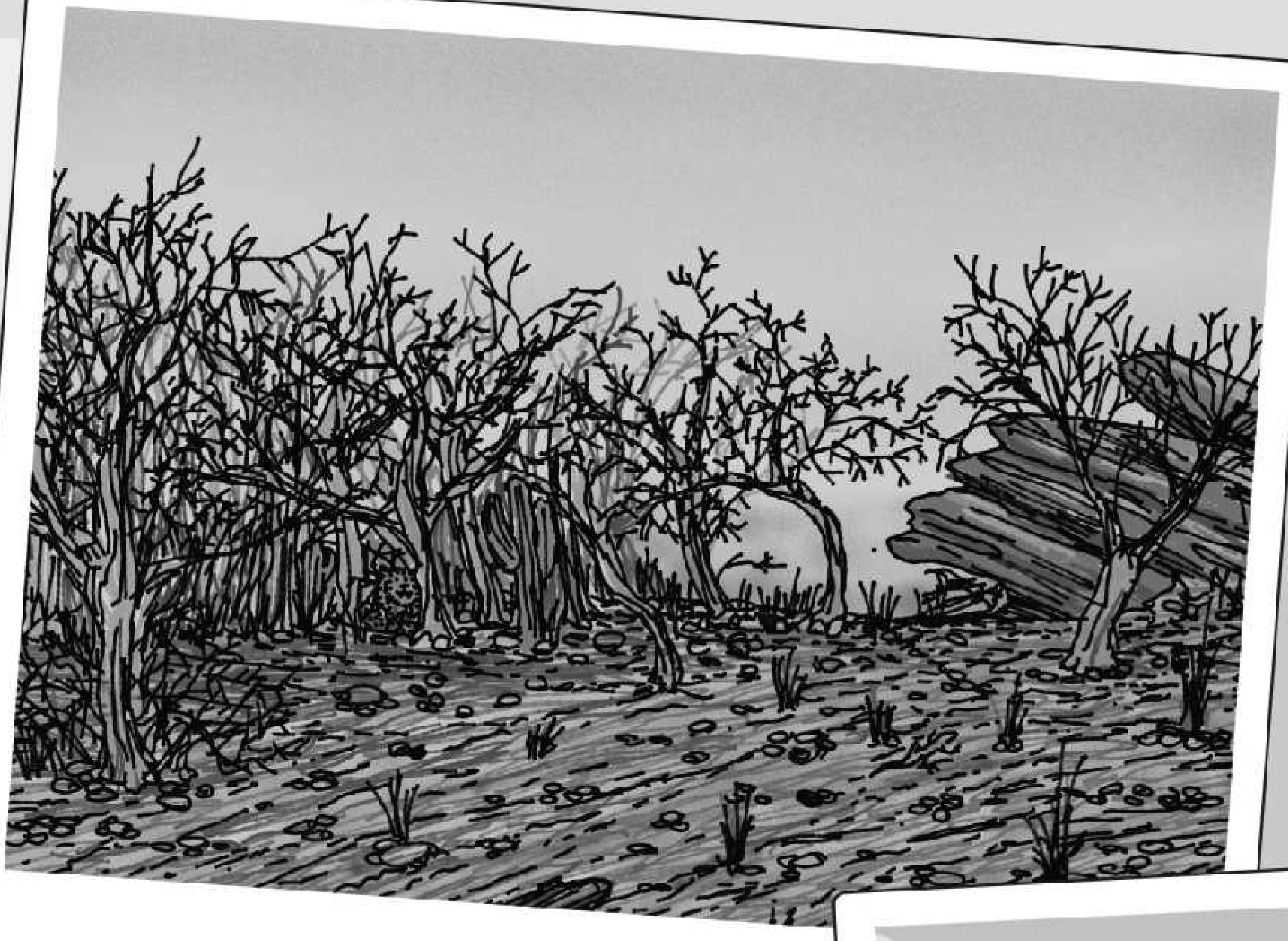

CAATINGA

Você conseguiu encontrar uma onça em cada uma dessas paisagens? Então agora escreva abaixo qual dessas paisagens melhor representa o lugar onde você mora.

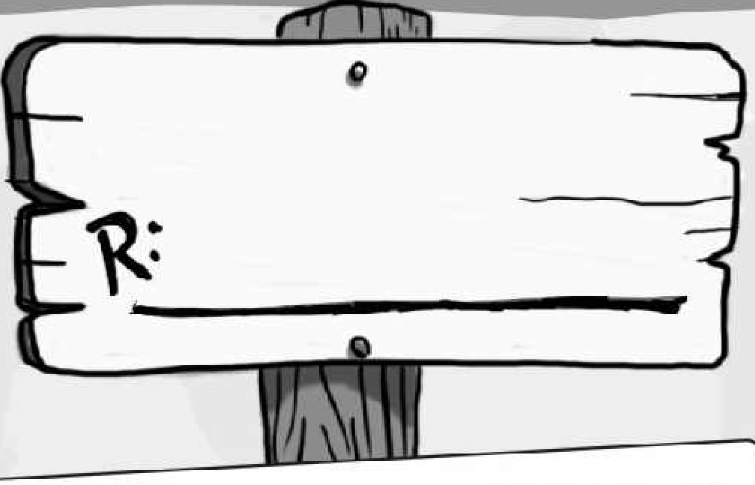


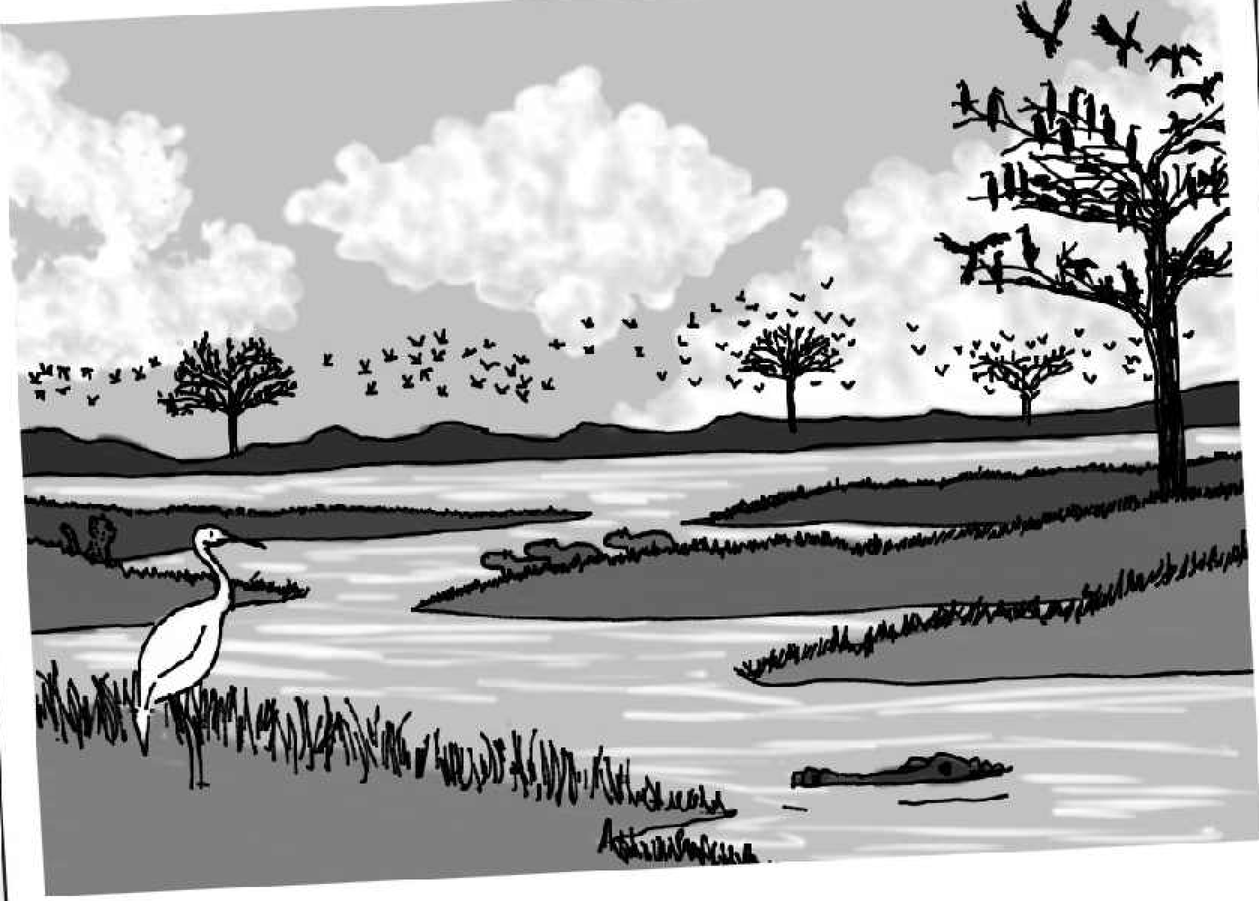

PANTANAL

# ONÇAS-PINTADAS: O QUE SÃO E COMO VIVEM

Concluindo, uma onça-pintada precisa de **abrigo, água e alimento** para viver. Além disso, precisa de um parceiro ou uma parceira para se reproduzir. Para encontrar tudo isso, a onça precisa também de espaço, muito espaço.

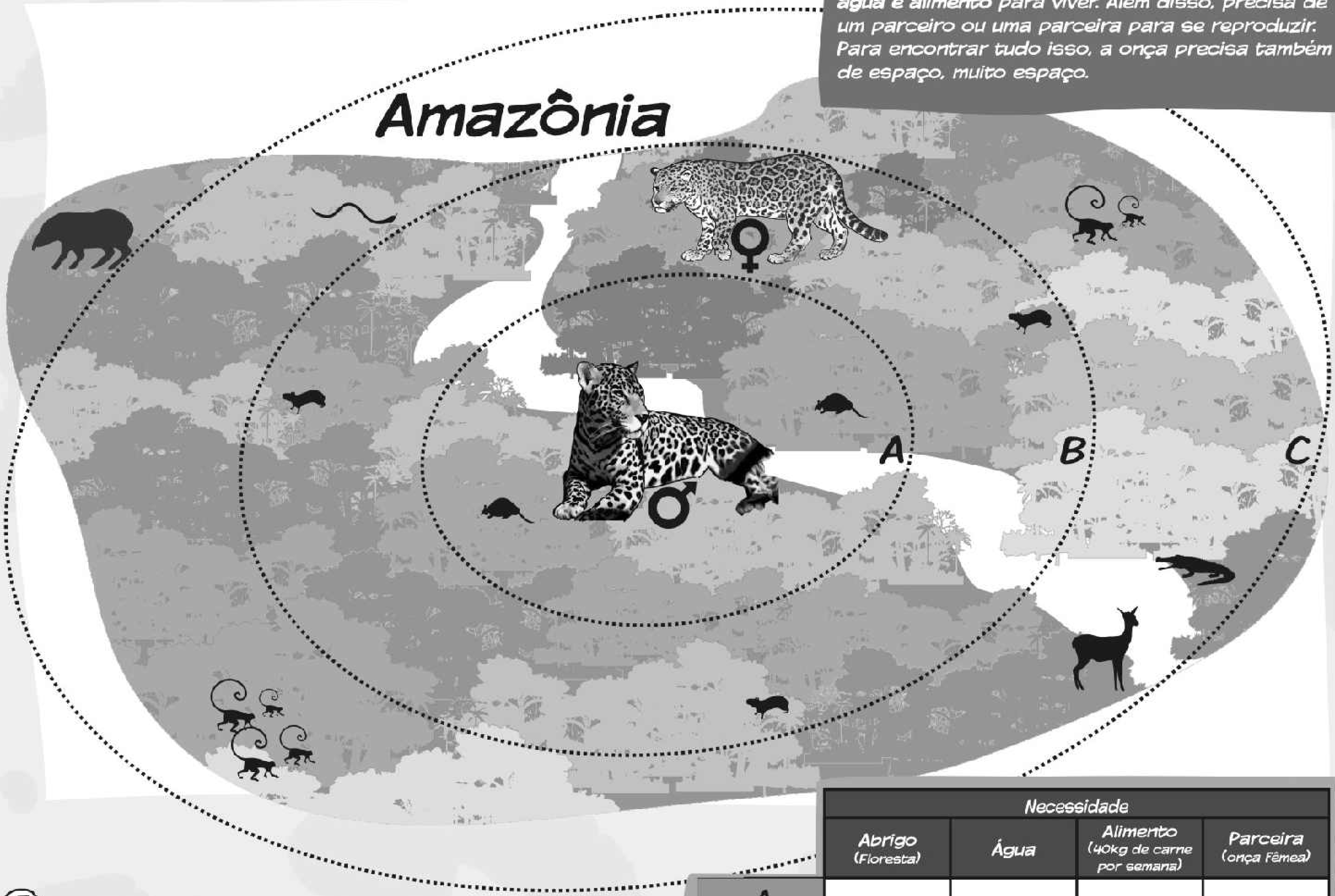


**7**

Descubra o tamanho da área que uma onça macho precisa para encontrar abrigo, água e alimento suficiente (40 quilos de carne por semana: use a tabela na página ao lado calcular o peso das presas dentro de cada área), além de fêmeas para namorar, na Amazônia.

| | Necessidade | | | |
|---|---|---|---|---|
| | Abrigo (Floresta) | Água | Alimento (40kg de carne por semana) | Parceira (onça fêmea) |
| A ($13km^2$) | | | | |
| B ($50km^2$) | | | | |
| C ($100km^2$) | | | | |

**Concluindo: uma onça macho precisa de ___$km^2$ para sobreviver na Amazônia.**

| Bicho: | Peso: |
|---|---|
| CAPIVARA: | 80 Kg |
| QUEIXADA: | 40Kg |
| PACA: | 10 Kg |
| TATÚ: | 10 Kg |
| JACARÉ: | 20Kg |

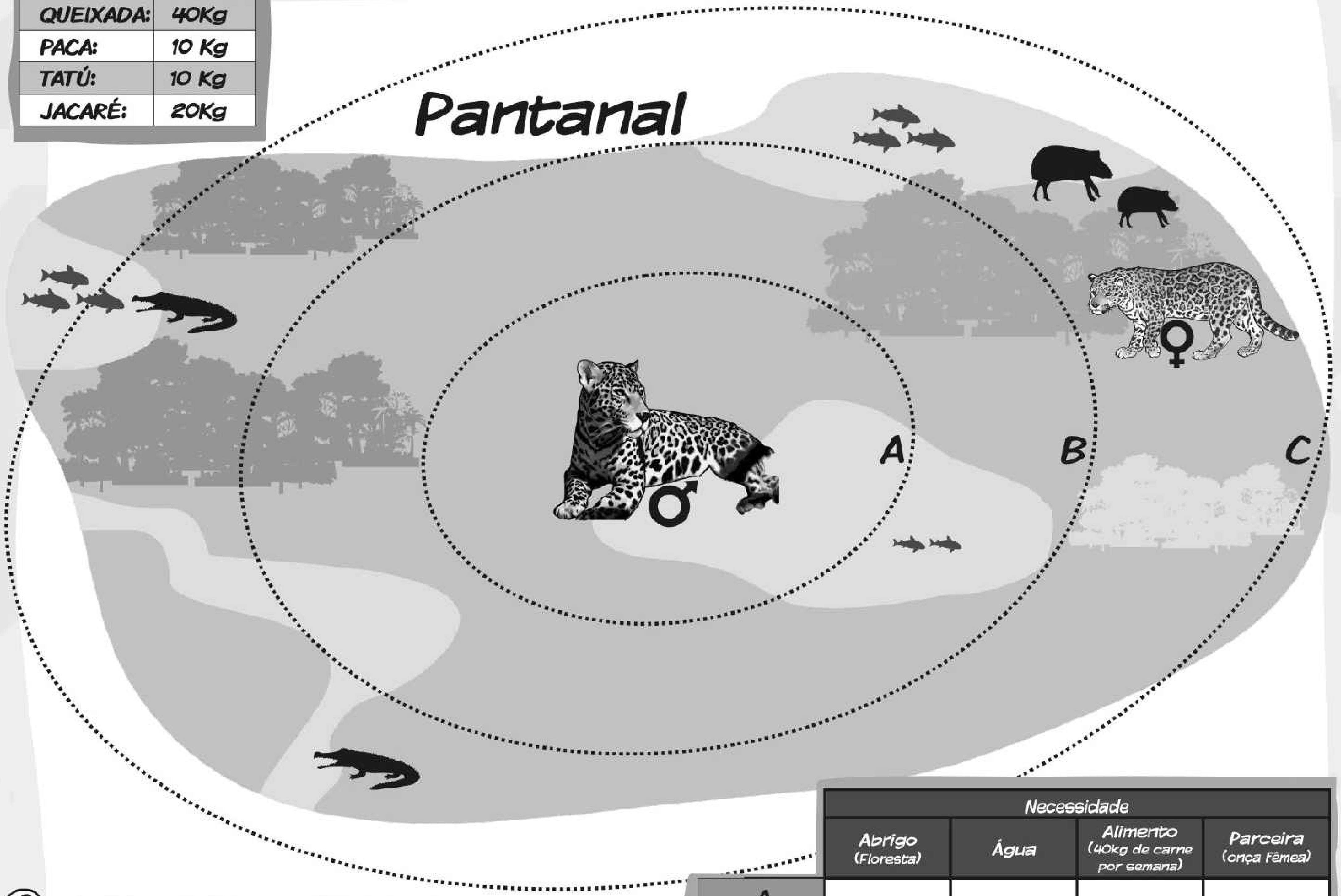


8 Agora faça a mesma coisa para o macho no Pantanal.

| | Necessidade | | | |
|---|---|---|---|---|
| | Abrigo (Floresta) | Água | Alimento (40kg de carne por semana) | Parceira (onça Fêmea) |
| A ($13km^2$) | | | | |
| B ($50km^2$) | | | | |
| C ($100km^2$) | | | | |

Concluindo: uma onça macho precisa de ___$km^2$ para sobreviver no Pantanal.

# ONÇAS-PINTADAS: SAIBA MAIS

## Onças-pintadas em livros.

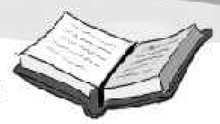

- **Guia de Convivência Gente e Onças,** de Silvio Marchini e Ricardo Luciano, 2008. Fundação Ecológica Cristalino e Wildlife Conservation Research Unit. Obra que serviu de referência para esse livro de atividades.

- Manual sobre Problemas de Predação do Gado por **Onças-Pintadas e Onças-Parda** em Fazendas de Gado, de Rafael Hoogesteijn. Wildlife Conservation Society, 2005. É uma referência importante para a resolução de problemas de ataque de onças ao gado.

- Manual de Identificação, Prevenção e Controle de Predação por Carnívoros, de Maria Renata Pereira Leite Pitman, Tadeu Gomes de Oliveira, Rogério Cunha de Paula e Cibele Incrusiak. Ibama, 2002. Outra referência importante para a resolução de problemas de predação por **onças**.

- Nas Selvas do Brasil, de Theodore Roosevelt. Editora Itatiaia. Livro de 1914, relata as viagens do autor pelas selvas e rios do Brasil em companhia de Cândido Rondon. Ele descreve suas caçadas de **onça** no Pantanal.

- El Jaguar en el Nuevo Milenio, da Wildlife Conservation Society em 2002. Reúne artigos científicos sobre **onças**. Em espanhol.

- El Jaguar, Tigre Americano, de Rafael Hoogestejin. Ediciones Armitano (Venezuela), 1992. Traz informações gerais sobre biologia, ecologia e conservação de **onças**, com foco nos problemas de predação do gado. Em espanhol.

- Jaguar, de Alan Rabinowitz. Editora Arbor House, 2000. Relato da criação de uma reserva para a conservação de **onças** em Belize e da fundação de uma longa história de pesquisa sobre a espécie no país, pelo próprio autor. Em inglês.

- Tigrero!, de Sasha Siemel. Um clássico de 1953, editado pela Prentice-Hall. Relato do ucraniano que viveu no Brasil nos anos 50, realizando safáris e caçando onças com •zaguaia (empalando-as) no Pantanal. Em inglês.
- Jaguar Hunting in the Matto Grosso and Bolivia, de Tony de Almeida. Editora Safari Press. Outro clássico e obra rara, escrito em 1976. Entre as estórias de caçadas no Pantanal, o autor aborda, pela primeira vez, detalhes sobre a ecologia da **onça-pintada**. Em inglês.

## Onças-pintadas em revistas

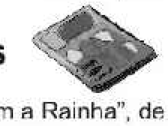

- **Revista Terra da Gente.** "Face a Face com a Rainha", de Helen Sacconi. Edição de Fevereiro de 2009.

- **Revista Terra.** "Com a onça na mira", com fotos e texto de Adriano Gambarini. Edição de Setembro de 2005.

- **National Geographic Magazine.** A famosa revista americana já publicou vários artigos sobre onças. Entre eles, destacam-se:
- **Path of the Jaguar**, de Mel White (Março de 2008)
- **Brazil' Wild Wet,** de Susan McGrath (Agosto de 2005)
- **Phantom of the Night**, de Douglas Chadwick (Maio de 2001)
- **Cats: Nature's Masterwork,** de Cathy Newman (Junho de 1977)
- **The Jungle was my Home,** de Sasha Siemel (Novembro de 1952)
- **King of Cats and his Court,** de Victor Cahalane (Fevereiro de 1943)

## Onças-pintadas na internet.

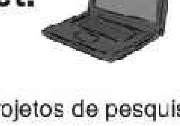

**Pró-carnívoros**
(http://www.procarnivoros.org.br).
Informação sobre carnívoros brasileiros: projetos de pesquisa e conservação, cursos, referências bibliográficas.

**Wikipedia**
(http://pt.wikipedia.org/wiki/On%C3%A7a-pintada).
Informação enciclopédica sobre a onça-pintada.

**National Geographic**
(http://animals.nationalgeographic.com/animals/mammals/jaguar.html).
Informações gerais, mapa de distribuição, multimídia.

**IUCN/Cat Specialist Group**
(http://lynx.uio.no/lynx/catsgportal/cat-website/20_catwebsite/home/index_en.htm).
Descrição da onça-pintada.

**Jaguar Conservation Network**
(http://www.jaguarnetwork.org).
Portal de informação sobre projetos de pesquisa, conservação e resolução de conflitos com onças-pintadas. Em inglês.

**Save the Jaguar**
(http://www.savethejaguar.com).
Informação geral sobre onças e projetos de pesquisa e conservação da Wildlife Conservation Society. Em inglês.

**Wildlife and People**
(http://www.peopleandwildlife.org.uk).
Portal sobre conflitos entre gente e fauna silvestre: projetos de pesquisa, eventos, referências bibliográficas, manuais de resolução de conflitos. Em inglês.

**Jaguar Conservation Fund**
(http://www.jaguar.org.br)
Informação sobre onças e projetos desenvolvidos pelo Instituto Onça-Pintada.

**Projeto Puma**
(http://uniplac.net/~puma/page_main_port3.html)
Informação sobre onça-parda e problemas de depredação associados à espécie.

## Onças-pintadas em vídeos e documentários

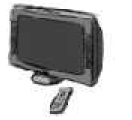

- YouTube (http://www.youtube.com).
  Possui vários vídeos que mostram **onças pintadas** em diversas situações. A qualidade dos vídeos é variável. Para encontrar vídeos de **onças** no YouTube, entre as palavras "**onça**" ou "**jaguar**" no buscador.

- National Geographic. O documentário "In Search of the Jaguar" pode ser adquirido na Amazon.com (http://www.amazon.com/National-Geographic-Search-Jaguar/dp/B000J4QW9U). Além disso, vários vídeos curtos estão disponíveis no site desta instituição: procure em http://video.nationalgeographic.com/video/player/animals/index.html

- Sasha Siemel, o Caçador de **Onças.** Documentário de Cândido Alberto Fonseca sobre o letoniano que caçava **onças** com zaguaia – uma espécie de lança - no Pantanal nos anos 50.

## Para ver e fotografar onças-pintadas.

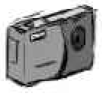

- ***Na natureza.*** **Onças** são extremamente difíceis de ver na natureza. Abundância relativamente baixa, hábitos arredios e silenciosos, e preferência por matas densas e por caminhar no escuro, fazem da **onça-pintada** uma das espécies menos avistadas da nossa fauna. As regiões do Brasil com infra-estrutura turística e melhores chances de ver **onças** são o rio Araguaia, o Pantanal Norte (especialmente na segunda metade da Rodovia Transpantaneira e ao longo do rio Cuiabá em Poconé e rio Paraguai em Cáceres) e Pantanal Sul (nos hotéis-fazenda e rios de Miranda). **Atenção:** *caso tenha o privilégio de avistar uma onça, mantenha-se sempre a uma distância segura dela.*

- ***Com "armadilhas fotográficas".*** Armadilhas fotográficas são câmeras fotográficas acopladas a sensores de infravermelho que disparam automaticamente com a aproximação de um animal de sangue quente. Instaladas ao longo de estradas e trilhas, elas fazem o trabalho de esperar por dias pela passagem de uma **onça** a ser fotografada, enquanto você fica em casa. As câmeras podem ser convencionais ou digitais. Nesse último caso, elas podem ser programadas para filmar animais que passam a sua frente, em lugar de fotografar. As marcas mais importantes de armadilhas fotográficas são as americanas Camtrakker (**www.camtrakker.com**) e as nacionais Tigrinus (**www.tigrinus.com.br**).

- ***Em cativeiro.*** Segundo o CENAP[1], existem aproximadamente 160 **onças-pintadas** em cativeiro no Brasil, a maioria delas em zoológicos abertos à visitação pública. Portanto, se você quer ver uma **onça** ao vivo, a maneira mais prática e garantida de fazê-lo é visitando o zôo mais perto de você. Veja a seguir a lista de zoológicos que abrigam **onças-pintadas.**

# ONÇAS-PINTADAS: ONDE ENCONTRAR?

Criadouro da Itaipu Binacional em Foz do Iguaçu (PR)
Criadouro Luciano Sabóia em Curitiba (PR)
Fundação Zoobotânica de Belo Horizonte (MG)
Parque Ambiental Chico Mendes em Rio Branco (AC)
Zôo da Santur em Balneário Camboriú (SC)
Zôo de Americana (SP)
Zôo de Aracaju (SE)
Zôo de Araçatuba (SP)
Zôo de Bauru (SP)
Zôo de Brasília (DF)
Zôo de Brusque (SC)
Zôo de Cachoeira do Sul (RS)
Zôo de Campinas (SP)
Zôo de Cascavel (PR)
Zôo de Catanduva (SP)
Zôo de Curitiba (PR)
Zôo de Foz do Iguaçu (PR)
Zôo de Goiânia (GO)
Zôo de Guarulhos (SP)
Zôo de Ilha Solteira (SP)
Zôo de Ipatinga (MG)
Zôo de João Pessoa (PB)
Zôo de Leme (SP)
Zôo de Limeira (SP)
Zôo de Lins (SP)
Zôo de Pedreira (SP)
Zôo de Piracicaba (SP)
Zôo de Pomerode (SC)
Zôo de Pouso Alegre (MG)
Zoo de Recife (PE)
Zôo de Ribeirão Preto (SP)
Zôo de Rio Preto (SP)
Zôo de São Carlos (SP)
Zôo de São Paulo (SP)
Zôo de São Vicente (SP)
Zôo de Sapucaia (SP)
Zôo de Sorocaba (SP)
Zôo de Taboão da Serra (SP)
Zôo de Teresina (PI)
Zôo da UFMT em Cuiabá (MT)
Zôo do Hotel Fazenda Mato Grosso
Zôo de Varginha (MG)
Zôo de Volta Redonda (RJ)
Zôo do Centro de Instrução de Guerra na Selva em Manaus (AM)
Zôo do Hotel Tropical em Manaus (AM)
Zôo do Museu Paraense Emílio Goeldi em Belém (PA)
Zôo do Rio de Janeiro (RJ)
Zôo Irmão Ézio Danza e Silva em Poços de Caldas (MG)

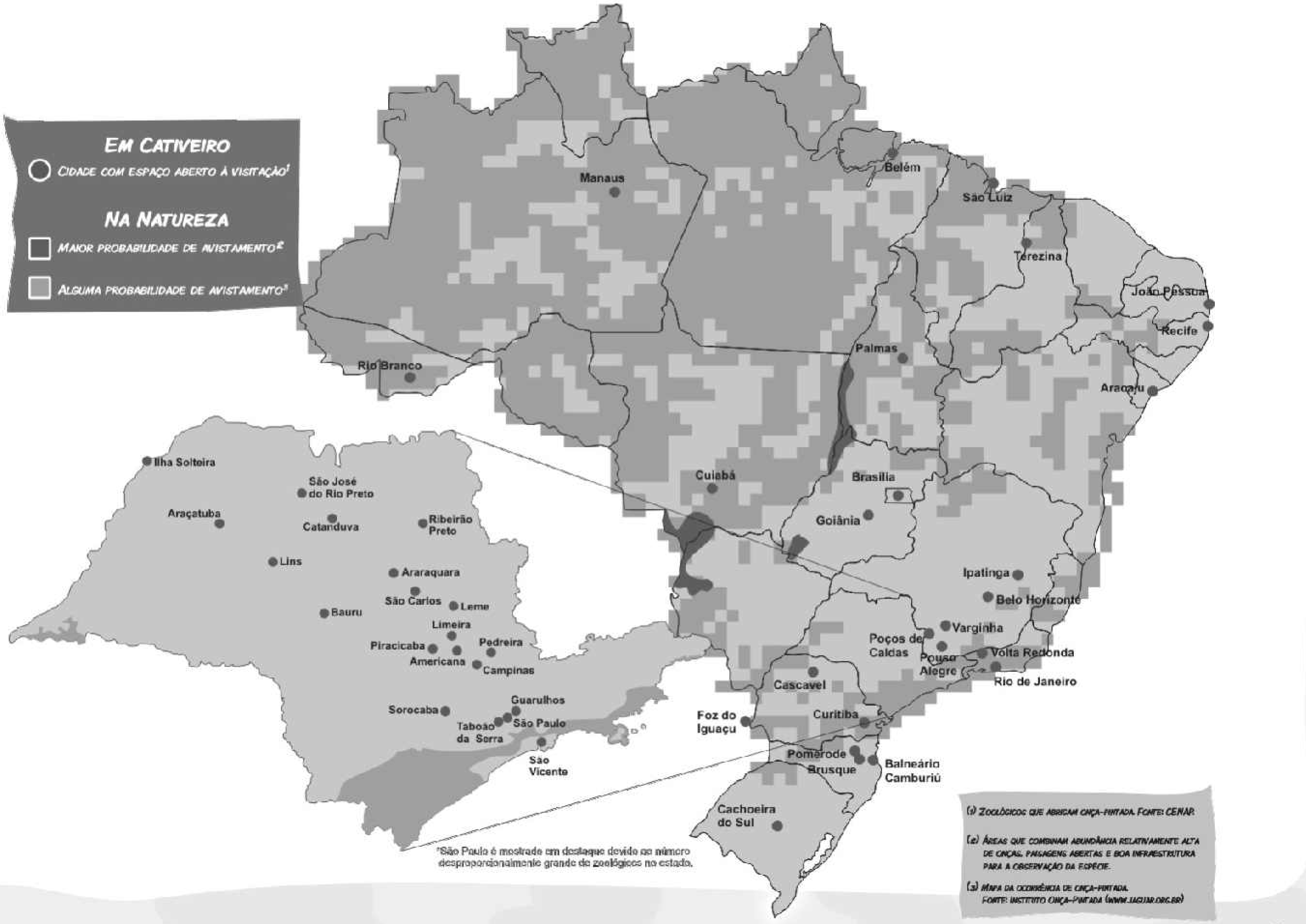


*São Paulo é mostrado em destaque devido ao número desproporcionalmente grande de zoológicos no estado.

(1) Zoológicos que abrigam onça-pintada. Fonte: CENAP.

(2) Áreas que combinam abundância relativamente alta de onças, paisagens abertas e boa infraestrutura para a observação da espécie.

(3) Mapa da ocorrência de onça-pintada. Fonte: Instituto Onça-Pintada (www.jaguar.org.br)

CENAP: Centro Nacional de Pesquisas para a Conservação de Predadores Naturais.
http://www.procarnivoros.org.br/cenapibama.php

Agradecemos a todos aqueles que de alguma forma contribuíram para que esse guia fosse concluído. Entre eles, agradecemos especialmente a Vitória da Riva, Renato Farias, Edson Grandisoli, Rafael Hoogesteijn, Peter Crawshaw, Ronaldo Gonçalves Morato, Tathiana Bagatini de Alcântara, Leandro Silveira, Natália Mundim Torres, Renata Leite Pitman e Fernanda Michalski. Agradecemos também aos apoiadores do Projeto Conviver Gente e Onças, em especial a Kevin Duncan.

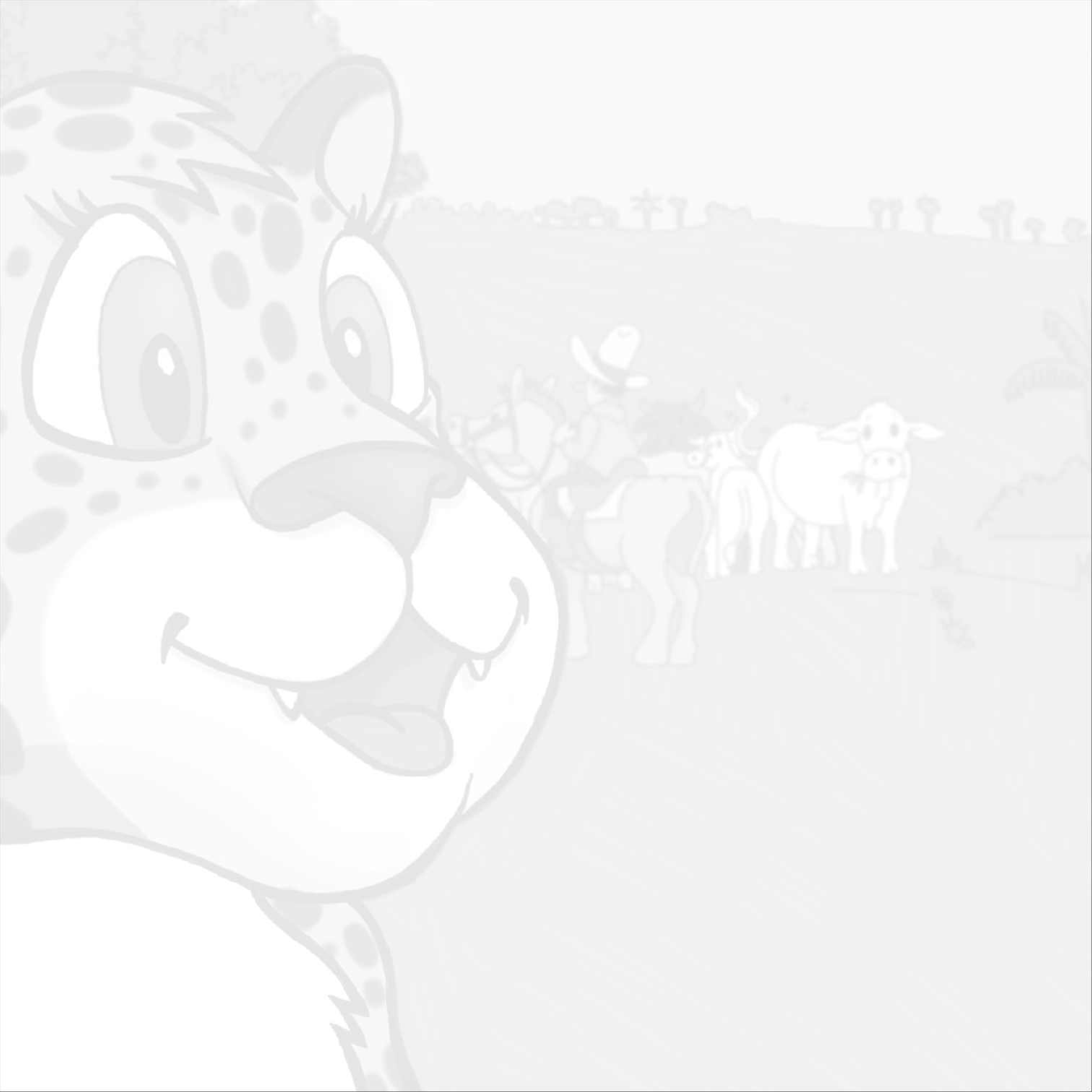

O **Projeto Conviver Gente & Onças** é uma realização da **Fundação Ecológica Cristalino (FEC)** e da **Wildlife Conservation Research Unit (WildCRU)**.

A **FEC** foi fundada em 1999 com a missão de promover a conservação da bio-diversidade na fronteira agrícola da Amazônia no norte do Mato Grosso e, em particular, na área de entorno do **Parque Estadual Cristalino**. Ela tem quatro frentes principais de atuação: políticas públicas, áreas protegidas, pesquisa e educação. As atividades de educação são desenvolvidas pela **Escola da Amazônia**. Além de campanhas de informação e sensibilização sobre onças-pintadas, a Escola da Amazônia realiza oficinas para crianças, jovens e educadores sobre temas relacionados à conservação da bio-diversidade.

Fundação Ecológica Cristalino

www.fundacaocristalino.org.br

Silvio Marchini
Ricardo Luciano

LIVRO DE ATIVIDADES
GENTE E ONÇAS:
CONFLITO E CONVIVÊNCIA

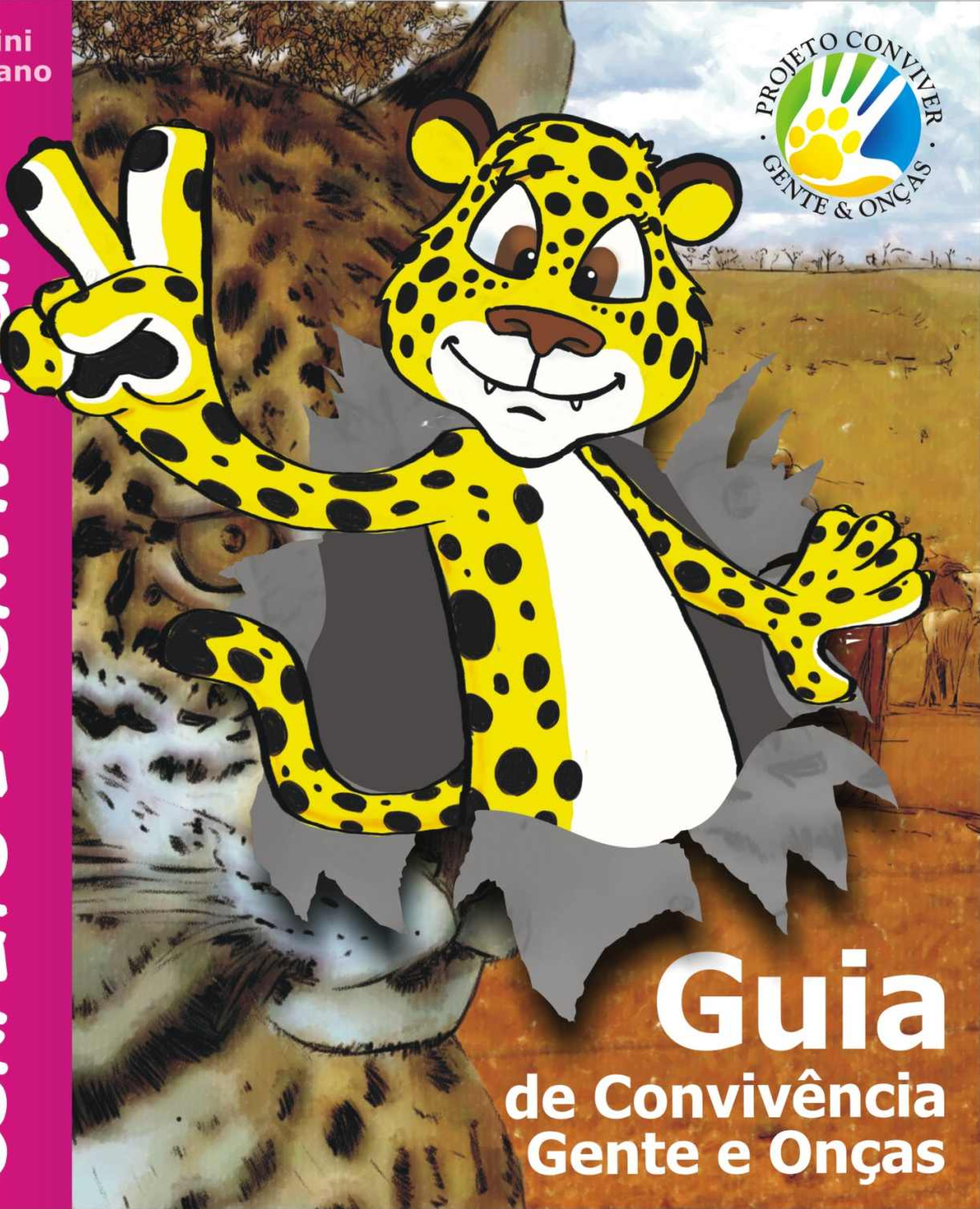


# Guia
## de Convivência Gente e Onças

Fundação Ecológica Cristalino

**Presidente**
Vitoria da Riva Carvalho

**Diretor Executivo**
Renato Aparecido de Farias

**ISBN**: 000-00-000000-0-0

Copyright © 2008 by Editora Amazonarium Ltda

**Autor:** © Silvio Marchini
**Ilustrador:** © Ricardo Luciano
**Páginas:** 24

Impresso no Brasil

Todos os direitos reservados. Nenhuma parte deste livro pode ser reproduzida ou utilizada por qualquer meio, sem prévia autorização por escrito da editora.

**Projeto gráfico e diagramação:**
Ricardo Luciano

# COLEÇÃO GUIA DE CONVIVÊNCIA

## Livro de Atividades II

# GENTE E ONÇAS: CONFLITO E CONVIVÊNCIA

Texto
**Silvio Marchini**

Ilustração
**Ricardo Luciano**

Uma realização
**Fundação Ecológica Cristalino**
**Wildlife Conservation Research Unit**

**1ª Edição**

Alta Floresta, Mato Grosso, Brasil
Maio de 2009

# Coleção guias de convivência

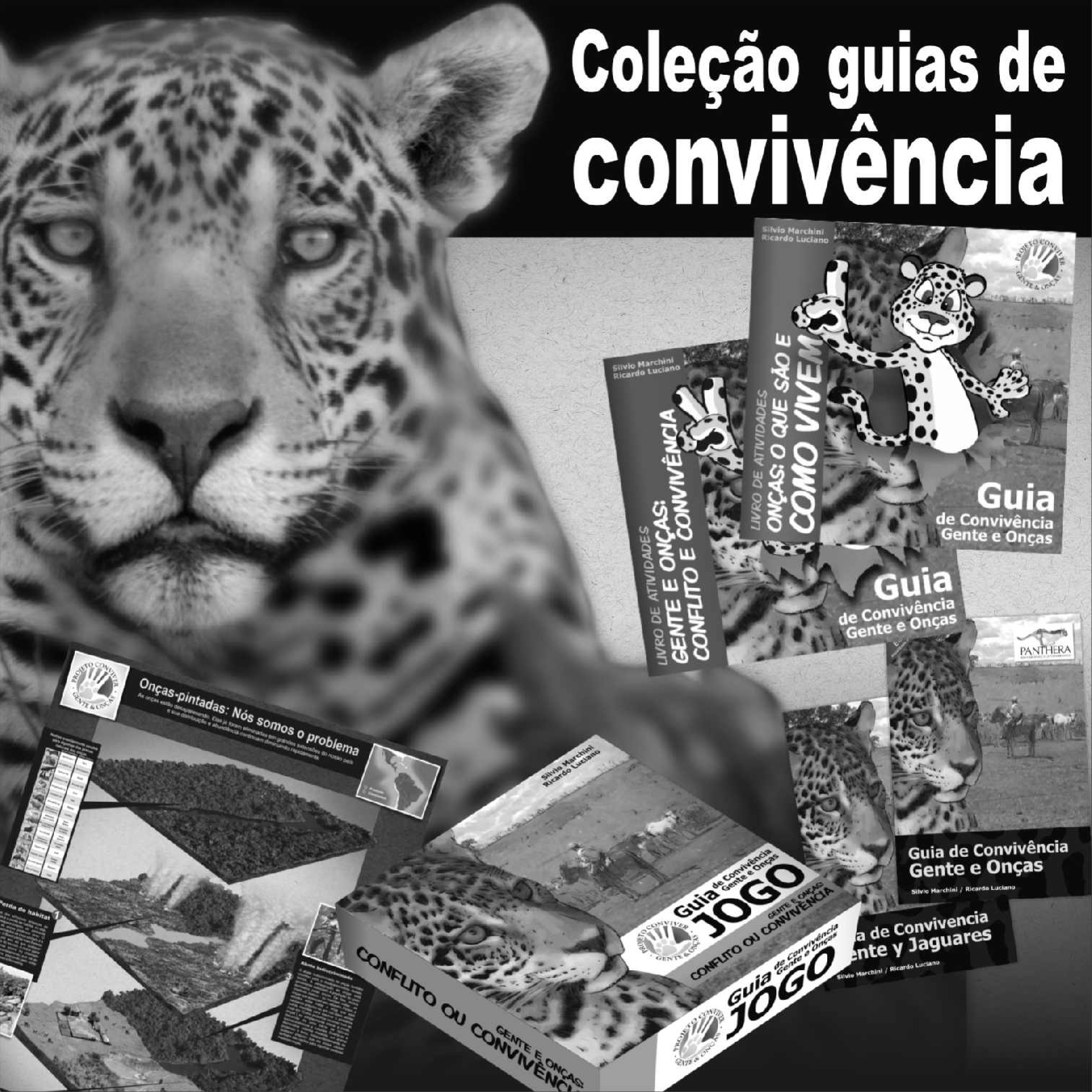

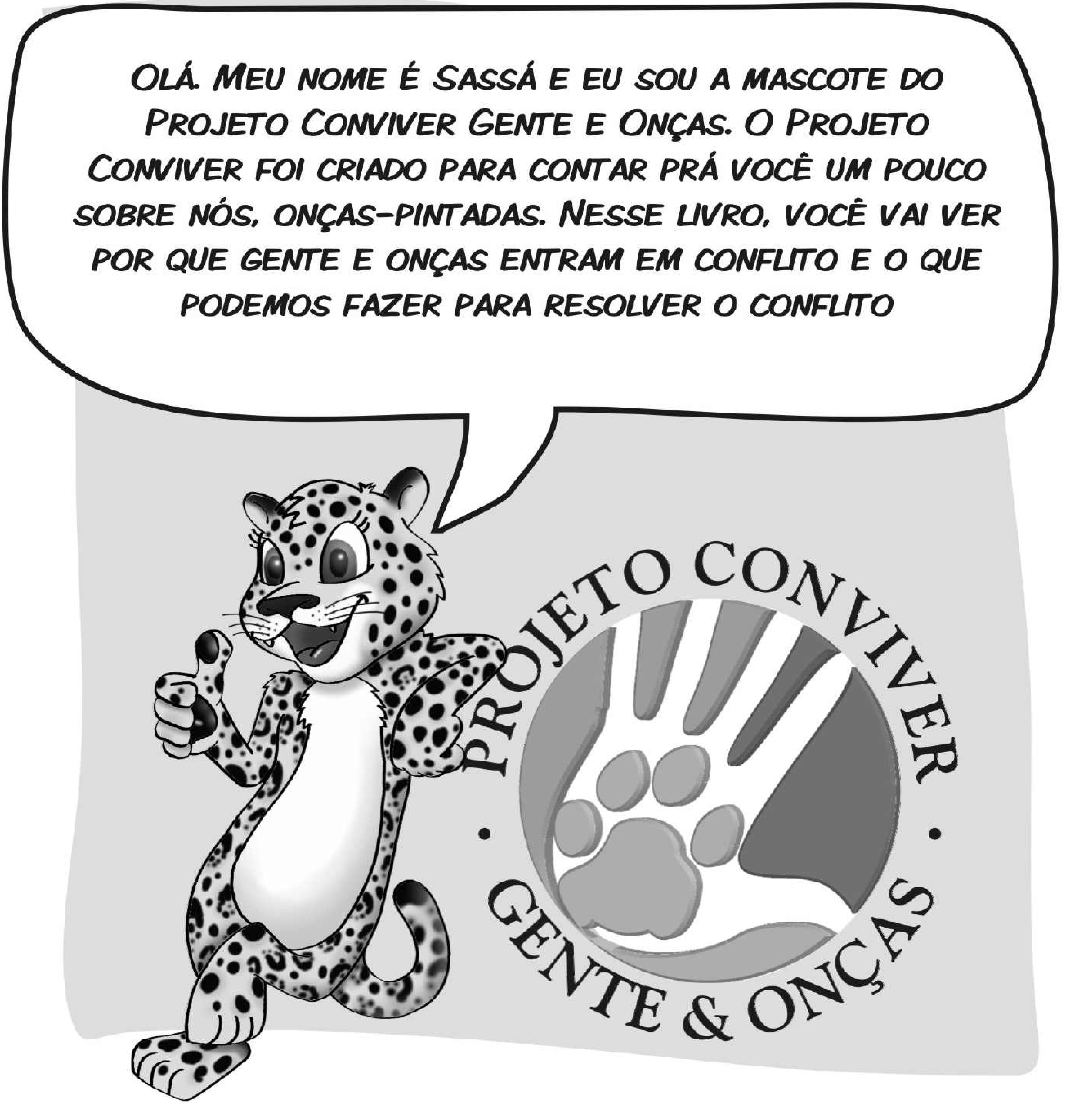
OLÁ. MEU NOME É SASSÁ E EU SOU A MASCOTE DO PROJETO CONVIVER GENTE E ONÇAS. O PROJETO CONVIVER FOI CRIADO PARA CONTAR PRÁ VOCÊ UM POUCO SOBRE NÓS, ONÇAS-PINTADAS. NESSE LIVRO, VOCÊ VAI VER POR QUE GENTE E ONÇAS ENTRAM EM CONFLITO E O QUE PODEMOS FAZER PARA RESOLVER O CONFLITO
PROJETO CONVIVER
GENTE & ONÇAS

# ONÇAS-PINTADAS: CONFLITO E CONVIVÊNCIA

VOCÊ TEM MEDO DE ONÇA-PINTADA? SE RESPONDEU "SIM", VOCÊ NÃO ESTÁ SOZINHO OU SOZINHA, POIS A MAIORIA DAS PESSOAS TEM MEDO DE ONÇA. ALIÁS, O MEDO É UMA DAS PRINCIPAIS CAUSAS DO CONFLITO ENTRE GENTE E ONÇAS. MAS SAIBA QUE SE VOCÊ NÃO MEXER COM A ONÇA, O PERIGO DELA PEGAR VOCÊ É INSIGNIFICANTE. PRATICAMENTE TODAS AS PESSOAS ATACADAS POR ONÇAS ESTAVAM, DE ALGUMA FORMA, MEXENDO COM ELA.

1. Marque com X as situações em que existe o risco da onça atacar o ser humano.

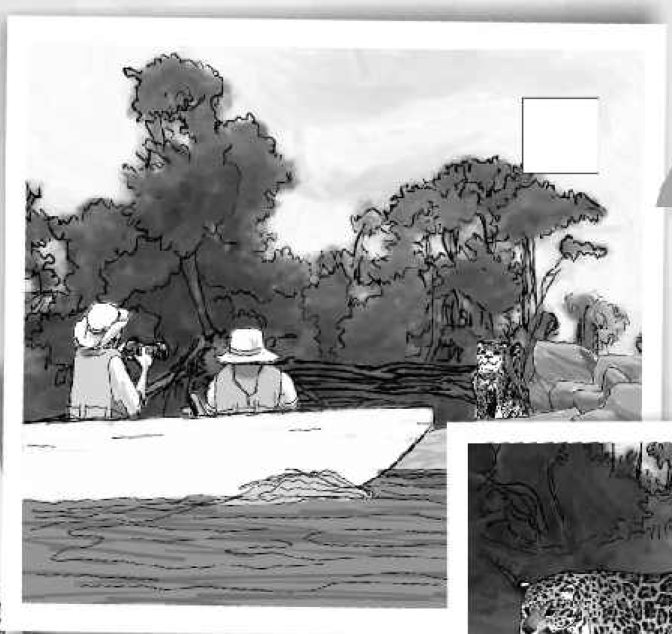

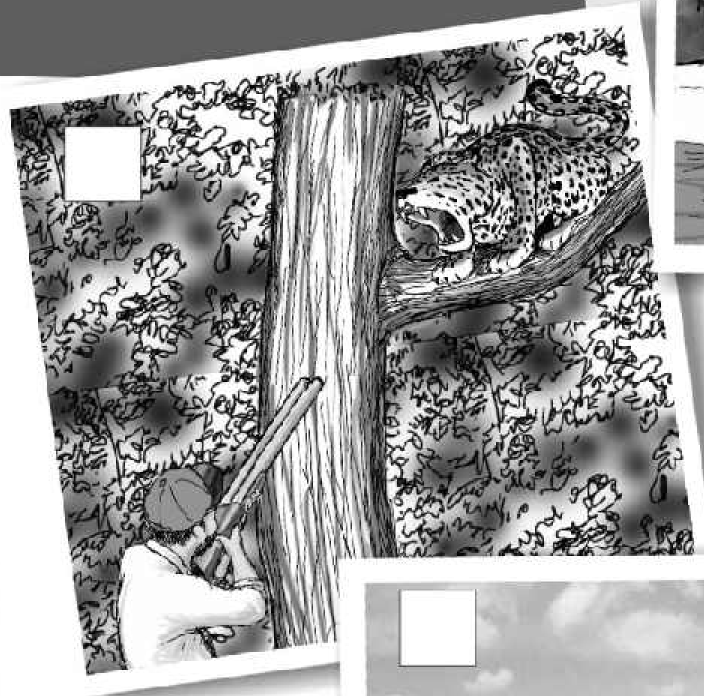

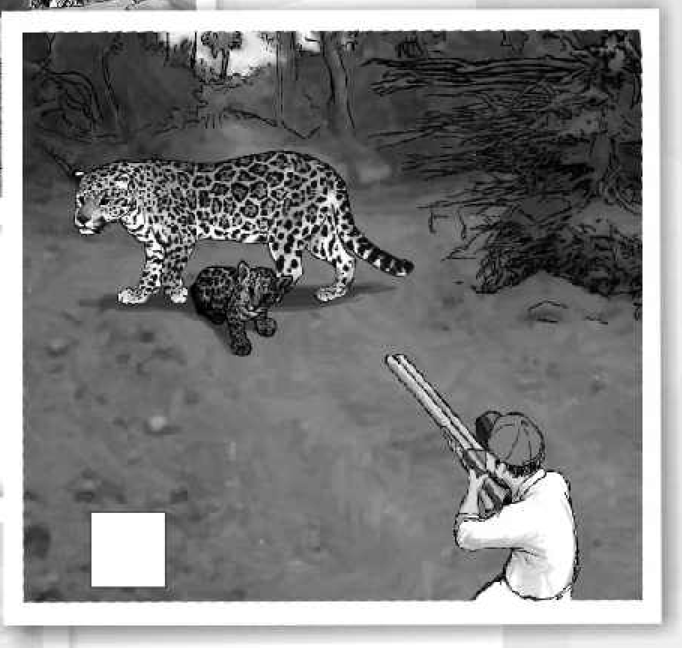

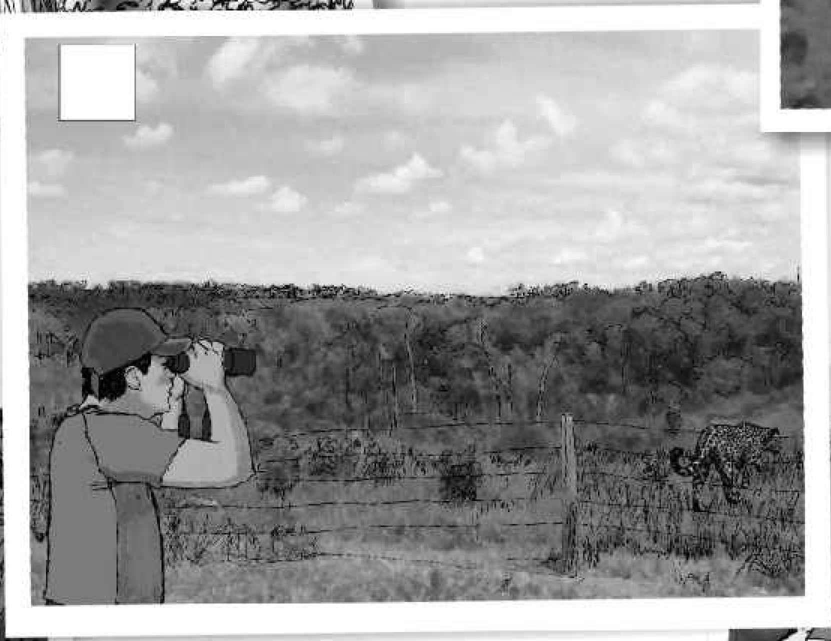

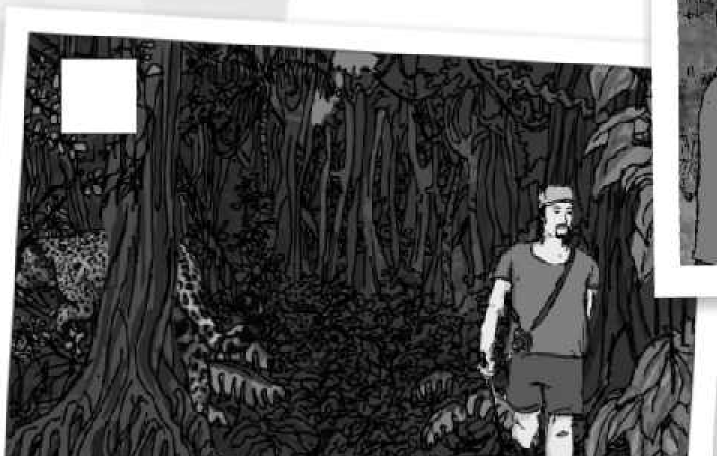

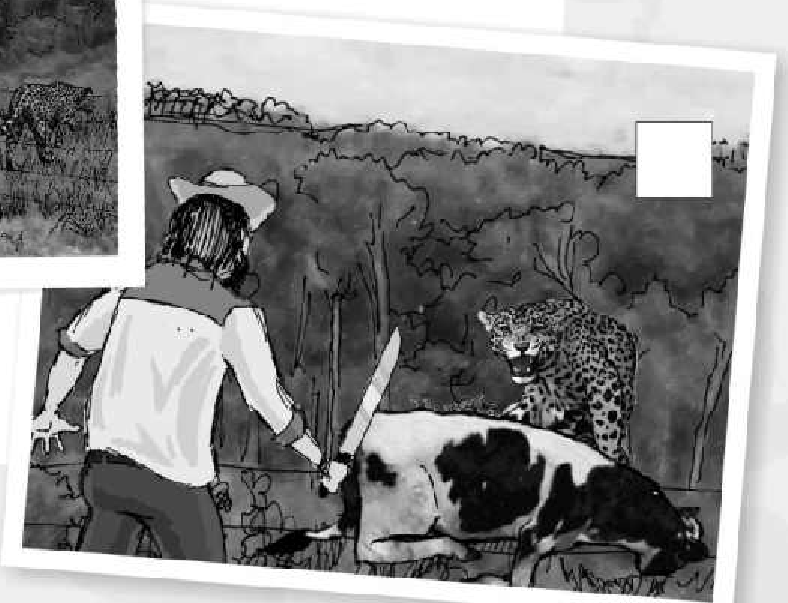

Marque com X no que você faria se desse de cara com uma onça-pintada: ②

ALÉM DISSO, AS ONÇAS SÃO GERALMENTE ARREDIAS E EVITAM A PRESENÇA DE GENTE, SEM CONTAR QUE, COMPARADA COM A MAIORIA DOS OUTROS ANIMAIS, AS ONÇAS EXISTEM EM BAIXA DENSIDADE (OU SEJA, UMA AQUI E A PRÓXIMA A QUILÔMETROS DE DISTÂNCIA). POR ISSO, AS CHANCES DE UMA ONÇA-PINTADA CRUZAR SEU CAMINHO SÃO MUITO PEQUENAS. MAS SE ALGUM DIA POR ACASO VOCÊ SE DEPARASSE COM UMA ONÇA, O QUE VOCÊ FARIA?

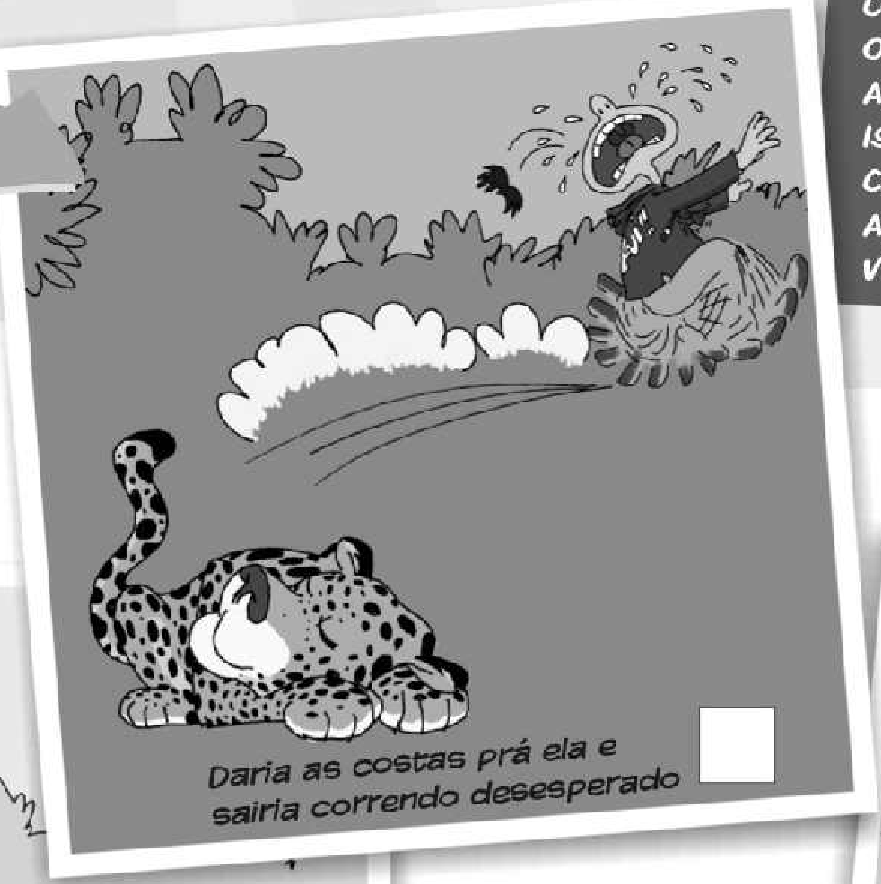

Daria as costas prá ela e sairia correndo desesperado

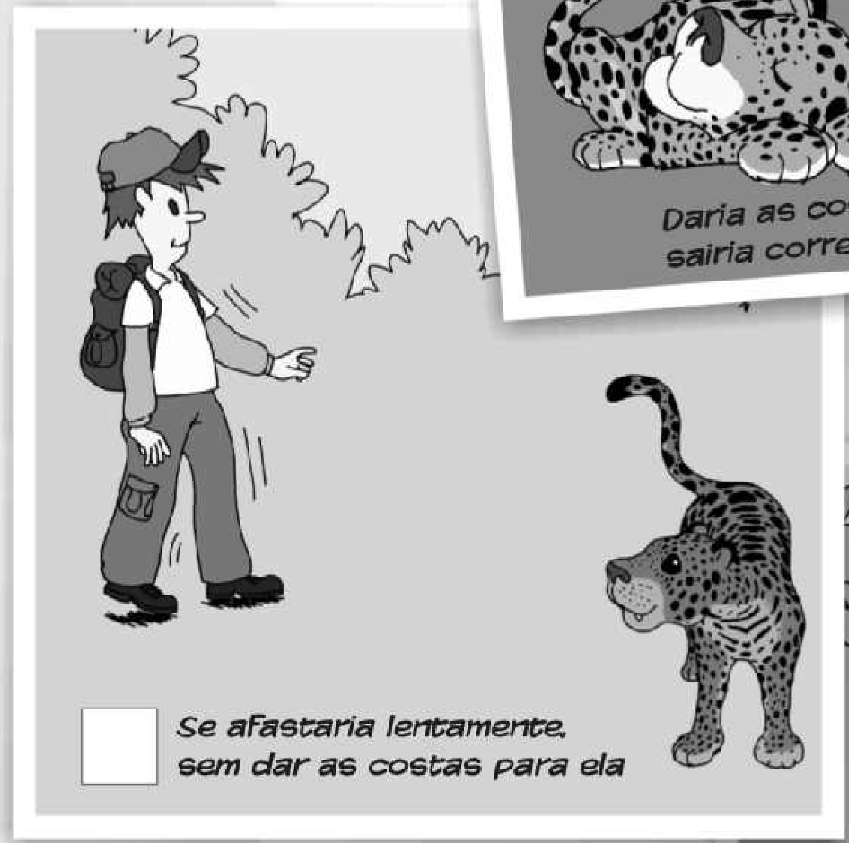

Se afastaria lentamente, sem dar as costas para ela

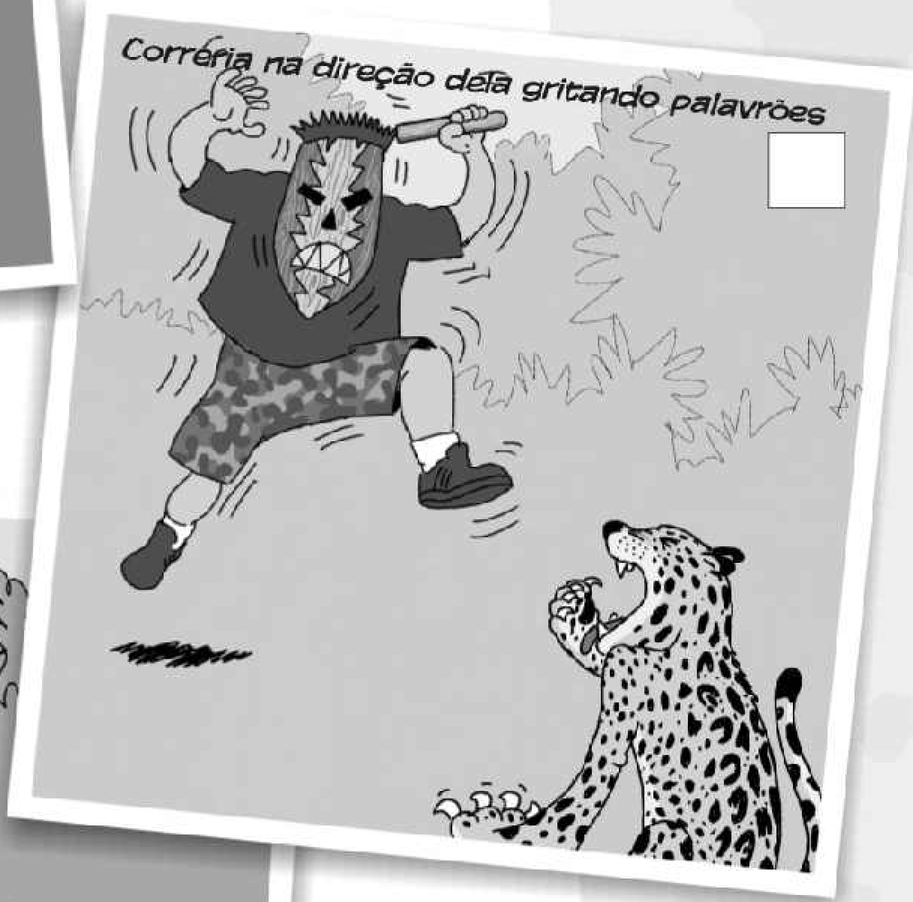

Correria na direção dela gritando palavrões

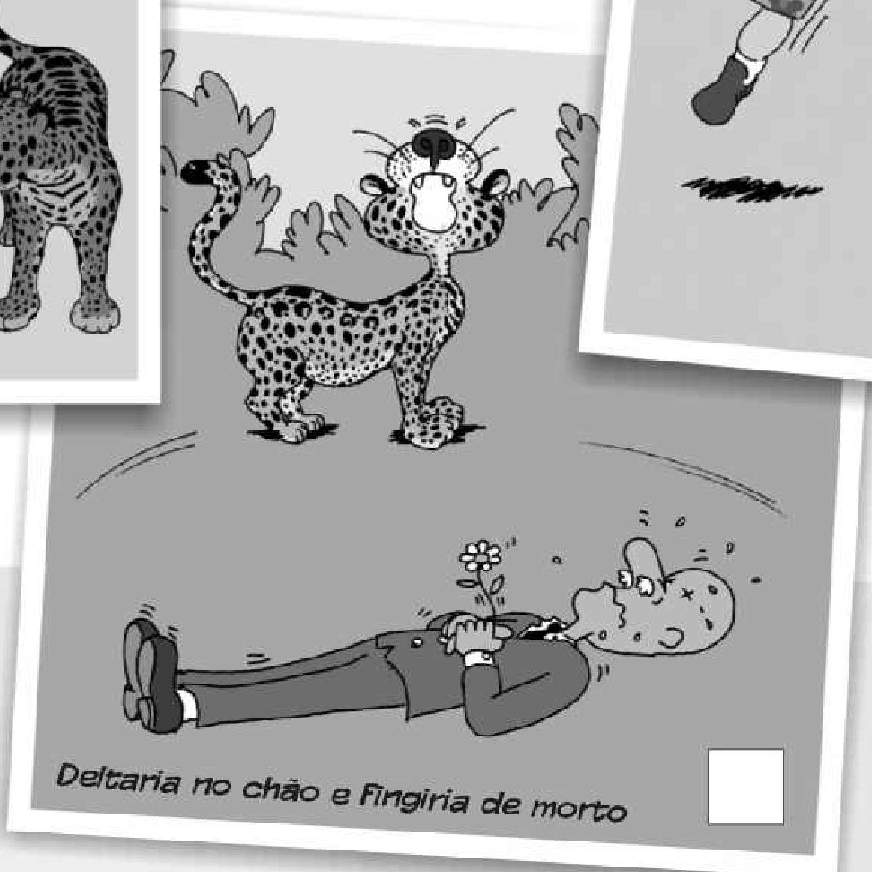

Deitaria no chão e fingiria de morto

Veja as respostas certas e O QUE NÃO FAZER em um encontro com uma onça na página 22

# ONÇAS-PINTADAS: CONFLITO E CONVIVÊNCIA

Ligue a Figura ao texto correspondentes (3)

ANTES DE SE PREOCUPAR COM O RISCO DE SER ATACADO POR UMA ONÇA-PINTADA, VEJA COMO ALGUNS OUTROS BICHOS OFERECEM MUITO MAIS PERIGO PARA O SER HUMANO.

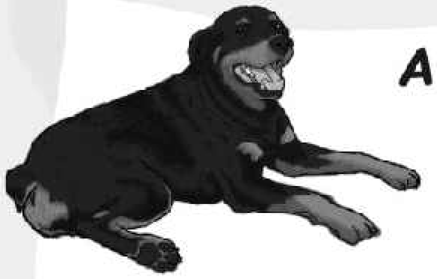

A

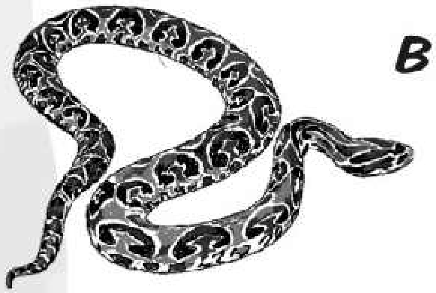

B

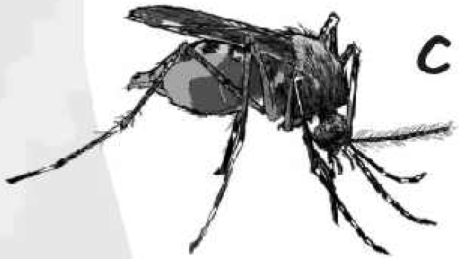

C

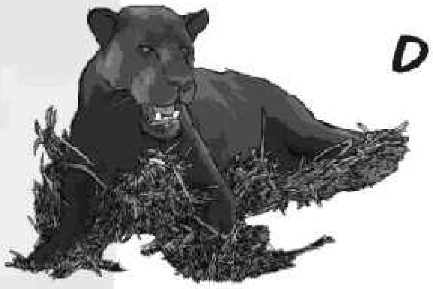

D

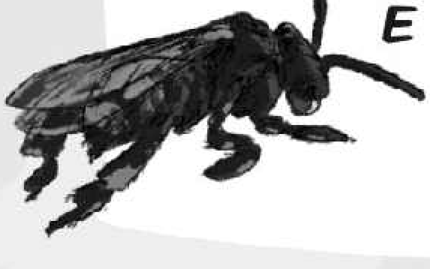

E

☐ TRANSMITEM DOENÇAS INFECCIOSAS, ENTRE ELAS DENGUE, LEISHMANIOSE, FEBRE AMARELA E MALÁRIA. MATAM MILHÕES DE PESSOAS NO MUNDO TODOS OS ANOS.

☐ PODEM SER ÚTEIS POR PRODUZIREM ALIMENTO. UMA DELAS SOZINHA OFERECE POUCO PERIGO, MAS COMO GERALMENTE VIVEM EM COLÔNIAS DE DEZENAS A CENTENAS DE INDIVÍDUOS, UM ATAQUE MACIÇO PODE SER FATAL. MATAM CENTENAS DE PESSOAS NO MUNDO TODOS OS ANOS.

☐ SÃO CONHECIDOS COMO "O MELHOR AMIGO DO HOMEM". MAS NEM TODOS SÃO TÃO AMIGÁVEIS. NA VERDADE, ALGUNS SÃO BRAVOS O SUFICIENTE PARA ATACAR E MATAR O SER HUMANO. ELES MATAM, EM MÉDIA, 150 PESSOAS POR ANO NO MUNDO.

☐ ELAS SEMPRE FORAM TEMIDAS. ATÉ NA BÍBLIA UMA DELAS APARECE COMO A PRIMEIRA VILÃ DA HISTÓRIA. EMBORA A MAIORIA DAS ESPÉCIAS É INOFENSIVA, ALGUMAS POSSUEM VENENOS FATAIS. CAUSAM MILHARES DE MORTES POR ANO NO MUNDO.

☐ APESAR DA FAMA DE MÁ E DE SER UMA PREDADORA SUPER BEM ARMADA, ELA É TÍMIDA E PREFERE EVITAR O CONFRONTO COM O HOMEM. ATACA PARA SE DEFENDER OU DEFENDER SEUS FILHOTES. NO BRASIL, EXISTE APENAS UM ATAQUE FATAL CONHECIDO.

OUTRA RAZÃO PELA QUAL MUITA GENTE NÃO QUER VER ONÇAS POR PERTO É O PREJUÍZO QUE ELAS CAUSAM AO MATAR ANIMAIS DOMÉSTICOS. A ONÇA É UM PREDADOR OPORTUNISTA, OU SEJA, ELA COME AQUILO QUE TEM A OPORTUNIDADE DE COMER. QUANDO FALTA QUEIXADAS, CAPIVARAS, JACARÉS E TATÚS MAS SOBRA VACAS E BEZERROS, ORA, ELA VAI COMER VACAS E BEZERROS.

4

INDIQUE COM X OS ANIMAIS QUE SERVEM DE ALIMENTO PARA A ONÇA-PINTADA.

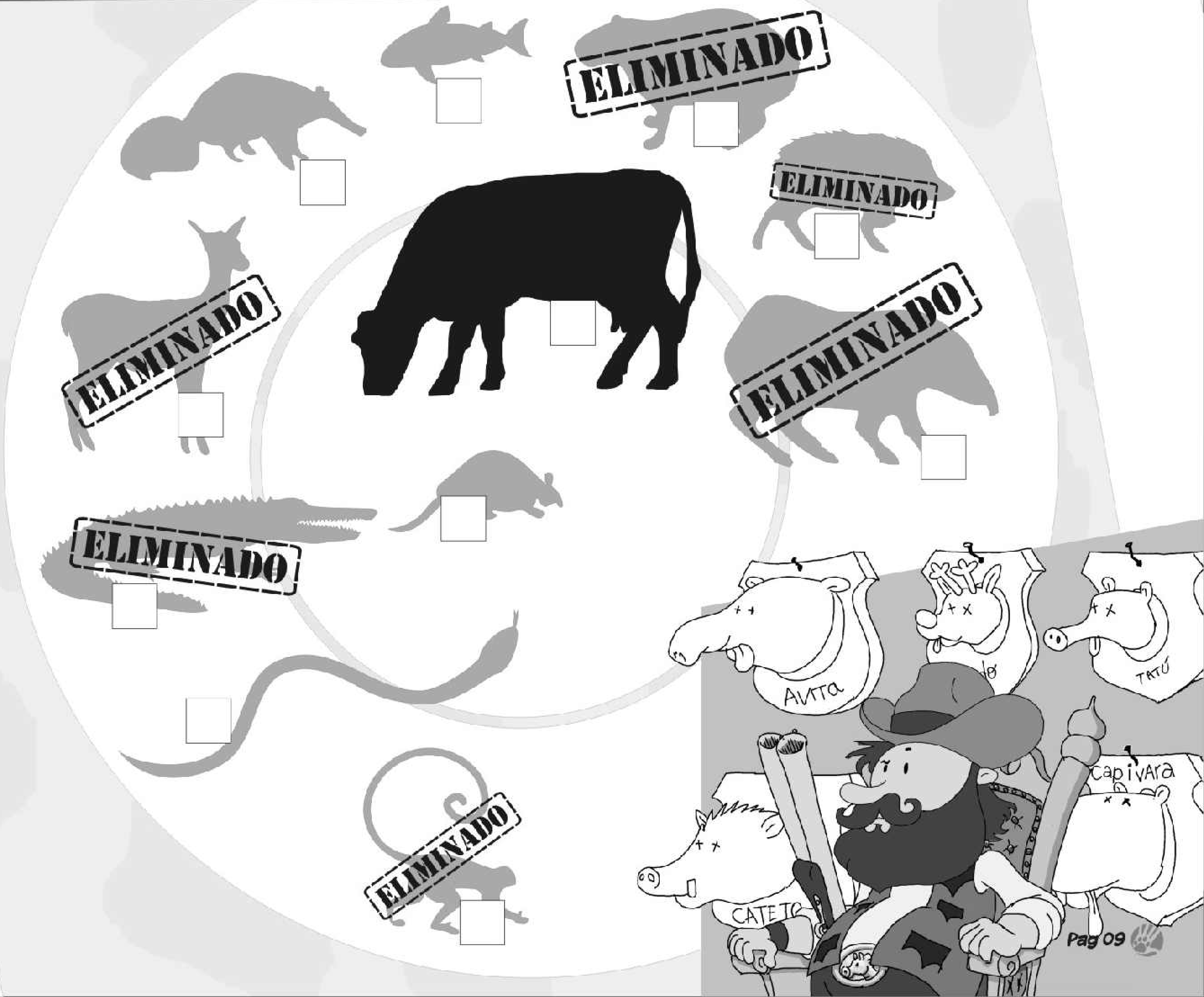

# ONÇAS-PINTADAS: CONFLITO E CONVIVÊNCIA

EMBORA EM ALGUNS CASOS AS ONÇAS CAUSEM GRANDE PREJUÍZO AO PRODUTOR RURAL, NA AMAZÔNIA E NO PANTANAL AS ONÇAS MATAM EM MÉDIA 1% DO REBANHO (1 A CADA 100).



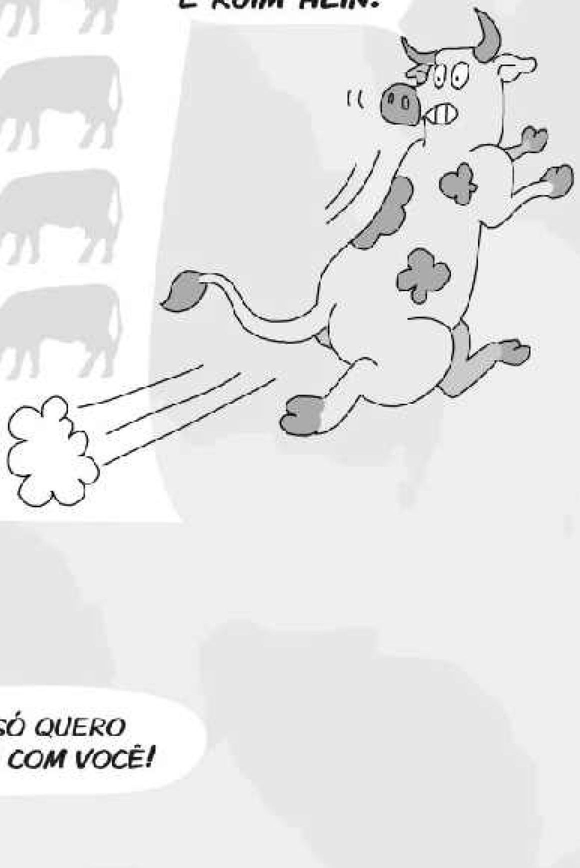


**5** SE UMA A CADA 100 VACAS É COMIDA POR ONÇA, QUANTAS SÃO COMIDAS DE UM REBANHO DE:

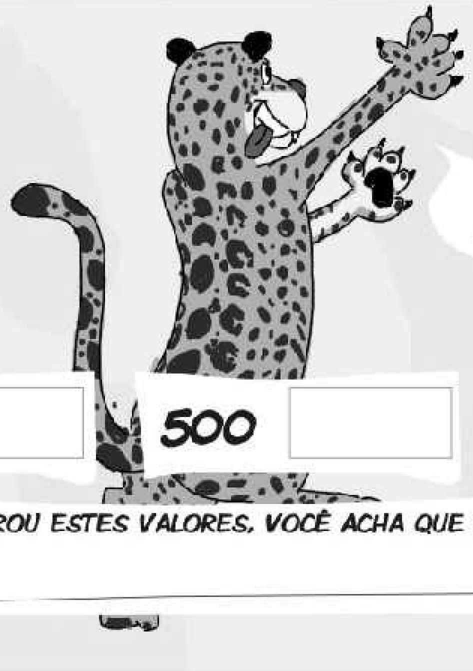


| | | | | | | | | | | | |
|---|---|---|---|---|---|---|---|---|---|---|---|
| 50 | | 100 | | 250 | | 500 | | 1500 | | 2000 | |

AGORA QUE VOCÊ ENCONTROU ESTES VALORES, VOCÊ ACHA QUE VALE A PENA MATAR UMA ONÇA POR ISSO? POR QUE?

R: ______________________________

MESMO QUE UMA VACA EM CEM POSSA PARECER BASTANTE PRA VOCÊ, SAIBA QUE VACAS E BEZERROS MORREM POR OUTRAS RAZÕES TAMBÉM: DOENÇAS, DESNUTRIÇÃO, PROBLEMAS DE PARTO, AFOGAMENTO E PICADAS DE COBRA PODEM MATAR MAIS VACAS E BEZERROS DO QUE ATAQUES DE ONÇAS.

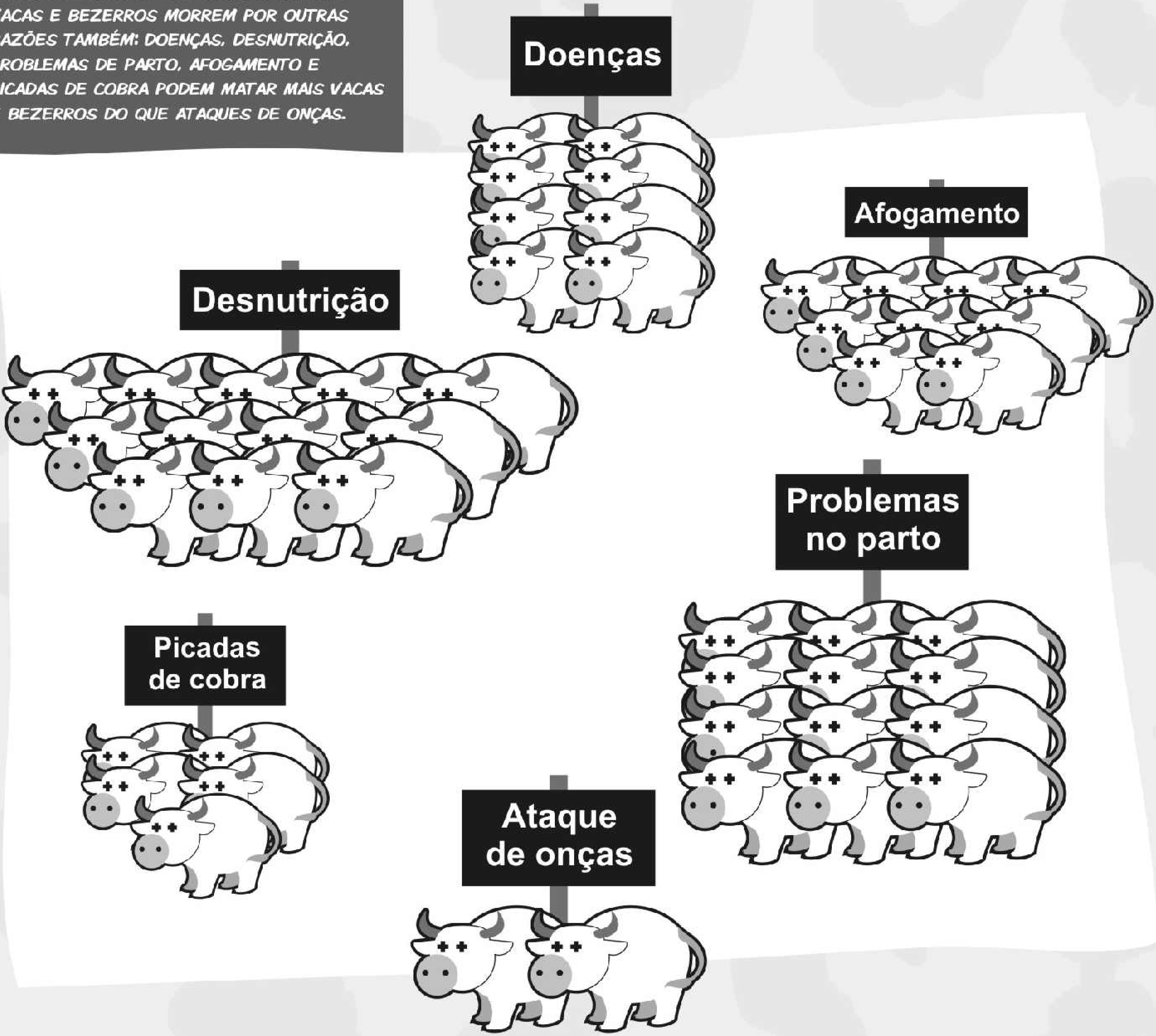

# ONÇAS-PINTADAS: CONFLITO E CONVIVÊNCIA

MUITO MAIOR DO QUE O PROBLEMA QUE AS ONÇAS CAUSAM AO SER HUMANO, É O PROBLEMA QUE O SER HUMANO CAUSAM ÀS ONÇAS. NÓS HUMANOS ESTAMOS DESTRUINDO O ABRIGO DAS ONÇAS, OU SEJA, DESMATANDO NOSSAS FLORESTAS E CERRADOS, NÓS ESTAMOS CAÇANDO OS BICHOS QUE SERVEM DE ALIMENTO PARA AS ONÇAS (EXEMPLO: ANTAS, CAITITUS E VEADOS) E, PRINCIPALMENTE, ESTAMOS MATANDO AS ONÇAS.

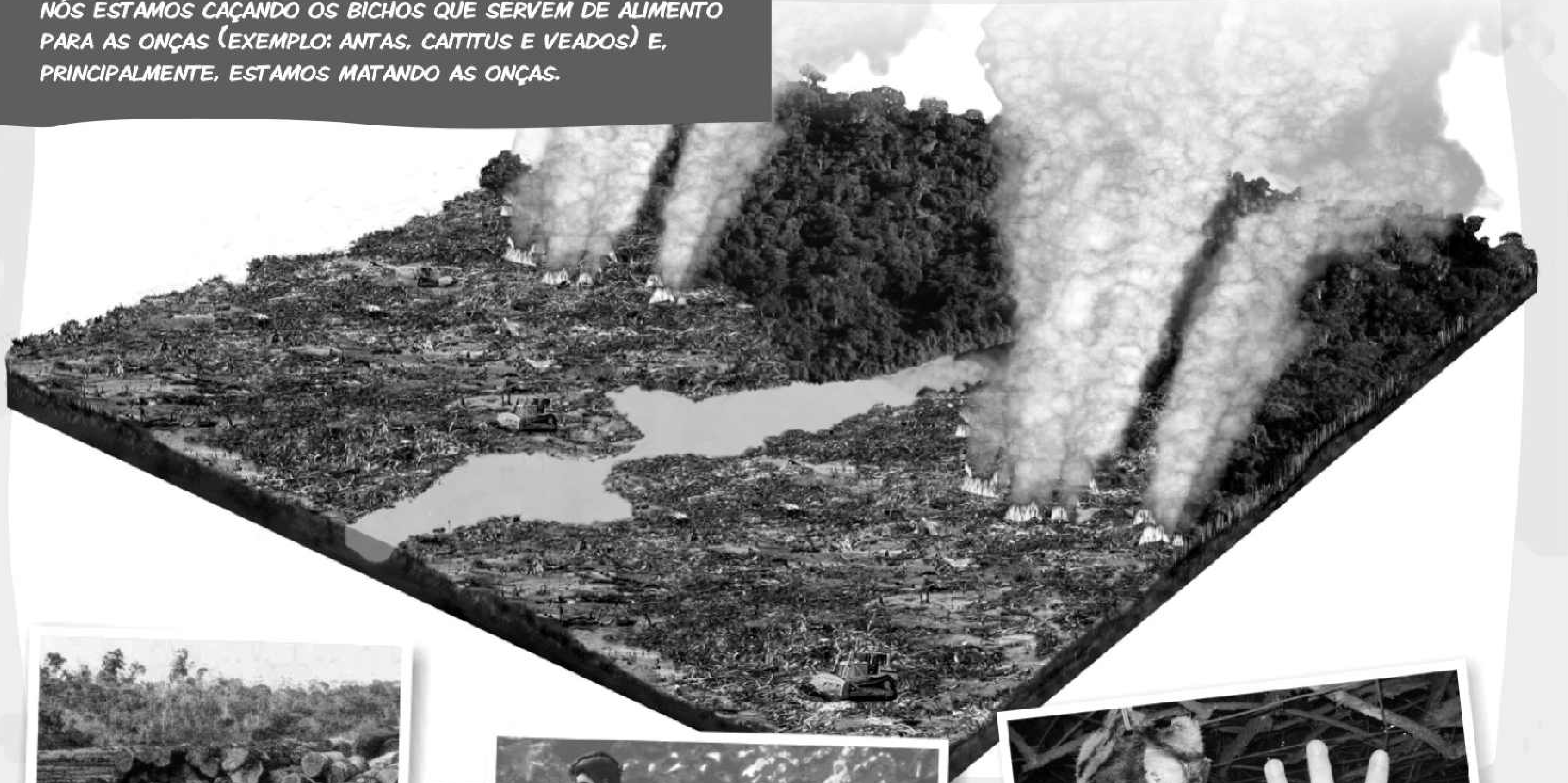

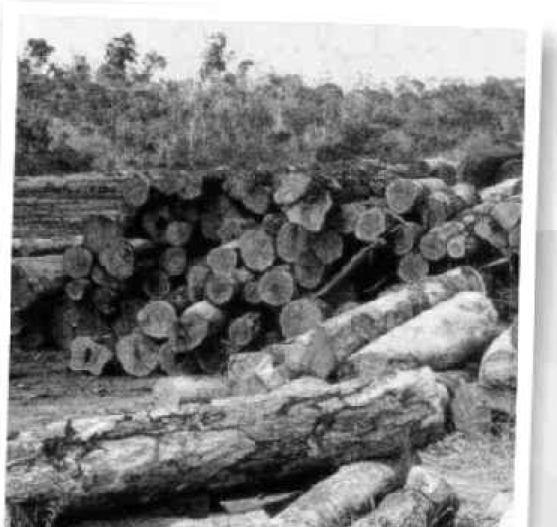

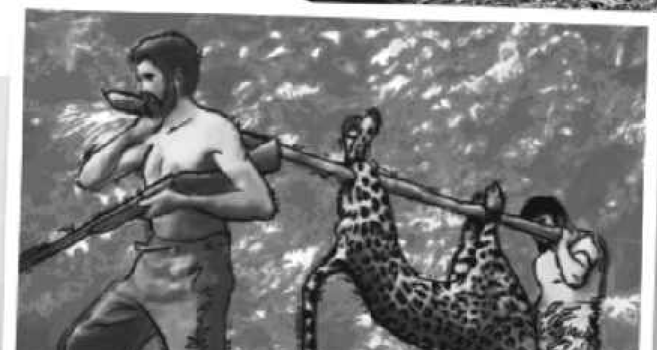

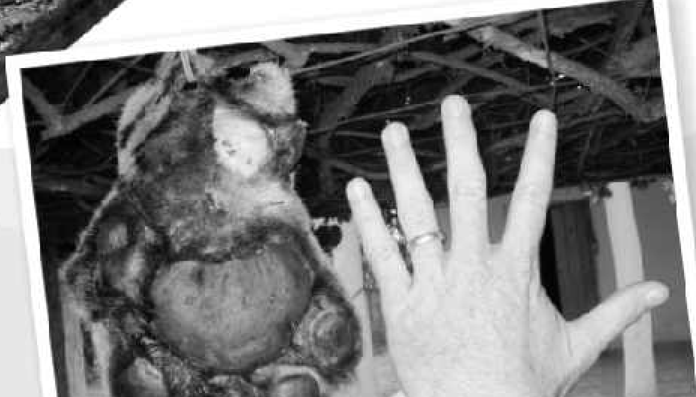

Na paisagem abaixo, descubra que animais servirão de alimento para a onça. Lembre-se que uma onça precisa de abrigo (mata), água e alimento (40 kg de carne por semana) para sobreviver, além de parceiros para reproduzir.

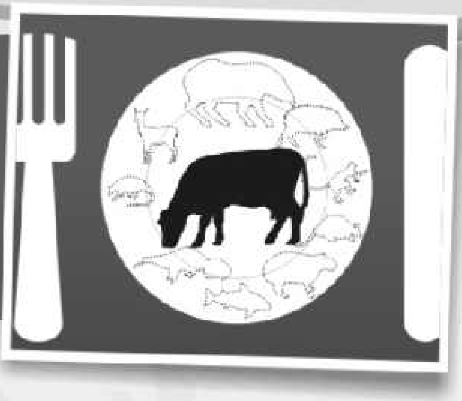

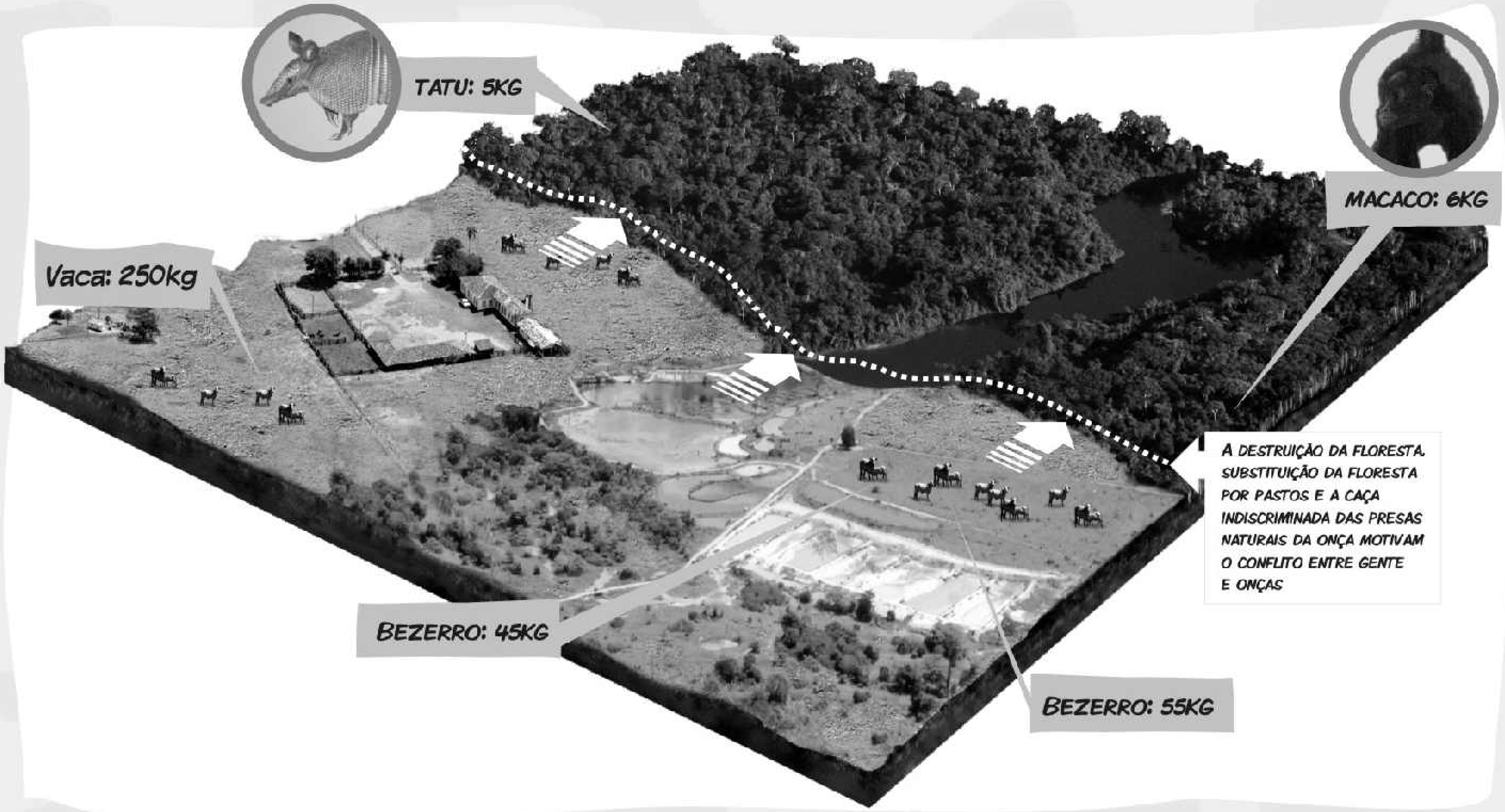


Conclusão, quando as matas e cerrados são transformados em pasto e os bichos da mata são substituídos pelo gado doméstico, a onça vai se alimentar principalmente de:

R: ______________________________

# ONÇAS-PINTADAS: CONFLITO E CONVIVÊNCIA

O que você acha que acontece quando a onça, por falta de bichos do mato para se alimentar, acaba matando e se alimentando de vacas e bezerros?

Faça um X na figura que melhor representa o que acontece depois que uma onça mata uma vaca ou um bezerro.

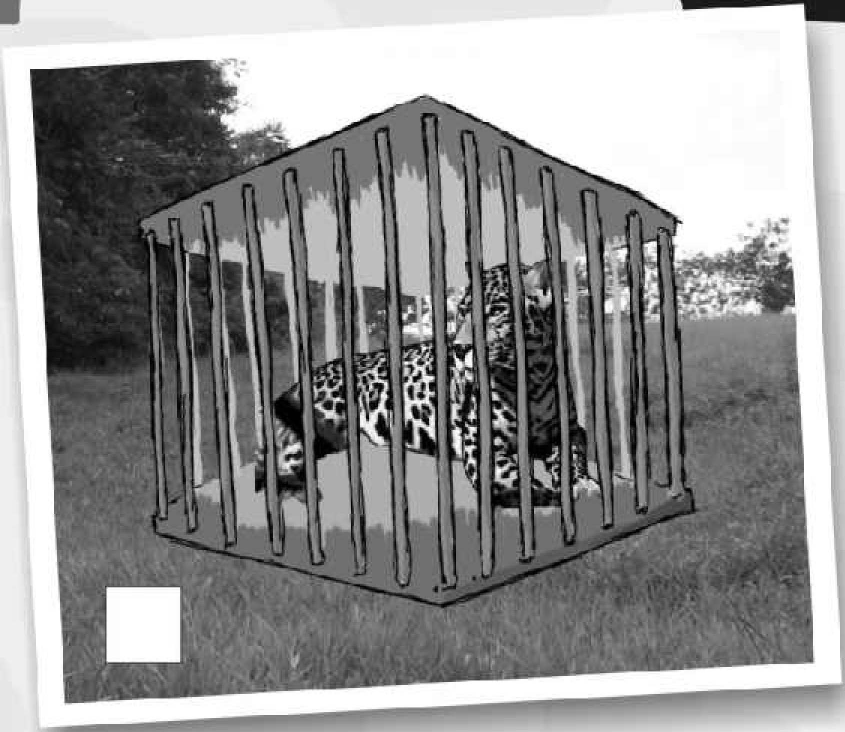

Ela é enjaulada

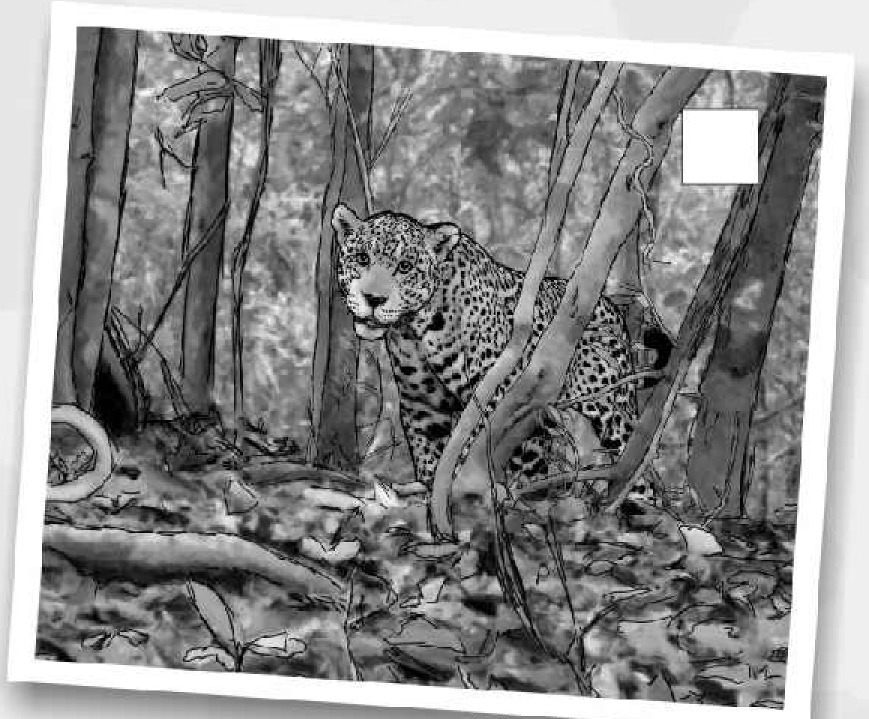

Ela é solta em outro local

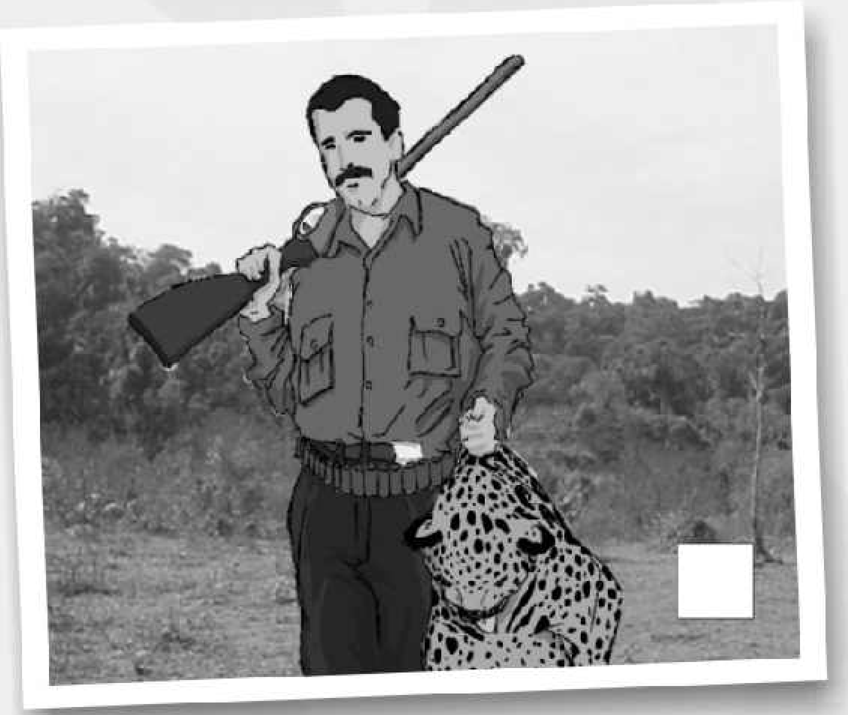

Ela é caçada e morta

## VOCÊ SABIA...

Graças ao desmatamento, ao sumiço dos bichos da mata, e ao abate de onças, esses lindos Felinos estão desaparecendo. A onças já desapareceram de metade da área onde existiam no passado.

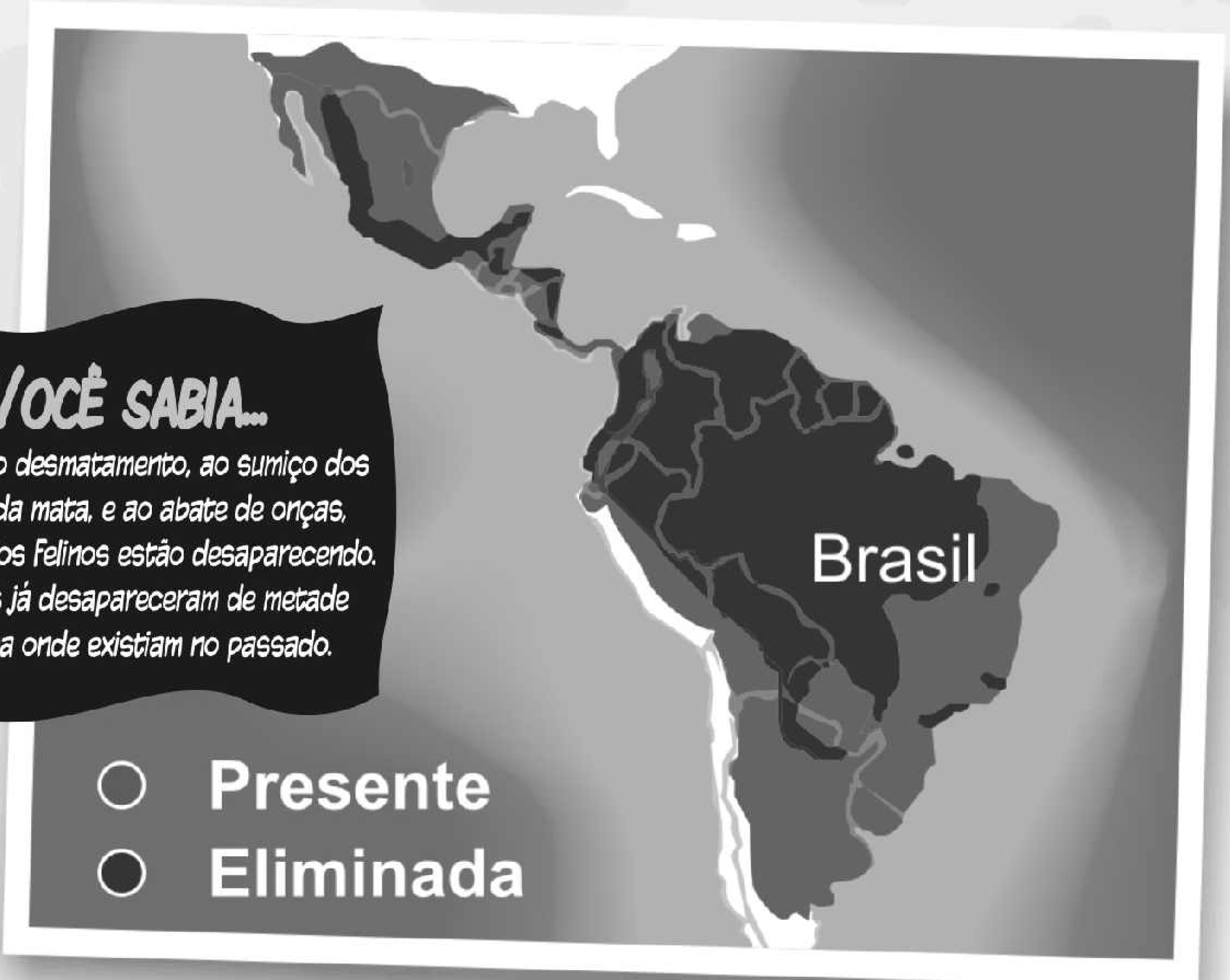

# ONÇAS-PINTADAS: RAZÕES PARA CONVIVER

Será que podemos continuar causando tantos problemas para as onças? Será que podemos deixar que elas desapareçam para sempre? Afinal, queremos viver em um mundo sem onças ou com onças? Bem, saiba que existem várias razões para preferirmos continuar dividindo nosso espaço com as onças. Conheça abaixo algumas dessas razões.

## Razões Ecológicas.

A onça-pintada é o maior predador terrestre do Brasil e se alimenta de uma grande diversidade de animais menores. Entre as presas naturais das onças, existem outros carnívoros, como o lobinho e o mão-pelada, e também espécies que se alimentam de folhas e sementes, como os veados, a anta, a paca e a cutia. Acredita-se que ao se alimentar dessas espécies, as onças-pintadas "controlam" suas populações, ou seja, impedem que as populações cresçam demais. O lobinho e o mão-pelada, por sua vez, controlam as populações de suas presas - ratos, caranguejos, caracóis - assim como os animais comedores de folhas e sementes controlam as populações de plantas das quais se alimentam. Desse modo, por meio de um "efeito-cascata", as onças-pintadas teriam uma ampla influência sobre a floresta onde vivem, afetando desde insetos até árvores.

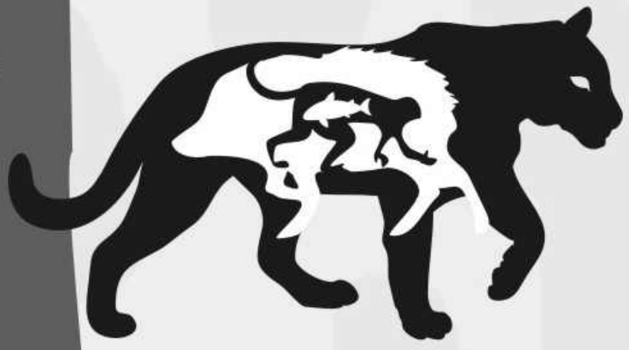

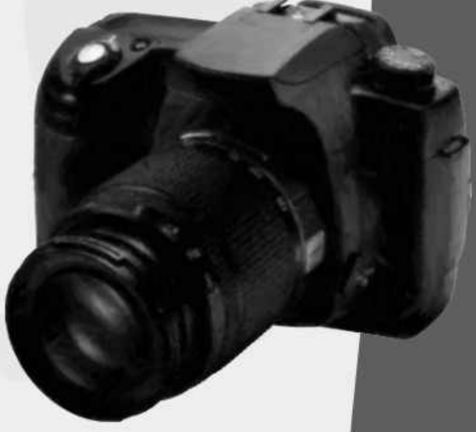

## Razões Econômicas.

A onça-pintada é dos mais belos e fascinantes animais da fauna brasileira. Por essa razão, sua imagem é usada para fins comerciais, especialmente pelo setor turístico. No Estado de Mato Grosso, por exemplo, a onça-pintada aparece mais nos cartazes e folhetos de propaganda turística - produzidos pela Secretaria de Turismo e por agências de turismo - do que qualquer outra espécie (exceto o Tuiuiú). De fato, as onças podem contribuir para o turismo, mesmo que as chances de serem avistadas por turistas sejam remotas. Um número cada vez maior de turistas está disposto a pagar mais pela chance de avistar ou ouvir uma onça, pela experiência de simplesmente estar no território de uma onça, ou ainda pela oportunidade de contribuir para conservação dessa espécie por meio do turismo.

## Razões legais.

Matar onças é ilegal. Mais do que isso, matar onças é um crime segundo a Lei de Crimes Ambientais. Segundo o Artigo 29 daquela lei, "Matar, perseguir, caçar, apanhar, utilizar espécimes da fauna silvestre, nativos ou em rota migratória, sem a devida permissão, licença ou autorização da autoridade competente, ou em desacordo com a obtida: Pena - detenção de seis meses a um ano, e multa".

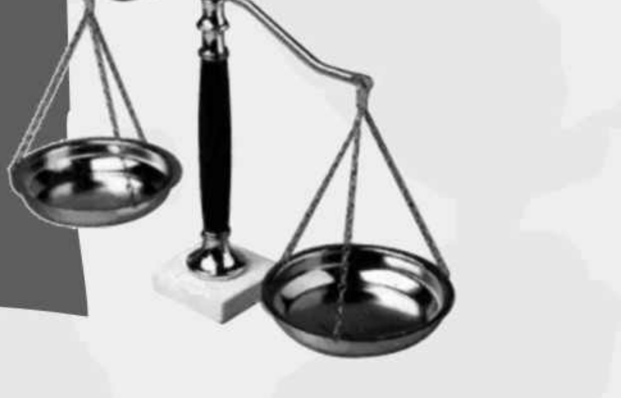

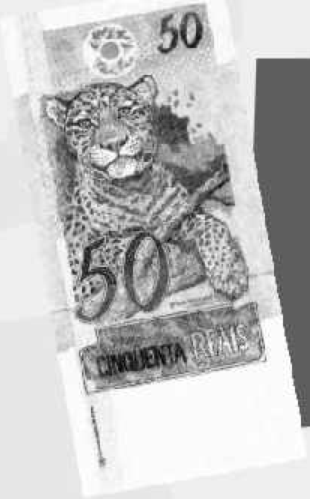

## Razões culturais.

A onça é um ícone da cultura brasileira. Ela contribui para a manifestação de nossa cultura em suas mais variadas formas, do artesanato local aos clássicos da literatura infantil, de pinturas que retratam a história do país à cédula de cinqüenta reais. Poucas espécies de nossa fauna possuem presença tão marcante como símbolo. A onça é símbolo de astúcia, agilidade, vigor e velocidade, e acima de tudo, a espécie tem sido usada para expressar o poder da Natureza. Por capturar a atenção e o interesse tanto de adultos quanto de crianças, a onça é o ponto de partida ideal para atividades de educação e comunicação para a conservação da natureza.

## Razões emocionais.

Os felinos sempre exerceram um grande fascínio sobre as pessoas. Por serem os maiores felinos do continente e por sua beleza excepcional, as onças despertam emoções que vão da admiração ao medo, do fascínio à raiva. Nenhum outro animal de nossa fauna desperta sentimentos tão fortes e contrastantes. Além disso, é por razões emocionais que não queremos que as onças desapareçam para sempre. É parte da natureza humana valorizar a diversidade em todas as suas dimensões, seja ela material, cultural ou natural. Pela mesma razão que tombamos edifícios históricos e abrigamos obras de arte em museus, nos sentimos apegados às onças o suficiente para preferir que elas continuem existindo.

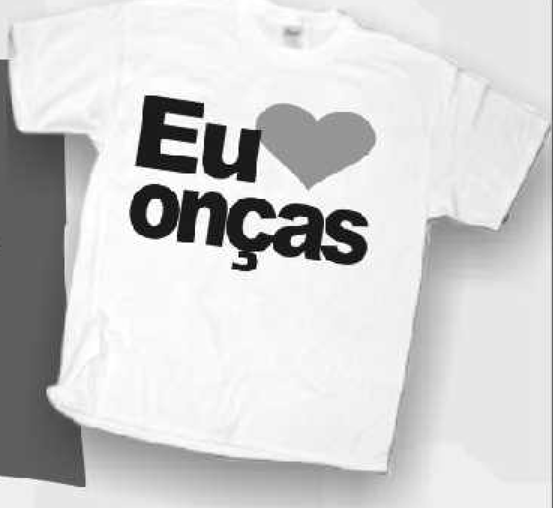


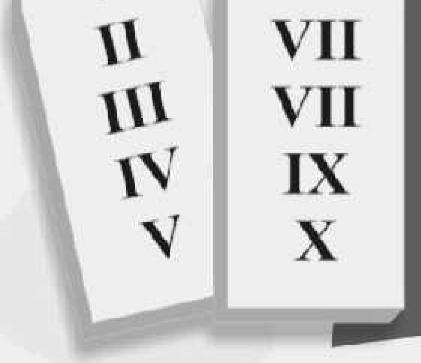


## Razões éticas.

"Matar onças é errado". "Levar uma espécie à extinção é imoral". É nisso que acredita um número cada vez maior de pessoas. A compreensão de que não são as onças que invadem o espaço das pessoas, mas sim as pessoas que invadem o espaço das onças, e de que as onças não causam prejuízo propositalmente ou por maldade, mas sim por seguirem seus instintos de predador, contribuem para a percepção de que matar onças é errado. Além disso, a noção de que as onças, assim como as pessoas, têm o direito de existir e de manter seu modo de vida ancestral tornam imoral nossas ações que ameaçam as onças de extinção.

# ONÇAS-PINTADAS: COMO PODEMOS CONVIVER

A principal maneira de conter a perda do habitat das onças é a criação de unidades de conservação, ou seja, de áreas que são protegidas do desmatamento e outras perturbações. Criar unidades de conservação sempre foi responsabilidade do Estado, mas proprietários rurais também podem desempenhar um papel importante, seja cumprindo a legislação ambiental que estabelece a existência da Área de Preservação Permanente (APP) e da Reserva Legal, seja criando uma Reserva Particular do Patrimônio Natural (RPPN). Para maiores informações sobre a criação de uma RPPN, visite www.ibama.gov.br/rppn.

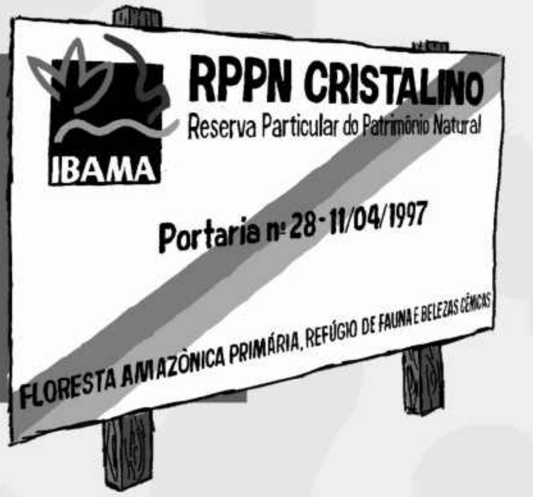


Veja o significado das placas na página ao lado e aprenda como transformar uma área em uma fazenda modelo!

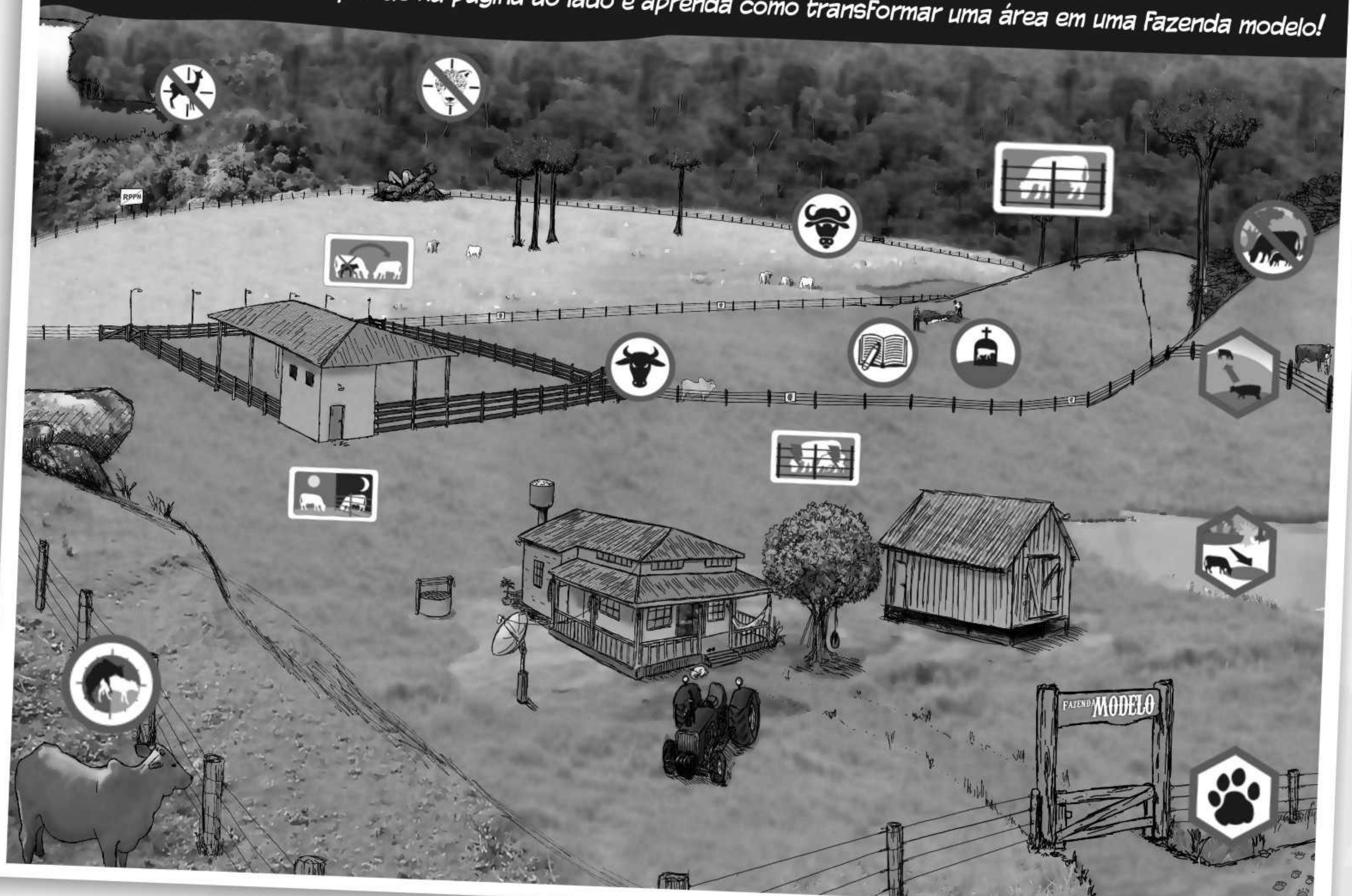

CONCLUINDO, SE VAMOS CONVIVER COM AS ONÇAS-PINTADAS, ENTÃO PODEMOS TOMAR VÁRIAS MEDIDAS PARA DIMINUIR OS PROBLEMAS QUE ELAS NOS CAUSAM ASSIM COMO OS PROBLEMAS QUE CAUSAMOS A ELAS. ENTRE AS VÁRIAS MEDIDAS RECOMENDADAS PARA A PREVENÇÃO DO ATAQUE DE ONÇAS AO GADO DOMÉSTICO[1], DESTACAM-SE:

Não caçar e não permitir a caça às presas naturais das onças.

Não caçar e não permitir a caça à onça. A caça à onça pode resultar emonças com limitações físicas (por exemplo, dentes quebrados) que asimpedem de capturar suas presas naturais, forçando-as a atacar o gadodoméstico.

Usar cercas para impedir que o gado entre na mata.

Construir reservatórios (poços ou tanques) de água, sempre que possível, longe da mata.

Não manter vacas prenhes ou com bezerros próximas à mata. Estas vacas devem ser mantidas em áreas abertas, preferencialmente perto da sede ou de outras habitações humanas.

Usar cercas elétricas ao redor de pastos usados como maternidade. Cercas elétricas, porém, são caras e exigem manutenção frequente e minuciosa para garantir seu bom funcionamento.

Manter, ao invés de vender, alguns animais experientes do rebanho (bois ou vacas velhas com chifres) que ensinem aos animais mais jovens um comportamento adequado de agrupamento para evitar os ataques por onça. Além disso, pode-se pendurar sinos em alguns indivíduos do rebanho, o que também constitui um medida útil.

Em áreas de matas extensas com alta incidência de predação, recolher os animais ao anoitecer em mangueiros adequados e/ou próximos a habitações humanas ou em áreas com cerca elétrica. Apesar de um pequeno aumento nos custos de operação, esta medida simples é muito eficiente para reduzir os impactos negativos da predação e os animais se acostumam facilmente a ela. A instalação de luzes nos mangueiros também constitui um medida útil.

Em locais com alta incidência de predação, substituir a atividade de cria por recria e/ou engorda. Ou seja, estas áreas devem ser utilizadas com bovinos acima de 1-2 anos de idade.

Estabelecer estações de monta curtas, de 3-4 meses de duração, ao invés de fazer a monta o ano todo. Durante a temporada mais reduzida de partos, é conveniente uma boa supervisão dos partos e dos bezerros recém-nascidos.

Deslocar os rebanhos que pastam nas áreas baixas alagáveis em direção a áreas mais altas para que não fiquem isolados e debilitados pelas enchentes, o que os torna mais vulneráveis ao ataque por onças.

Desfazer-se convenientemente dos corpos de animais domésticos mortos por outras causas (picada de cobra, vacas mortas por problemas de parto, etc.), para impedir que sejam devorados por felinos e estes adquiram a tendência para seu consumo.

Em áreas inundáveis com alta incidência de ataque de onças ao gado doméstico, a introdução de pequenos rebanhos de búfalos leiteiros mansos (da raça Murrah), manejados isoladamente ou em conjunto com os rebanhos bovinos, se revelou uma técnica eficiente para diminuir os problemas de depredação em várias fazendas de gado na Venezuela. É importante destacar que os búfalos necessitam de um trato contínuo e intensivo para evitar que se tornem selvagens ou baguás se transformando em outro problema ambiental. Este é uma problema que pode ser facilmente evitado com um bom manejo. Além disso, existe evidência de que nesses tipo de ambiente o búfalos são muito mais produtivos que o gado bovino.

Conhecer a aparência e os sinais das presas domésticas predadas por felinos e saber diferenciá-las daquelas causadas por bandos de cães selvagens ou baguás e por ladrões de gado.

Manter registros detalhados da mortalidade e suas causas e manter em dia o inventário, com contagens mensais, além de verificar as perdas reais e suas causas e comparar anualmente as informações de porcentagem de mortalidade e suas causas.

1. Manual sobre problemas de predação do gado por onças-pintadas e onças-pardas em fazendas de gado, de Rafael Hoogesteijn

# ONÇAS-PINTADAS: SAIBA MAIS

## Onças-pintadas em livros.

- **Guia de Convivência Gente e Onças**, de Silvio Marchini e Ricardo Luciano, 2008. Fundação Ecológica Cristalino e Wildlife Conservation Research Unit. Obra que serviu de referência para esse livro de atividades.

- Manual sobre Problemas de Predação do Gado por **Onças-Pintadas e Onças-Parda** em Fazendas de Gado, de Rafael Hoogesteijn. Wildlife Conservation Society, 2005. É uma referência importante para a resolução de problemas de ataque de onças ao gado.

- Manual de Identificação, Prevenção e Controle de Predação por Carnívoros, de Maria Renata Pereira Leite Pitman, Tadeu Gomes de Oliveira, Rogério Cunha de Paula e Cibele Incrusiak. Ibama, 2002. Outra referência importante para a resolução de problemas de predação por **onças**.

- Nas Selvas do Brasil, de Theodore Roosevelt. Editora Itatiaia. Livro de 1914, relata as viagens do autor pelas selvas e rios do Brasil em companhia de Cândido Rondon. Ele descreve suas caçadas de **onça** no Pantanal.

- El Jaguar en el Nuevo Milenio, da Wildlife Conservation Society em 2002. Reúne artigos científicos sobre **onças**. Em espanhol.

- El Jaguar, Tigre Americano, de Rafael Hoogestejin. Ediciones Armitano (Venezuela), 1992. Traz informações gerais sobre biologia, ecologia e conservação de **onças**, com foco nos problemas de predação do gado. Em espanhol.

- Jaguar, de Alan Rabinowitz. Editora Arbor House, 2000. Relato da criação de uma reserva para a conservação de **onças** em Belize e da fundação de uma longa história de pesquisa sobre a espécie no país, pelo próprio autor. Em inglês.

- Tigrero!, de Sasha Siemel. Um clássico de 1953, editado pela Prentice-Hall. Relato do ucraniano que viveu no Brasil nos anos 50, realizando safáris e caçando onças com
•zaguaia (empalando-as) no Pantanal. Em inglês.
- Jaguar Hunting in the Matto Grosso and Bolivia, de Tony de Almeida. Editora Safari Press. Outro clássico e obra rara, escrito em 1976. Entre as estórias de caçadas no Pantanal, o autor aborda, pela primeira vez, detalhes sobre a ecologia da **onça-pintada**. Em inglês.

## Onças-pintadas em revistas

- **Revista Terra da Gente.** "Face a Face com a Rainha", de Helen Sacconi. Edição de Fevereiro de 2009.

- **Revista Terra.** "Com a onça na mira", com fotos e texto de Adriano Gambarini. Edição de Setembro de 2005.

- **National Geographic Magazine.** A famosa revista americana já publicou vários artigos sobre onças. Entre eles, destacam-se:
- **Path of the Jaguar**, de Mel White (Março de 2008)
- **Brazil' Wild Wet,** de Susan McGrath (Agosto de 2005)
- **Phantom of the Night**, de Douglas Chadwick (Maio de 2001)
- **Cats: Nature's Masterwork,** de Cathy Newman (Junho de 1977)
- **The Jungle was my Home,** de Sasha Siemel (Novembro de 1952)
- **King of Cats and his Court,** de Victor Cahalane (Fevereiro de 1943)

## Onças-pintadas na internet.

**Pró-carnívoros**
(http://www.procarnivoros.org.br).
Informação sobre carnívoros brasileiros: projetos de pesquisa e conservação, cursos, referências bibliográficas.

**Wikipedia**
(http://pt.wikipedia.org/wiki/On%C3%A7a-pintada).
Informação enciclopédica sobre a onça-pintada.

**National Geographic**
(http://animals.nationalgeographic.com/animals/mammals/jaguar.html).
Informações gerais, mapa de distribuição, multimidia.

**IUCN/Cat Specialist Group**
(http://lynx.uio.no/lynx/catsgportal/cat-website/20_catwebsite/home/index_en.htm).
Descrição da onça-pintada.

**Jaguar Conservation Network**
(http://www.jaguarnetwork.org).
Portal de informação sobre projetos de pesquisa, conservação e resolução de conflitos com onças-pintadas. Em inglês.

**Save the Jaguar**
(http://www.savethejaguar.com).
Informação geral sobre onças e projetos de pesquisa e conservação da Wildlife Conservation Society. Em inglês.

**Wildlife and People**
(http://www.peopleandwildlife.org.uk).
Portal sobre conflitos entre gente e fauna silvestre: projetos de pesquisa, eventos, referências bibliográficas, manuais de resolução de conflitos. Em inglês.

**Jaguar Conservation Fund**
(http://www.jaguar.org.br)
Informação sobre onças e projetos desenvolvidos pelo Instituto Onça-Pintada.

**Projeto Puma**
(http://uniplac.net/~puma/page_main_port3.html)
Informação sobre onça-parda e problemas de depredação associados à espécie.

## Onças-pintadas em vídeos e documentários

- YouTube (http://www.youtube.com). Possui vários vídeos que mostram **onças pintadas** em diversas situações. A qualidade dos vídeos é variável. Para encontrar vídeos de **onças** no YouTube, entre as palavras "**onça**" ou "**jaguar**" no buscador.

- National Geographic. Vídeos curtos estão disponíveis no site da organização. Dois exemplos:

    - Jaguars vs Croc **(http://video.nationalgeographic.com/video/player/animals/index.html)**

    - Jaguar **(http://video.nationalgeographic.com/video/player/specials/photography specials/behind-the-shot/jaguar-sartore.html)**.

- Sasha Siemel, o Caçador de **Onças**. Documentário de Cândido Alberto Fonseca sobre o letoniano que caçava **onças** com zaguaia – uma espécie de lança - no Pantanal nos anos 50.

## Para ver e fotografar onças-pintadas.

- ***Na natureza.*** **Onças** são extremamente difíceis de ver na natureza. Abundância relativamente baixa, hábitos arredios e silenciosos, e preferência por matas densas e por caminhar no escuro, fazem da **onça-pintada** uma das espécies menos avistadas da nossa fauna. As regiões do Brasil com infra-estrutura turística e melhores chances de ver **onças** são o rio Araguaia, o Pantanal Norte (especialmente na segunda metade da Rodovia Transpantaneira e ao longo do rio Cuiabá em Poconé e rio Paraguai em Cáceres) e Pantanal Sul (nos hotéis-fazenda e rios de Miranda). **Atenção:** *caso tenha o privilégio de avistar uma onça, mantenha-se sempre a uma distância segura dela.*

- ***Com "armadilhas fotográficas".*** Armadilhas fotográficas são câmeras fotográficas acopladas a sensores de infravermelho que disparam automaticamente com a aproximação de um animal de sangue quente. Instaladas ao longo de estradas e trilhas, elas fazem o trabalho de esperar por dias pela passagem de uma **onça** a ser fotografada, enquanto você fica em casa. As câmeras podem ser convencionais ou digitais. Nesse último caso, elas podem ser programadas para filmar animais que passam a sua frente, em lugar de fotografar. As marcas mais importantes de armadilhas fotográficas são as americanas Camtrakker (**www.camtrakker.com**) e as nacionais Tigrinus (**www.tigrinus.com.br**).

- ***Em cativeiro.*** Segundo o CENAP[1], existem aproximadamente 160 **onças-pintadas** em cativeiro no Brasil, a maioria delas em zoológicos abertos à visitação pública. Portanto, se você quer ver uma **onça** ao vivo, a maneira mais prática e garantida de fazê-lo é visitando o zôo mais perto de você. Veja a seguir a lista de zoológicos que abrigam **onças-pintadas**.

# Onças-pintadas: onde encontrar?

Criadouro da Itaipu Binacional em Foz do Iguaçu (PR)
Criadouro Luciano Sabóia em Curitiba (PR)
Fundação Zoobotânica de Belo Horizonte (MG)
Parque Ambiental Chico Mendes em Rio Branco (AC)
Zôo da Santur em Balneário Camboriú (SC)
Zôo de Americana (SP)
Zôo de Aracaju (SE)
Zôo de Araçatuba (SP)
Zôo de Bauru (SP)
Zôo de Brasília (DF)
Zôo de Brusque (SC)
Zôo de Cachoeira do Sul (RS)
Zôo de Campinas (SP)
Zôo de Cascavel (PR)
Zôo de Catanduva (SP)
Zôo de Curitiba (PR)
Zôo de Foz do Iguaçu (PR)
Zôo de Goiânia (GO)
Zôo de Guarulhos (SP)
Zôo de Ilha Solteira (SP)
Zôo de Ipatinga (MG)
Zôo de João Pessoa (PB)
Zôo de Leme (SP)
Zôo de Limeira (SP)
Zôo de Lins (SP)
Zôo de Pedreira (SP)
Zôo de Piracicaba (SP)
Zôo de Pomerode (SC)
Zôo de Pouso Alegre (MG)
Zoo de Recife (PE)
Zôo de Ribeirão Preto (SP)
Zôo de Rio Preto (SP)
Zôo de São Carlos (SP)
Zôo de São Paulo (SP)
Zôo de São Vicente (SP)
Zôo de Sapucaia (SP)
Zôo de Sorocaba (SP)
Zôo de Taboão da Serra (SP)
Zôo de Teresina (PI)
Zôo da UFMT em Cuiabá (MT)
Zôo do Hotel Fazenda Mato Grosso
Zôo de Varginha (MG)
Zôo de Volta Redonda (RJ)
Zôo do Centro de Instrução de Guerra na Selva em Manaus (AM)
Zôo do Hotel Tropical em Manaus (AM)
Zôo do Museu Paraense Emílio Goeldi em Belém (PA)
Zôo do Rio de Janeiro (RJ)
Zôo Irmão Ézio Danza e Silva em Poços de Caldas (MG)

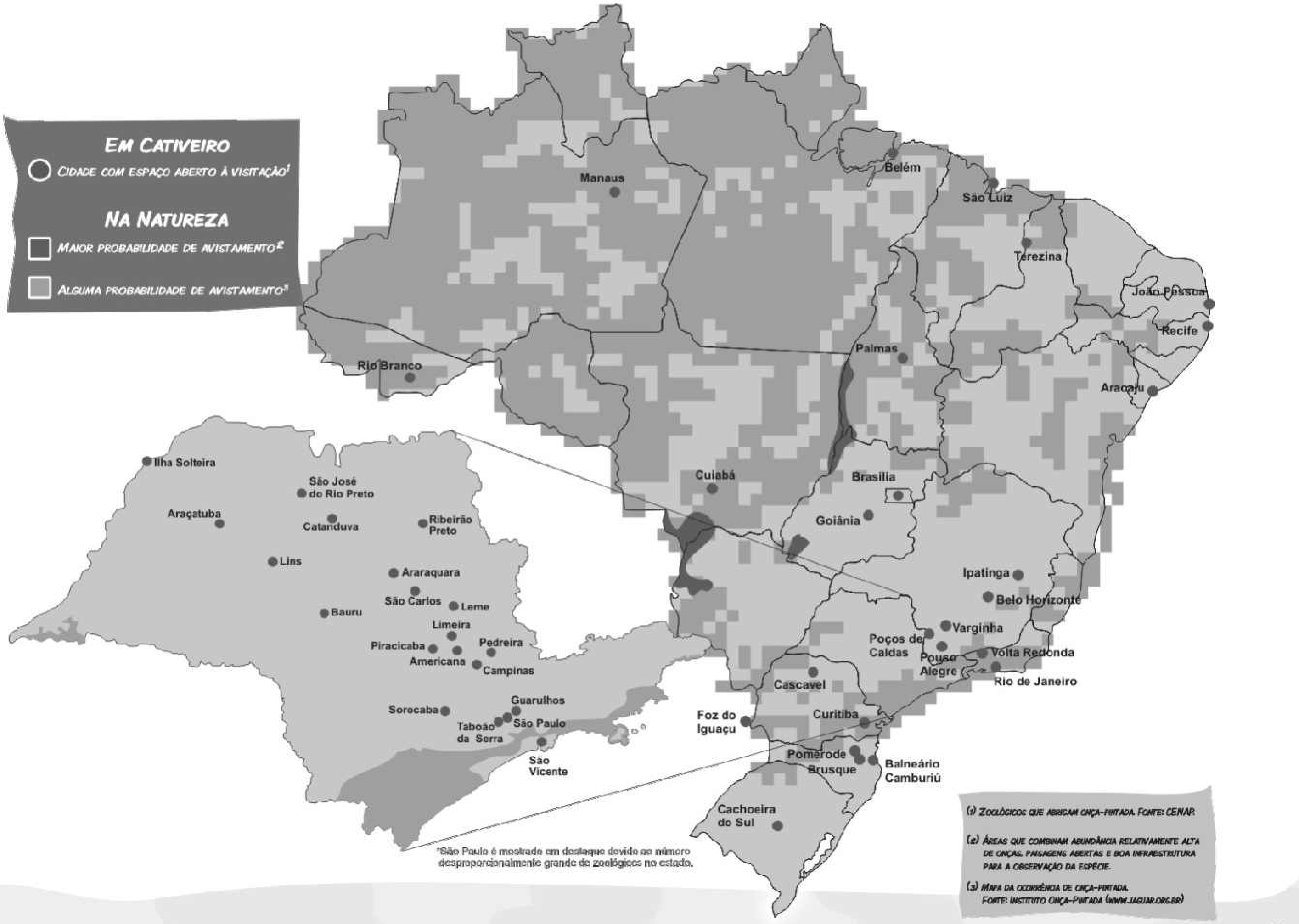


(1) Zoológicos que abrigam onça-pintada. Fonte: CENAP.

(2) Áreas que combinam abundância relativamente alta de onças, paisagens abertas e boa infraestrutura para a observação da espécie.

(3) Mapa da ocorrência de onça-pintada. Fonte: Instituto Onça-Pintada (www.jaguar.org.br)

CENAP: Centro Nacional de Pesquisas para a Conservação de Predadores Naturais.
http://www.procarnivoros.org.br/cenapibama.php

# GABARITO

## 1

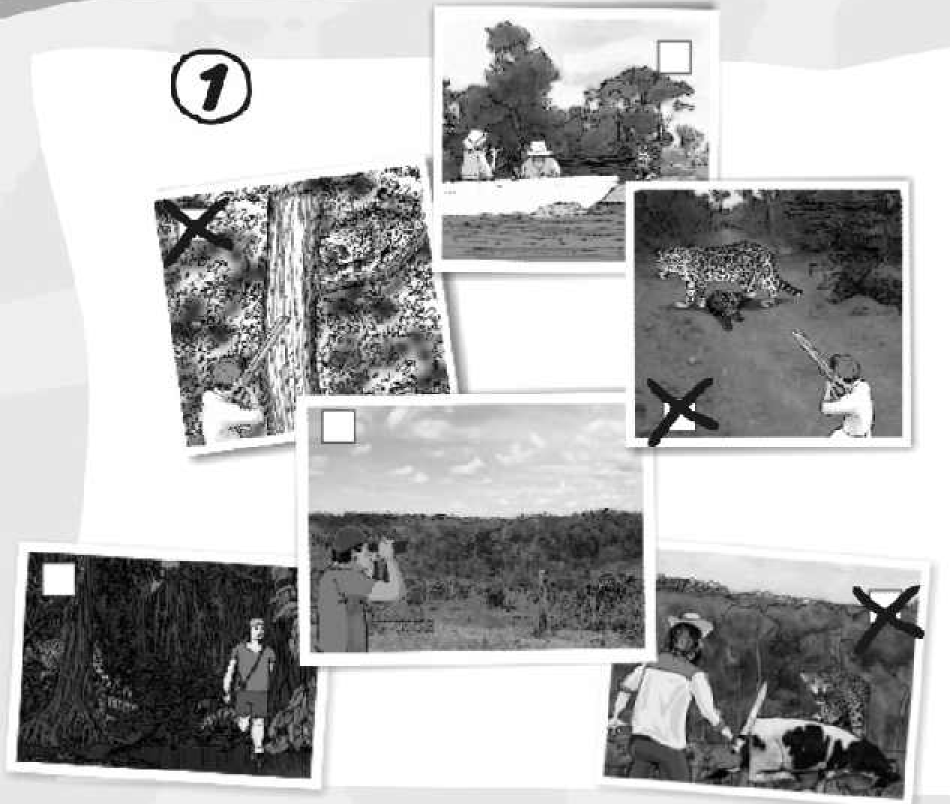

## 2

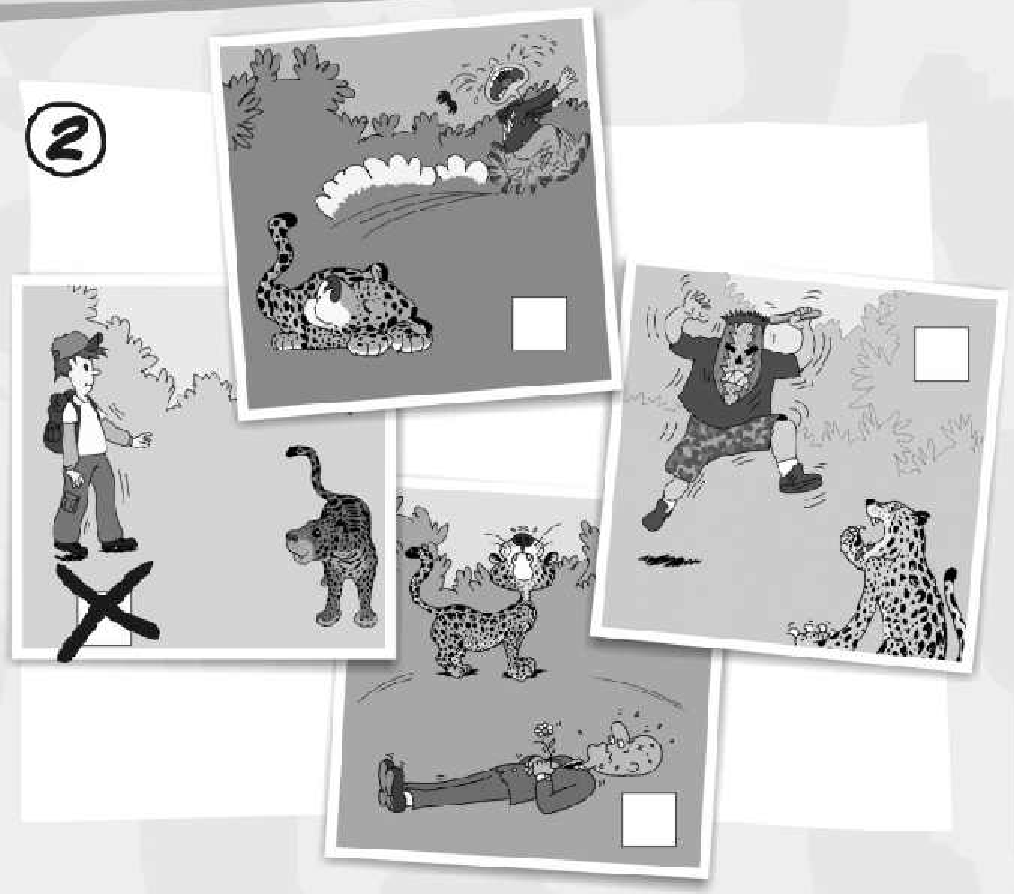

## 3

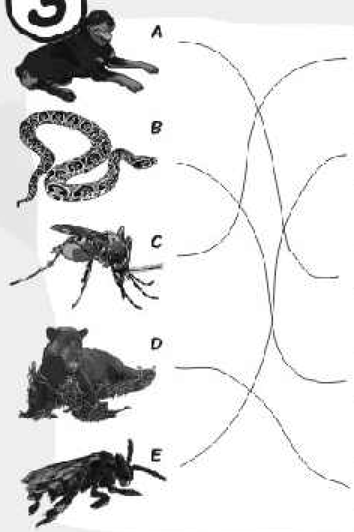


**C** — Transmitem doenças infecciosas, entre elas dengue, leishmaniose, febre amarela e malária. Matam milhões de pessoas no mundo todos os anos.

**E** — Podem ser úteis por produzirem alimento. Uma delas sozinha oferece pouco perigo. Mas como geralmente vivem em colônias de dezenas a centenas de indivíduos, um ataque maciço pode ser fatal. Matam centenas de pessoas no mundo todos os anos.

**A** — São conhecidos como "o melhor amigo do homem". Mas nem todos são tão amigáveis. Na verdade, alguns são bravos o suficiente para atacar e matar o ser humano. Eles matam, em média, [illegible] pessoas por ano no mundo.

**B** — Elas sempre foram temidas. Até na Bíblia uma delas aparece como a primeira vilã da história. Embora a maioria das espécies é inofensiva, algumas possuem venenos fatais. Causam milhares de mortes por ano no mundo.

**D** — Apesar da fama de má e de ser uma predadora super bem armada, ela é tímida e prefere evitar o confronto com o homem. Ataca para se defender ou defender seus filhotes. No Brasil, existe apenas UM ataque fatal conhecido.

## 4

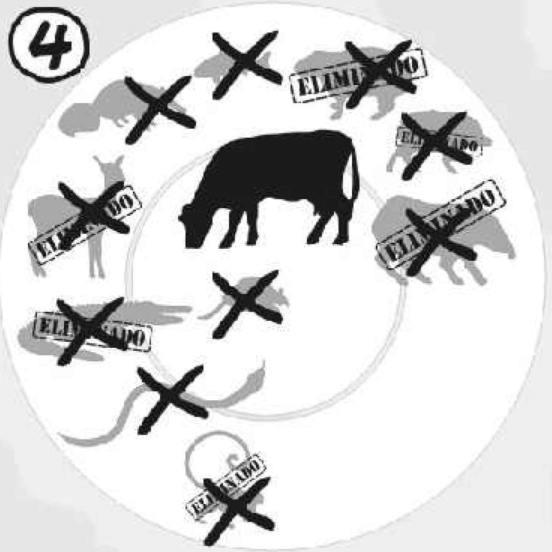


## 5

| | | | | | | | | | | | |
|---|---|---|---|---|---|---|---|---|---|---|---|
| 50 | ½ | 100 | 1 | 250 | 2½ | 500 | 5 | 1500 | 15 | 2000 | 20 |

## 6

R: Bezerros e Vacas

## 7

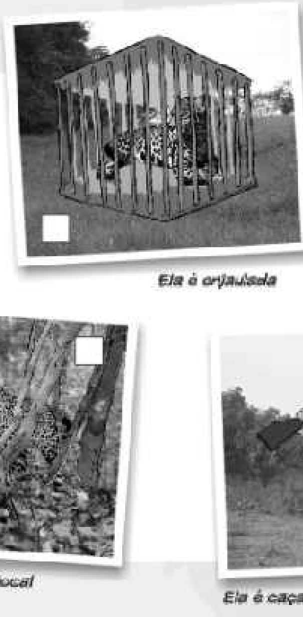

Ela é enjaulada

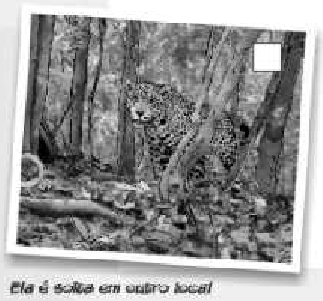

Ela é solta em outro local

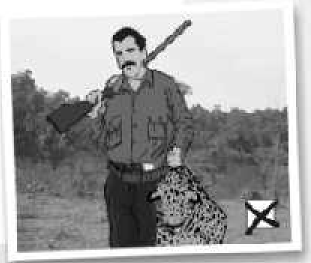

Ela é caçada e morta

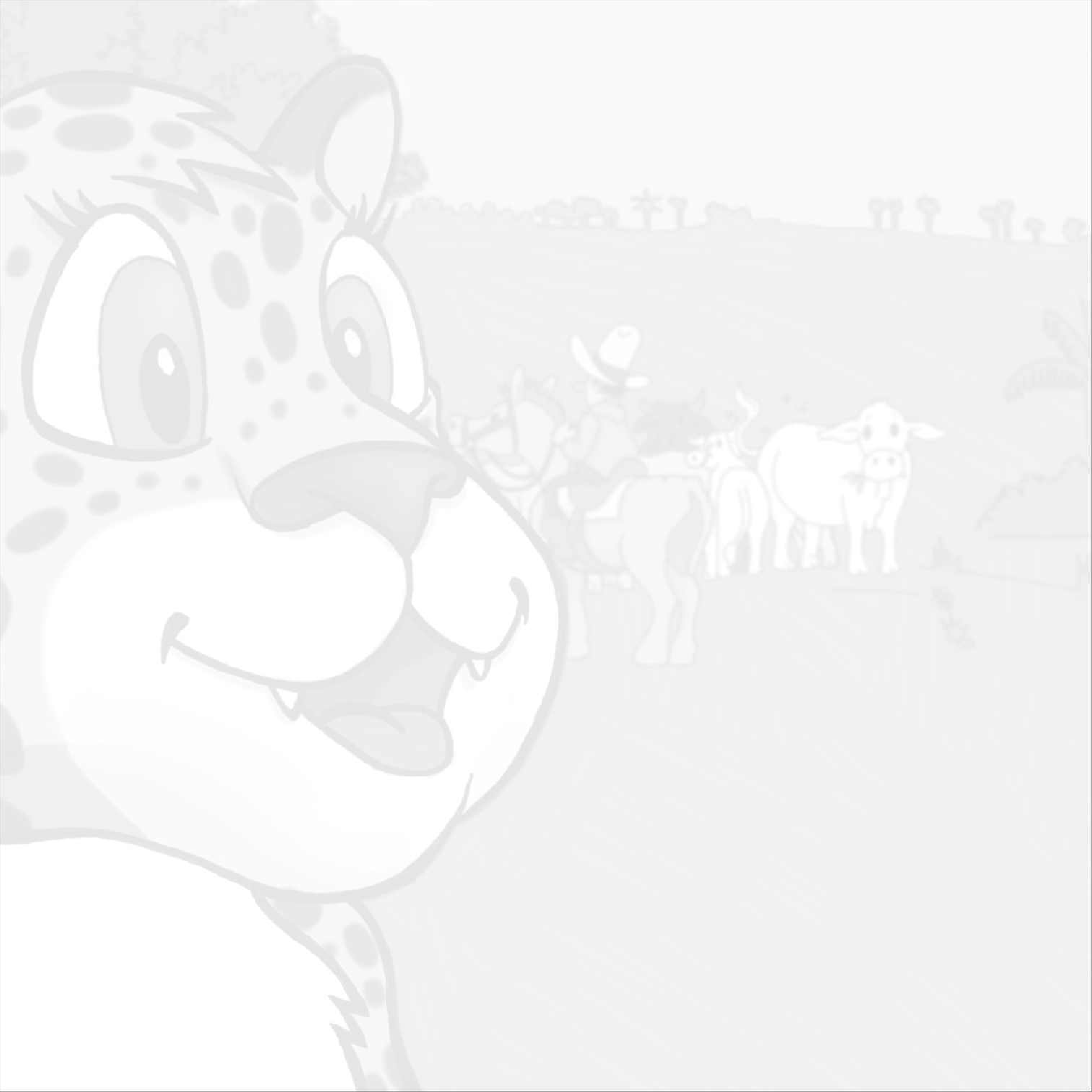

O **Projeto Conviver Gente & Onças** é uma realização da **Fundação Ecológica Cristalino (FEC)** e da **Wildlife Conservation Research Unit (WildCRU)**.

A **FEC** foi fundada em 1999 com a missão de promover a conservação da bio-diversidade na fronteira agrícola da Amazônia no norte do Mato Grosso e, em particular, na área de entorno do **Parque Estadual Cristalino**. Ela tem quatro frentes principais de atuação: políticas públicas, áreas protegidas, pesquisa e educação. As atividades de educação são desenvolvidas pela **Escola da Amazônia**. Além de campanhas de informação e sensibilização sobre onças-pintadas, a Escola da Amazônia realiza oficinas para crianças, jovens e educadores sobre temas relacionados à conservação da bio-diversidade.

A **WildCRU** foi fundada em 1986 como parte do **Departamento de Zoologia da Universidade de Oxford**, na Inglaterra, e é reconhecida hoje como uma das mais importantes instituições no mundo dedicadas à pesquisa e conservação de carnívoros. Sua missão é desenvolver soluções práticas para problemas de conservação por meio de pesquisa científica do mais alto calibre. A **WildCRU** também oferece treinamento em pesquisa e conservação, participa da implementação de projetos de conservação em vários países e reconhece a necessidade de educar e envolver a sociedade a fim de se obter soluções duradouras.

www.fundacaocristalino.org.br

**Appendix S3. Elaboration exercise.**

Schedule

1) Chairs were rearranged in the classroom to form a circle so that students could face each other; students were given paper and pensil;

3) Each student wrote down the name of the animal he/she disliked most and, one by one, showed and spoke to the group his/her choice and one reason for choosing it;

4) The group was divided into two sub-groups: the ones who disliked jaguars and the others (most of whom disliked snakes, so they all represented snakes thereafter);

5) Each sub-group listed on a flipchart all the possible arguments to support their choice; as a way of motivation, the researcher announced that the sub-group that raised the largest number of arguments would win that stage of the game;

6) Sub-groups presented their results to each other; all the members of the sub-group stood beside the flipchart and one or two representatives of the sub-group presented the results orally; the researcher encouraged the debate between sub-groups by disputing some of the arguments; group debated;

8) Sub-groups switched sides: the sub-group that had raised arguments against snakes had to add arguments to the list of arguments against jaguars and vice-versa; as a way of motivation, the researcher announced that the sub-group that raised the largest number of additional arguments would win that stage of the game;

10) Sub-groups presented the results to each-other; the researcher encouraged the debate between sub-groups by disputing some of the arguments: group debated;

11) Students returned to their chairs; the researcher presented to the group the flipchart with all the arguments against jaguars and orally synthesized the results;

12) The researcher invited the students for an exercise of imagination: "Imagine that you have the power to make all the jaguars disappear forever by simply pressing a button. Would you press that button?";

13) Students answered individually by writing *yes* or *no* on a piece of paper; one by one, they showed and spoke to the group their decision and explained why;

14) The group was divided into two sub-groups: the ones who would press the button and the ones who would not;

15) Each sub-group listed on a flipchart all the possible benefit they would derive from maintaining or exterminating the jaguars; as a way of motivation, the researcher announced that the sub-group that raised the largest number of arguments would win that stage of the game;

16) Sub-groups presented their results to each other and debated;

17) Students were given the chance of switching sides.

**Appendix S4. Book 'People and jaguars: a guide for coexistence'.**

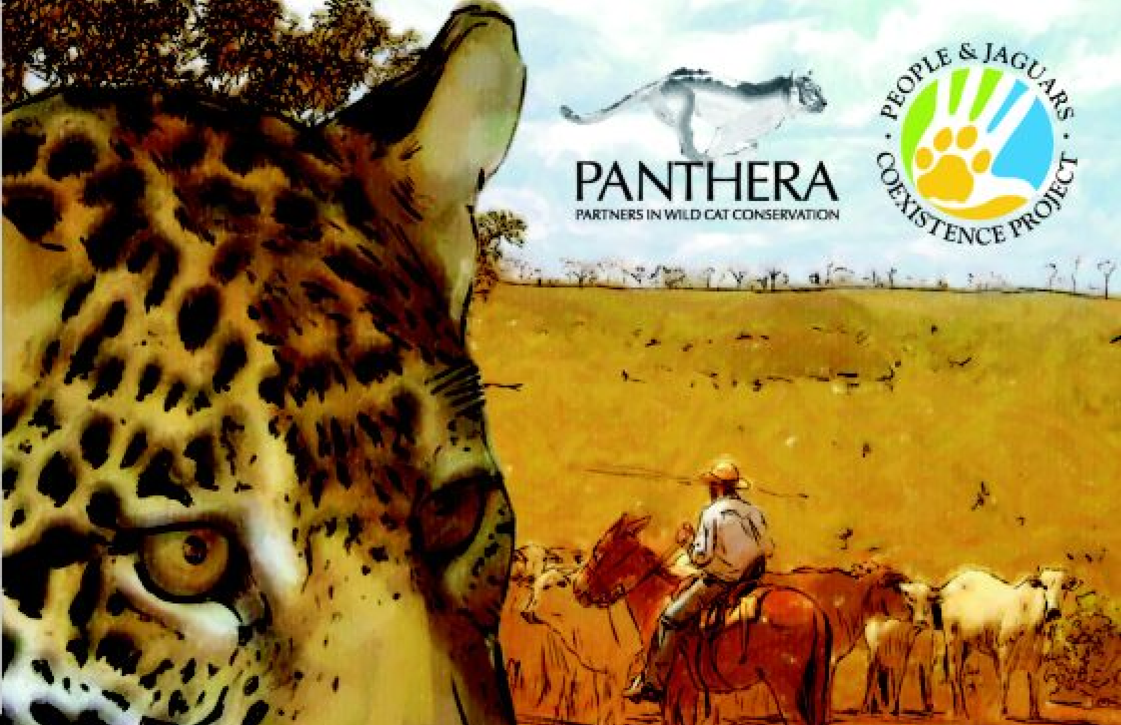


# People and Jaguars
# A Guide for Coexistence

Silvio Marchini / Ricardo Luciano / Rafael Hoogesteijn

Marchini, Luciano & Hoogesteijn

People and Jaguars: A Guide for Coexistence

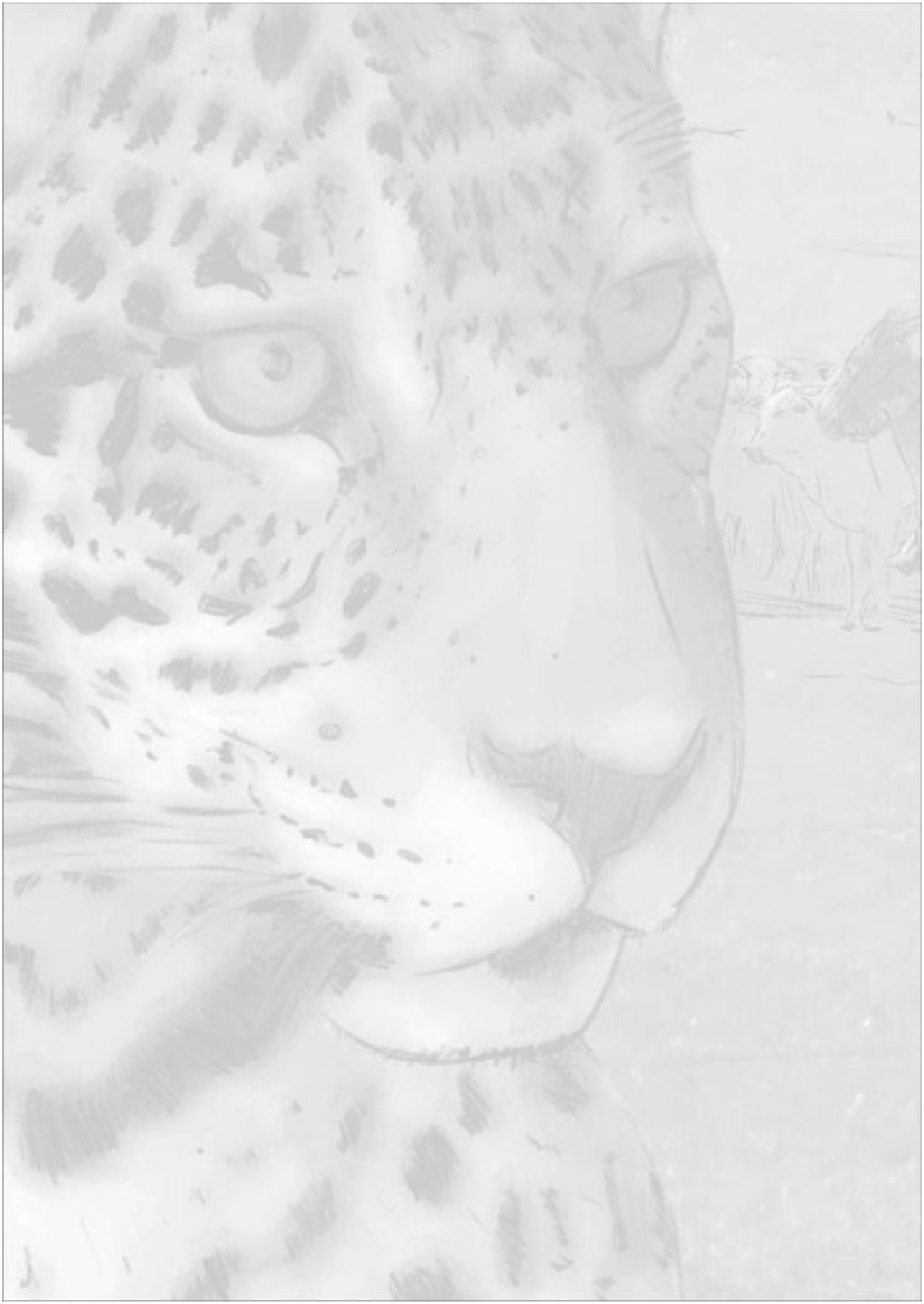

# PEOPLE AND JAGUARS: A GUIDE FOR COEXISTENCE


Text
**Silvio Marchini**

Illustration
**Ricardo Luciano**

English translation and technical revision
**Rafael Hoogesteijn, Howard Quigley and Andrea Heydlauff**


PEOPLE & JAGUARS COEXISTENCE PROJECT


A publication by
**Cristalino Ecological Foundation**
**Wildlife Conservation Research Unit**

First edition in English for internet use
published with the support of:

PANTHERA
PARTNERS IN WILD CAT CONSERVATION

Piracicaba, São Paulo, Brazil
June 2010

Copyright © 2010 by Amazonarium Press

**Author:** © Silvio Marchini
**Illustrator:** © Ricardo Luciano
**Translation and technical revision:** Rafael Hoogesteijn, Howard Quigley and Andrea Heydlauff
**Páginas:** 52

All rights reserved, No part of this publication shall be copied, reproduced or utilized by any means, without the previous authorization of the publisher.

**Graphics and Drawings:**
Ricardo Luciano

Dedicated to the children of Latin America. We hope that in the near future they can live in a world where the coexistence between people and jaguars is not a concern.

# Preface

Throughout their range, from northern Mexico to northern Argentina, unknown numbers of jaguars are killed every year because they are considered to be a threat to cattle and other domestic livestock. And every year, some livestock are either killed by jaguars or the death of livestock is blamed on jaguars. Until recently, there was little hope of changing this pervasive pattern.

We now have the opportunity to improve this situation for people and for jaguars. As you will see in the text that follows, there are many options for ranchers and farmers who are looking for ways to reduce the potential for jaguar conflict. In many areas, it is unlikely that the application of these methods will eliminate all livestock killing by jaguars. However, we can reduce it in ways that benefit both ranchers and livestock production. People and jaguars can live together. We know that.

Rafael Hoogesteijn has been a key force in bringing together technical information that provides a foundation for changing the paradigm of jaguar-human conflict (www.panthera.org/technical_manuals.html). Using more than twenty years of data, and experience from his own efforts and that of others, he has provided a user-friendly technical reference for those who want to reduce jaguar-livestock conflict. Silvio Marchini and Ricardo Luciano's publication has taken these efforts to the next level. They have produced an exceptionally readable reference for farmers, ranchers, and anyone interested in reducing jaguar-livestock problems.

Many may ask, why? Why do we need to change how we approach ranching? Why do we need to change the way we approach jaguars and other carnivores? We have proven that there is one extreme method by which we can eliminate the conflict between jaguars and people: eliminate the jaguars and their habitat. But, then, what do we have? How have we changed ourselves? What have we lost in losing the jaguar? When you walk through these areas, you find out.

The removal of jaguars from the human landscape is a loss to the biological and cultural heritage of the region. A land devoid of jaguars is a land devoid of some of its most powerful, natural energy that once kept life in balance. It is a loss to our own well being and to that of the world around us. Removal of the top predator is like pulling out one of the central pieces of a child's pile of building blocks; the structure destabilizes and either collapses or changes form. Jaguars are important components to the health of the world around us. Without a natural balance in place, we decrease the purity and function of our air, our water, and the products that the forest can provide. We now know that we can have livestock production, and still maintain jaguars and other living components of the forest, and maintain ecosystem health.

When we began our jaguar field research decades ago, in Belize and Brazil, we were confronted many times with the results of conflict between jaguars and people on ranches. We saw the dead jaguars and the dead cattle, and we heard the voices of people asking for help and for alternatives. In the pages that follow, the authors present some alternatives. And, we at Panthera will continue to build upon these efforts, providing research, information, training, and assistance to continually improve the situation for both the jaguar and the people of the jaguar.

**Alan Rabinowitz**
CEO, President, PANTHERA

**Howard Quigley**
Director, PANTHERA Western Hemisphere Program

www.panthera.org

The coexistence of people and jaguars constitutes a challenge. Jaguars are not welcome in rural areas and their presence can be intolerable for many people. Ironically, this intolerance is caused by the similarity between jaguars and ourselves, human beings. Jaguars and humans are approximately the same size; we both eat meat, and, therefore, we contend for the same prey species, wild or domestic. The most common argument for not wanting to coexist with jaguars is the fact that they feed on what should be exclusively people's food: domestic cattle. Another similarity between jaguars and humans is that both are formidable predators. Jaguars are admired for their hunting skills, but feared for their ability to defend themselves or attack humans, which very rarely happens. In reality, it is us, humans, who persecute and kill the jaguars. And as a result, jaguars are disappearing.

In this Guide, we accept the somewhat difficult task of promoting the coexistence of people and jaguars. We present information on how jaguars live and why they come into conflict with people; we suggest practical measures to prevent jaguar depredation on livestock and show that the damage caused by jaguars is small when compared to the devastating impact that we have on them. In addition, we present many of reasons why we can and should coexist with jaguars. With this guide we seek to reduce the intolerance and eliminate some prejudices toward jaguars, in the hope that people and jaguars will share the forests, savannas and grasslands of our countries.

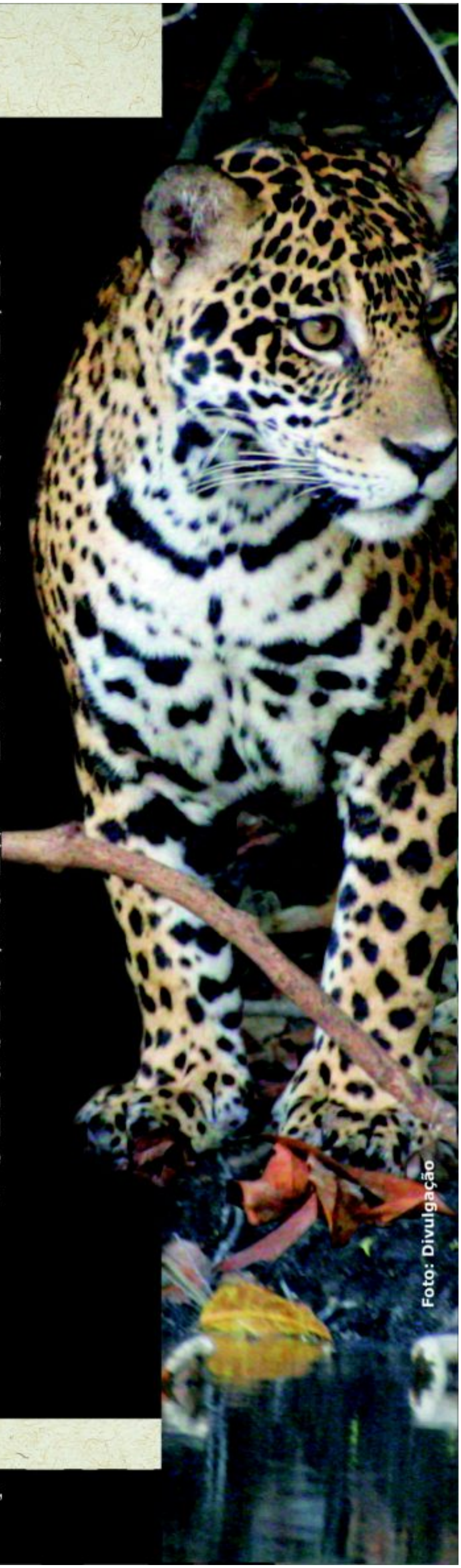


Young jaguar at the margins of the Cristalino River, in southern Amazonia.

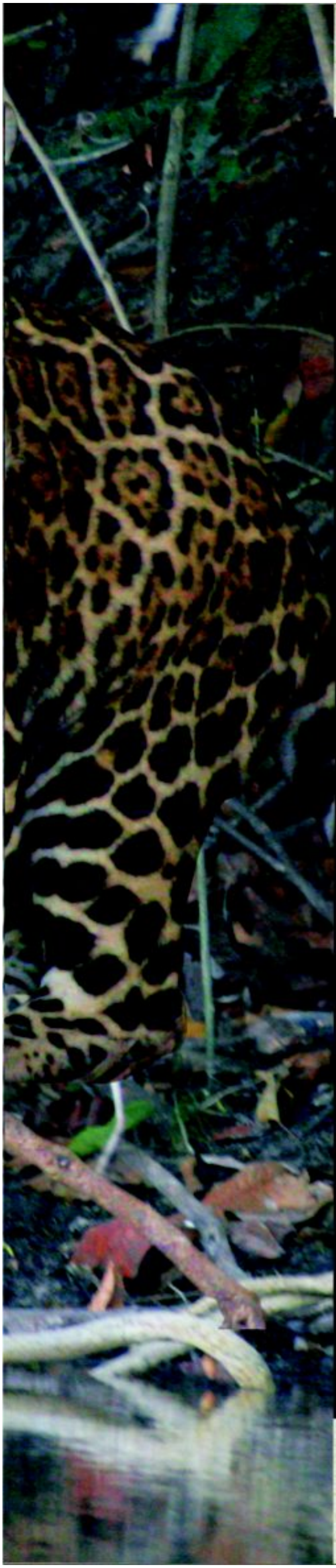

# People and Jaguars
# A Guide for Coexistence

## Index


**10** **JAGUARS:** What they are and how they live

**22** **JAGUARS:** Are they a problem?

**26** **JAGUARS:** We are the problem

**30** **REASONS TO** Coexist with jaguars

**32** **HOW TO** Coexist with jaguars

**38** **BEYOND COEXISTENCE:** Jaguars are interesting and fun

**44** **PEOPLE AND JAGUARS** Coexistence project

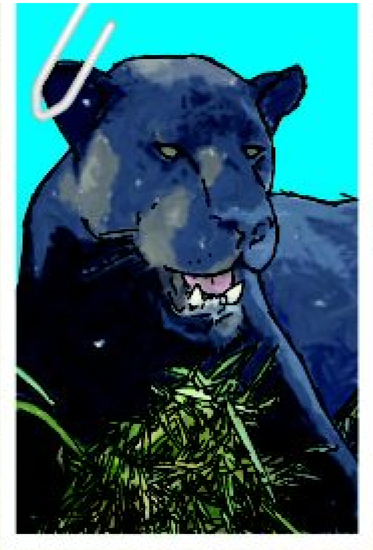

# Jaguars:
## what they are and how they live

The jaguar[1] (scientific name *Panthera onca*) is the largest terrestrial carnivore of Latin America and the third largest feline of the world (after the Asiatic tiger and the African lion). Adult jaguars can reach a total length of 2.2 to 2.5 meters, from the nose to the end of the tail, and can attain 80 centimeters in height. The largest jaguars are found in the Pantanal region of Brazil, where males weigh 100 kilograms (kg) and the females 76 kg, on average; males up to 150 kg have been found in the Pantanal. In forested areas like the Amazon region and Central America, jaguars are smaller.

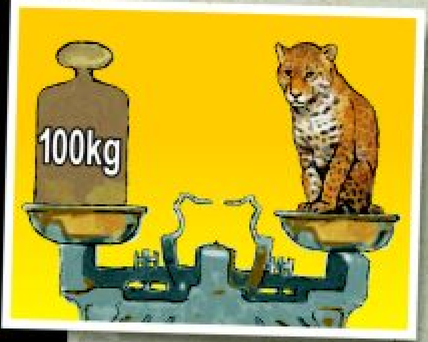


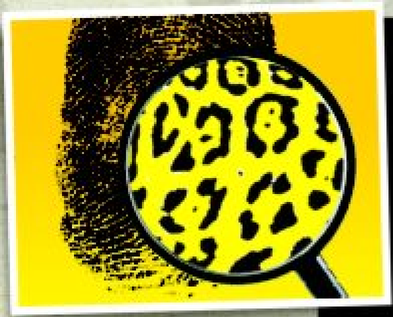

The jaguar's spots are certainly its most prominent visible feature. On the head, nape, and tail, the spots are solid (full), while at the sides of the body, or flanks, they form rosettes, with openings in the central part of the spots. The largest rosettes have one or more smaller spots inside. The pattern of spots and rosettes on each jaguar is variable, and can be used for individual identification, as a sort of fingerprint. The spots prominently display against a clear background that can vary from black or dark brown to a very clear, bright yellow, the most common color, though this background can be even a reddish brown in some variations. In the throat, belly, and internal parts of the legs, the background color is white. The spotted pattern of the jaguar looks similar to that of the leopard of Africa and Asia (where it sometimes can be called panther), but the leopard has no rosettes with spots inside, and the leopard is lighter in body weight, with a less robust structure.

[1]In Brazil, the jaguar is called "onça-pintada" and the puma "onça-parda" or "suçuarana".

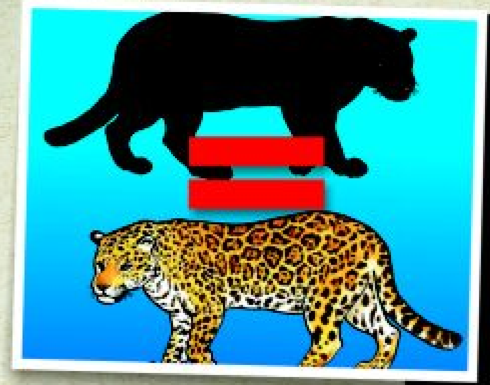

The yellow-background and the black-background jaguars belong to the same species, and they interbreed, giving birth to normal cubs that can be yellow or black. The reason for the difference between these two forms lays in the quantity of melanin (black pigment) of their hair. That's why the black jaguar is also known as a "melanistic form". There is no evidence that black and yellow jaguars behave differently; though many believe that one or the other form is more dangerous, brave or shy, that one form attacks more cattle, and so on.

The jaguar can be confused with the puma, but they belong to different species. The puma (scientific name: Puma concolor, also known as mountain lion, cougar, Florida panther, and catamount) is smaller and has a less stout body. Also, the puma cannot roar like the jaguar[1], but produces sounds more like screams, chirps, and meowing.

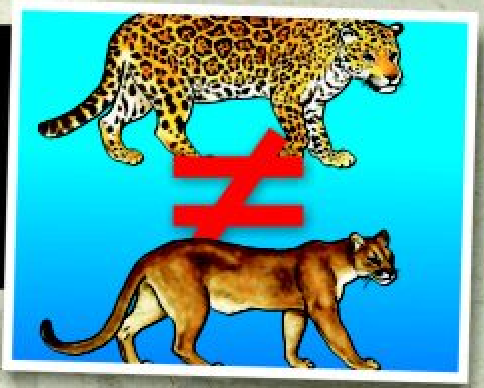

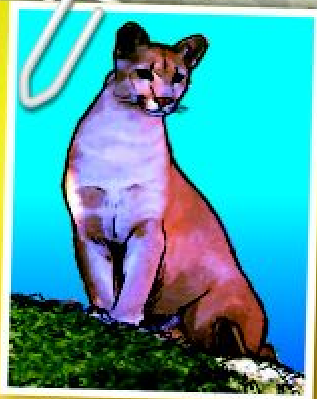

## Puma

Pumas are lean and agile felines. They are very adaptable generalists, and that's why they are found in all the principal biomes of the Americas, from northern Canada to Patagonia. Adult males can attain 80 cm height at the shoulders, and approximately 2.4 meters in length, measured from the nose to the end of the tail; but they can vary between 1.65 and 2.75 meters. The puma's size is smaller in tropical regions near the equator, and larger in the colder regions. Males have an average weight of 53 to 72 kg and in very rare cases can go up to 120 kg in the two extremes of their geographical distribution. The female's average weight lies between 34 and 48 kg.

[1] To hear the sound of jaguars and pumas, visit People and Jaguars Coexistence Project's website **www.amazonarium.com.br/onca.**

It's even easier to confuse the tracks of these two species: jaguars and pumas; but with some practice, it's possible, with confidence, to distinguish the two. The track of the jaguar is generally bigger, the toes are rounded, and the track is a little wider than long; The track of the puma is generally smaller overall, and little longer than wide, with the ends of the puma's toes more pointed (similar to those of the domestic dog, but without marking the nails).

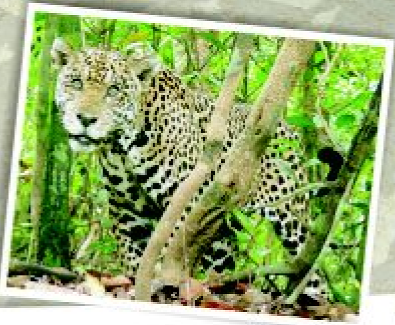

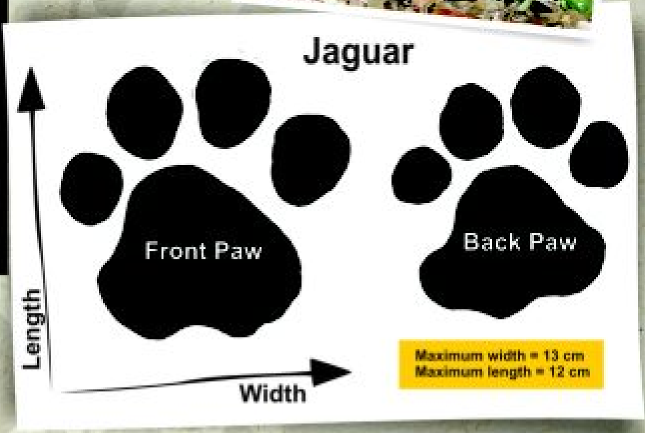


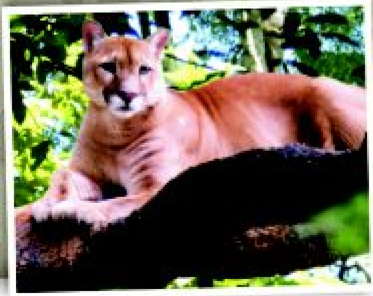

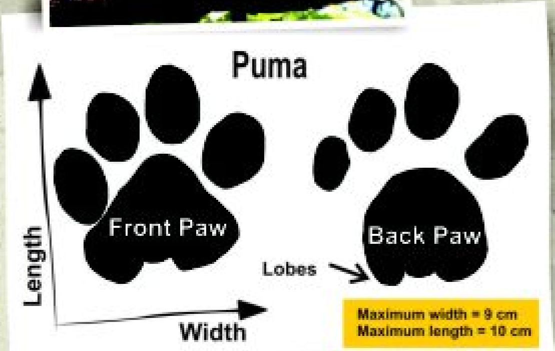


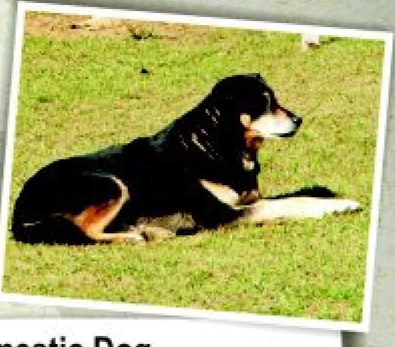

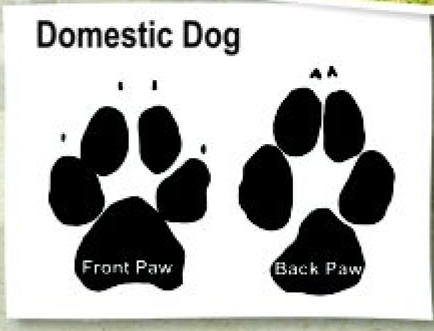

Jaguar cubs are blind for up to two weeks after birth; they begin to eat meat at two and a half months of age; and, they suckle maternal milk until approximately their third month; at some point, probably between 4 and 6 months of age, they begin to leave the den and accompany their mother. Cubs will stay with their mothers for one-and-a-half to two years of age. Female jaguars can begin to reproduce between two and three years of age, and the males between 3 and 4 years, after establishing a home range. Females can have litters of up to four cubs, after a pregnancy of 90 to 115 days; in the majority of cases, however, they have two cubs. In the wild, jaguars can live up to 15 years; thus, normally, a female will produce no more than 10 to 12 cubs in her lifetime. In captivity, jaguars are known to live up to 23 years.

**Example of a line of life of a female jaguar**

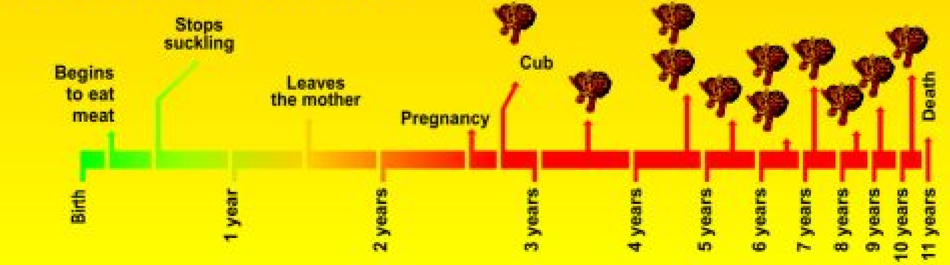


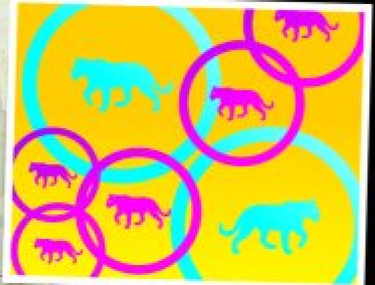

As a general characterization, jaguars are solitary species, adults meeting only for mating. Males will roar to advertise their area and reduce contact with other males; urine and feces also help claim an area. Jaguar home ranges vary in size. In Central America, a jaguar home range may be no larger than 35 square kilometers (3,500 hectares or 8.650 acres), while in the Pantanal on the other hand, males will use and defend territories up to 100 square kilometers (10,000 hectares or 24,700 acres). These areas can be nearly exclusive and non-overlapping with other males, or they can have some degree of overlap, depending on the area, the resources available, or the population studied. Female home ranges tend to be about half the size of a male's and more overlapping with neighboring females. The home range of a male may overlap the ranges of two or three females.

As large predators, jaguars prefer large prey. Their natural prey includes tapirs, different species of deer, two species of peccaries, capybaras, anteaters, sloths, and caiman. The jaguar also feeds on any small animal it can capture including monkeys, armadillos, paca, birds, turtles, tortoises, toads and even fish.

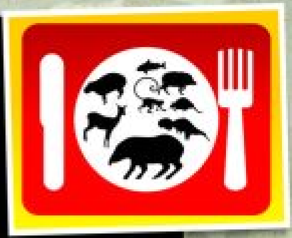

## Examples of prey species of jaguars

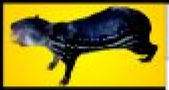
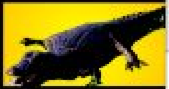
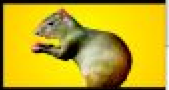
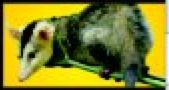
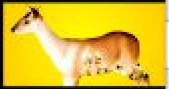
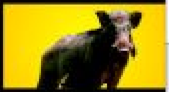
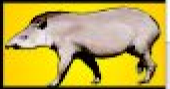
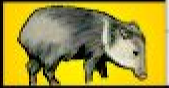
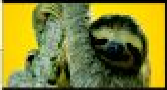
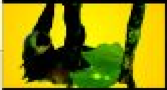
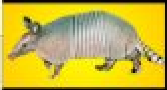
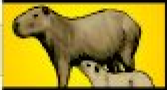
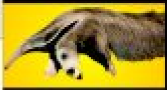
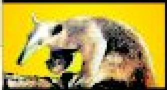
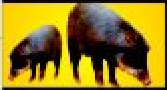

| Scientific name | English name |
|---|---|
| *Agouti paca* | Paca |
| *Bradypus sp.* | Three toed sloth |
| *Caiman sp.*[1] | Caiman |
| *Choloepus sp.* | Two toed sloth |
| *Dasyprocta sp.* | Agouti |
| *Dasypus sp.*[2] | Armadillo |
| *Didelphis sp.* | Opossum or possum |
| *Hydrochoerus hydrochaeris* | Capybara |
| *Mazama americana* | Red brocket deer |
| *Mazama gouazoubira* | Grey brocket deer |
| *Myrmecophaga tridactyla* | Giant anteater |
| *Sus scrofa* | Feral hog |
| *Tamandua tetradactyla* | Lesser anteater |
| *Tapirus sp.* | Tapir |
| *Tayassu pecari*[3] | White lipped peccary |
| *Tayassu tajacu*[4] | Collared peccary |
| *Nasua nasua* | Coati |

[1] *Most consumed prey in the Pantanal, Brazil*

[2] *Most consumed prey in the Cockscomb Basin, Belize*

[3] *Most consumed prey in the Pantanal, Brazil and in the Corcovado National Park, Costa Rica*

[4] *Most consumed prey at Hato Piñero, Venezuela*

Being an opportunistic hunter, jaguars can also eat domestic stock, mainly cattle, when available.

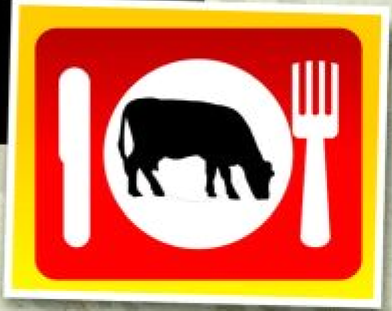

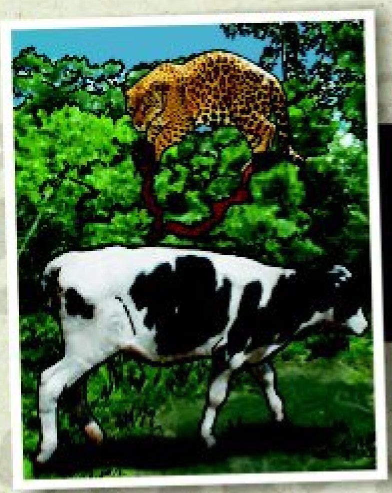

Jaguars rarely use a chase to secure their prey, but move as close as possible to their prey without being perceived, and then pounce on its victim. The ambush capacity of the jaguar is considered exceptional in the animal world. The attack can even happen in water, and the jaguar is able to swim carrying its prey.

As the other large felines (lion, tiger, leopard, and puma), the jaguar can kill its prey with a suffocating bite to the throat. But they frequently use a unique an efficient method of biting the nape of the animal, severing the spine, or even biting the head directly and puncturing the brain case.

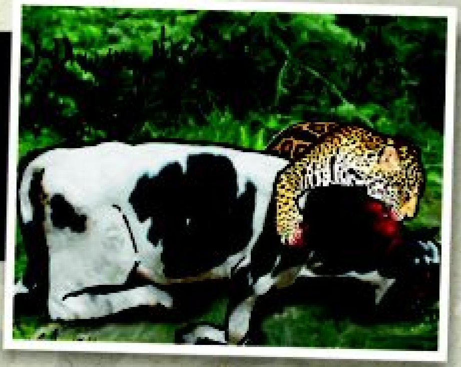

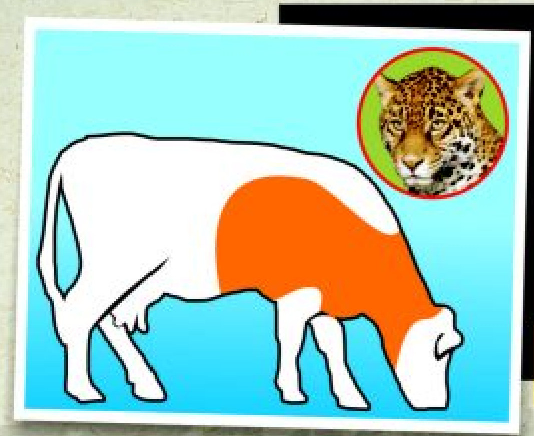

Once prey is captured and killed, the jaguar often drags it to a place with closed vegetation. The jaguar usually consumes first the forequarters of large prey, choosing the meat from the neck, chest, shoulders, and ribs, spending several days consuming the same prey. Calves and smaller prey can often be entirely eaten.

The preferred prey body areas consumed by the jaguar are marked in orange.

## Puma

The puma, on the other hand, prefers the hindquarters and the ribs, and also the liver, heart, and lungs of its prey. In addition, the puma covers (caches) its prey with leaves and other vegetation scraped over the carcass from the immediate area.

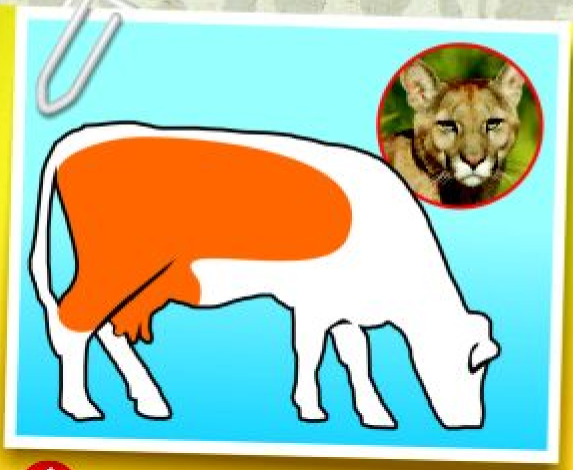

The preferred prey body areas consumed by the puma are marked in orange.

## Other predators

Dogs can cause a lot of damage to domestic animal breeders, killing or injuring animals of small to medium size. Domestic stock attacked by dogs, show numerous bytes, mainly in the hindquarters, legs, ears, and muzzles.

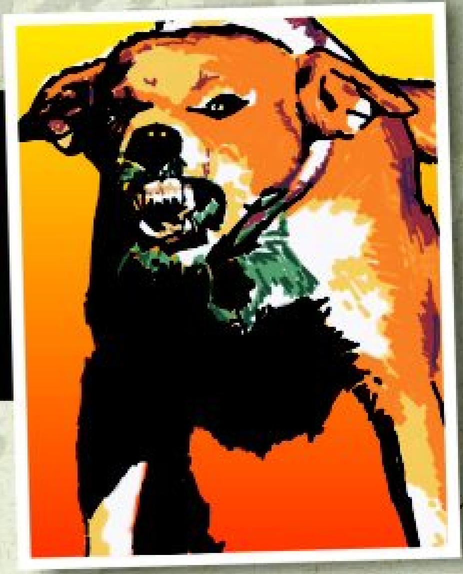

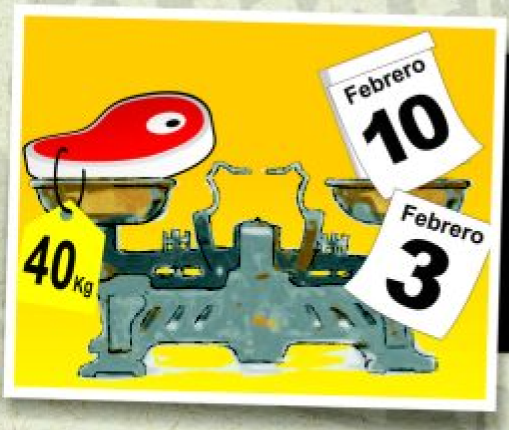


Nevertheless in the wild, the jaguars do not eat every day and spend a lot of energy capturing their prey. In fact, a jaguar will alternate periods of gorging and fasting, eating up to 25 kg of meat in one meal. On the average a jaguar eats between 35 to 40 kilos per week.

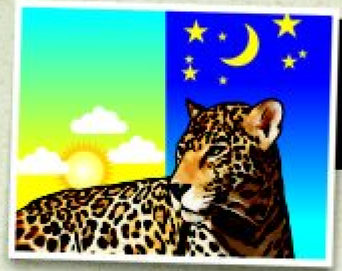

Jaguars are very active animals that can hunt during day and night, but they show more activity after sundown and before sunrise.

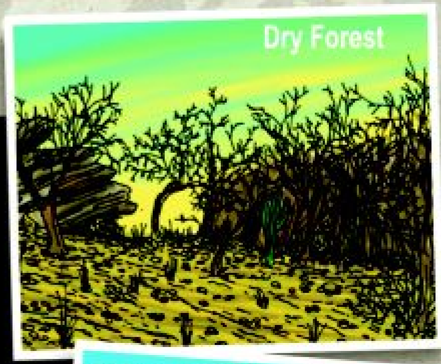


The habitat of the jaguar includes humid forests like the Amazon and the coastal forests, forested savannas (like the Cerrado in Brazil, and the Chaco in Bolivia and Paraguay), and areas seasonally flooded like the Pantanal (Brazil, Bolivia and Paraguay), the Llanos (Venezuela and Colombia), and the Beni (Bolivia). In other countries like Argentina, México, and the United States, jaguars also live in drier environments. They can be found in mountains up to 3,800 meters elevation, but normally prefer areas with ample availability of surface water, like river margins and flooded areas with dense vegetation, where they can ambush and surprise their prey.

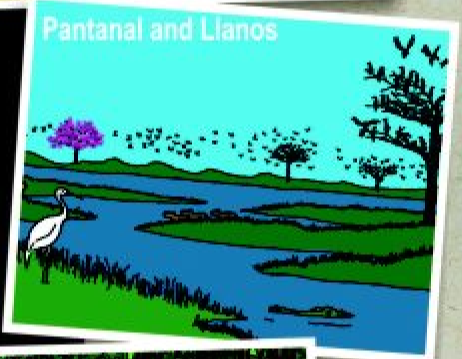


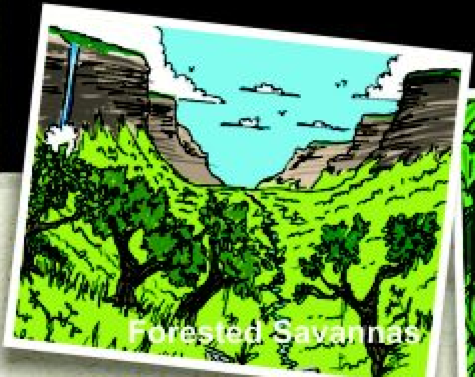


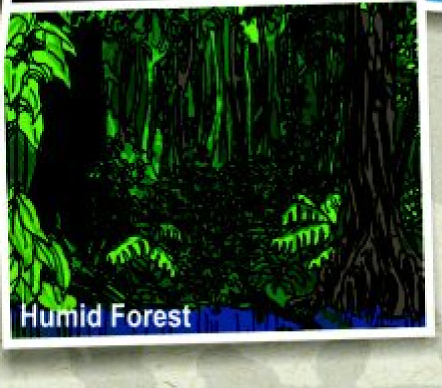

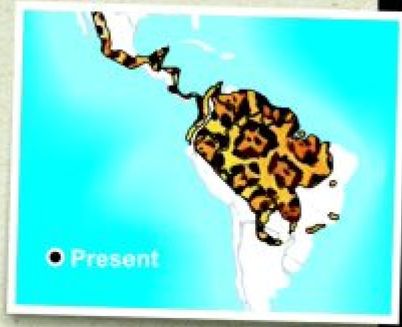


The present distribution of jaguar populations extends from Mexico, through Central America and South America to northern Argentina, including Amazonia. The jaguar is present in the following countries: Argentina, Belize, Bolivia, Brazil, Colombia, Costa Rica, Ecuador, French Guiana, Guatemala, Guyana, Honduras, Mexico, Nicaragua, Panama, Paraguay, Peru, Surinam, United States and Venezuela. The jaguar is extinct in El Salvador and Uruguay.

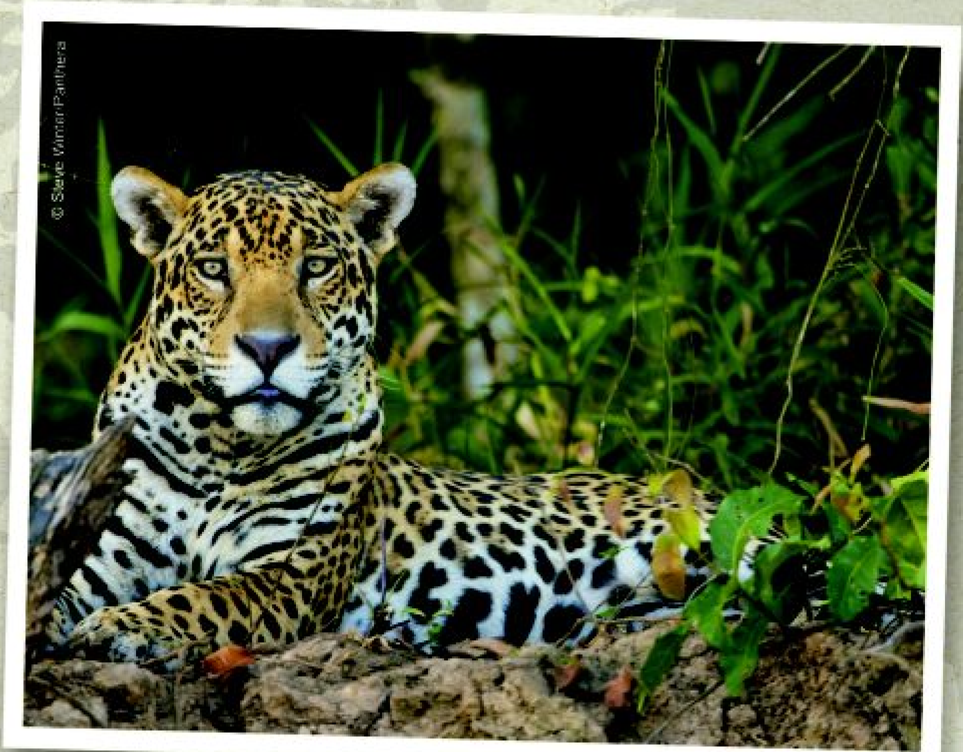

© Steve Winter/Panthera

# Jaguars: what they are and how they live

The jaguar (scientific name Panthera onca) is the largest terrestrial carnivore of Latin America and the third largest feline of the world (after the Asiatic tiger1 and the African lion). Adult jaguars can reach a total length of 2.2 to 2.5 meters, from the nose to the end of the tail, and can attain 80 centimeters in height. The largest jaguars are found in the Pantanal region of Brazil, where males weigh 100 kilograms (kg) and the females 76 kg, on average; males up to 150 kg have been found in the Pantanal. In forested areas like the Amazon region and Central America, jaguars are smaller.

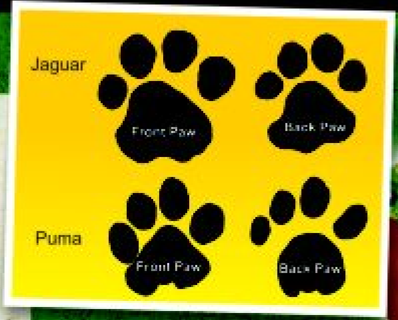


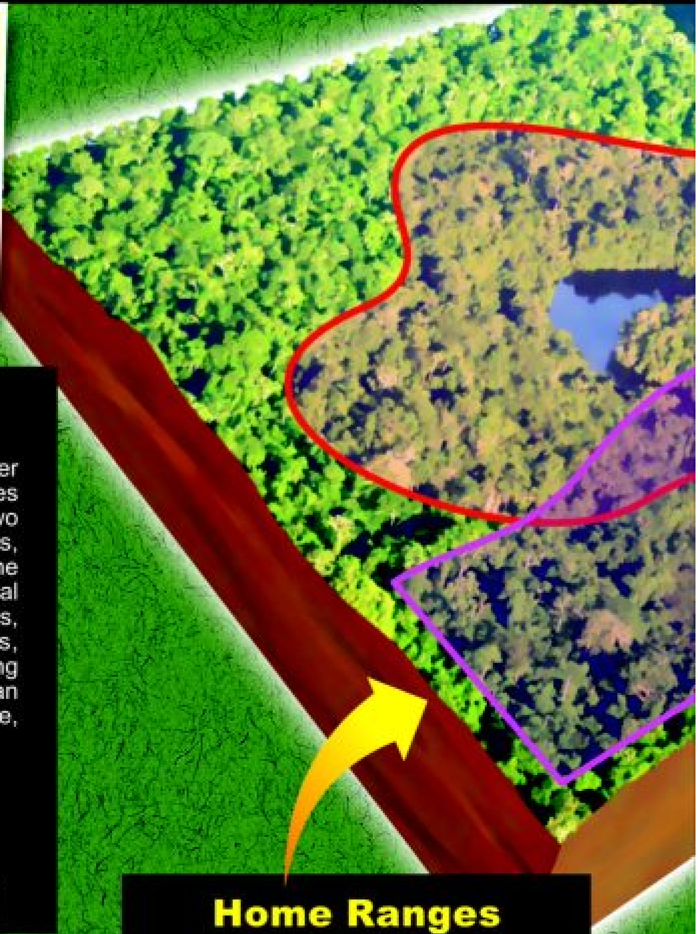

## Alimentation

As large predators, jaguars prefer large prey. Their natural prey includes tapirs, different species of deer, two species of peccaries, capybaras, anteaters, sloths, and caiman. The jaguar also feeds on any small animal it can capture including monkeys, armadillos, paca, birds, turtles, tortoises, toads and even fish. Being an opportunistic hunter, jaguars can also eat domestic stock, mainly cattle, when available.

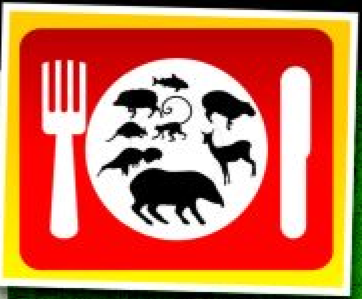

## Home Ranges

- Male 100 km2
- Female A 52 km2
- Female B 49 km2
- Female C 50 km2

Hypothetic home ranges and distributions of jaguars in the Pantanal (Brazil). In other regions, home ranges are smaller.

PROYECTO CONVIVIR GENTE & JAGUARES

## Habitat Use

Jaguar's habitat include forests and forested savannas, preferring areas with abundant water availability, as river margins, and flooded areas with dense vegetation where prey can be ambushed.

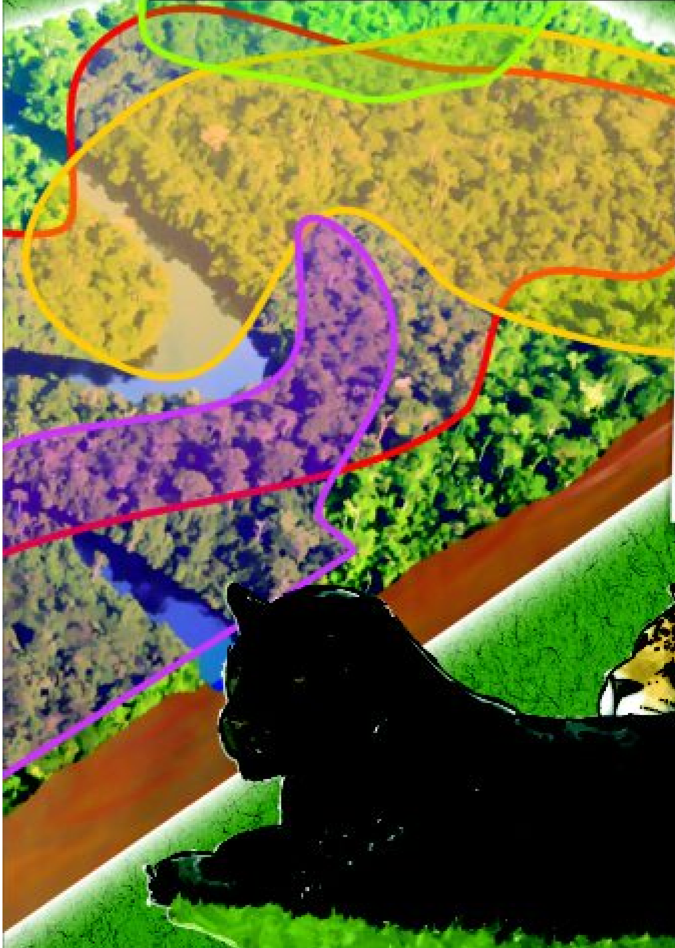

## Geographical distribution

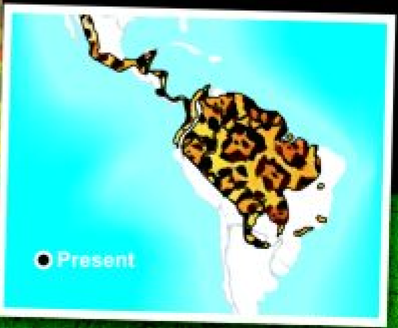


## Reproduction

Jaguar cubs are blind for up to two weeks after birth; they begin to eat meat at two and a half months of age; and, they suckle maternal milk until approximately their third month; at some point, probably between 4 and 6 months of age, they begin to leave the den and accompany their mother. Cubs will stay with their mothers for one-and-a-half to two years of age. Female jaguars can begin to reproduce between two and three years of age, and the males between 3 and 4 years, after establishing a home range. Females can have litters of up to four cubs, after a pregnancy of 90 to 115 days; in the majority of cases, however, they have two cubs. In the wild, jaguars can live up to 15 years; thus, normally, a female will produce no more than 10 to 12 cubs in her lifetime. In captivity, jaguars are known to live up to 23 years.

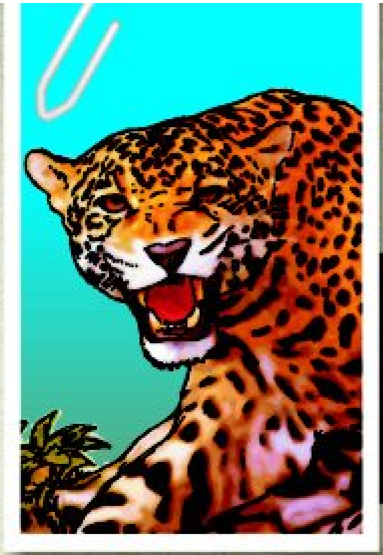

# Jaguars:
## are they a problem for us?

Contrary to the rare, but well-documented, cases that have happened with lions, tigers, and leopards, jaguars don't eat people. There are cases of jaguars attacking people, but the majority has happened in hunting situations, in which the jaguar was cornered or shot at. Jaguars can also attack to defend their cubs or their prey.

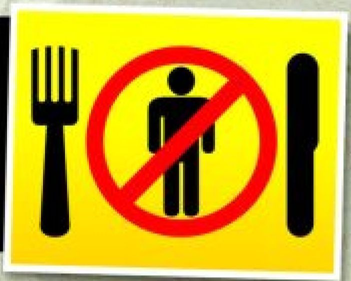

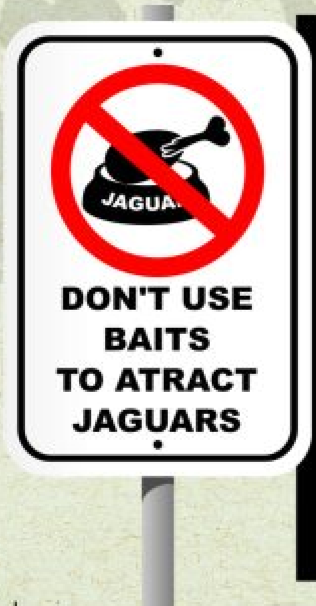


Inadequate ecotourism practices also can increase jaguar attack risks. The use of baits to attract jaguars to river mouths or beaches, and other open areas where they can be more easily observed by tourists, can habituate jaguars to human presence. That means that they lose their natural fear for humans. And worse, this practice can lead jaguars to associate the presence of people with food. This combination of loss of fear and association of people with food can provoke an attack on human spectators. Even when the encounter with a jaguar in the wild can be the best award for an eco-tourist, and the use of baits can increase the (typically remote) possibility of an encounter, this practice can result in disastrous consequences (for both parties), and has to be rejected and strongly prohibited.

**WARNING: What to do in a close encounter with a jaguar?**

- Stay calm
- Never give your back to the feline, or run away
- Without taking your sight off of the animal, or looking at the ground in front of him, begin to walk slowly backwards, until you get to a certain distance where you can go on your way. The jaguar will probably do the same.
- If there are children in the group, put them behind you (so that there always will be an adult between the child and the jaguar) and follow the same previous recommendation

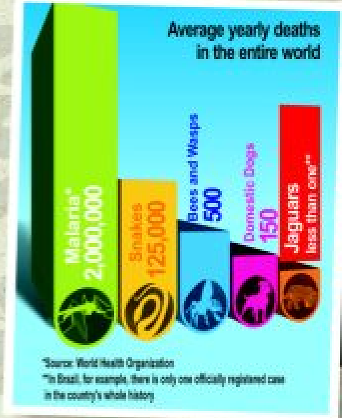


Before getting worried about a jaguar attack, consider the danger represented by some other species. Consider first that one of the animals that kills more people in the world is the apparently inoffensive mosquito. Even domestic dogs, deserve more worry than jaguars, since each year dozens of people are killed by them. Documented cases of humans killed by jaguars in Brazil in particular and in Latin America in general are very rare.

## Puma

There are more registered human attacks by pumas than by jaguars. There are more than 100 confirmed attacks on humans in North America since 1890. Fifty of those incidents happened since 1991. Twenty of those resulted in death of the victim. Of the twenty mortal victims, twelve were children 13 years of age or less. In Brazil, the only fatal attack registered, happened in Amazonia in 1992, and the victim was a child. Nevertheless, compared with the animals of the figure mentioned above, even pumas represent an insignificant danger for man.

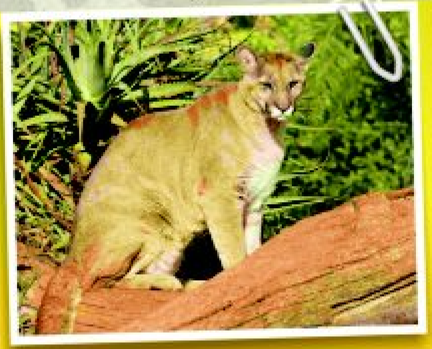

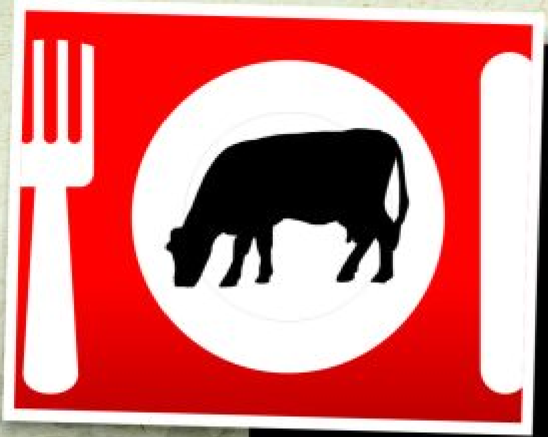

Jaguars kill domestic animals. And where they do – especially where there are chronic problems – these areas are strongly associated with areas where natural prey has been diminished by deforestation, associated to wood extraction, hunting and the advance of agriculture and introduced pastures. These activities increase the proximity between jaguars and domestic animals and decrease the potential for jaguars to encounter natural prey.

Although in some isolated cases jaguars can cause a high degree of damage to cattlemen, studies done at the Pantanal, southern Amazonia, and in the western state of Paraná (all of them in Brazil), and also in the Llanos of Venezuela, have shown that, on average, one or two animals for every 100 head of cattle were lost due to jaguar attacks.

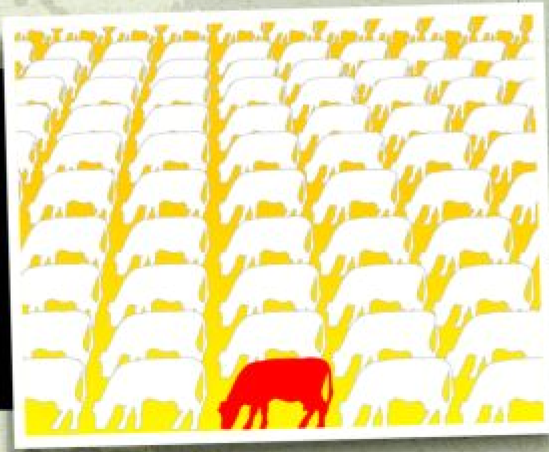

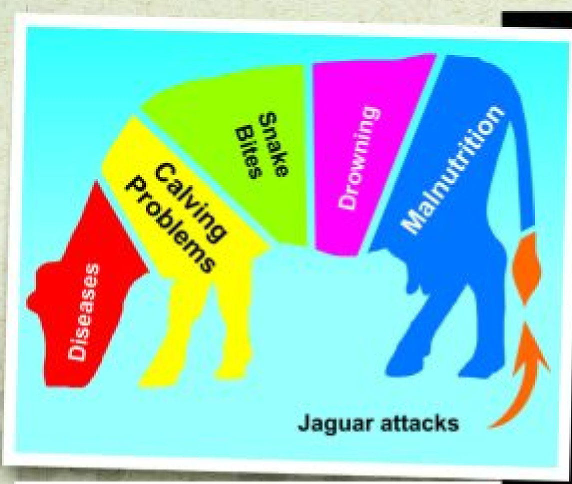


Again, it is important to see things in their proper context. For a herd owner of 100 cattle, though the loss of one calf looks large, there are other general causes of mortality such as diseases, calving problems, snake bites, drowning, malnutrition (and in many countries we also have to include cattle rustling or theft), that cause much greater losses to cattle producers than jaguar predation. So, to enhance cattle productivity in general, it makes more sense to invest in improving management practices than to invest in eliminating jaguars.

**Note:** Learn to avoid the problem of jaguar attacks on domestic stock in the chapter **"How to coexist with jaguars"**

## Puma

As is the jaguar's case, pumas can also prey upon domestic stock, and this appears most probable when their natural prey is scarce. Domestic stock such as goats, sheep, and pigs are most vulnerable to pumas, and they may kill many sheep in one attack. Herds rounded up and penned up at night near human habitation suffer less risk, since pumas avoid human proximity. In cattle raising areas, where pumas share the habitat with jaguars, pumas tend to kill smaller calves (up to 6 months of age), while jaguars generally kill larger calves and adult cows. It is very important that stock owners and managers learn to distinguish puma from jaguar attacks: occasionally the jaguar is persecuted, when the problem is actually caused by the puma.

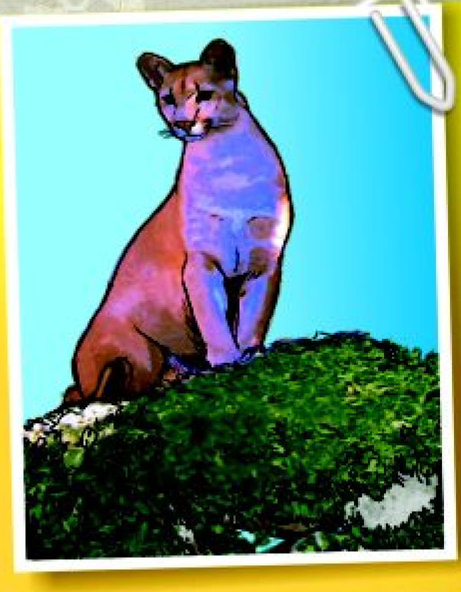

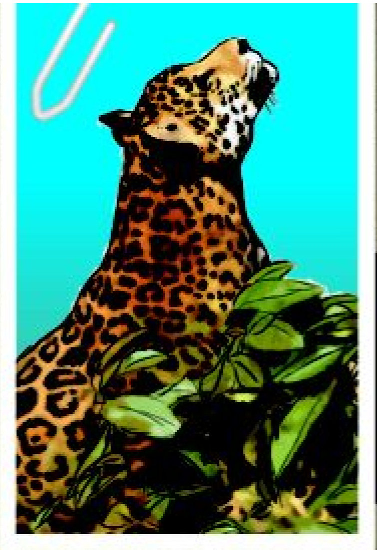

# Jaguars:
## we are the problem

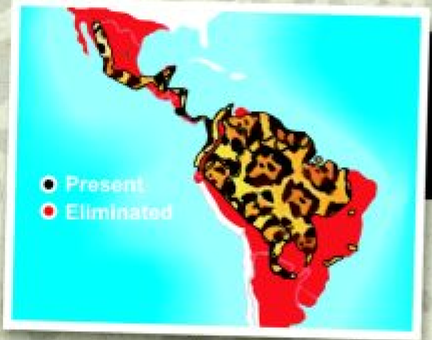


Jaguars are disappearing. They already have been eliminated in large areas of many countries, and their distribution and abundance continues to diminish rapidly.

One of the principal threats for jaguars is habitat loss; jaguars are losing the environment in which they prefer to live. The principal habitat of the jaguar is the forest. Latin American countries loose immense areas of tropical forests each year because of the deforestation for agricultural and mining. Some countries, like El Salvador, have eliminated so much jaguar habitat that they have not significant populations of jaguar left. Brazil, as another example, looses a larger area of tropical forests per year than any other country in the world. The largest portion of that loss occurs in the so called "Arc of Deforestation", stretching from the state of Pará to the state of Acre, passing through northern Mato Grosso and Rondonia. Regulating deforestation is an important factor in conserving jaguar populations, but the loss of habitat continues, as does the loss of jaguars with it.

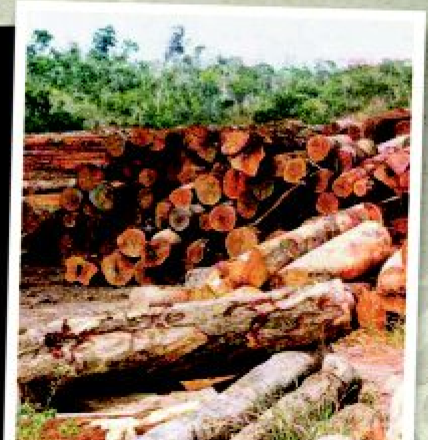

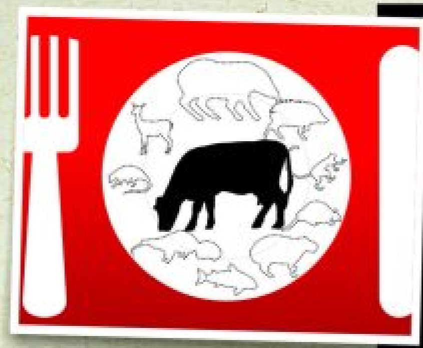

Another important threat for jaguars is the disappearance of their natural prey, by loss or modification of habitat, and also through over hunting. In many regions where jaguars still exist, people hunt peccaries, tapir, and deer, the preferred prey of jaguars. In some cases, because of the lack of natural prey, jaguars turn more commonly to cattle as a feeding alternative.

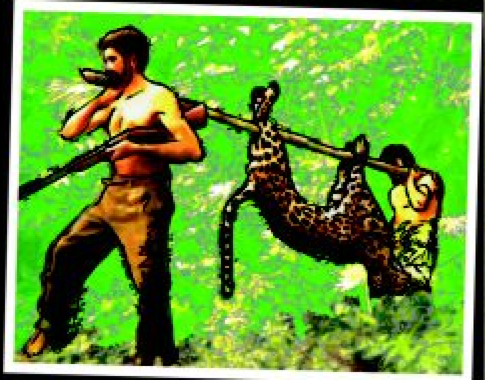

The indiscriminate, direct killing of jaguars also contributes hasten the disappearance of this felid. The persecution of jaguars is usually associated with the predation of domestic stock. Ranchers will kill – or mandate the killing of – jaguars that simply show up on their properties or are directly associated with losses in their cattle herds. However, the decision between killing the problem jaguar, taking management actions with the herd to solve the problem (see them in "How can we live with jaguars"), or simply tolerating the loss, depends on a series of socioeconomic and cultural reasons. In addition, jaguars are also killed even when they do not threaten cattle. The fear of these cats, the sport and entertainment of the hunt, and/or the demonstration of bravery by confronting a powerful animal, are also reasons why some people kill jaguars.

## Puma

Pumas are also threatened by the loss of forests and decreases in natural prey populations, but mainly because of the persecution by rural livestock and agricultural producers. Puma populations have stabilized or even increased in some areas of North America due primarily to direct population management, control of hunting, and habitat protection; they continue to decrease in Latin America because of the advance of the agricultural frontiers.

# Jaguars:  we are the problem

Jaguars are disappearing, they already have been eliminated in large areas of many countries, and their distribution and abundance continues to shrink rapidly.

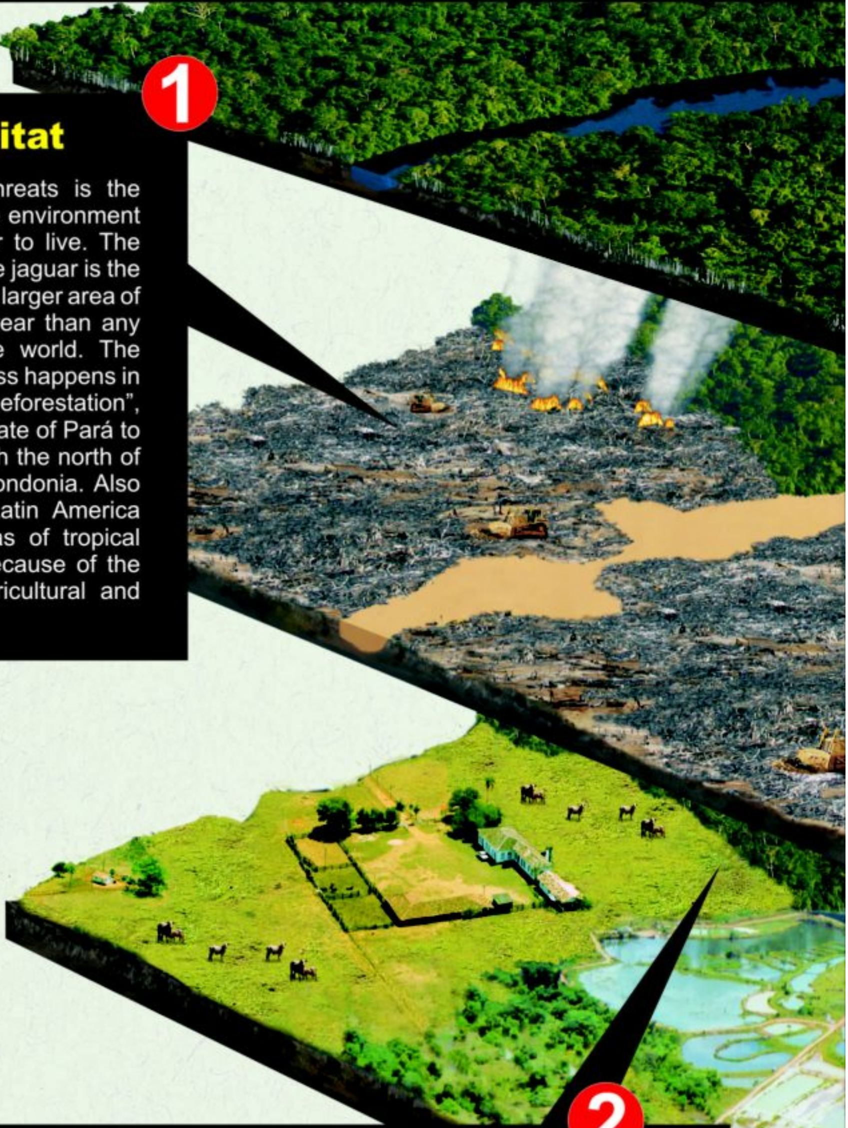

## Loss of Habitat

One of the main threats is the disappearance of the environment where jaguars prefer to live. The principal habitat of the jaguar is the forest. Brazil looses a larger area of tropical forests per year than any other country in the world. The larger share of that loss happens in the so called "Arc of Deforestation", that goes from the State of Pará to Acre, passing through the north of Mato Grosso and Rondonia. Also other countries in Latin America loose immense areas of tropical forests each year because of the deforestation for agricultural and mining purposes.

## Disappearance of their natural prey species

The disappearance can happen by loss or modification of habitat, and also because of hunting. In many regions where jaguars still subsist, people hunt peccaries, tapirs and deer; that are also the preferred prey species of jaguars. In some cases because of the lack of natural prey, jaguars turn to predate on cattle, as a feeding alternative.

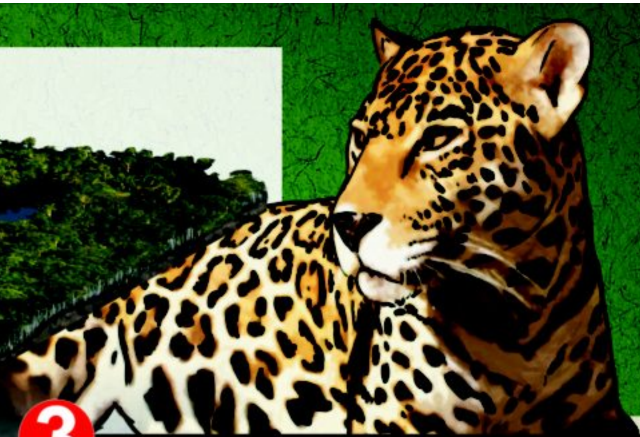

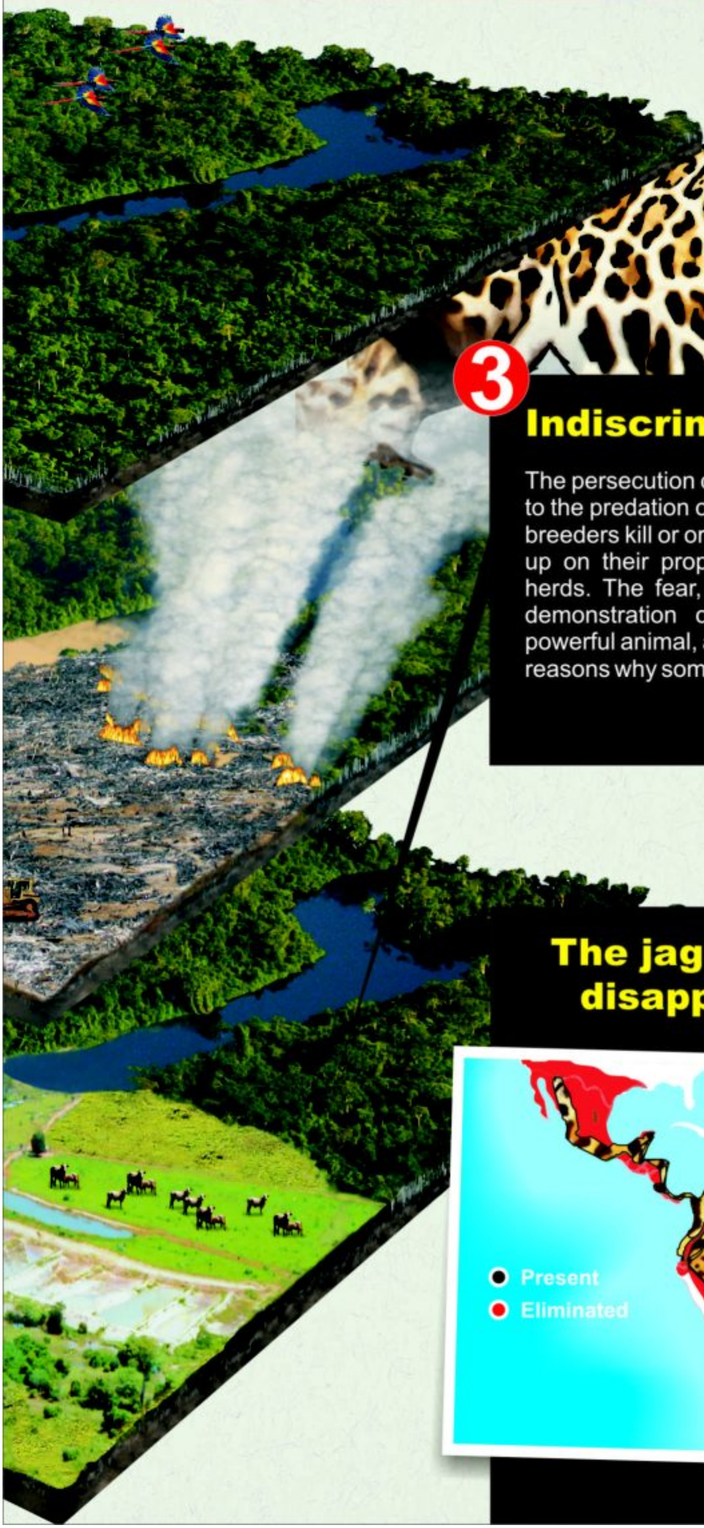

## 3 Indiscriminate killing

The persecution of jaguars is usually associated to the predation of domestic stock. In fact, some breeders kill or order to kill the jaguars that show up on their properties or menace their cattle herds. The fear, entertainment of the hunt or demonstration of bravery by confronting a powerful animal, able to defend himself, are also reasons why some people kill jaguars.

## The jaguars are disappearing

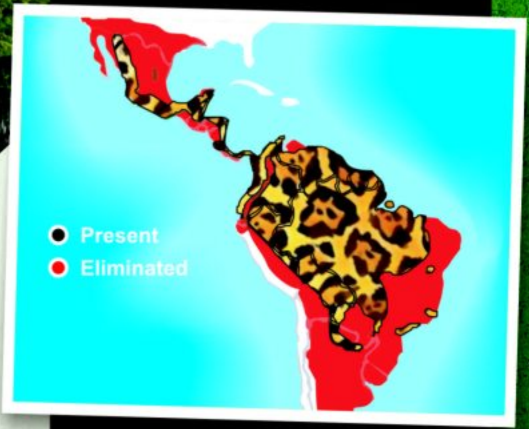

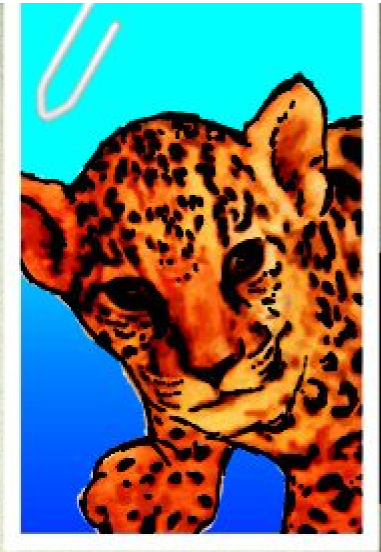

# Reasons to coexist with jaguars

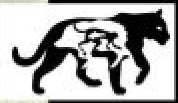

**Ecological reasons.**

The jaguar is the largest terrestrial predator of Latin America and feeds on a diversity of other animals, including species that feed on leaves and seeds like deer, tapir, pacas and agoutis, and carnivores like foxes and raccoons. Feeding on these species, jaguars "control" their populations; that is, they impede prey population increases to over abundance. Foxes and raccoons in turn, control their prey populations, rats, crabs, and snails; in the same way, leave and seed eaters control the plant populations on which they feed on. Thus, through a "cascade effect", jaguars have an ample influence over the forests where they live, imparting affects on natural communities, from the insects to the trees. Also, even when it has not been scientifically proven yet, it is possible that jaguars help to diminish the transmission of diseases between different animal species and also from these species to man, since they prey more easily on ill or weak individuals.

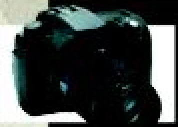

**Economical Reasons.**

The jaguar is one of the more beautiful and fascinating species of the Latin American wildlife. For this reason, its image is used for commercial purposes, especially by the tourism industry. For example, in the state of Mato Grosso (Brazil), in the posters and booklets of tourism advertisement, produced by the State Tourism Office and by tourism agencies, the jaguar is the animal most often shown (with the probable exception of the jabiru stork). In fact, jaguars can contribute to tourism, even when the chance of seeing them is remote. Commonly, a larger number of tourists is likely to pay more for the occasion of observing or hearing a jaguar, or simply for the experience of being in jaguar territory, or even for the opportunity of contributing to the conservation of this species through tourism. Some cattle ranches in the Pantanal (Brazil) are now receiving larger revenues from eco-tourism (with the jaguar as flagship species) than from their cattle operations.

**Legal Reasons.**
Killing jaguars is illegal, and killing a jaguar is a crime in jaguar range countries, as defined by their environmental laws. For example, in Brazil, article 29 of the Brazilian Law for Environmental Crimes states, it is illegal to "kill, persecute, hunt, capture, utilize native or migrating specimens of wildlife, without the proper licenses, permits or authorizations given by the competent authority or in disagreement to the one obtained: penalty of detention of six months to one year and fine."

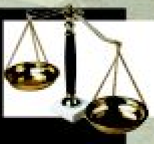

**Cultural Reasons.**
The jaguar is an icon of Latin American culture. In practically all the indigenous Latin American cultures, the jaguar occupies a special position as another powerful human or as a messenger to the gods. Being a predator of extremely good hunting skills, like humans, he possesses a peculiarly human cunning and shows up in the myths of almost all of these cultures as the living symbol of the Gods and of their power and their strength. The jaguar symbolizes cunning, vigor, speed, and above all, this felid is used to express the power of nature. For the modern man, the jaguar contributes to cultural manifestations in their most varied forms, from local crafts to children tales; from paintings that portray the history of the country to the 50 Reais bank notes in Brazil. For capturing the attention and interest of children and grow-ups, the jaguar is the starting point of educational and communication activities for the conservation of nature.

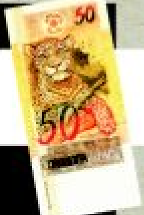


**Emotional Reasons.**
Felids always have ignited a great fascination in people. Being the largest felid in the Western Hemisphere, and also because of their exceptional beauty, jaguars awaken emotions ranging from admiration to fear, from fascination to rage. No other wildlife species elicits such strong and contrasting feelings. In addition, it is a part of human nature to give value to diversity in all its dimensions, be it material, cultural, or natural. We are affectively attached to jaguars and, for the same reasons we restore our historical buildings and safeguard art pieces in museums, we want jaguars to continue to exist.

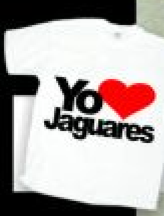


**Ethical Reasons.**
"Killing jaguars is wrong". "Push a species to extinction is immoral". More and more people are supporting these beliefs. Understanding the idea that it is not jaguars that are invading human space, but humans invading jaguar space, and understanding that jaguars don't cause damage on purpose or through malice, but they are simply following their predatory instincts, has helped bring about acceptance of the perception that killing jaguars is something wrong. In addition, and above all, the view that jaguars – like people – have an inalienable right to exist and persist in their natural ways would render immoral  actions that counter that right and seek to drive jaguars toward extinction.

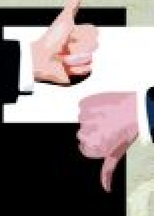

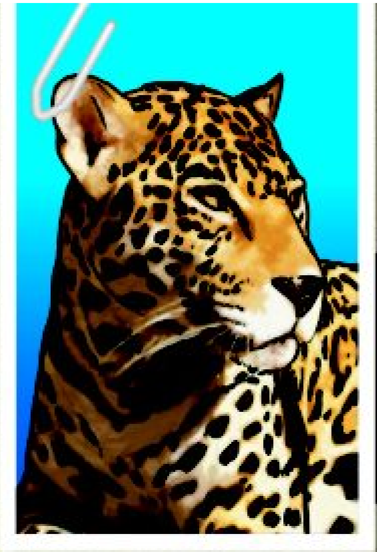

# How to coexist with jaguars

Perhaps the most effective way to stem habitat loss is through the creation of protected areas. The creation of protected areas (where human development, use, and impact is minimized) has traditionally been the role of the state or federal government, but rural landowners also can play a very important role. For example, in the case of Brazil, rural landowners must follow Environmental Legislation that mandates Permanent Preservation Areas and Legal Reserves be established in any farm or rural property; this also applies in establishing Private Natural Heritage Reserves (RPPN; www.ibama.gov.br/rppn).

**The following strategies can be applied for the prevention of jaguar (and puma) attacks on cattle and other domestic stock[1]:**

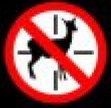

Don't hunt and don't allow any hunting of the jaguar's natural prey.

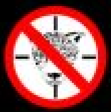

Don't hunt jaguars and don't allow the hunting of jaguars. Jaguar hunting produces individuals with physical limitations (such as broken teeth or mobility issues) that will diminish their capacity for capturing their natural prey, leading them to attack cattle.

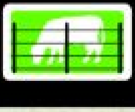

Build and locate fences to prevent cattle from forested areas.

[1] Hoogesteijn, R. and A. Hoogesteijn. 2005 Manual sobre problemas de depredación causados por grandes felinos en hatos ganaderos. Programa de Extensión para Ganaderos. Programa de Conservación del Jaguar, Wildlife Conservation Society. Campo Grande, Brazil (Spanish edition: ISBN 85-905237-2-1., Portuguese edition: ISBN 85-905237-1-3).

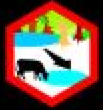

Build water sources (wells or lagoons) away from forested areas, if possible.

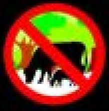

Don't hold late-term cows or cows with young calves near forested areas. These animals should be grazing in open areas, preferably near farm buildings and human activity.

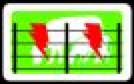

Use electrical fences around the maternity paddocks[2].

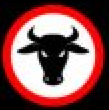

For the purpose of repelling felid attacks, the electrical fence final voltage has to stay over 4,500 to 5,000 volts, and the fence should have 3 strands of electrical wire at 20, 40 and 60 cm off the ground and one upper negative strand at 85 cm. This installation is easier to use in small paddocks or night enclosures (where the herds are penned at night). Large paddocks suffer problems of electricity supply. This is a technified system that functions well and is very effective, but requires attentive monitoring so maintenance issues can be resolved quickly.

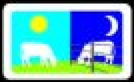

Maintain some horned, older, and experienced cows or steers in the herd; these older animals help instill grouping behavior in the herd that is effective in avoiding jaguar attacks. Bells can be hung from the necks of some tame animals as another helpful deterrent device.

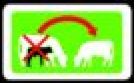

In extensive forested areas with high predation incidence, reunite the herds and pen them at night in a corral or enclosure near human habitations, in small paddocks, well fenced, or with an electrical fence (as described above). Despite a small increase in operation costs, this simple strategy is very helpful in reducing the impacts of predation (and, in addition, rustling, or cattle theft), and the animals easily adapt to this management; installation of lights in the night enclosures or corrals is also useful, as is the burning of some dry dung to keep away mosquitoes and biting insects, which tames and tranquilizes the enclosed herds.

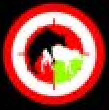

In properties with high predation incidence, replace cattle breeding operations with rearing or fattening operations. In this way, these areas will be utilized with animals above 1 to 2 years of age. Establish short breeding seasons of 3-4 months of length, instead of having breeding and calving year round. During the reduced calving season, it is very important to maintain intensive surveillance of the calving process and of the newborn calves. This managerial decision, will not only help manage and organize a productive cattle herd in an organized and predictable manner, but assist in predicting and managing all the important annual events (weaning, palpation, vaccinations, culling, sales, cattle selection, replacement quality, etc.) in a much more efficient way, enhancing the farm productivity. This action, combined with the calving paddocks with electrical fences and the night penning up of the herds, provide a very efficient strategy to reduce calf predation.

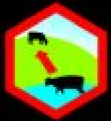

During wet season, move cattle from low, flood-prone areas, to higher grounds so they don't stay isolated and weakened by the flooding, making them more vulnerable to jaguar attacks (This is especially important in floodplain areas, such as the Llanos, Pantanal, and Beni).

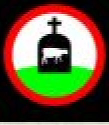

Dispose of the carcasses of domestic animals that died due to other causes (such as snake bites, calving problems and diseases) so they cannot be devoured by cats and promote the tendency to consume cattle.

[2] Scognamillo, D., I. Maxit, M. Sunquist and L. Farrell. 2002. Ecología del jaguar y el problema de la depredación de ganado en un hato de los Llanos Venezolanos. In: R.A. Medellín, C. Equihua, C. Chetkiewicz, P.G. Crawshaw Jr., A. Rabinowitz, K.H. Redford, J.G. Robinson, E.W. Sanderson and A.B. Taber (Eds.). El Jaguar en el Nuevo Milenio. Fondo de Cultura Económica, Universidad Autónoma de México and Wildlife Conservation Society.

In flooded areas with high incidence of predation problems, the introduction of small and well managed herds of milking water buffalo (of the Murrah breed), managed alone or in combination with cattle herds, has demonstrated to be an efficient way to reduce predation problems in many Venezuelan cattle farms[3]. It is important to note that buffalo need constant and intensive human handling, to prevent them from becoming feral, and creating an environmental problem. This is easily solved with good management; it is not a species problem. Also, buffalo in flooded environments have demonstrated higher productivity than cattle.

Be familiar with the appearance and signs of domestic animals depredated by felines, and know how to differentiate these from those losses caused by packs of wild or feral dogs, and by rustlers or cattle thieves.

Keep detailed records on mortality and its causes in the ranch's cattle registries and check the real losses and their causes, and compare the information from each year in terms of the percentages or mortality and its causes.

[3] Hoogesteijn, R. and A. Hoogesteijn. 2008. Cattle and water buffalo jaguar – related mortality – could water buffalo facilitate jaguar conservation and cost – effective ranching in the Neotropics? Oryx 42 (1), 132 – 138.
Hoogesteijn R. and A. Hoogesteijn. 2009. El búfalo de agua, eficiente solución a los problemas de depredación por felinos en las sabanas inundables del trópico suramericano. In: J. Salomón, R. Romero, J. De Venanzi and M. Arias. (Eds.). XXIV Cursillo sobre Bovinos de Carne. Universidad Central de Venezuela, Facultad de Ciencias Veterinarias, Maracay, Venezuela.

PEOPLE & JAGUARS · COEXISTENCE PROJECT ·

Even with the creation of conservation units and with the adoption of prevention strategies for reducing jaguar attacks on cattle, it is possible that a jaguar passes through a ranching area, or lives in a ranching area, and eventually kills and consumes domestic stock. This is the price we pay for coexisting with this fascinating animal. Therefore, this coexistence of jaguars and people requires tolerance from our side. In general, though, our tolerance increases as we learn more about the situation. The more we learn about jaguars – their behavior and their biology – the better we relate to them and understand how important they can be in our lives; with this; with this understanding comes tolerance in the relationship between jaguars and humans. Thus, information is fundamental for the coexistence of people and jaguars.

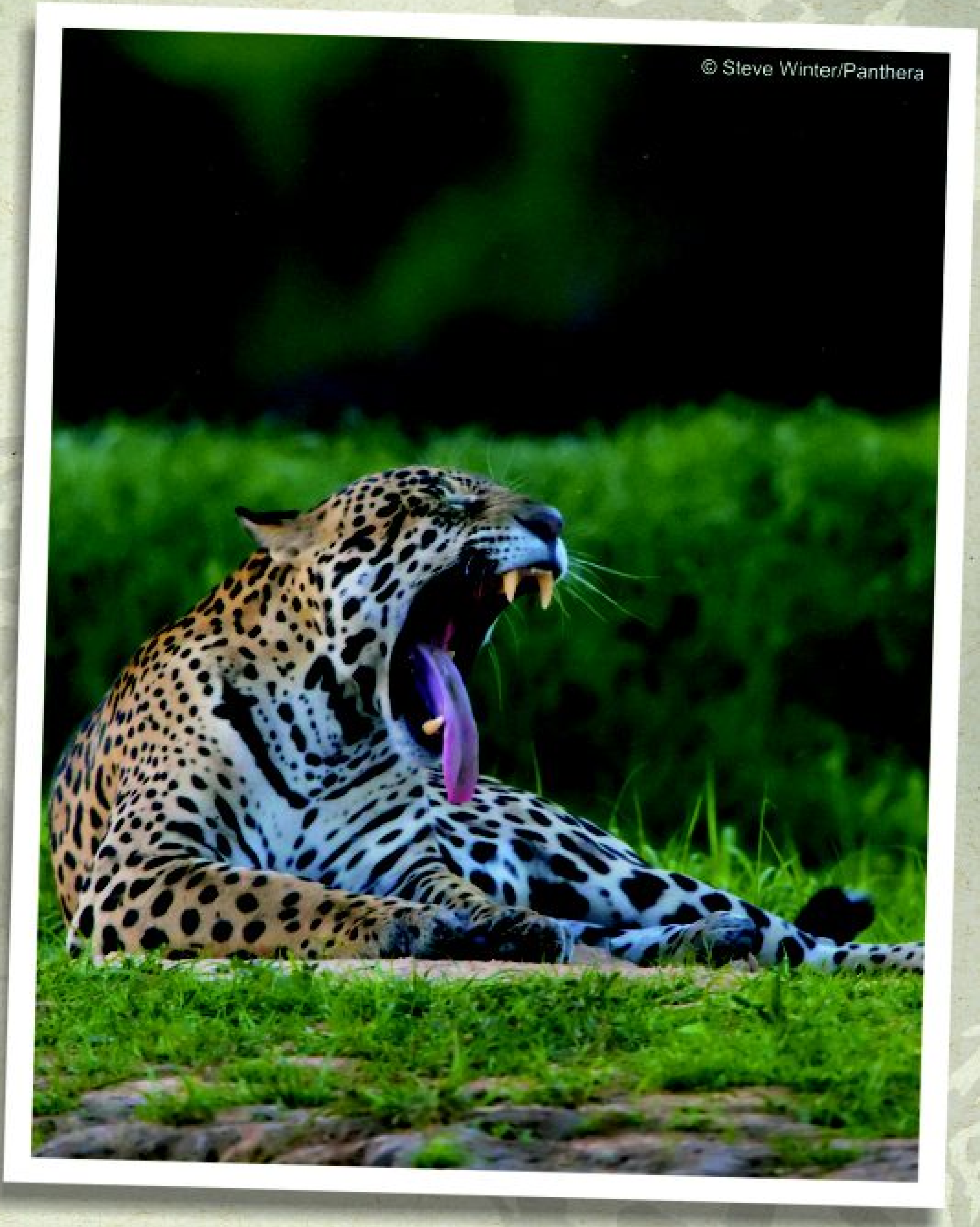
© Steve Winter/Panthera

# How to coexist with jaguars

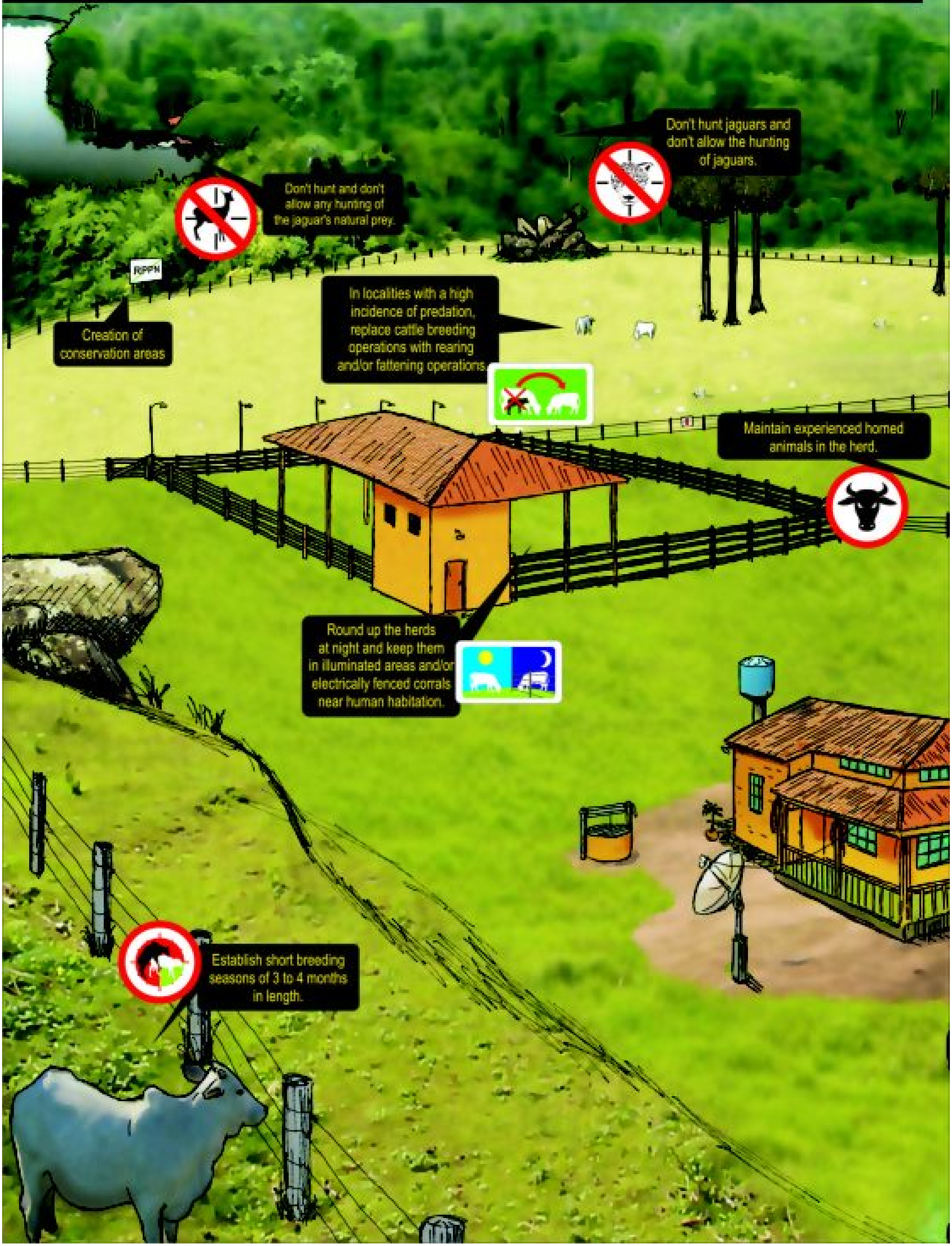

Protect the habitat and the natural prey species of jaguars, and follow the recommendations for the prevention of jaguar attacks on domestic stock. These are the actions that can lead to better coexistence between people and jaguars. But, even when these strategies are implemented, a jaguar may pass through a farm or ranching area, or live within the farm or ranch, and attack domestic stock. This is the price we pay for coexisting with this fascinating animal. Therefore, the coexistence of jaguars and people requires tolerance from our side. The more we learn about jaguars and the importance of their lives, the more tolerant we get in relationship to them. Thus, information is fundamental for the coexistence of people and jaguars.

PEOPLE & JAGUARS · COEXISTENCE PROJECT ·

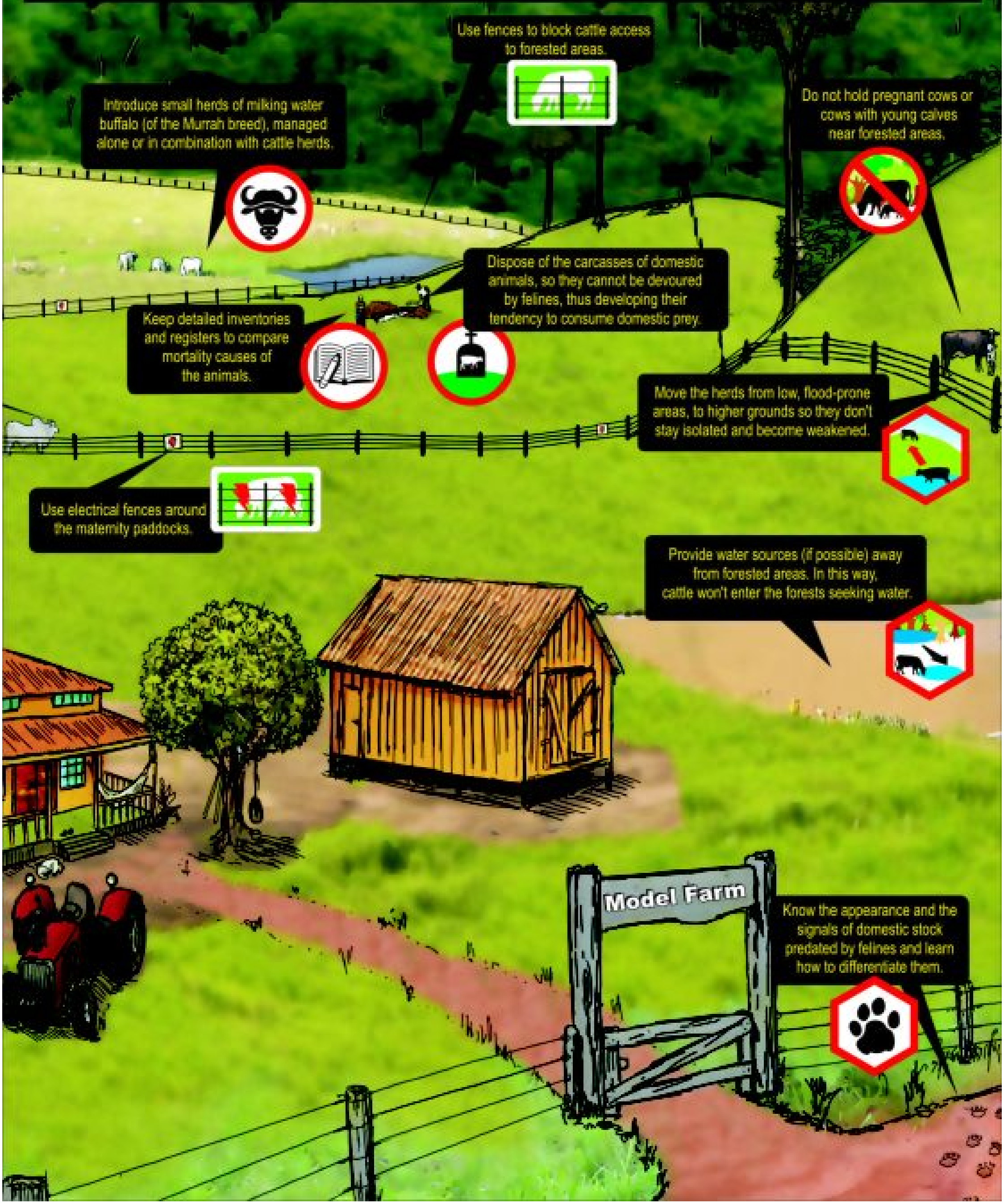

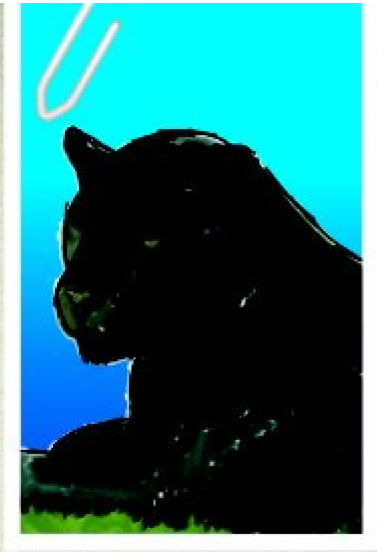

# Beyond coexistence:
## jaguars are interesting and fun

### Jaguars in Books

Hoogesteijn, R. and A. Hoogesteijn. 2005. Manual sobre problemas de depredación causados por grandes felinos en hatos ganaderos. Programa de Extensión para Ganaderos. Programa de Conservación del Jaguar. Wildlife Conservation Society. Campo Grande, Brazil (Spanish edition: ISBN 85-905237-2-1 and Portuguese edition: ISBN 85-905237-1-3). This is arguably the most important reference on human-jaguar conflicts.

Leite, M.R.P., T.G. Oliveira, R. C. de Paula and C. Indrusiak. 2002. Manual de Identificação, Prevenção e Controle de Predação por Carnívoros, IBAMA. Another important reference for the resolution of predation problems by jaguars.

Roosevelt, T. 1914. Through the Brazilian Wilderness. New York. Charles Scribner's Sons Describes the travels and explorations of the author through the forests and rivers of Brazil in the company of Colonel Cândido Mariano Rondón, describing also jaguar hunting forays in the Pantanal.

Medellín, R.A., C. Equihua, C. Chetkiewicz, P.G. Crawshaw Jr., A. Rabinowitz, K.H. Redford, J.G. Robinson, E.W. Sanderson and A.B. Taber (Eds). 2002. El Jaguar en el Nuevo Milenio. Fondo de Cultura Económica, Universidad Autónoma de México y Wildlife Conservation Society, México. Gathers the largest number of scientific articles on jaguars in one volume, presented at a scientific meeting of experts on jaguars from different Latin American countries.

Hoogesteijn, R. and E. Mondolfi, 1992. The Jaguar. Ediciones Armitano, Caracas. Venezuela. Spanish, English and German editions. Book that brings general information on jaguar biology, ecology and conservation, focused on cattle predation problems.

Rabinowitz, A. 1986. Jaguar: Struggle and triumph in the jungles of Belize. Arbor House, New York. Story of the creation of the first jaguar conservation preserve in Belize and of the foundation of a large history of research on the species in that country, by the author.

## Jaguars in Books

Siemel, S. 1953. Tigrero. Prentice - Hall. Story of a Letonian that lived in Brazil during the fifties, organizing safaris and spear-hunting jaguars in the Pantanal. English and German editions.

Duguid, Julian. Tiger-Man. 1932. The Century Company, New York, London. Book about the life of Sasha Siemel in the Brazilian Pantanal, and his jaguar hunting exploits.
Siemel, S. Jr. and E. O'Brien. 1965. Sashino. Sidgwick and Jackson Limited. London. Written by the son of Sasha Siemel, about his life in the Pantanal.

Almeida, T. 1976. Jaguar Hunting in the Mato Grosso. Stanwill Press, England. A not common classic, with a second enlarged Edition by Safari Press. Between jaguar's hunting histories in the Pantanal of Brazil and Bolivia, the author describes for the first time, details over the jaguar natural history and ecology. English and German editions.

## Jaguars in Magazines

**Terra da Gente.** "Face a Face com a Rainha", by Helen Sacconi. February 2009.

**Terra da Gente.** "Com a onça na mira" with photos and text by Adriano Gambarini. September 2005.

**National Geographic Magazine.** The famous North American Magazine has published numerous articles on jaguars. Among the most prominent:

Path of the Jaguar, by Mel White (March 2009)
Brazil' Wild Wet, by Susan McGrath (August 2005)
Phantom of the Night, by Douglas Chadwick (May 2001)
Cats: Nature's Masterwork, by Cathy Newman (June 1977)
The Jungle was my Home, by Sasha Siemel) November 1952)
King of Cats and his Court, by Victor Cahalane (February 1943)

## Jaguars on the Internet

**Panthera**
(http://www.panthera.org)
Description of projects, information on grants, publications and news on feline conservation.

**Wikipedia**
(http://en.wikipedia.org/wiki/Panthera_onca)
Encyclopedic information on jaguars.

**National Geographic**
(http://animals.nationalgeographic.com/animals/mammals/jaguar.html).
General information, maps of distribution range, multimedia.

**IUCN / Cat Specialist Group**
(http://lynx.uio.no/lynx/catsgportal/cat-website/catfolk/onca-02.htm).
Jaguar description

## Jaguars on the Internet

**Jaguar Conservation Network**
(http://www.jaguarnetwork.org)
Information on research projects, conservation and conflict resolution.

**Wildlife and People**
(http://www.peopleandwildlife.org.uk)
Conflicts between humans and wildlife, research projects, events, bibliographical references, conflict resolution manuals. In English.

**Carnivore Ecology and Conservation**
(http://www.carnivoreconservation.org/portal/index.php)
Bibliographical information source on carnivores, updated with a large number of scientific publications and complete thesis for access, meeting details, job advertisement, and ecological knowledge monitoring.

**Pró-Carnívoros**
(http://www.procarnivoros.org.br)
Information on Brazilian carnivores, research and conservation projects, courses and bibliographical references.

**Jaguar Conservation Fund**
(http://www.jaguar.org.br)
Information on jaguars and projects developed by the Jaguar Conservation Fund (Instituto Onça-Pintada).

## Jaguars in Documentaries and Videos

**YouTube.**
Numerous videos that show jaguars, including a rare shooting of a jaguar killing a capybara in the Pantanal.
http://www.youtube.com

**National Geographic.**
The documentary "In search of the Jaguar" can be found in Amazon.com. Short videos are available at the site of this organization. Look for
http://video.nationalgeographic.com/video/player/animals/index.html

**"Sasha Siemel, the Jaguar Hunter".**
Documentary by Cándido Alberto Fonseca, on the Letonian that spear-hunted jaguars in the Pantanal in the nineteen fifties. In Portuguese.

## To watch and photograph jaguars

**In the Wild.** Jaguars are extremely difficult to see in the wild. Their relative low abundance, shy and silent habits, preference for dense vegetation, and restricted movements in dark areas, all contribute to the jaguar being one of the least visible species of tropical wildlife. In Brazil, the regions with tourist infrastructure and the best opportunities to see jaguars are: The Araguaia River, The Northern Pantanal (specially in the second half of the Transpantaneira Road and along the Cuiabá River in Poconé, and the Paraguay River in Cáceres) and in the Southern Pantanal (in the hotel-ranches and Rivers of the Miranda and Rio Negro Region). Attention: In case you have the privilege of observing a jaguar, stay calm, and always at a safe distance.

**With "camera traps".** Camera traps are photographic cameras coupled to infra-red light sensors that shoot automatically when an animal comes close to the camera. The camera sits ready, waiting for days along a trail or road for the passing of a jaguar to be photographed, while you stay at home. Cameras can be conventional or digital. In the latter case, they can be programmed to film animals that pass in front of them, instead of photographing. Two brands are the North American Camtrakker (www.camtrakker.com) and the Brazilian Tigrinus (www.tigrinus.com.br), but others are also available.

**In Captivity.** There is a large number of jaguars in various zoo collections in principal Latin American cities, open to public visit. If you would like to see a live jaguar, the most practical and guaranteed way of doing so is to visit the zoo nearer to you, although the experience never will be the same as seeing the animal in its own environment.

## To see jaguar signs

**Tracks.**
Finding tracks of jaguars is much easier than seeing the animal itself. Jaguars like to walk along trails and roads, leaving ample evidence of their passing. The best occasion to find tracks is in the first days after a rain, when the sand or soil surface is softer and more homogeneous. Sandy or muddy beaches of rivers and lagoons are also good places to look for tracks.

**Collecting tracks.**
You can collect jaguar (and also other species) tracks, building a gypsum cast of the track. To do so, you are going to need the following materials:

Cardboard strips (60 cm x 5 cm)
Paper clips
Water
Gypsum powder
Plastic container (plastic bottle cut in half)
Spoon
Gardener shovel
Old tooth brush

*Continues*

## To see jaguar sign

Select a sharp fresh track and put the cardboard strip around, forming a cylinder and secure it with the clip. Bury the cardboard slightly in the mud or sand. Prepare the gypsum, putting a little bit of water in a recipient, adding the gypsum powder and mixing it slowly with the water until it gets a creamy consistency. It has to be filled carefully in the cylinder to prevent the inclusion of air bubbles. Let it dry for 15 to 20 minutes. With the little shovel unbury the gypsum mold with the mud around. Wrap it up in newspaper and take it home. Wait one day for the gypsum to solidify. After the mold is hardened, take away the cardboard and remove the mud or soil with the toothbrush.

**Urine and Feces.** Jaguars urinate over the vegetation at the edge of roads and trails. Urine is expelled in a shower, covering a relatively large area. The jaguar's depositions can also be found in trails. They are cylindrical and measure between 3 and 4 centimeters in diameter. The strong smell and the presence of hairs, nails, feathers, hooves and bone fragments are typical of jaguar's feces.

**Scratches.** Jaguars can scratch tree trunks, their principal feature of which is that the distance between the claw marks is homogeneous and measures between 2 and 4 centimeters. Scratches can be deep and large and can attain more than 2 meters off the ground.

**Prey remains.** Natural or domestic prey showing scratches as described above, or holes produced by robust canines (more often in the nape or the lower and posterior part of the cranium), are signaling the presence of jaguars. These felines normally drag their prey to areas with dense or closed vegetation and prefer to eat the front-end of the body first.

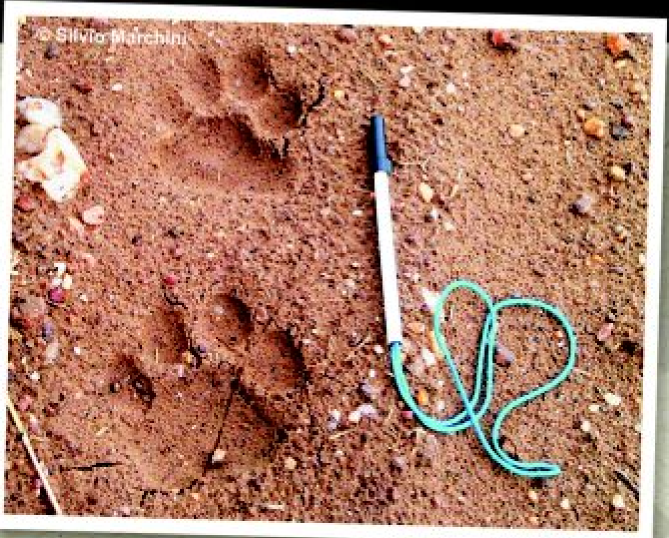

## To hear jaguars

**In nature.** Hearing a jaguar in the forest is an unforgettable experience. Male jaguars roar or "snore" ("roncar" and "esturrar" are the terms used in many areas of Latin America to define the jaguar roar) as an advertisement of their presence. And they will often respond to the sound of another roar. In Latin America, hunters sometimes use rustic instruments (called "chorunos" in Bolivia), or calabashes and empty gourds (called "esturradores" in Brazil and "corotos" in Venezuela), to emit the amplified roars through them. These instruments and also any roar recording (playback) can be used in the intent of hearing a jaguar in the wild. However, they have to be used with caution, since a male jaguar can believe that another roaring male is challenging him in his own home range and can get close with aggressive intentions.

**In the Internet.** There is a more practical way of hearing a jaguar roar, like using the internet. Some sites have the reproductions of the sounds of the roars. The site Projeto Conviver Gente e Onças is one of them: **www.amazonarium.com.br/onca.**

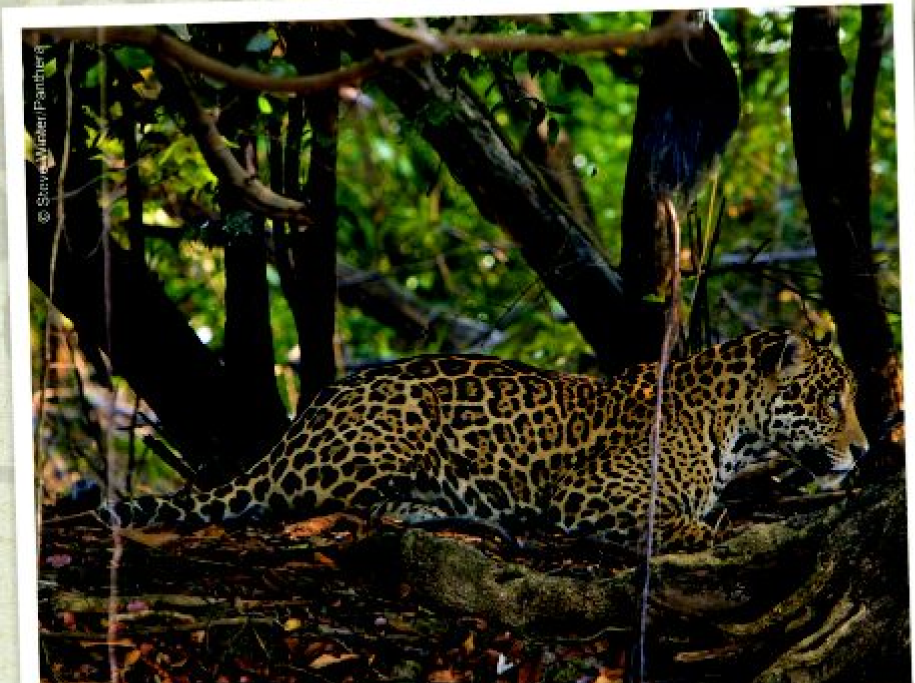

# People and Jaguars Coexistence Project

Ultimately, the major reason to persecute and kill jaguars is not the threat they represent for our domestic stock or for our personal safety. Rather, it is the perception people have of jaguars as a menace. In general, in our relation with the large carnivores, the perception of the threat is worse than the real threat.

The People and Jaguars Coexistence Project has examined the perceptions people have of jaguars: the way this perceptions develop in children and teenagers and how they translate in the persecution and killing of jaguars by cattlemen. The project, conducted in Brazil, evaluated landowners and their families in the agricultural frontier of Amazonia, the Pantanal, and the Atlantic coastal forest. Based upon the results, the project has designed and implemented education and communication campaigns to increase tolerance to jaguars.

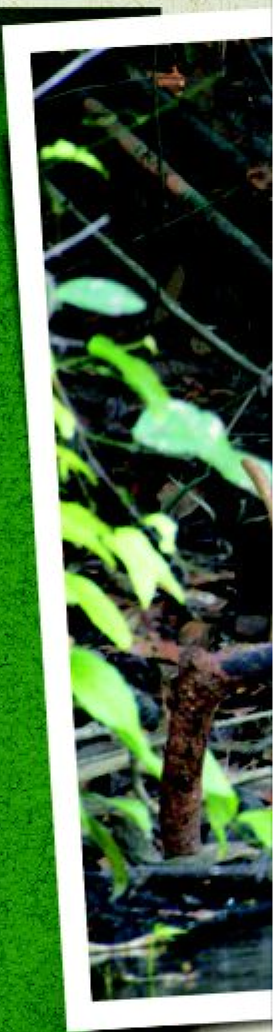

PEOPLE & JAGUARS COEXISTENCE PROJECT

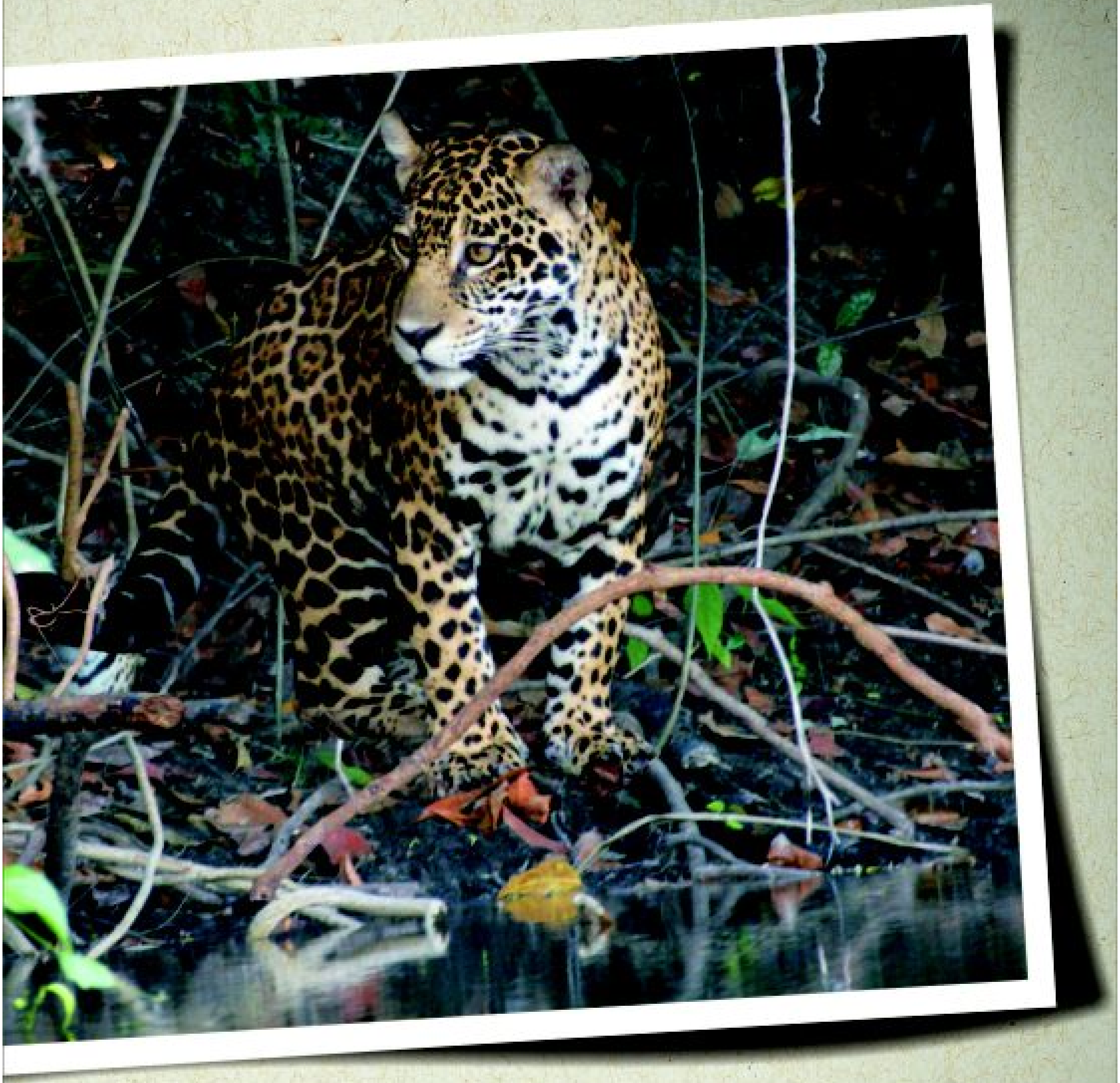

Based on the findings of this research, and through Escola da Amazonia (the School of Amazonia), the project has developed and implemented education and communication in the agricultural frontier of the Amazon region to increase the tolerance to the predation problems caused by felines and promote the coexistence between people and jaguars in the region. This Guide is part of these actions.

Escola da AMAZÔNIA
www.escoladaamazonia.org

PEOPLE & JAGUARS COEXISTENCE PROJECT

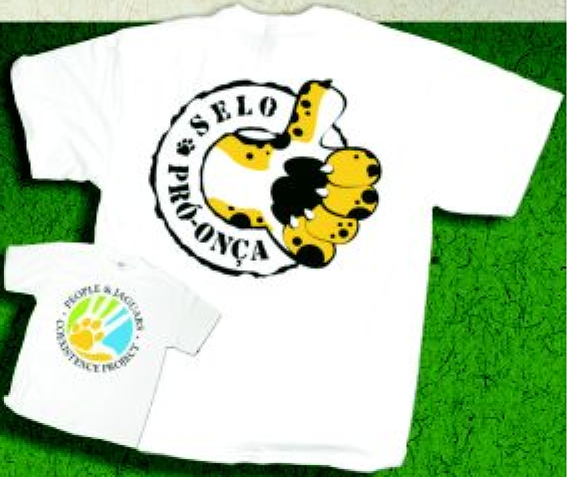


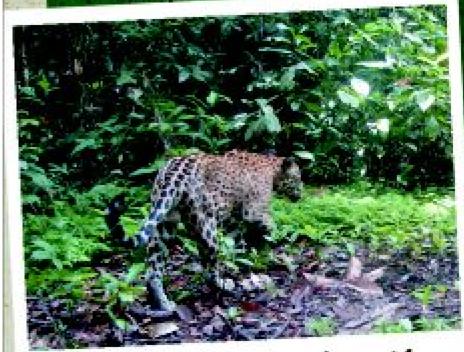

Research and education for the coexistence of people and jaguars

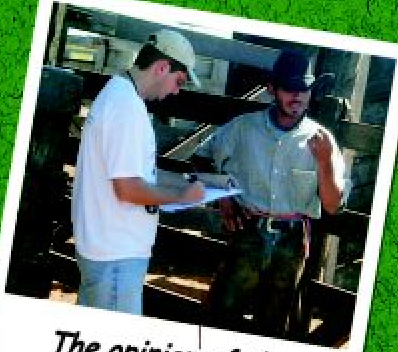

The opinion of the local community as starting point

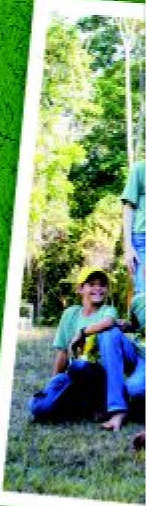

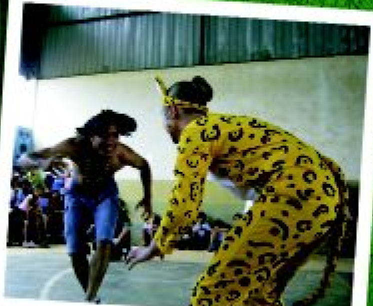

Theater Presentation: "Sassa the Jaguar"

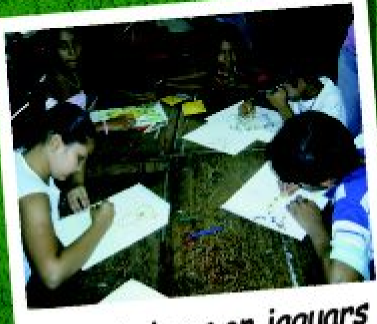

Workshops on jaguars and forests in the schools of the region

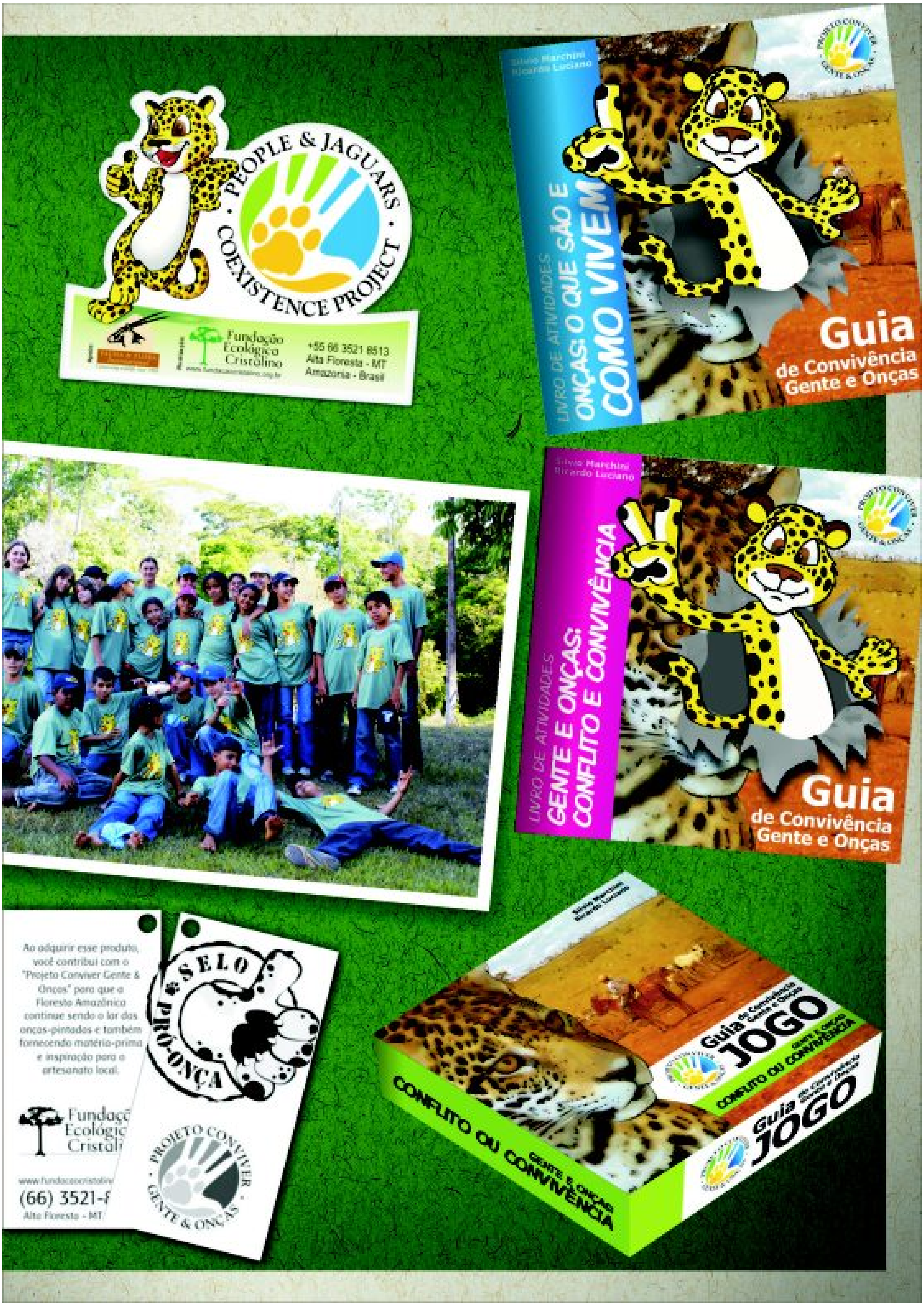
PEOPLE & JAGUARS · COEXISTENCE PROJECT ·
Fundação Ecológica Cristalino
+55 66 3521 8513
Alta Floresta - MT
Amazonia - Brasil
Silvio Marchini
Ricardo Luciano
LIVRO DE ATIVIDADES
ONÇAS: O QUE SÃO E COMO VIVEM
Guia
de Convivência
Gente e Onças
PROJETO CONVIVER GENTE & ONÇAS
Silvio Marchini
Ricardo Luciano
LIVRO DE ATIVIDADES
GENTE E ONÇAS: CONFLITO E CONVIVÊNCIA
Guia
de Convivência
Gente e Onças
Ao adquirir esse produto, você contribui com o "Projeto Conviver Gente & Onças" para que a Floresta Amazônica continue sendo o lar das onças-pintadas e também fornecendo matéria-prima e inspiração para o artesanato local.
Fundaçã
Ecológic
Cristali
(66) 3521-
Alta Floresta - MT
SELO PRÓ-ONÇA
PROJETO CONVIVER GENTE & ONÇAS
Guia de Convivência Gente e Onças
JOGO
GENTE E ONÇAS
CONFLITO OU CONVIVÊNCIA

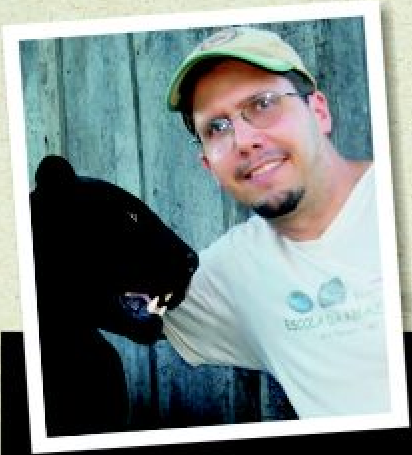

## Silvio Marchini
silvio@escoladaamazonia.org

Founder and Coordinator of the Escola da Amazonia. PhD in Wildlife Conservation with the Wildlife Conservation Research Unit (WildCRU) from the University of Oxford, United Kingdom and, MSc in Ecology with the Missouri Botanical Garden and the University of Missouri in St Louis, Missouri, United States. Conducted research on ecology and conservation of tropical forests in the reserves of the "Biological Dynamics of Forest Fragments" Project, in Manaus, Brazil, in Barro Colorado Island, Panamá, in the La Selva Biological Station, Costa Rica, and in the Luquillo National Forest, Puerto Rico. He was Academic Director of the Program Management of Natural Resources and Human Ecology in Amazonia of the School for International Training (SIT). He also worked as coordinator of the Pantanal Conservation Program for the Wildlife Conservation Society (WCS) and the Mamirauá Institute.

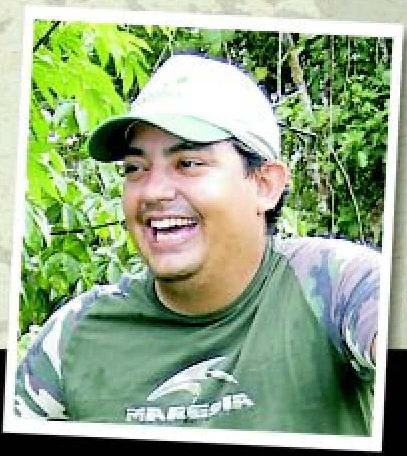

## Ricardo Luciano
osmosis3000@gmail.com

Initiated his activities in the market of the skate and surf industries in Paraná and São Paulo, where he also enhanced his techniques. He developed advertising campaigns, illustrations and traditional animations for agencies in Goiás and Mato Grosso. Looking for more knowledge in environmental communication, he began to develop works on conservation themes, working in partnership with the Cristalino Ecological Foundation, where he currently occupies the position of Coordinator of the Communication Program.

# People and Jaguars:
## a Guide for Coexistence Series

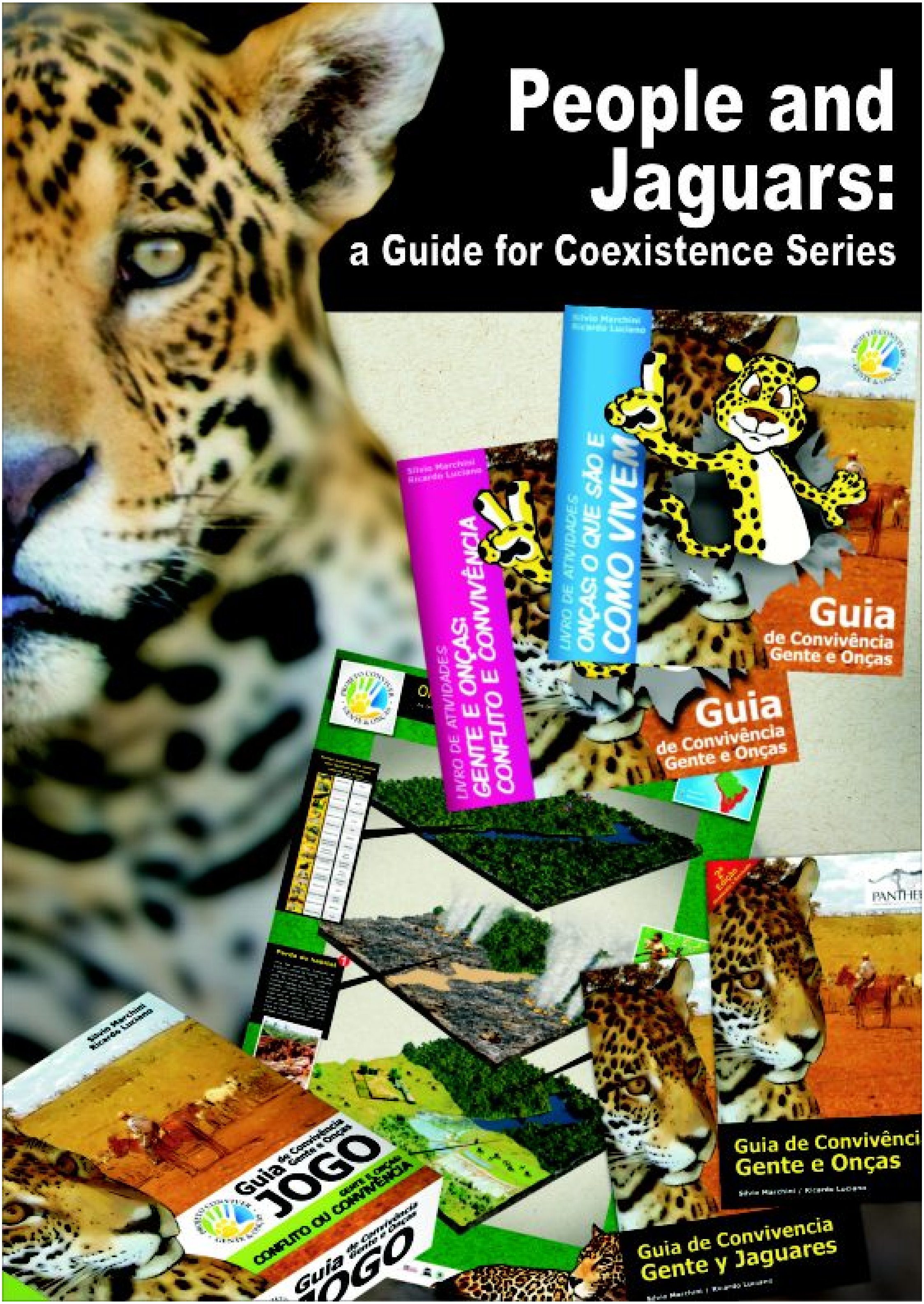

Thanks to Howard Quigley and Andrea Heydlauff for supporting the publication of this book.

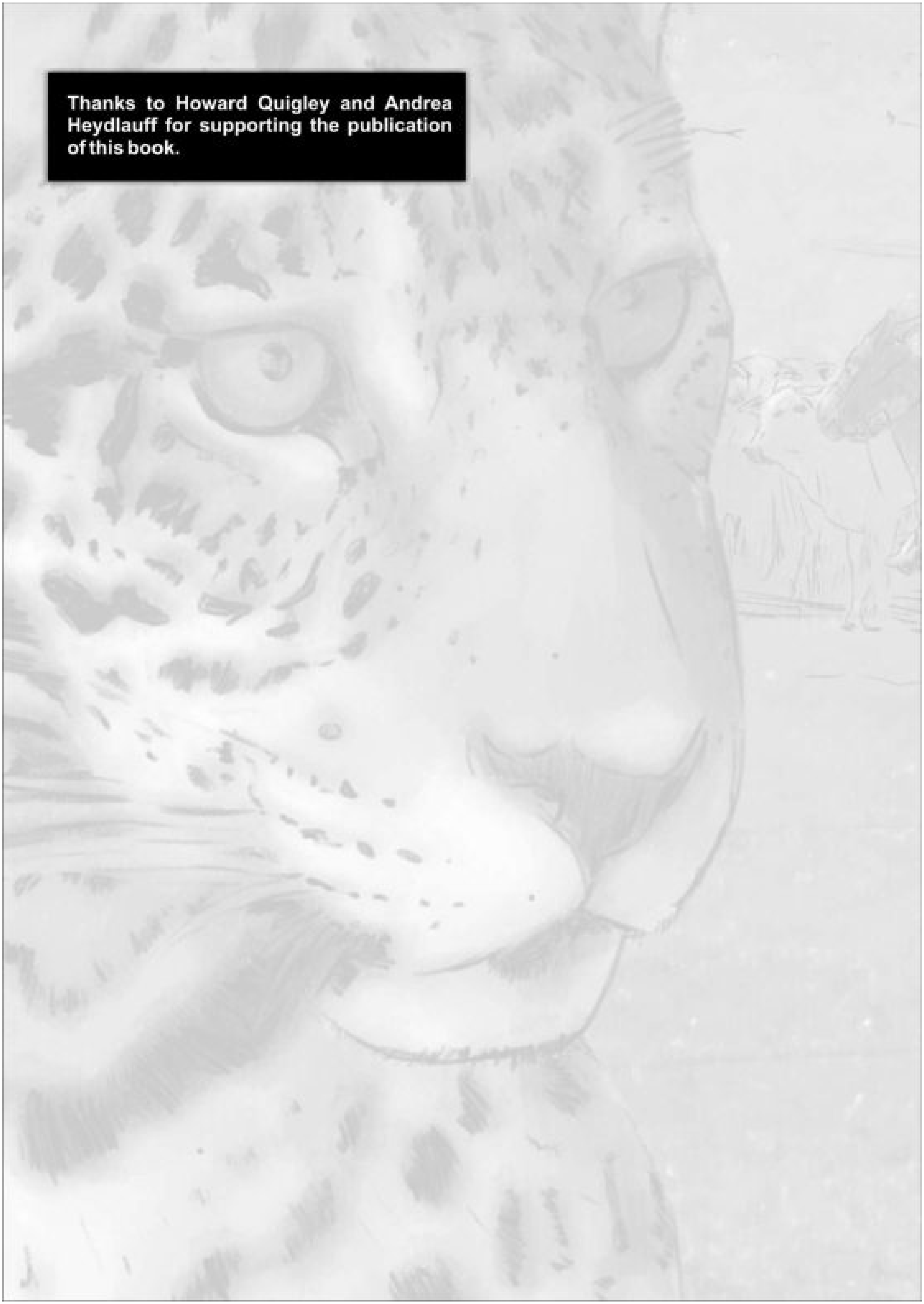

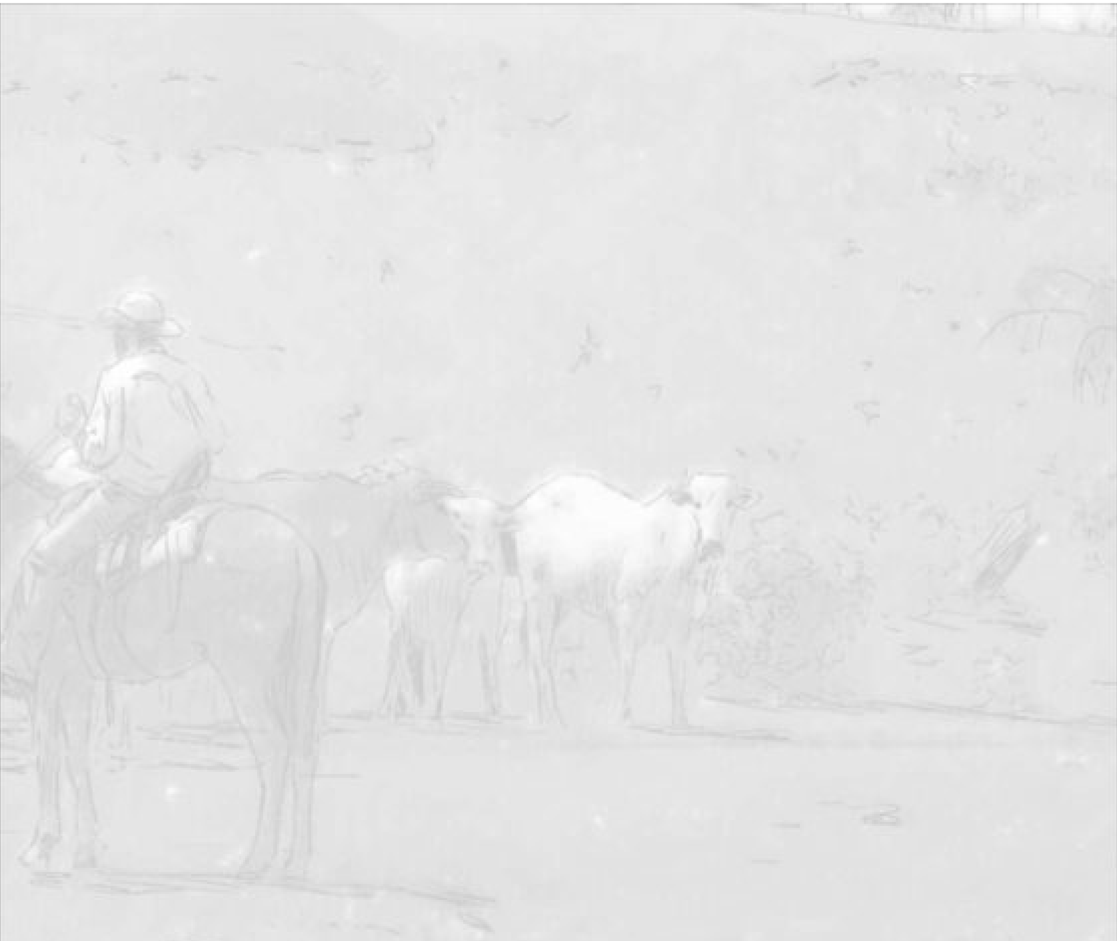

Fundação Ecológica Cristalino

Fundação Ecológica Cristalino,
Av. Perimetral Oeste, 2001
Alta Floresta, MT 78580-000
+55 (66) 3521-8513
www.fundacocristalino.org.br

People and Jaguars Coexistence Project is a cooperation between Cristalino Ecological Foundation (Fundação Ecológica Cristalino, FEC) and the Wildlife Conservation Research Unit (WildCRU). This book is a publication of the Coexistence Project and has the support of Panthera.

Cristalino Ecological Foundation was established in 1999 with the mission of promoting the conservation of biodiversity at the Amazon Agricultural Frontier in the north of the State of Mato Grosso (Brazil), particularly in the area that surrounds the Cristalino State Park. The Foundation has four principal action fronts: public policy, protected areas, research and education.

The WildCRU was founded in 1986 as part of the Department of Zoology of the University of Oxford, in the United Kingdom, and is recognized today as one of the most important institutions in the world dedicated to carnivore research and conservation. The mission of WildCRU is to develop practical solutions to conservation problems through high level scientific research.

Panthera was founded in 2006 with the singular mission of conserving the world's 36 species of wild cats – from the diminutive black footed cat of southern Africa to the massive tiger of Asia. Panthera currently focuses its range–wide conservation strategies efforts on the world's largest, most imperiled cats: tigers, lions, snow leopards and jaguars; and is planning similar global approaches for cheetahs, leopards and pumas. At smaller scales, Panthera also fosters and supports the very best conservation efforts on the world's small cat species. For more information access www.panthera.org.

Fundação Ecológica Cristalino
www.fundacaocristalino.org.br

WILDCRU
Wildlife Conservation Research Unit

PANTHERA
PARTNERS IN WILD CAT CONSERVATION

ANGLO AMERICAN

RAINFOREST CONCERN

CHESTER

INSTITUTO HSBC SOLIDARIEDADE

CLEVELAND METROPARKS ZOO

WOODLAND PARK ZOO
